# Possible Ancestral Structure in Human Populations

**DOI:** 10.1371/journal.pgen.0020105

**Published:** 2006-07-28

**Authors:** Vincent Plagnol, Jeffrey D Wall

**Affiliations:** Department of Molecular and Computational Biology, University of Southern California, Los Angeles, California, United States of America; University of Chicago, United States of America

## Abstract

Determining the evolutionary relationships between fossil hominid groups such as Neanderthals and modern humans has been a question of enduring interest in human evolutionary genetics. Here we present a new method for addressing whether archaic human groups contributed to the modern gene pool (called ancient admixture), using the patterns of variation in contemporary human populations. Our method improves on previous work by explicitly accounting for recent population history before performing the analyses. Using sequence data from the Environmental Genome Project, we find strong evidence for ancient admixture in both a European and a West African population (*p ≈* 10^−7^), with contributions to the modern gene pool of at least 5%. While Neanderthals form an obvious archaic source population candidate in Europe, there is not yet a clear source population candidate in West Africa.

## Synopsis

Determining the evolutionary relationships between modern humans and fossil hominine groups such as Neanderthals has been a question of enduring interest in human evolutionary genetics. In this paper, Plagnol and Wall present a new method for addressing whether archaic human groups contributed to the modern gene pool. Using sequence data from the Environmental Genome Project, they find strong evidence for ancient admixture in both a European and a West African population, with contributions to the modern gene pool of at least 5%. While Neanderthals form an obvious archaic source population candidate in Europe, there is not yet a clear source population candidate in West Africa. The authors' results have direct implications for the competing models of modern human origins. In particular, their estimates of non-negligible contributions of archaic populations to the modern gene pool are inconsistent with strict forms of the Recent African Origin model, which posits that modern humans evolved in a single location in Africa and from there spread and replaced all other existing hominines.

## Introduction

A long-standing controversy in the field of human evolution concerns the origin of modern humans [1,2]. The debate focuses on the relationship between various groups of archaic humans, such as Neanderthals or Asian *Homo erectus,* and anatomically and behaviorally modern Homo sapiens (i.e., modern humans). At one end of the spectrum, the multiregional model claims that modern humans evolved in concert across the Old World from various archaic groups [3]. At the other end, the Recent African Origin (RAO) model posits that modern humans evolved in a single location in Africa and from there spread and replaced all other existing hominids [4]. Currently, most but not all of the fossil evidence seems to support the RAO model [5,6]. From a genetic perspective, we can rephrase the debate in terms of what contribution archaic human populations have made to the contemporary human gene pool. The multiregional model predicts that this contribution would be substantial while the RAO model predicts that this contribution is negligible. Other models predict intermediate contributions of archaic populations to the modern gene pool [7].

The easiest way to answer this question is through a direct comparison of DNA sequences from both archaic and modern populations. Recently, researchers have managed to sequence fragments of Neanderthal mtDNA from fossil bones [8–11]. All published Neanderthal mtDNA sequences are quite different from all known modern human mtDNA sequences, and it is extremely unlikely that Neanderthals made any contribution to the modern human mtDNA gene pool. Although this observation is consistent with the RAO model, it does not prove that Neanderthals and modern humans did not interbreed—the two groups may have mixed but Neanderthal mtDNA may have been lost by the chance action of genetic drift. Subsequent studies have concluded that the data are consistent with a Neanderthal contribution of up to 25% of the modern gene pool [10,12]. A comparison of Neanderthal nuclear DNA with modern human nuclear DNA has the potential to clarify the precise genetic relationship between Neanderthals and modern humans. So far no Neanderthal nuclear DNA sequences have been determined, though recent technological advances give us the hope that such sequences may be recovered in the future [13,14].

In this paper, we take a different approach to the question. We look for signs of Neanderthal admixture by analyzing the patterns of linkage disequilibrium (LD) in contemporary human DNA sequences. Our method relies on the observation that the genetic signature of ancient admixture is so strong that even tens of thousands of years of random mating is not enough to obscure it [15]. To see this, consider the following crude approximation: at the time of (putative) admixture, extensive LD would extend across the whole genome. After 2,000 generations of random mating (40,000 years, assuming a generation time of 20 years), LD would still extend roughly 0.05 cM on average, equivalent to approximately 40 Kb, assuming 1.25 cM/Mb (cf. [16]). We look for evidence of ancient admixture in patterns of LD at intermediate distances (e.g., 5–50 Kb).

To avoid possible confounding effects, we first use extant sequence data to estimate parameters for a demographic null model that incorporates several known features of modern human history: recent population growth, a bottleneck in Europeans, and population differentiation between European and African populations [17]. Then, we introduce a new measure of LD called S^*^ which generalizes the work of [15]. We use S^*^ to test whether there has been archaic admixture in the history of modern Europeans and West Africans.

## Results

### Estimation of Demographic Parameters

Our analysis is based on 135 finished genes from the NIEHS Environmental Genome Project (EGP, as of February 2006, see [18,19] and Materials and Methods for details). We first model the recent history of European (CEPH [Centre d'Etude du Polymorphisme Humain 1980 database of people living in Utah with ancestry from Northern and Western Europe]) and Yoruba populations. We restrict our study to these two samples in order to limit the number of parameters that need to be estimated. The model must be simple enough to allow a precise estimation of its parameters yet sophisticated enough to capture the characteristics of both populations. Differences between the samples are illustrated by commonly used summary statistics (Table 1): Watterson's estimator of *θ* [20], Hudson's estimator of *ρ* [21], Tajima's *D* [22], and *F_ST_* [23]. The bottom part of the table gives the values of these statistics for our best-fitting model.

**Table 1:**
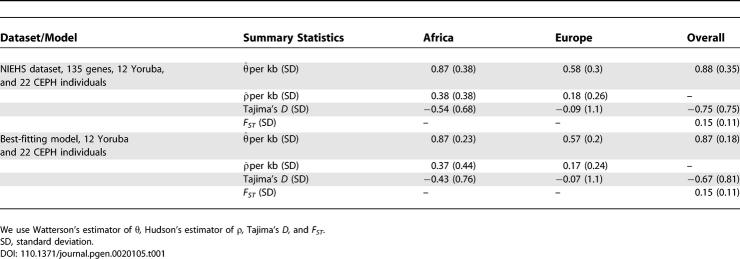
Distribution of Summary Statistics for the NIEHS-EGP Dataset and Our Best-Fitting Model

We use a simple two-island model with islands representing European and African populations. Initially, we considered models where there was no migration between the two populations after they split. These models did not fit the data well (unpublished data) so we use a model that incorporates a low level of migration between the populations. We include population growth in each population as well as a bottleneck in the European branch. We estimate a total of six parameters and the likelihood is estimated over a grid of values to find the maximum (see Materials and Methods for details). The scaling in years has been done assuming an ancestral population size of 10,000 diploid individuals (as estimated in [17]) and a generation time of 20 years. We fitted a gamma distribution to the variability of the recombination rate *ρ* to reproduce the variability observed in the Yoruba sample (see Materials and Methods).

To estimate parameters we use a composite-likelihood approach based on various summary statistics (cf. [17]). We used two sets of summary statistics. The first set consists of four statistics: Tajima's *D* in each sample, Fu and Li's D^*^ in the CEPH sample, and *F_ST_*. Tajima and Fu and Li's D^*^ measures the frequency spectrum, and *F_ST_* the level of divergence between the populations. For a given value of the parameter, the joint likelihood of these statistics is estimated by fitting to the data a multivariate Gaussian distribution. Parameters of this distribution are estimated using Monte Carlo simulations.

The second set of summary statistics divides the SNPs at a locus in three categories: private in the CEPH sample, private in the Yoruba sample, and segregating in both samples. Sites segregating in both samples are subsequently divided between low and high frequency (we set the threshold at 10%). SNPs segregating in both samples and at low frequency are characteristic of recent migrations and help to estimate this rate. This set of statistics has the useful property that the joint distribution can be computed exactly for a given realization of the genealogical process (Ancestral Recombination Graph [ARG] [24]). The final likelihood is then averaged over a large number of ARG using Monte Carlo simulations.

Even though these two sets of summary statistics are correlated, we could not estimate their joint distribution. To estimate the overall likelihood we use a composite-likelihood approximation: precisely, we assume that both sets of summary statistics are independent.

Our approach does not provide an accurate estimate of the date of divergence between the populations. Interpreting confidence intervals is difficult because we use a composite-likelihood approach. Nevertheless, a *χ* ^2^ approximation for the composite-log-likelihood ratio provides our best estimate of the confidence interval. Using this approximation we find a lower bound at 120,000 years and no upper bound. Precisely, the goodness-of-fit for an equilibrium island model with a low rate of migration between both populations is only slightly worse than for our best-fitting model. We set the divergence date to the lower bound of the confidence interval (more consistent with our knowledge of human history) and verified that this choice does not affect qualitatively the results presented in this paper (see Discussion).

**Figure 1:**
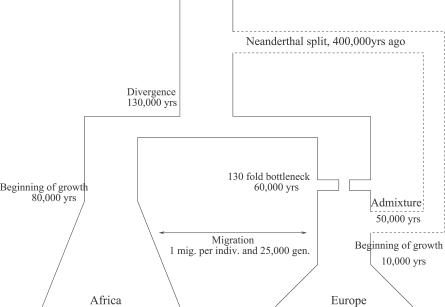
Demographic Model for European and African Populations with the Value of Our Best-Fitting Parameters DOI: 10.1371/journal.pgen.0020105.g001

Our procedure estimates that the bottleneck event is more ancient than the putative admixture event. We find that precise dating of this bottleneck is difficult because beyond 50,000 years, a change in the date of the bottleneck has very little effect on the pattern of polymorphism. The parameters of this model are presented in Figure 1 and average values of commonly used summary statistics are presented in Table 1. The dashed line in Figure 1 represents the potential admixture with an archaic population. We provide the likelihood profiles in Figure S1 and the associated *ms* [25] command line that generates this model in Protocol S1.

### Goodness of Fit

To be able to assess the significance of the pattern of LD, one needs to measure the goodness of fit of the model to confirm that it captures the main features of European and West African demography. We provide quantile–quantile plots between data and simulated distributions in Figures S2, S3, and S4 for the summary statistics used in the inference procedure as well as comparison of the simulated and observed frequency spectrum. These figures show that the model is mostly consistent with the data and explains well the summary statistics used in the fitting procedure. Though not directly comparable, it appears that our null model provides at least as good a fit as the demographic model proposed by [26]. However, we find some limitations in the goodness of fit. Precisely, the frequency spectrum (see Figure S4) does not fit very well: our model tends to simulate more singletons and fewer low-frequency SNPs (excluding singletons) than are observed in the Yoruba sample.

An important feature of our demographic model is that it reproduces well the ratio of the estimated recombination rate *ρ* between the CEPH and Yoruba populations. This is remarkable because no aspect of LD information was used in our fitting procedure.

### Measure of LD: S^*^

**Figure 2:**
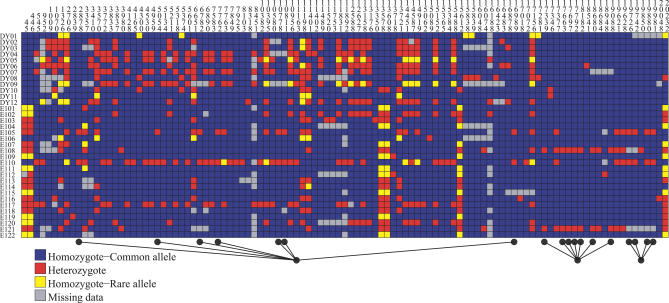
Polymorphism Data Using the Visual Genotype Display Format See [35,36] for the gene *chrna4.* The first 12 rows are Yoruba genotypes and the last 22 rows are CEPH genotypes. For the gene chrna4, S^*^_CEPH_ picks 18 SNPs divided into three congruent sets. All selected SNPs, denoted by a black dot, are segregating in the European sample and fixed in the Yoruba sample. The associated *p*-value is 0.039. DOI: 10.1371/journal.pgen.0020105.g002

We now show that we can detect a specific aspect of the level of LD that is directly affected by the level of admixture and that is not captured by the estimator of the recombination rate ρ. Our statistic S^*^ is designed to identify which SNPs are the most likely to have mutated in a putative archaic population. Typically these mutations accumulate on the same branch of the genealogical tree, generating an identical pattern of mutations called congruent sites [15]. S^*^ generalizes this concept and can extract from the sequence the largest subset of SNPs which are almost congruent, a concept that we define formally in the Methods. S^*^ is highly sensitive to ancient admixture, with higher levels leading to larger values if S^*^. We compute three different versions of S^*^. The first version uses all the available polymorphism data. However, if the admixture occurs within the European population and in the absence of migration (or at least at a very low level), the SNPs that originated in the archaic population must be private to the European sample. Hence, to test for a recent admixture in the European sample we need to restrict the computation of S^*^ to SNPs private to the CEPH sample. Alternatively, we only use SNPs private to the Yoruba sample when we test for admixture within the African branch. We denote these values as S^*^_All_,S^*^_Yor_, and S^*^_CEPH_. While S^*^_All_ typically captures information about the oldest and deepest branches of the genealogical tree, S^*^_CEPH_ provides information about branches internal to the European tree. These branches are expected to be the signature of an ancient admixture in Europe. An illustration of what S^*^_CEPH_ does is provided in Figure 2.

To illustrate the efficiency of the method, we compare a scenario where there is no archaic population to another where the admixture level is set to 5% in the European population. We assume the admixture occurred 50,000 years ago and use our best-fitting model, with and without admixture. We simulate 40-kb regions and use the recombination and mutation rates that were estimated from the EGP data.

**Figure 3:**
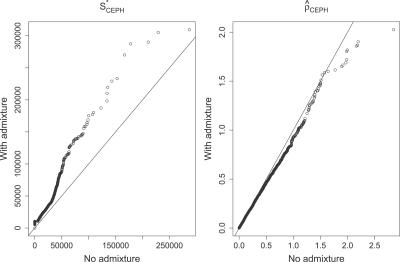
Quantile–Quantile Plot Comparing the Distribution of S^*^ (Left Graph) and the Recombination Rate ρ^_CEPH_ (Right Graph), When There Is No Admixture (*x*-Axis) and When the Level of Admixture is 5% (*y*-Axis) Since for S^*^_CEPH_ many points are far away from the diagonal, we can conclude that the two models are easily distinguishable from each other. This would not be possible based on the distribution of ρ^_CEPH_. DOI: 10.1371/journal.pgen.0020105.g003

We show a quantile–quantile plot comparing both simulated distributions of S^*^_CEPH_ in Figure 3. Distributions of S^*^_All_ and S^*^_Yor_ are not significantly affected by this admixture (unpublished data). We find that for S^*^_CEPH_ the difference of both simulated means is 60% of the standard deviation (computed under the no-admixture hypothesis). With such values a power study shows that a *t*-test with a sample size of 110 loci would provide a power of 95% for a type I error of 5%. Hence, at least in theory, the 135 genes in the dataset are sufficient to distinguish both hypotheses.

### Distribution of S^*^ in the Data

To test the null hypothesis of no ancient admixture, we calculate S^*^_All_,S^*^_Yor_ and S^*^_CEPH_ for each of the 135 loci. We estimate a *p*-value for each locus and each statistic by running simulations under the null model described in Figure 1 and comparing the actual S^*^ values to the distribution of simulated S^*^ values.

These 135 *p*-values are then combined to test if the data are consistent with our null model (see Materials and Methods for details). We then obtain an overall statistic that measures the consistency of the data with our expectations. Under the null this statistic is distributed as *χ* ^2^ with 2*n* = 270 degrees of freedom (*n* is the number of loci in the dataset). We used various models of recombination rate heterogeneity (see Materials and Methods) to assess the robustness of our findings. Results are reported in Table 2.

**Table 2:**
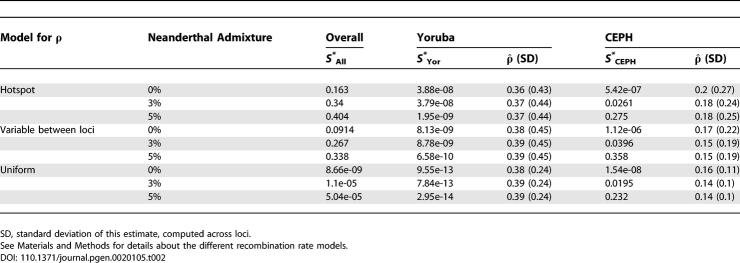
*p*-Values Associated with S^*^_All_, S^*^_Yor_, S^*^_CEPH_, and Average Values of ˆ in Each Sample for Three Different Levels of Neanderthal Admixture and Three Scenarios for Recombination Rate Heterogeneity

We first find that a constant recombination rate (within and between loci) cannot account for the distribution of S^*^_All_. This observation is consistent with the fact that the observed variability of ˆ exceeds the variance of the simulated distribution under the assumption of a uniform *ρ*. This discrepancy is associated with the strong heterogeneity of the recombination rate in the human genome: a large fraction of the loci has a lower recombination rate than the genome-wide average, generating elevated values of S^*^_All_.

**Figure 4:**
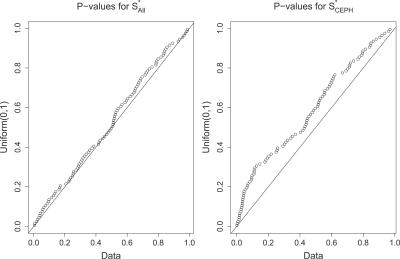
Quantile–Quantile Plot Comparing the Distribution of *p*-Values Associated with S^*^_All_ (Left) and S^*^_CEPH_ (Right) with the Expected Uniform Distribution between 0 and 1 The deviation from the diagonal line shows a discrepancy between the data and the null model for S^*^_CEPH_ but not for S^*^_All_. DOI: 10.1371/journal.pgen.0020105.g004

To account for this variability, we consider a random distribution for *ρ* fitted to reproduce the variability observed in the data (precisely by fitting the mean and the standard deviation, see Materials and Methods). This model still assumes that the recombination rate is homogeneous within a locus. We find that this model reproduces well the observed values of S^*^_All_. This consistent fit (see Table 2 and Figure 4) provides additional evidence that our null model explains the data well and that we calibrated the distribution of *ρ* reasonably.

We investigated a third model of recombination rate variability. In this model, we assume a uniform background rate and a random number of hotspots (parameters were estimated based on [27], see Materials and Methods). Using this model yields comparable results (see Table 2).

However, based on the values of S^*^_CEPH_ we find that within the CEPH sample the level of LD is higher than predicted by our model. This discrepancy is very strong and observed for all models of recombination. Even setting the recombination rate to zero does not account for the large values of S^*^_CEPH_ in the CEPH sample (*p* ≈ 0.04). This observation is also true for the Yoruba sample: values of S^*^_Yor_ found in the data are significantly higher than expected. The distribution of the *p*-values associated with S^*^_CEPH_ and S^*^_All_ are shown in Figure 4 (a list of the most significant genes in each sample is provided in Tables S1 and S2). Overall *p*-values are approximately equal to 10^−7^ for both samples, depending on the recombination model one considers (see Table 2). A complete list of the SNPs selected by S^*^_CEPH_ and S^*^_Yor_ is provided in Figures S5 and S6 (in the same format as Figure 2).

### Evidence for Ancestral Admixture

We now consider the effect of ancestral admixture on our inference procedure and show that it significantly improves the fit of our model, indicating strong evidence in favor of some form of ancestral admixture in the history of European and West African populations. We use the following approach: for different levels of admixture we reestimate the demographic parameters, and investigate if the newly estimated demography is consistent with the observed distribution of S^*^. One should remark that date of split and admixture with the archaic population are not estimated but chosen to correspond to plausible values for putative Neanderthal admixture.

We first observe that the level of admixture in Europe has little effect on the average ˆ in the European sample. Table 1 shows a slight decrease of the average ˆ in the CEPH sample, which is the expected trend in the presence of admixture (higher level of LD is associated with a lower estimated *ρ*).

Second, in the presence of admixture, the estimated demographic parameters are only slightly modified (see Protocol S1 for the associated *ms* command line). Moreover, adding a 5% level of admixture significantly improves the value of the composite likelihood: the log_10_-ratio between the maximum value estimated with a 5% admixture and no admixture equals three.

Third, this putative admixture in the European sample has a limited effect on the distribution of S^*^_All_ and S^*^_Yor_. However, it increases strongly the values of S^*^_CEPH_, as shown in Table 2. A level of admixture set to 3% is still not sufficient to explain the high values of S^*^_CEPH_ observed in the data (*p ≈* 0.03). We find that approximately 5% is required to account for the distribution of S^*^.

## Discussion

We use a model-based approach to describe the history of European and West African populations. This model predicts what pattern of polymorphism to expect in the absence of ancient admixture, and how such an event would affect the data. In the absence of admixture, the comparison of the data with our model shows a clear discrepancy that can be explained by an admixture rate of 5% in the European population. Even though we cannot exclude the possibility that an alternative demographic scenario is the cause of this pattern, this aspect of the data was chosen to be the most sensitive to an admixture event.

If the signal we observe is indeed the result of an admixture event, then these results would change our understanding of the origins of modern humans. It would indicate that archaic populations such as Neanderthals must have made a substantial contribution to the modern gene pool in Europe. We observe a similar pattern for West African populations even though a clear source population has not yet been found.

While the putative source population may not be as obvious as in Europe (Neanderthals), the fossil record shows that transitional forms of Homo were widespread in Africa, even after the time of emergence of modern humans. Other genetic studies have also found evidence for ancient structure in African populations [28–30]. In two of the three studies [28,30], the divergent lineage was found only in Pygmies, which suggests that the African population source differs from the European one.

Our model was designed to be as simple as possible while still capturing the main features of human polymorphism. We assessed qualitatively its goodness of fit and we found that it fits the data well: both the statistics we fitted and also the estimates of the recombination rate in both populations are consistent with the data. Our model makes simpler and fewer assumptions about human demography than a previous study with different findings [31], and we believe this makes our estimates of significance more reliable.

Our inference procedure based on summary statistics has similarities to two previous studies [17,26]. However, our statistical approach differs as both of these studies try to minimize an ad-hoc distance between simulations and data using the mean and variance of the summary statistics computed across loci. Instead, we estimate the composite likelihood independently for each locus, which should be more informative. Moreover, [26] use ascertained markers so their results are sensitive to the particular method used to correct for the ascertainment. In addition, the scale of the NIEHS-EGP dataset is much larger than the datasets used in both of these studies: Voight et al. [17] resequenced a total of 118 kb whereas the 135 loci we analyze add up to 3,305 kb of sequences for a total of 13,460 markers (compared with 3,738 markers studied in [26]).

We found that the choice of summary statistics is very important in our inference procedure. In particular, not incorporating a statistic which measures the level of migration between European and African branches leads to a different maximum of the composite likelihood where the divergence date is much lower and the estimates of LD are biased. If we had not added this summary statistic we could not have observed this poor goodness of fit. Hence, we tried to fit in our inference procedure all components of the data which seemed relevant. However, we cannot exclude that an important feature has been missed because our summary statistics cannot measure it.

Our inference procedure cannot clearly reject an island model where both populations remain separated indefinitely with a low level of migration. Using a *χ* ^2^ approximation, the *p*-value associated with this null hypothesis is 10^−3^. This is relatively low but our composite-likelihood approach is likely to narrow confidence intervals, and given the uncertainty regarding our model (constant migration rate in particular) we cannot clearly exclude this model. However, the discrepancy between observations and expectations remain unchanged when looking at S^*^_Yor_ and S^*^_CEPH_ (*p <* 10^−7^) and this choice does not affect significantly our main findings. We note that an equilibrium island model, along with all models incorporating a substantial element of ancient admixture, is not compatible with simple forms of the RAO model.

We investigated the pattern of polymorphism of the SNPs selected by our method in both other samples available in the NIEHS dataset: Hispanics and Han Chinese. We found that 75% of the SNPs selected by S^*^_Yor_ in the Yoruba sample are fixed in the Hispanic and Chinese samples. However this number is not significantly different from other SNPs segregating in the Yoruba sample and fixed in the CEPH sample. Most SNPs selected by S^*^_CEPH_ in the CEPH sample are variable in the Hispanic (90%) and Chinese (50%) samples. These numbers also do not differ significantly from other SNPs fixed in the Yoruba sample and segregating in the CEPH sample. However, there is no clear expectation regarding those proportions because we do not precisely know where and when the admixture occurred. In addition, the pattern of polymorphism has been affected by recent migrations, in particular between non-African populations.

Some alternate explanations can potentially explain the elevated values of S^*^, including selective effects. Because natural selection tends to affect single loci, while demography affects the whole genome, we believe that considering 135 different loci allows us to capture the underlying demographic signal. Nevertheless, a strong selective sweep can generate a similar pattern of elevated level of LD. Investigating the function of the genes selected based on S^*^ did not show any significant pattern (see Tables S1 and S2 for a complete list). We also compared these genes with two genome-wide scans for selection [32,33]. We looked for our most significant genes among those analyzed in [33] and did not find significant correlations. Likewise, the ten most significant genes selected by S^*^_CEPH_ are not identified as positively selected by Voight et al. [32] in Europeans. One gene *(xrcc4)* is identified in the Yoruba sample by both studies. However, we note that we find four significant genes (*p* < 0.05 for S^*^_CEPH_) in the ADH cluster, where Voight et al. [32] find a strong signal of selection in the East Asian sample.

An advantage of our method is that in addition to showing some evidence in favor of a significant rate of admixture, we also specifically pick a subset of candidate “archaic” SNPs. The only way to be certain of the answer would be to verify that these SNPs are indeed mutated in the DNA of Neanderthal fossils. Estimating the significance of the observed pattern is difficult because modeling human demography is a complex task. However, it is likely that if there has been a significant level of admixture, the SNPs selected by S^*^ are the best available trace of this event. Such data is not available yet but technologies are currently being developed to sequence fossil nuclear DNA (cf. [13,14]). We should note, though, that fossil nuclear DNA sequencing studies may have a difficult time distinguishing between archaic human DNA sequences and modern human contaminants.

## Materials and Methods

### Dataset.

Our approach requires resequencing polymorphism data free of ascertainment bias and we based our analyses on data from the EGP. The data were generated at the University of Washington (Seattle, Washington, United States) (see [18,19]).

Our analysis requires that the ethnicity of the samples is known. 135 out of the 505 genes in the EGP dataset fit this criterion. The average length of the genes included in the study is 50 kb. The average sequenced length is 25 kb. We restricted our study to the 12 Yoruba and 22 Caucasian (CEPH) individuals. We excluded genes on the sex chromosomes. Indels as well as SNPs with more than two alleles are also excluded.

### Demographic inference.

We make several assumptions about the demographic scenario in order to limit the number of parameters to estimate. First, we assume that the population growth is 100-fold in each population. Second, we assume that the bottleneck lasts 1,000 years, and we only estimate the reduction of the population size during this period. The six parameters left to be estimated are: the date of the beginning of growth (one parameter for each population), the date of the bottleneck, and its intensity (reduction of the population size during the bottleneck), the date of divergence between European and African populations, and the migration rate after this divergence.

We use a grid for the parameter values on which we estimate the composite likelihood. The grid consists of ten values per parameter (10^6^ total) and we refined this grid several times to locate the maximum of the composite-likelihood surface. For each value of the parameters, the same set of simulations is used across different loci in the data (using the mean sequenced length). The log-likelihood is then summed across loci to obtain the final value.

The first set of summary statistics consists of Tajima's *D* in each sample, Fu and Li's *D* ^*^ in the CEPH sample, and *F_ST_*. We assume that the distribution of these parameters is multivariate Gaussian and for each value of the parameter on the grid, we estimate the vector of means and the covariance matrix using Monte Carlo simulations.

The second set of summary statistics divides the SNPs at a locus in four categories: private in the CEPH sample, private in the Yoruba sample, and segregating in both samples at low or high frequency (we set the threshold at 10%). For each branch of the ARG [24], all mutations on this branch will belong to a single category. Hence, given one realization of the ARG, one can parse this graph to estimate the probability (*f*_1_,*f*_2_,*f*_3_,*f*_4_) for a random SNP to belong to each of the four classes. Conditional on the ARG and the total number of SNPs *n,* the distribution of (*n*_1_,*n*_2_,*n*_3_,*n*_4_) is multinomial and can be obtained explicitly. By simulating a large number of ARGs and averaging the computed probabilities for each simulated ARG *τ_i_,* we obtain an estimate of the likelihood at a given locus:


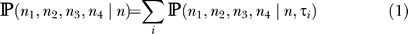


For each point on the grid, we found that 80,000 simulated ARGs are needed to obtain a precise value of the likelihood. The computation of the likelihood of the data at one point of the grid requires approximately five minutes on a 1.8-GHz Opteron processor. A significant amount of time is saved by stopping the computation after 20,000 simulations if the estimated likelihood at this point of the grid is clearly lower than the current maximum. The total computing time over the 10^6^ points of the grid takes approximately three days on 100 processors. All simulations in this paper use a modified version of *ms* [25].

### Recombination rate.

We consider various scenarios for the recombination rate. To describe the simulated distribution of *ρ,* we first estimate the average mutation rate *θ,* using all available loci, and parametrize the recombination rate *ρ* in terms of *f* = *ρ/θ*. We consider first a model where *ρ* is uniform within and between loci with *f =* 0.375.

A second model includes variability between loci but not within. Specifically, we set the distribution of *f* to be a gamma distribution with mean *μ* = 0.375 and standard deviation *σ/μ* = 0.29 ×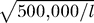 where *l* is the length of the locus we simulate (to account for a larger variability of the overall *ρ* in shorter loci). Within each simulated locus, the rate is uniform. With these parameters, the mean and the standard deviation of ˆ are consistent with observations (see Table 1).

Finally, we consider a model consisting of a background rate and a random number of hotspots. The background rate is variable with mean μ = f̄ = 0.21 and standard deviation *σ/μ* = 0.2 ×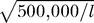 . The distribution of the number of hotspots is Poisson, whose intensity is chosen to obtain on average one hotspot per 57 kb (as estimated in [27]). Hotspots have a 2-kb width and an intensity 60 times higher than the background rate. Recombination rates for each locus are scaled so that the average overall recombination rate is proportional to the DECODE estimates [16]. With these parameters 80% of recombination events occur on average in 15% of the sequence (as estimated in [27]). Note that with this hotspot model the overall expected recombination rate must be significantly higher than under a uniform model to account for the distribution of ˆ (including hotspots f̄ = 0.65 under this model).

### Definition of S^*^.

We define S^*^ as follows:

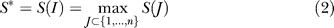
where

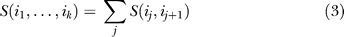
{1,…,*n*} designates the set of SNPs at this locus and *I* is the subset of SNPs that maximizes the score.

The score is computed as a sum over successive pairs of SNPs in the optimum subset *I*. Note that SNPs in *I* are not necessarily adjacent. We tried various definitions for *S*(*i*,*j*), and we chose the one that maximized the difference between our null model and the same model where the level of admixture is set to 10%.

We call the distance between two SNPs the number of chromosomes in the data at which the genotypes differ. Note that when—for a given pair of SNPs—an individual is a double heterozygote, we assume that the distance between both SNPs is zero (in other words we assume that both genotypes are in phase). If the total distance (summed over all individuals) between two SNPs is zero, both sites are congruent and the score is equal to the distance in bp between them plus 5,000. If this distance is greater than five, the score is set to −∞. If the distance is between one and five, the score is equal to −10,000. We also impose a minimum distance between sites within the optimum set *I* of at least 10 bp, to avoid contiguous and congruent SNPs (overrepresented in the human genome) to bias our estimates.

We also need to account for missing data. For a pair of SNPs to be congruent, we allow no more than two chromosomes with the property that a missing call at one SNP is associated with the minor allele at the other SNP. When one of the two SNPs has a minor allele frequency of two, we make this criteria more stringent and allow only one such chromosome.

The computation of S^*^ can be done efficiently using a forward–backward algorithm, sometimes called dynamic programming and typically used in the Smith-Waterman algorithm [34]. Specifically, if we define:

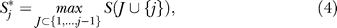
then we have the recursion:


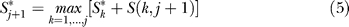


### Computation of *p*-values.

Because each locus has a different length, and different regions were not scanned for polymorphism, different sets of simulations (which reproduce these precise characteristics) are used for each locus to estimate the distribution of S^*^_All_,S^*^_Yor_, and S^*^_CEPH_. On each simulated ARG we place a number of mutations equal to the number of variable sites at this locus in the data. This is done to avoid biases due to variability in the mutation rate. Also, a random fraction of the genotyping calls is labeled missing. The probability of being missing is equal to the fraction of missing calls in the data at this locus.

For each simulated genealogical tree, we obtain a value for S^*^_All_,S^*^_Yor_, and S^*^_CEPH_ defined as 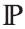 (S^*^ ≥ S^*^_data_) where S^*^_data_ is the value computed from the data. We can obtain an overall *p*-value by using the fact that if 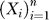 is uniformly distributed between 0 and 1 then 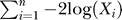 is distributed as *χ* ^2^ with 2*n* degrees of freedom.

## Supporting Information

Figure S1 Profile Likelihood for the Demographic Inference (100 KB PDF)

Figure S2 QQ-Plot between Simulated and Observed Values for the First Set of Summary Statistics (100 KB PDF)

Figure S3 QQ-Plot between Simulated and Observed Values for the Second Set of Summary Statistics (120 KB PDF)

Figure S4 Comparison of Frequency Spectrum between Data and Best-Fitting Model (64 KB PDF)

Figure S5 List of Selected SNPs in the CEPH Sample (2.8 MB PDF)

Figure S6 List of Selected SNPs in the Yoruba Sample (2.8 MB PDF)

Protocol S1 *ms* Command Lines Associated with the Best-Fitting Models (28 KB PDF)

Table S1 Most Significant Genes in the CEPH Sample (21 KB PDF)

Table S2 Most Significant Genes in the Yoruba Sample (29 KB PDF)

## Abbreviations

ARG: ancestral recombination graph
CEPH: Centre d'Etude du Polymorphisme Humain 1980 database of people living in Utah with ancestry from Northern and Western Europe
EGP: Environmental Genome Project
RAO: recent African origin
