# Supplementary material for: Possible Ancestral Structure in Human Populations
Source: PLoS Genet. 2006 Jul 28;2(7):e105. doi: 10.1371/journal.pgen.0020105 (PMC1523253; doi:10.1371/journal.pgen.0020105)
Supplement: Figure S1 — (100 KB PDF) [file pgen.0020105.sg001.pdf]

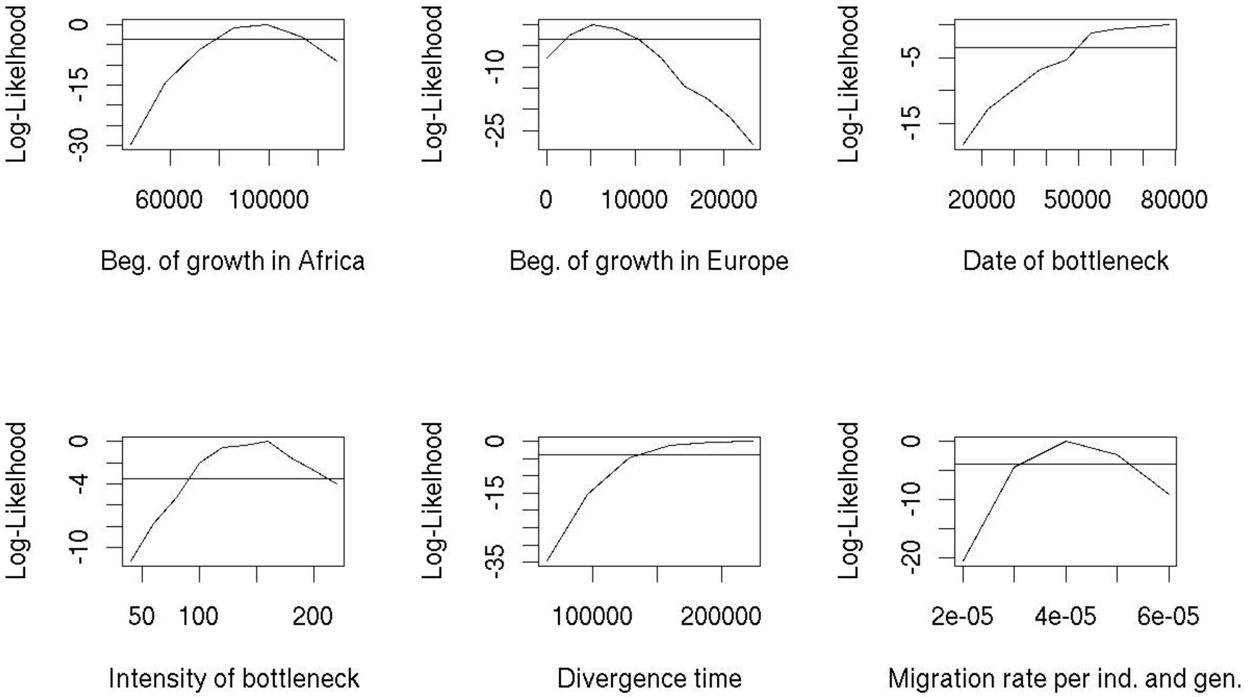

Figure 1: Profile likelihood for the six parameters estimated in our inference procedure (no admixture is assumed here). Computation limits prevent us from computing these statistics over a very dense grid. Our best estimates for the confidence interval (horizontal line) use a χ^2 approximation for the composite likelihood (threshold set to 10^{-2} in these graphs). As shown in this likelihood profile, we have little power to infer the divergence date between both populations. This date is set at 130,000 yrs.
