# Supplementary material for: Possible Ancestral Structure in Human Populations
Source: PLoS Genet. 2006 Jul 28;2(7):e105. doi: 10.1371/journal.pgen.0020105 (PMC1523253; doi:10.1371/journal.pgen.0020105)
Supplement: Figure S2 — (100 KB PDF) [file pgen.0020105.sg002.pdf]

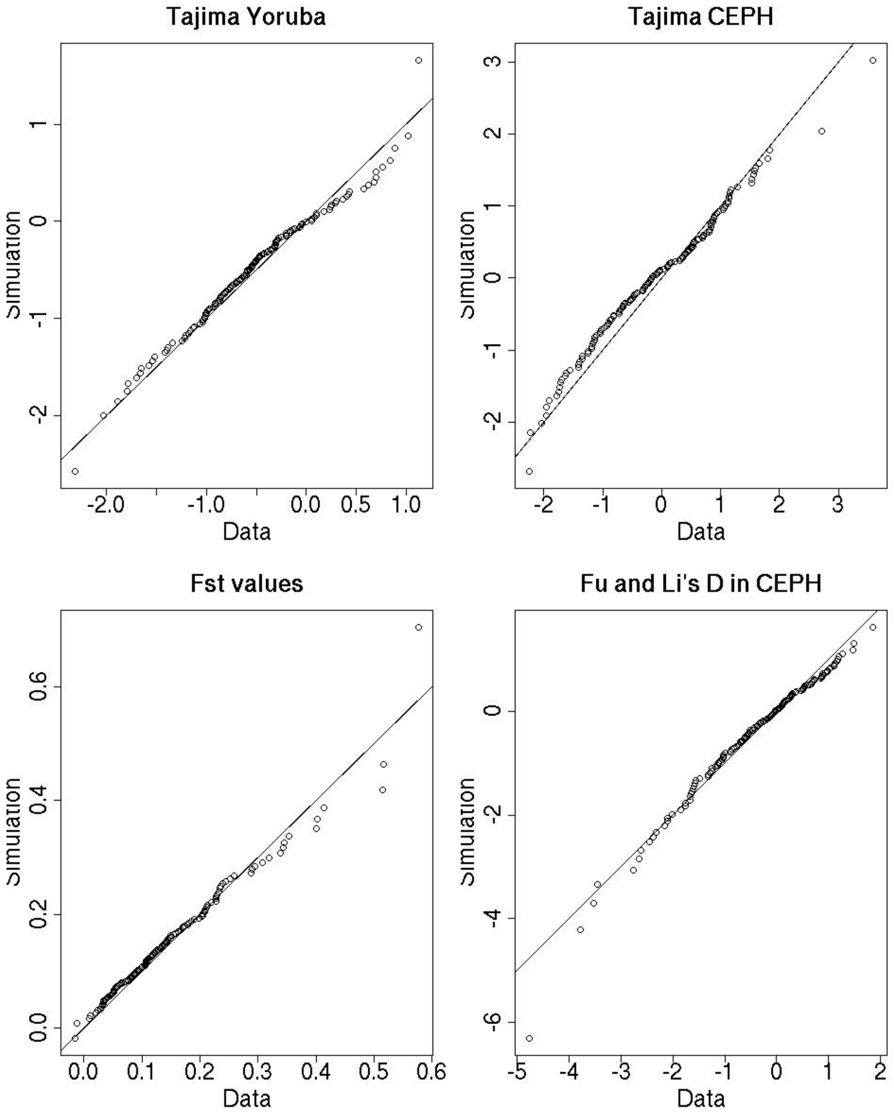


Figure 1: Quantile-quantile plot between simulated and observed values for the first set of summary statistics used in the inference procedure: Tajima's $D$ in the CEPH sample (top-left graph), Tajima's $D$ in the Yoruba (top-right graph) sample , Fu and Li's $D^*$ in CEPH (bottom right) and $F_{ST}$ (bottom left).
