# Supplementary material for: Possible Ancestral Structure in Human Populations
Source: PLoS Genet. 2006 Jul 28;2(7):e105. doi: 10.1371/journal.pgen.0020105 (PMC1523253; doi:10.1371/journal.pgen.0020105)
Supplement: Figure S3 — (120 KB PDF) [file pgen.0020105.sg003.pdf]

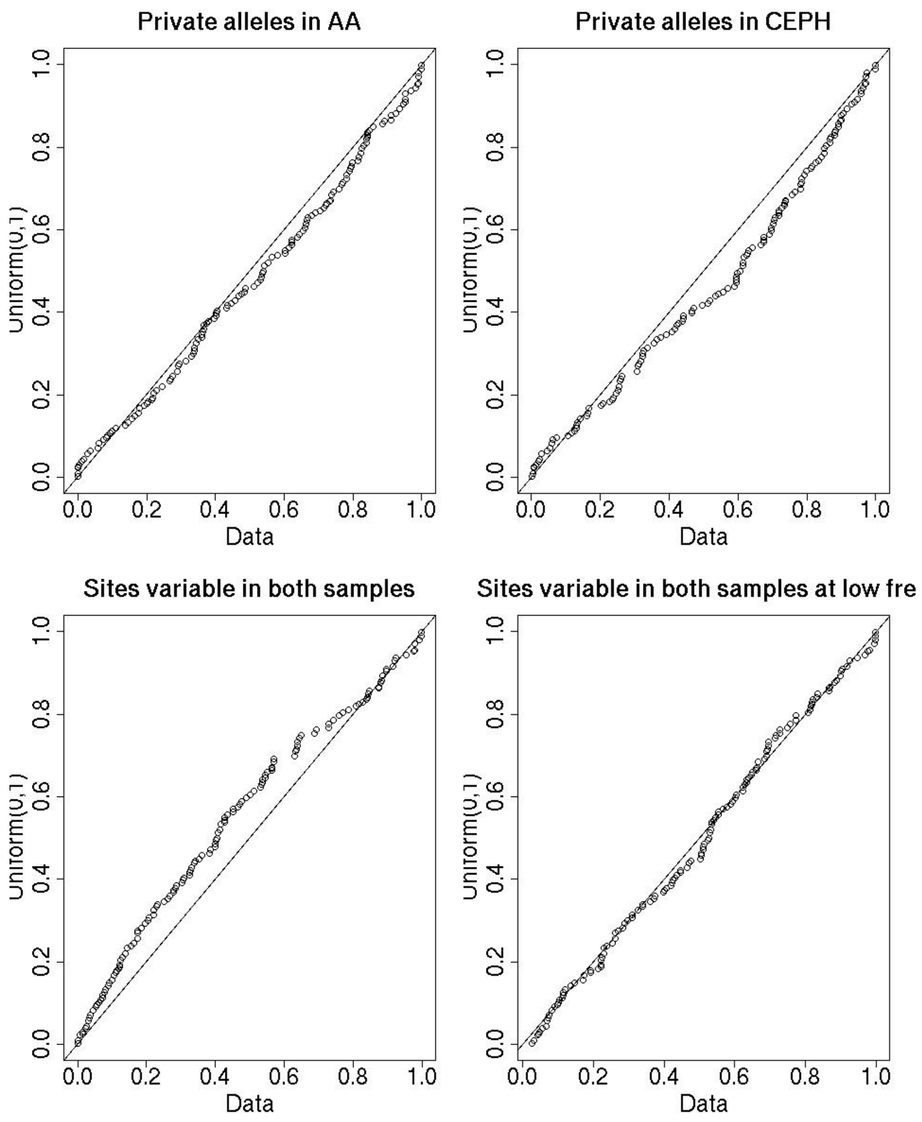

Figure 1: Quantile-quantile plot between simulated and observed values for the second set of summary statistics used in the inference procedure. The upper-left graphs shows the distribution across loci of the probability -under the null, and given the total number of segregating sites at this locus- that the number of sites private to the Yoruba sample is less or equal than what is observed in the data. It is plotted against a uniform distribution $(0, 1)$, which is the expected distribution if the null model explains the data well. The good agreement shows that the null model explains well this component of the data. The upper-right graph shows a similar graph for the sites private to the CEPH sample. The lower row shows similar graphs for the fraction of the SNPs segregating in both samples. This category is divided between SNPs at frequency higher (lower-left graph) or lower (lower-right graph) than 10%.
