# Supplementary material for: Possible Ancestral Structure in Human Populations
Source: PLoS Genet. 2006 Jul 28;2(7):e105. doi: 10.1371/journal.pgen.0020105 (PMC1523253; doi:10.1371/journal.pgen.0020105)
Supplement: Figure S4 — (64 KB PDF) [file pgen.0020105.sg004.pdf]

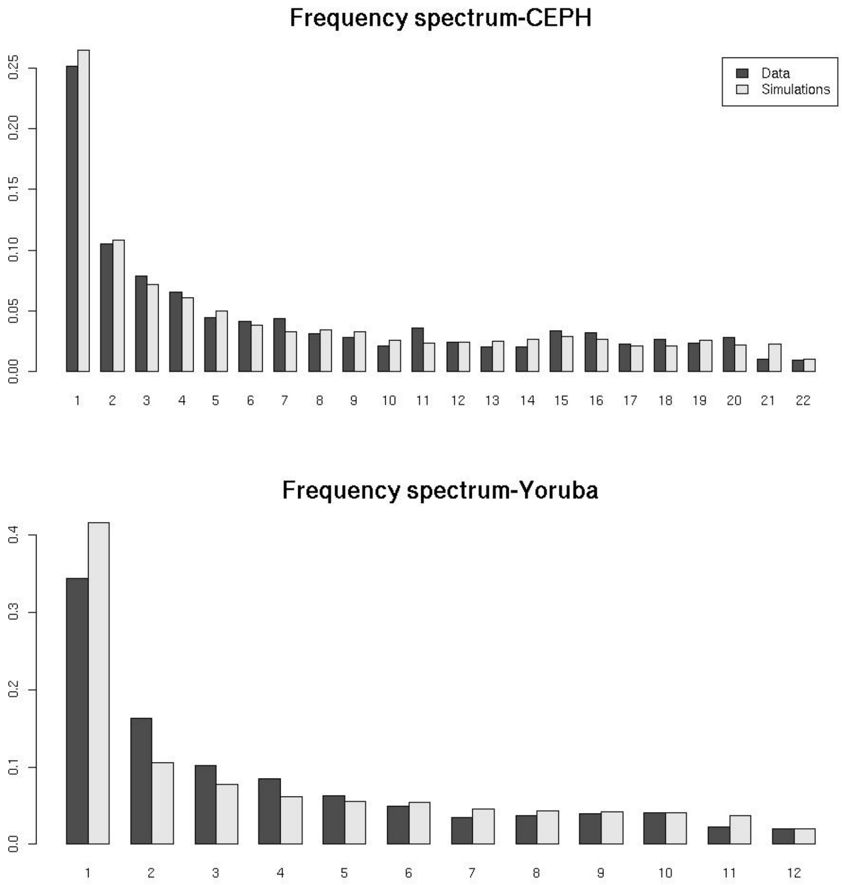

Figure 1: Frequency spectrum (European and Yoruba sample) in the data and for the best-fitting scenario (here with an admixture rate of 3%). Our best fitting scenario generates an excess of singletons in the Yoruba sample.
