# Supplementary material for: Possible Ancestral Structure in Human Populations
Source: PLoS Genet. 2006 Jul 28;2(7):e105. doi: 10.1371/journal.pgen.0020105 (PMC1523253; doi:10.1371/journal.pgen.0020105)

abcb1, p−value: 0.7356

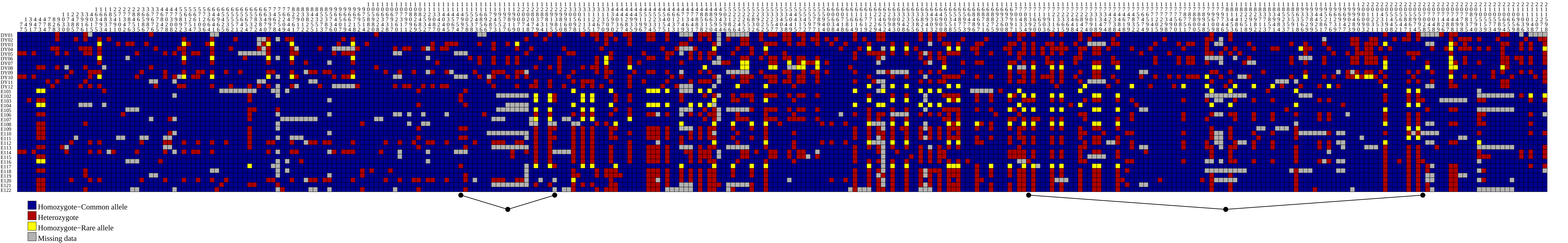

abl1, p−value: 0.9236

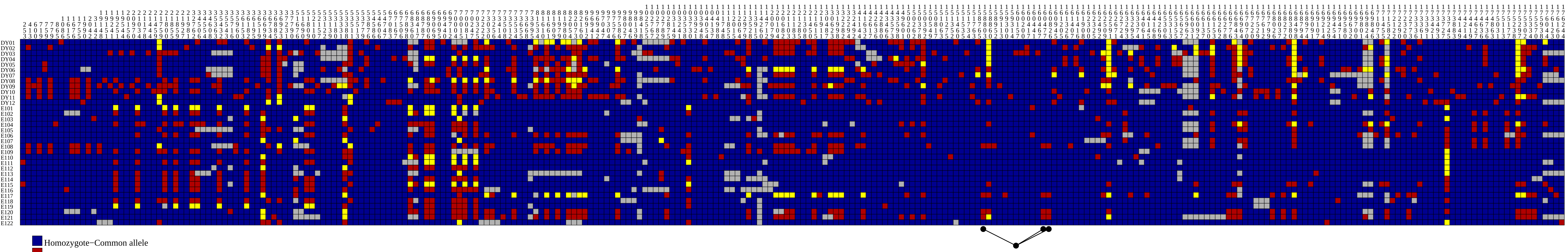

Homozygote−Common allele
Heterozygote
Homozygote−Rare allele
Missing data

abl2, p−value: 0.045

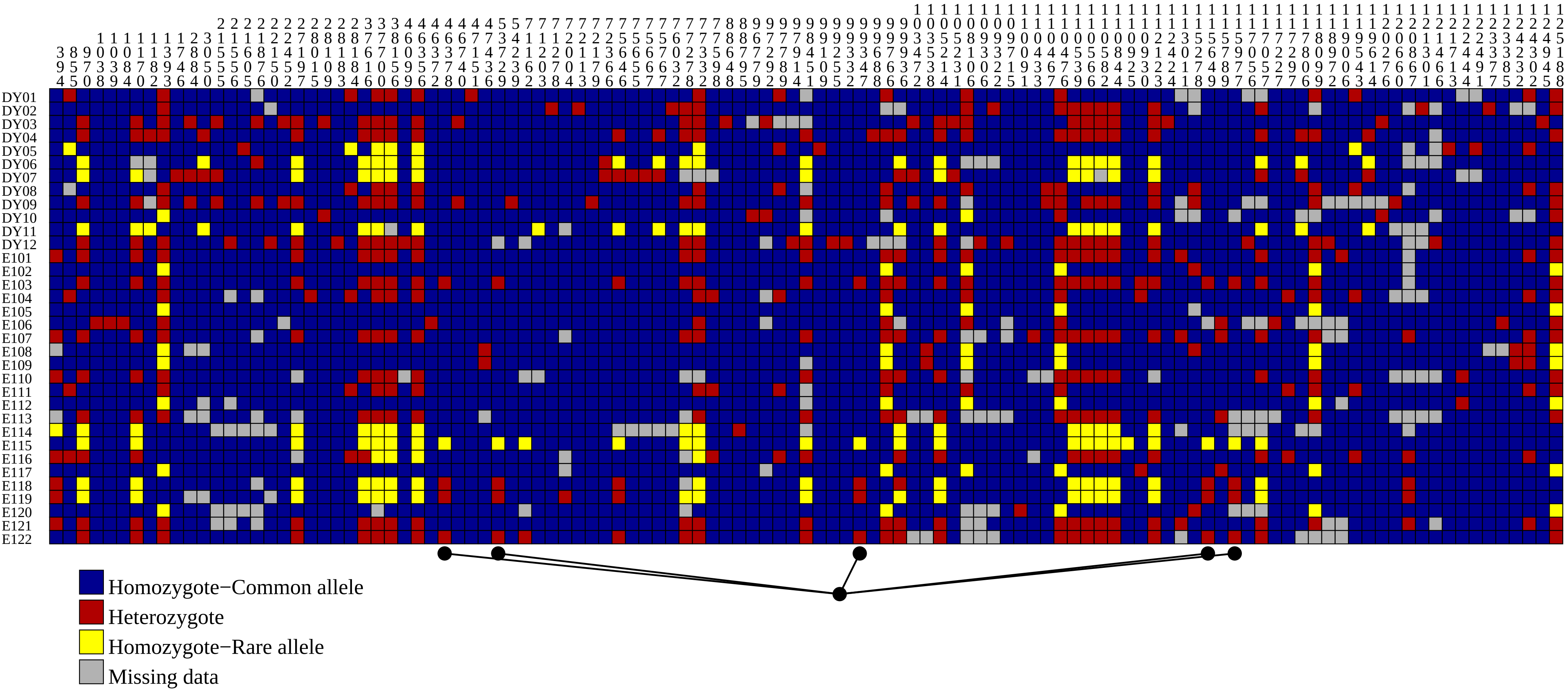

abp1, p−value: 0.6886

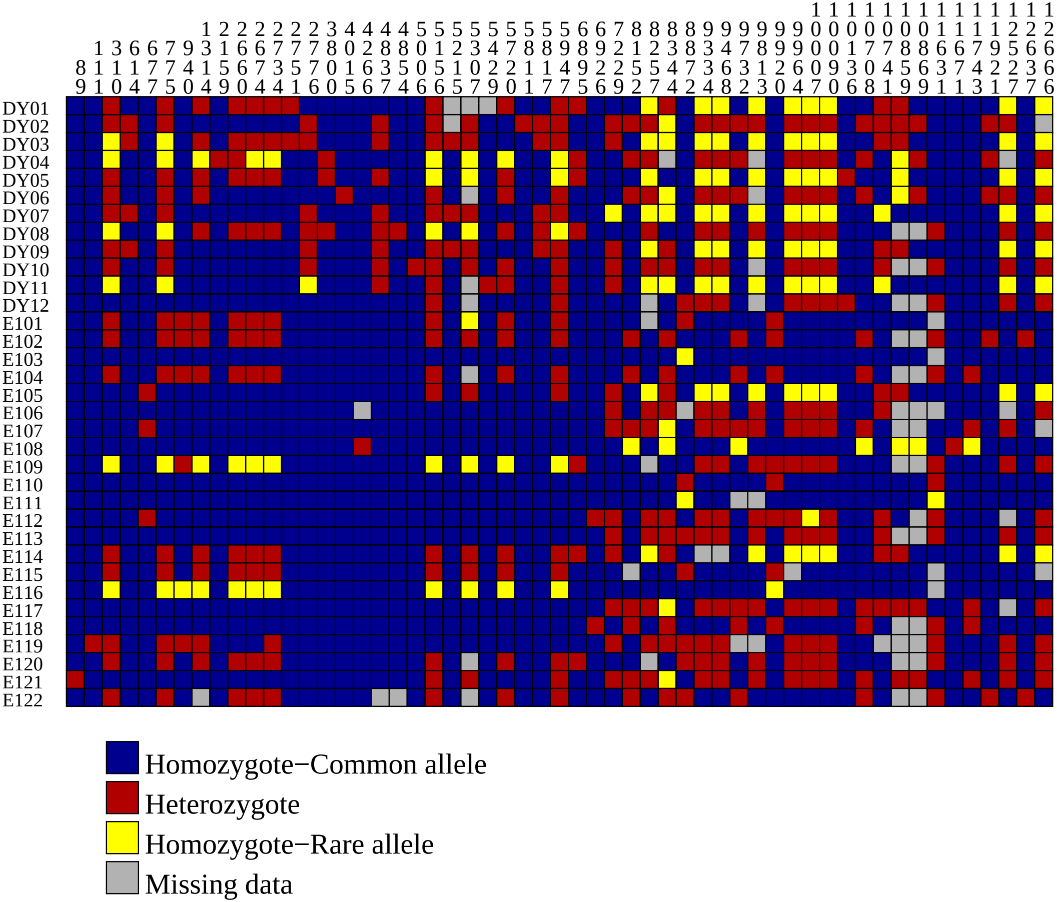

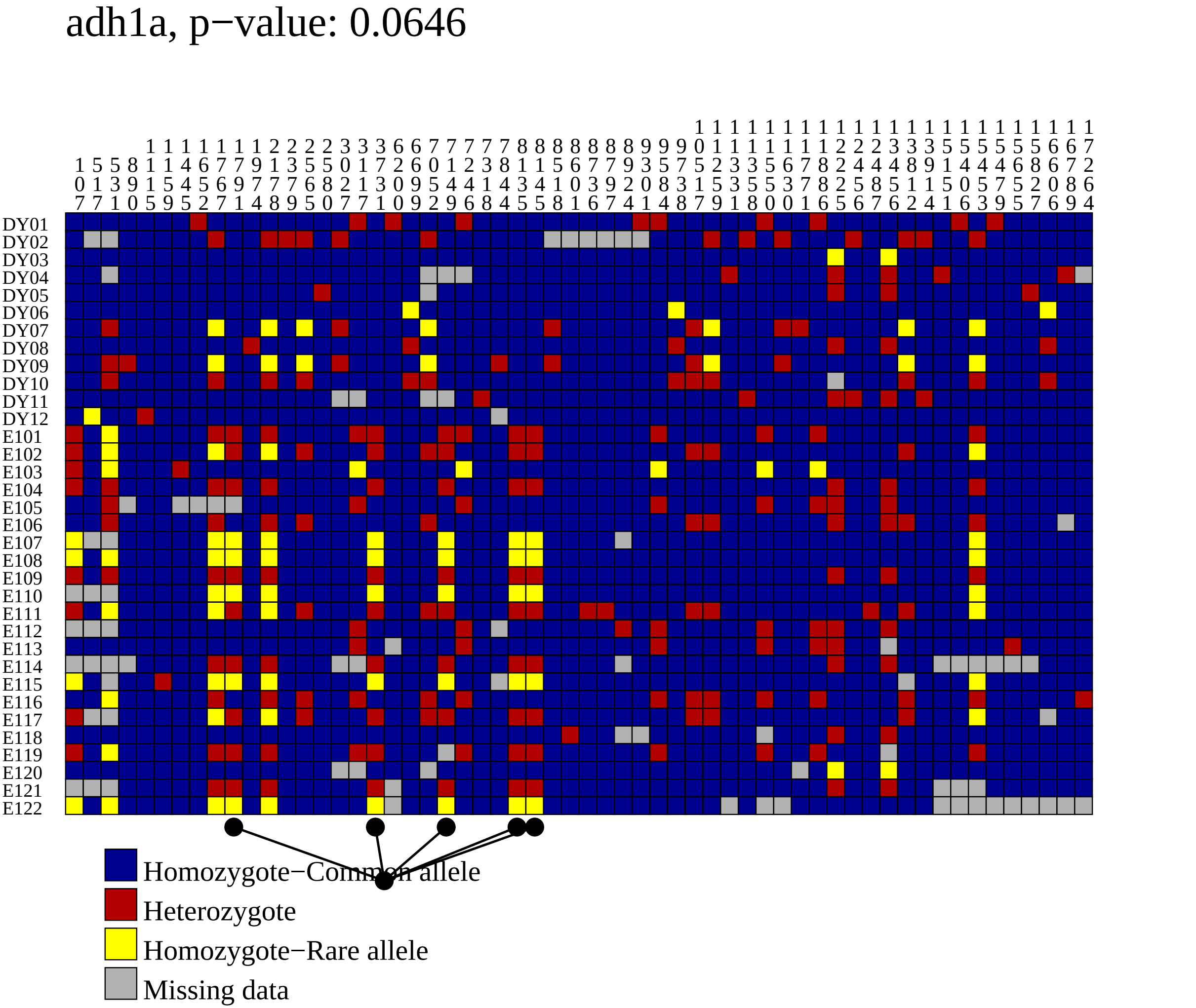
adh1a, p−value: 0.0646
Homozygote−Common allele
Heterozygote
Homozygote−Rare allele
Missing data

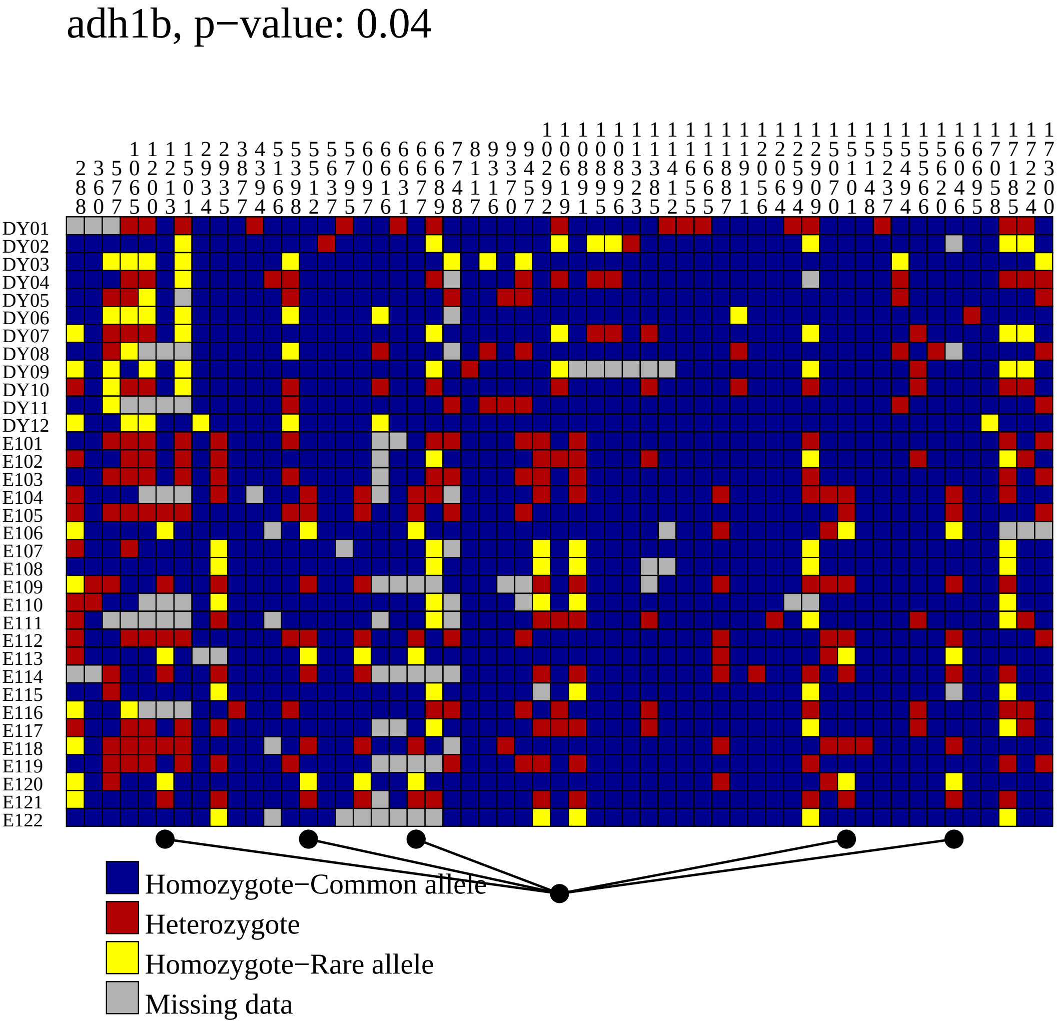
adh1b, p−value: 0.04
288
360
577
1065
1200
1213
1501
2934
2935
3837
4394
5166
5398
5512
5637
5795
6097
6616
6631
6677
6789
7748
8117
9316
9370
9457
10292
10619
10891
10895
10896
11323
11385
11412
11655
11665
11887
11916
12056
12064
12594
12909
15070
15101
15148
15237
15494
15566
15620
16046
16695
17058
17185
17724
17300
DY01
DY02
DY03
DY04
DY05
DY06
DY07
DY08
DY09
DY10
DY11
DY12
E101
E102
E103
E104
E105
E106
E107
E108
E109
E110
E111
E112
E113
E114
E115
E116
E117
E118
E119
E120
E121
E122
Homozygote−Common allele
Heterozygote
Homozygote−Rare allele
Missing data

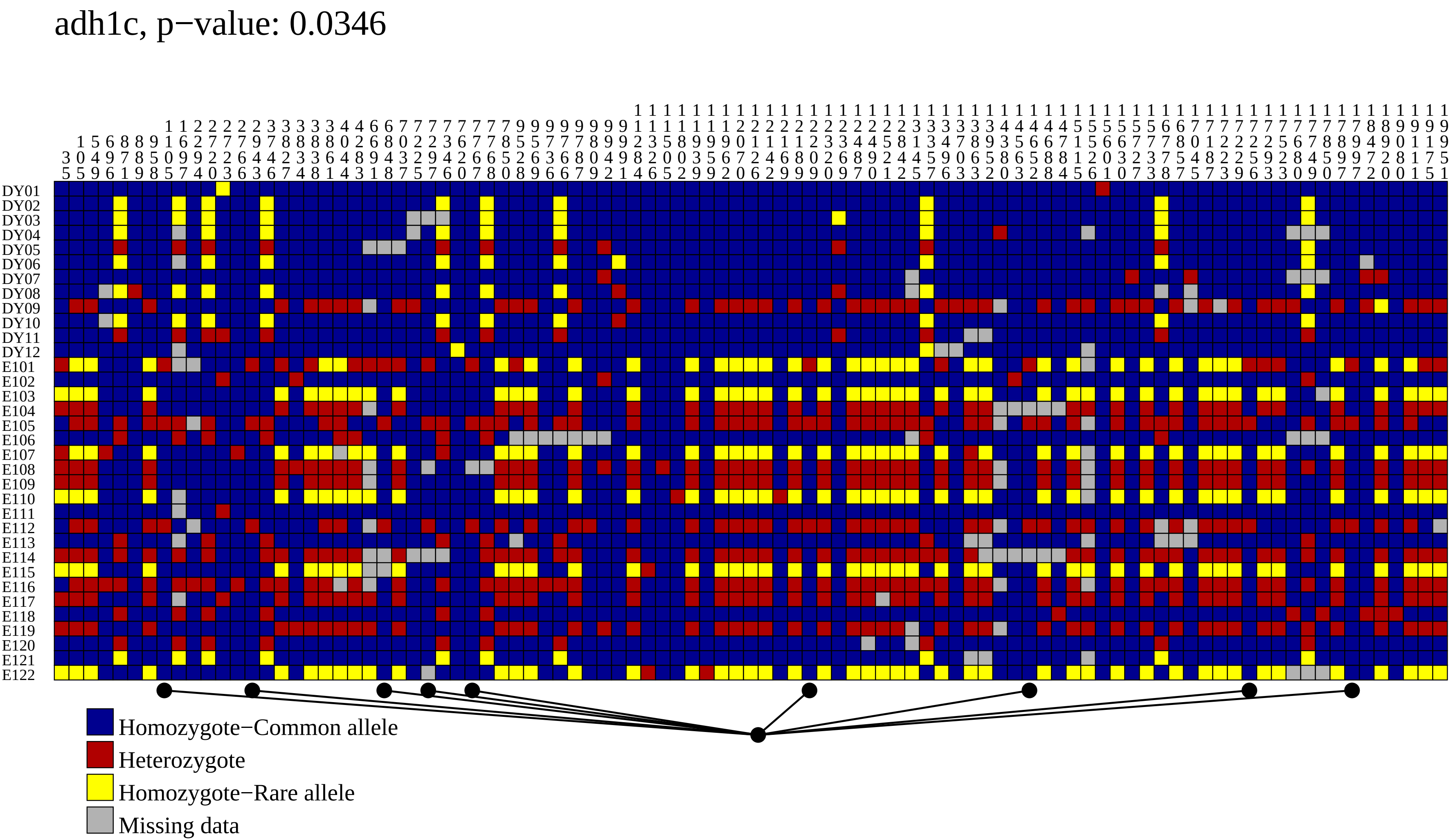
adh1c, p−value: 0.0346
Homozygote−Common allele
Heterozygote
Homozygote−Rare allele
Missing data

adh4, p−value: 0.3554

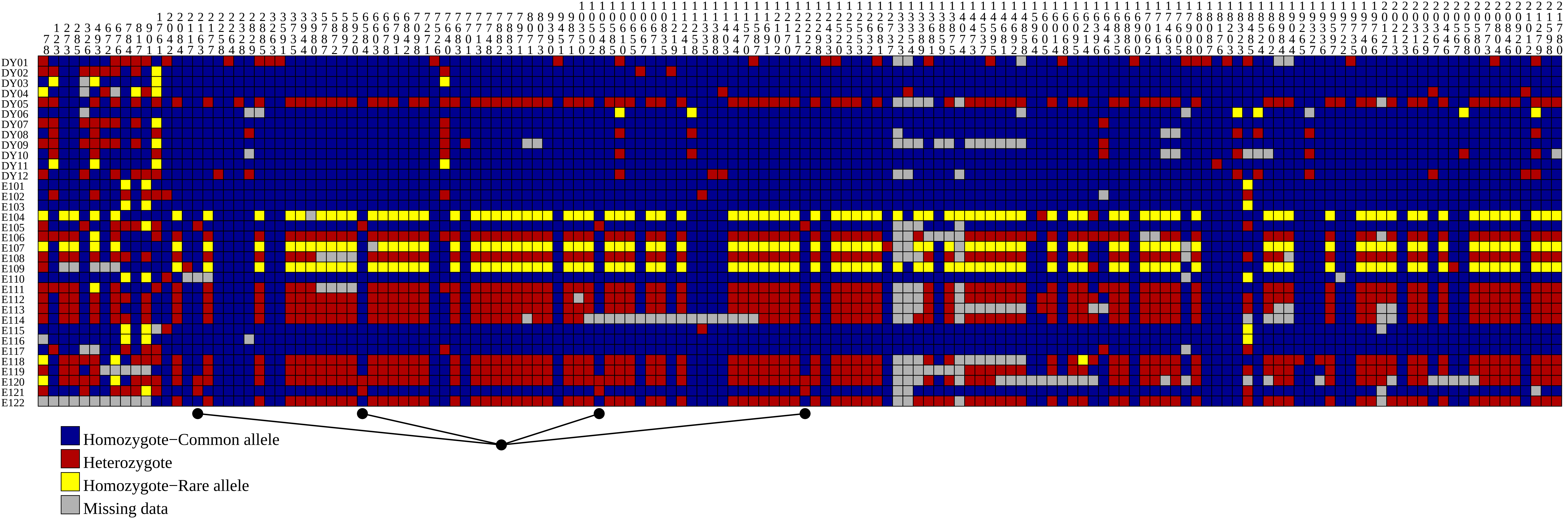

adh5, p−value: 0.0404

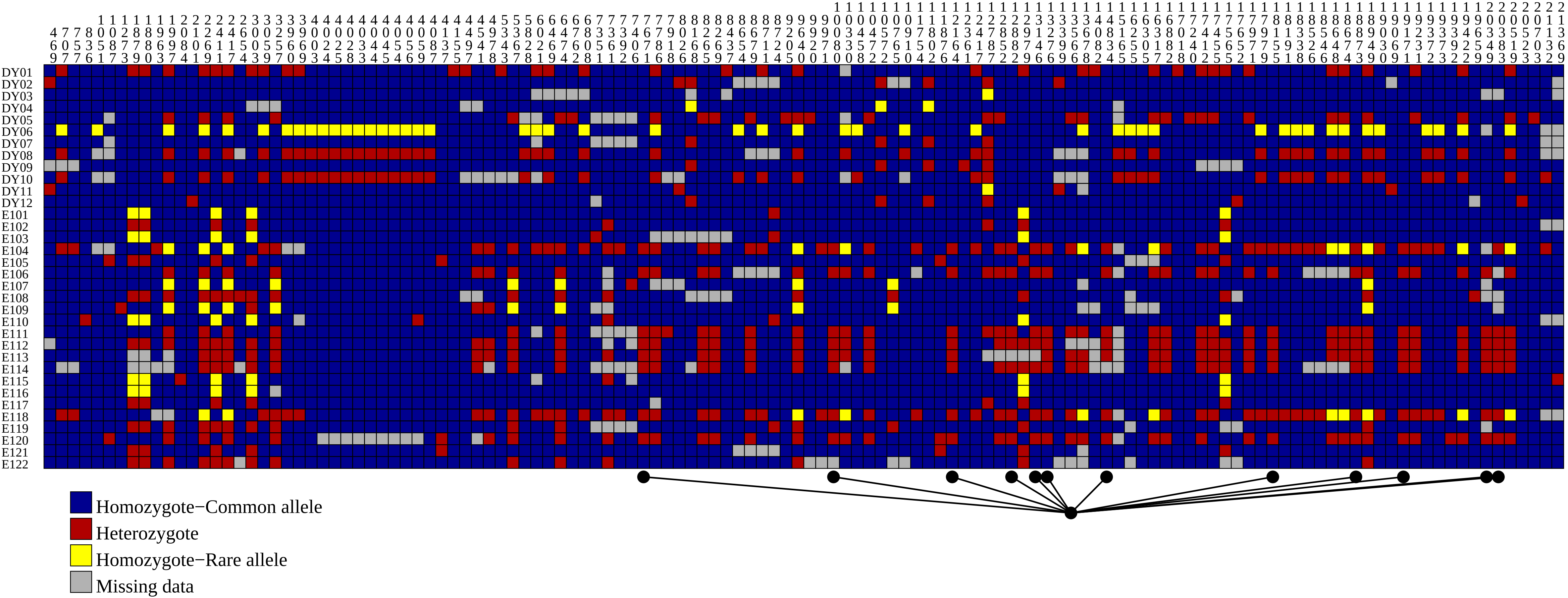

adh6, p−value: 0.042

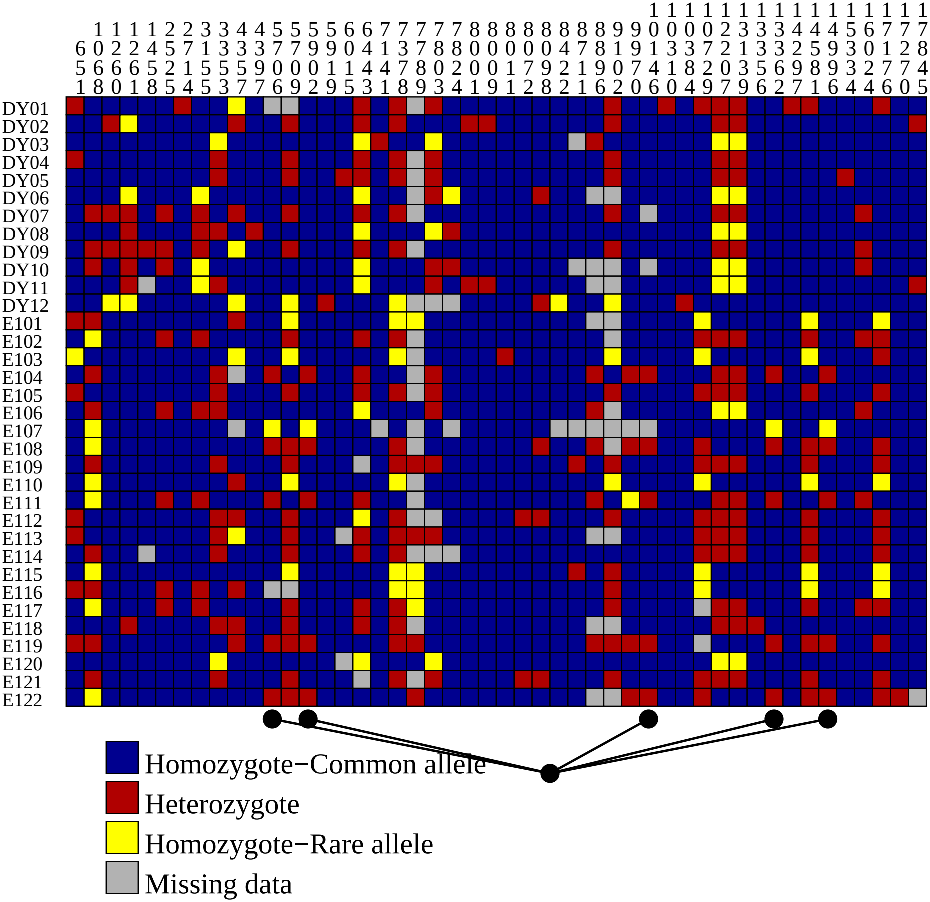

adm, p−value: 0.4336

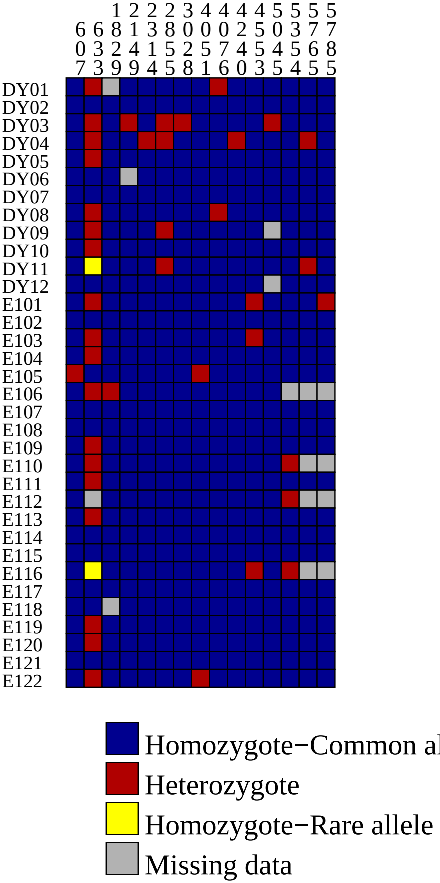

angptl7, p−value: 0.5992

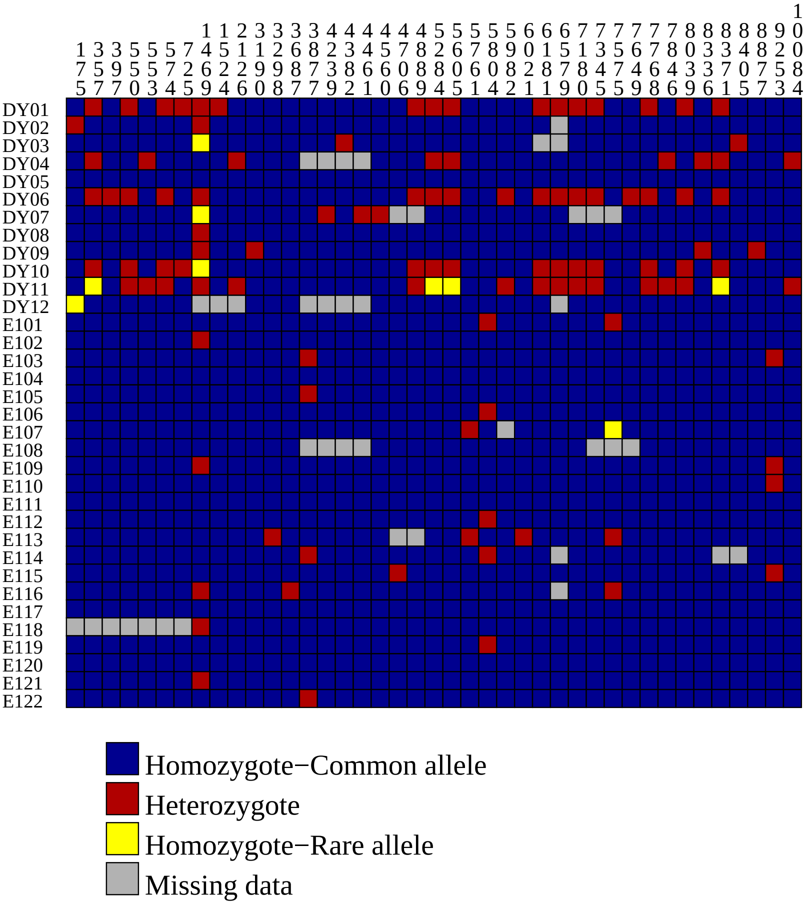

aoc2, p−value: 0.4684

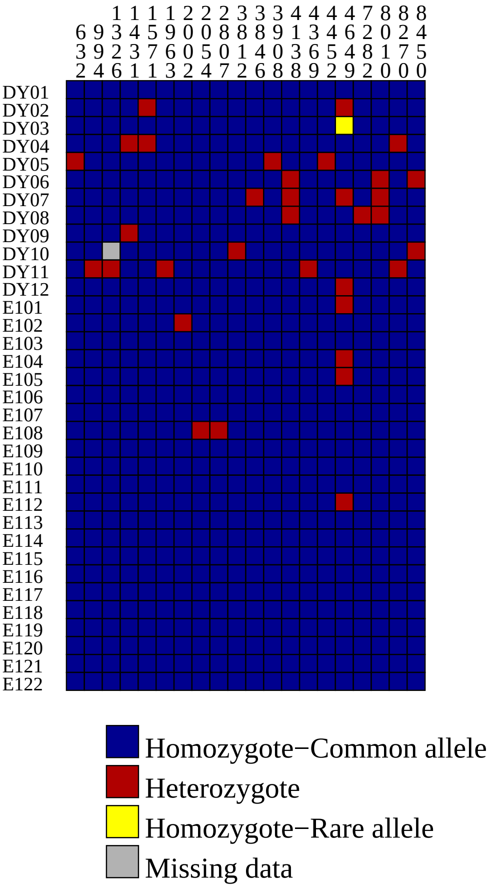

aoc3, p−value: 0.2806

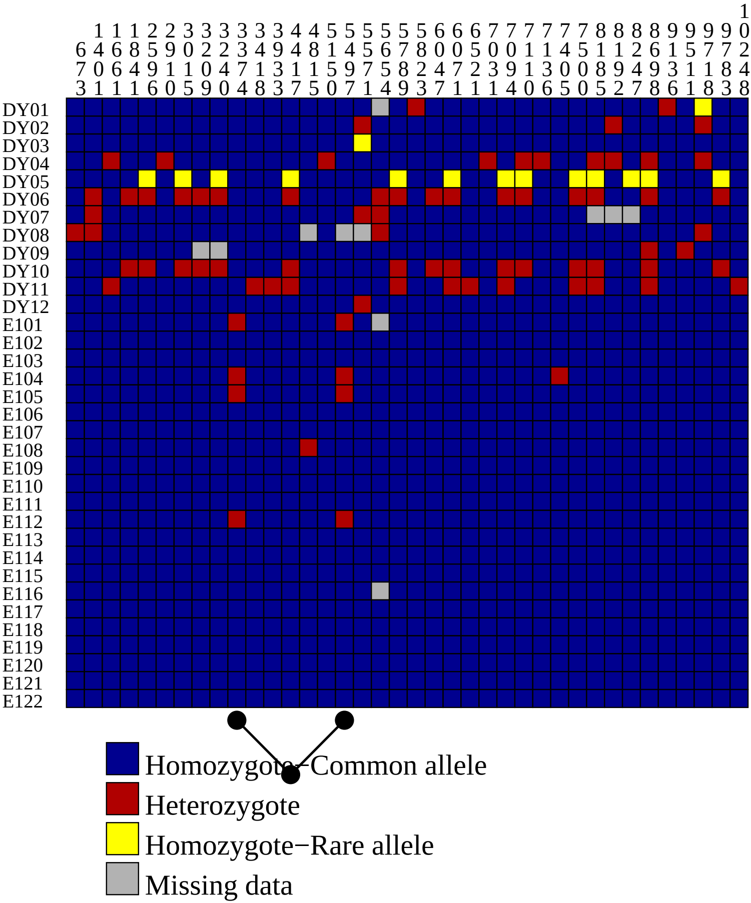

app, p−value: 0.1276

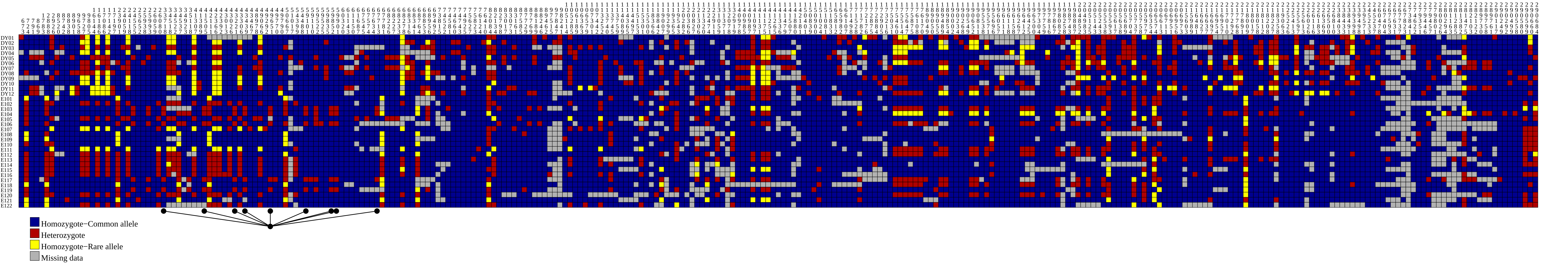

atox1, p−value: 0.7112

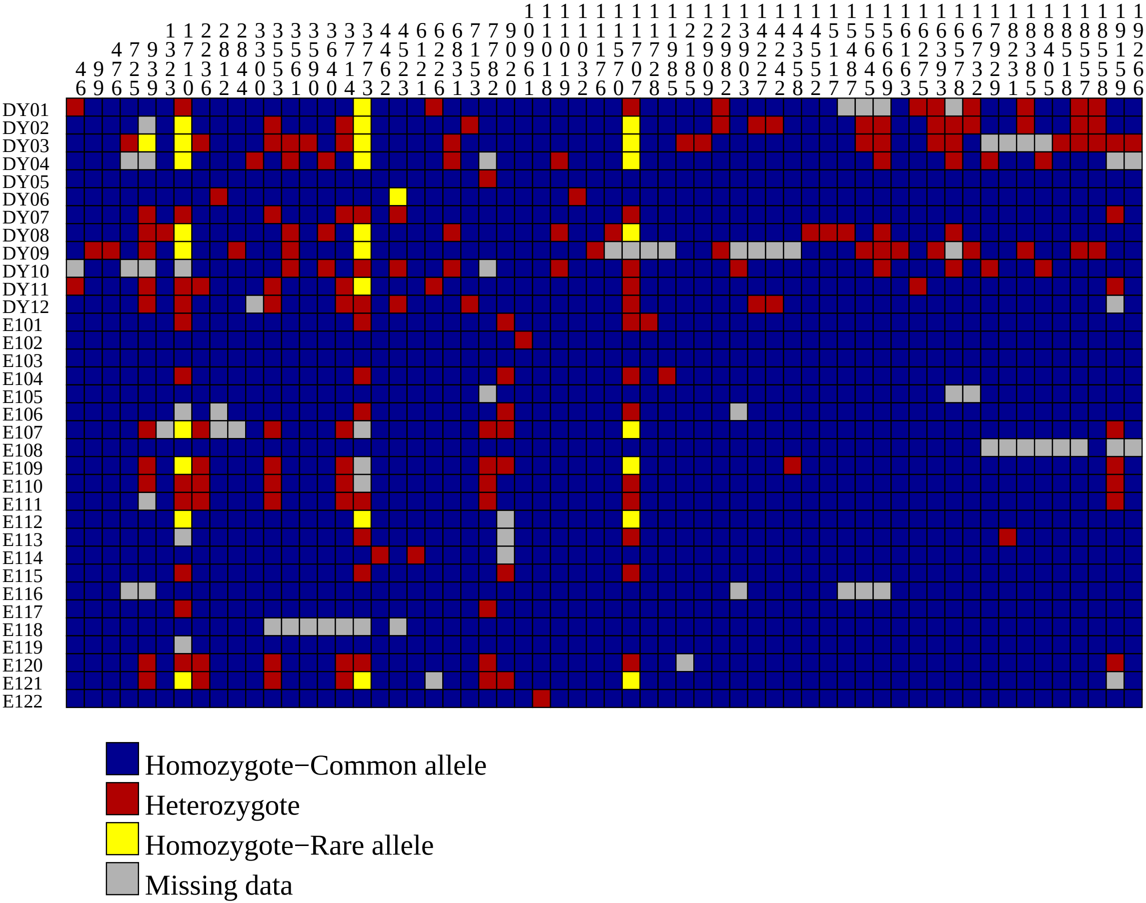

bace1, p−value: 0.0914

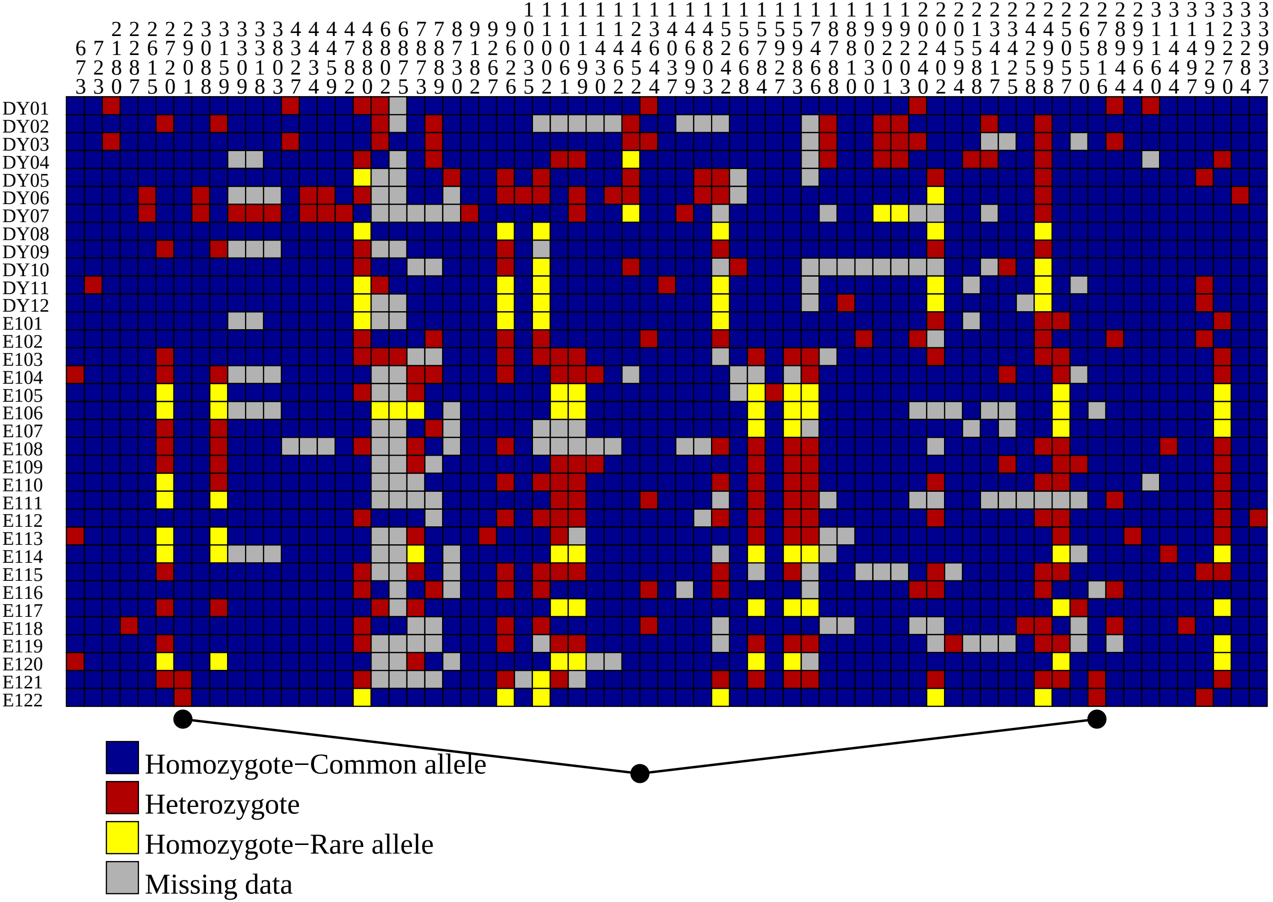

birc2, p−value: 0.0178

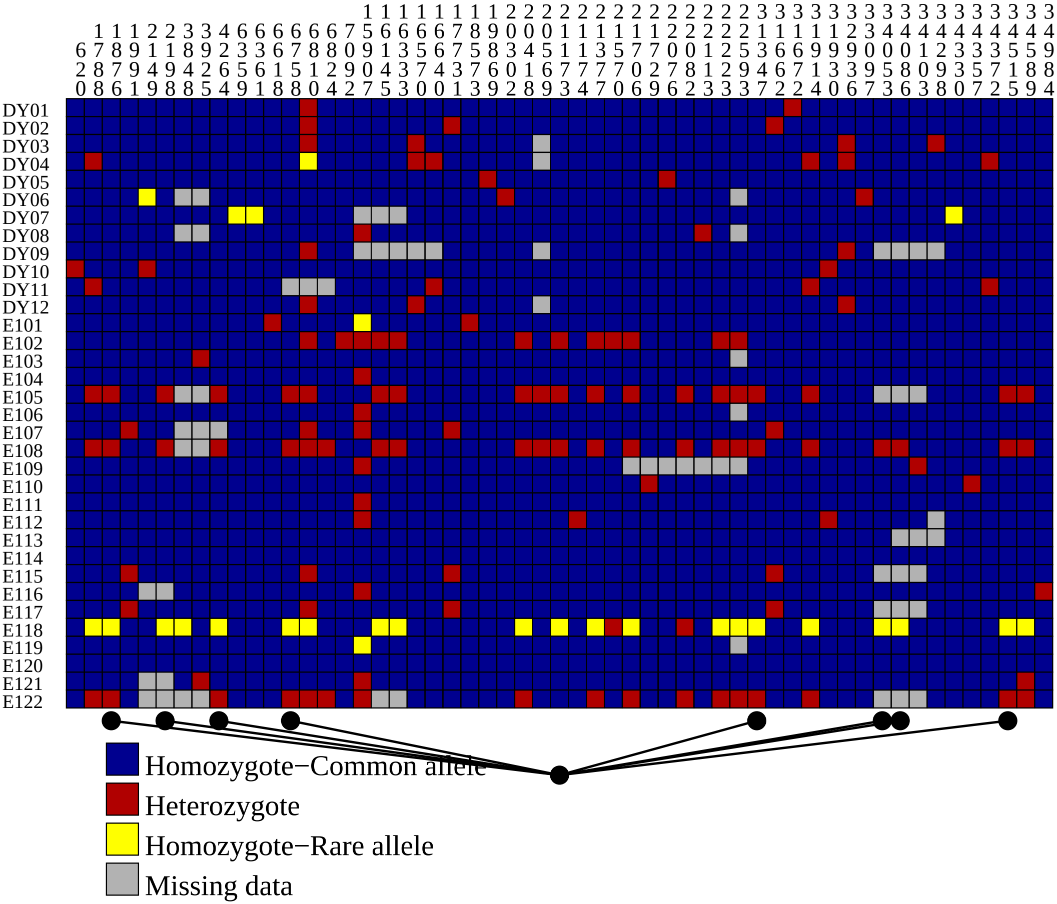

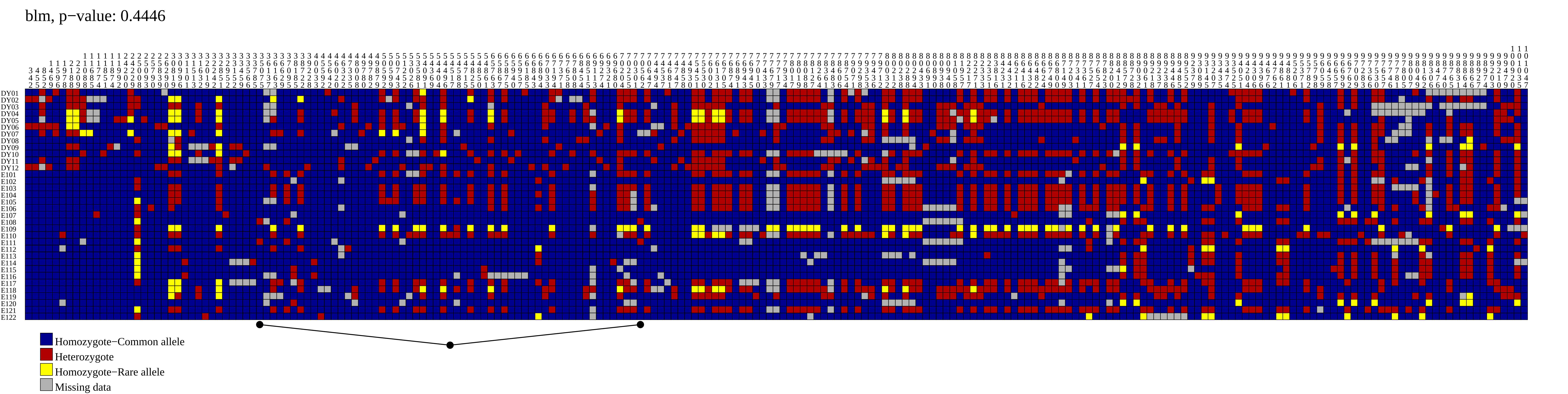
blm, p−value: 0.4446
DY01
DY02
DY03
DY04
DY05
DY06
DY07
DY08
DY09
DY10
DY11
DY12
E101
E102
E103
E104
E105
E106
E107
E108
E109
E110
E111
E112
E113
E114
E115
E116
E117
E118
E119
E120
E121
E122
Homozygote−Common allele
Heterozygote
Homozygote−Rare allele
Missing data

bnip3, p−value: 0.4528

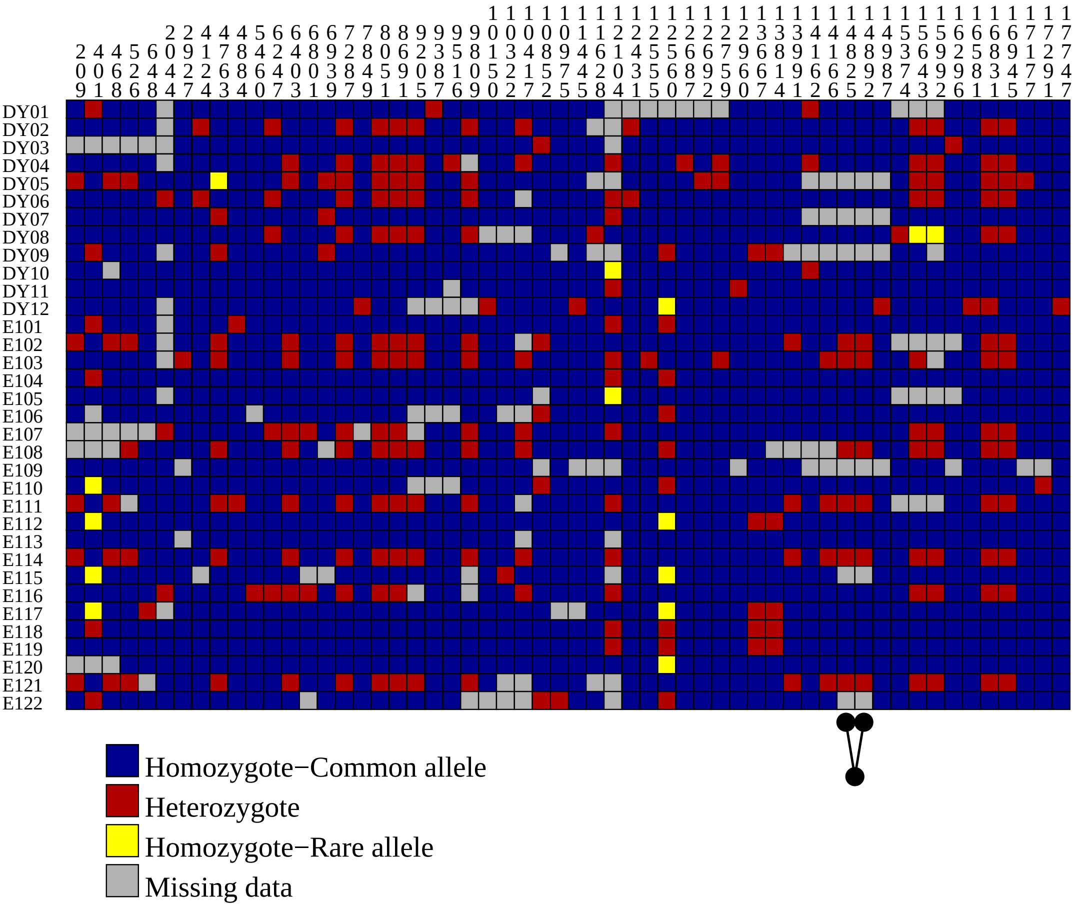

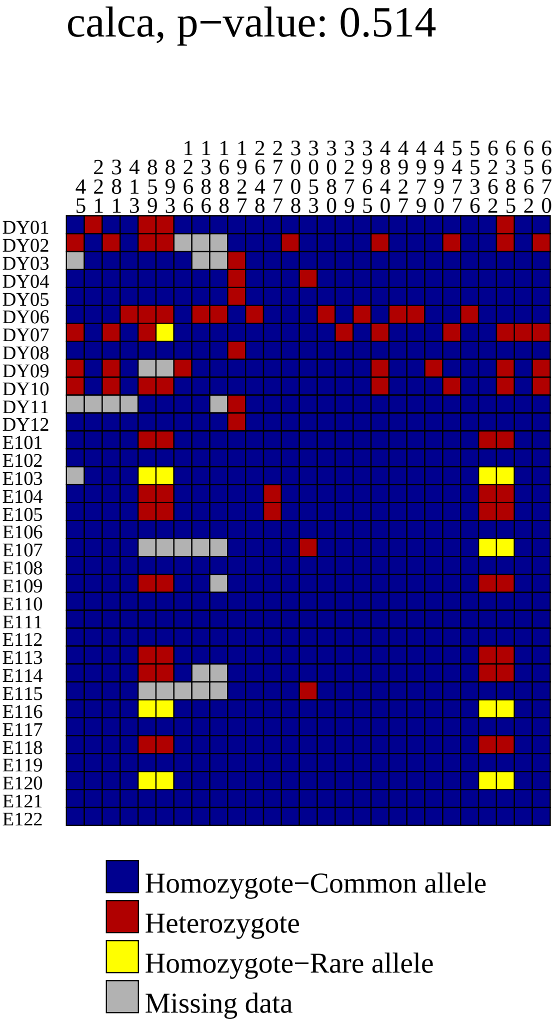

calca, p−value: 0.514
45
221
381
413
859
893
1266
1386
1688
1927
2648
2777
3008
3053
3080
3279
3965
4840
4927
4979
4990
5477
5536
6262
6385
6562
6670
DY01
DY02
DY03
DY04
DY05
DY06
DY07
DY08
DY09
DY10
DY11
DY12
E101
E102
E103
E104
E105
E106
E107
E108
E109
E110
E111
E112
E113
E114
E115
E116
E117
E118
E119
E120
E121
E122
Homozygote−Common allele
Heterozygote
Homozygote−Rare allele
Missing data

capn3, p−value: 0.7484

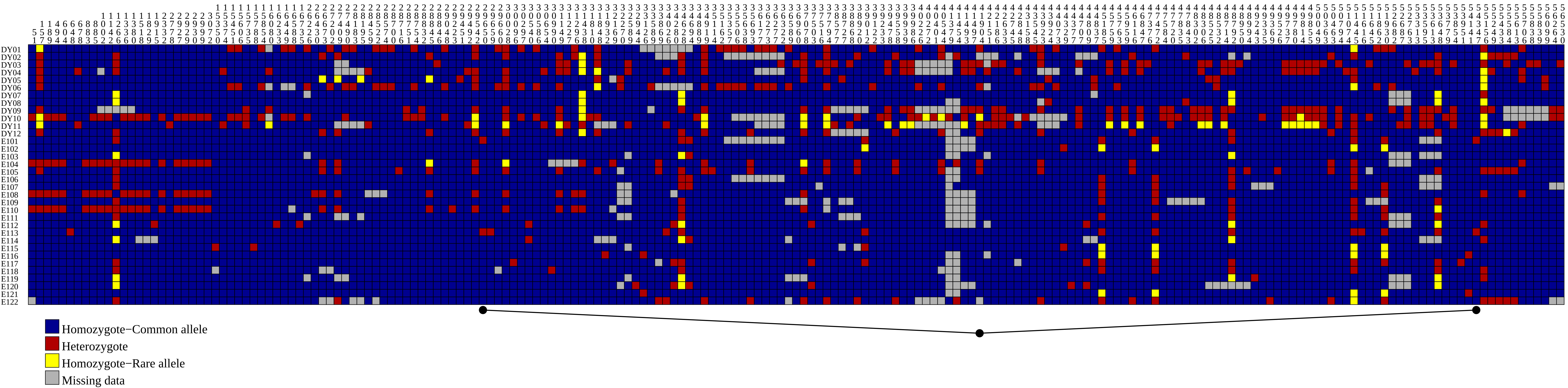

ccl5, p−value: 0.5222

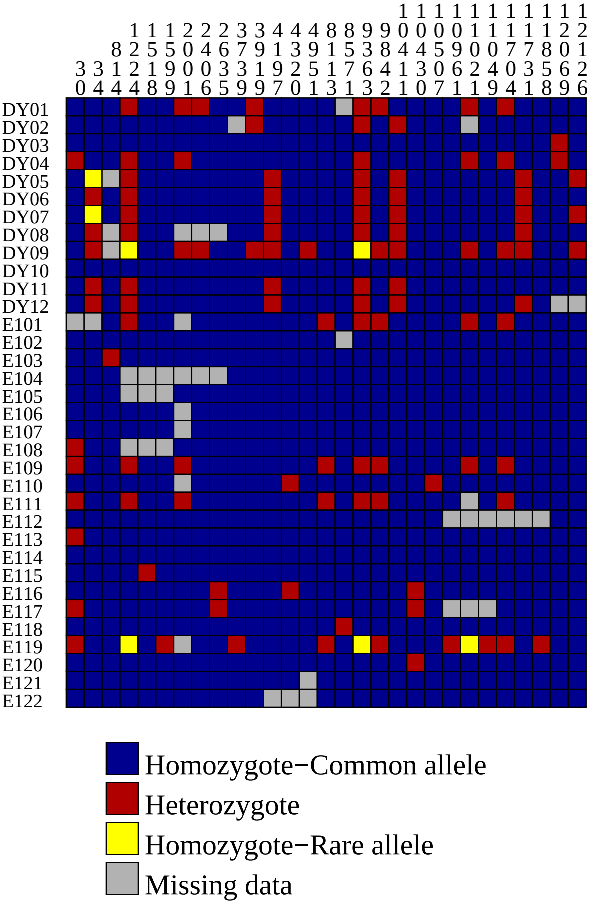

cd3z, p−value: 0.5518

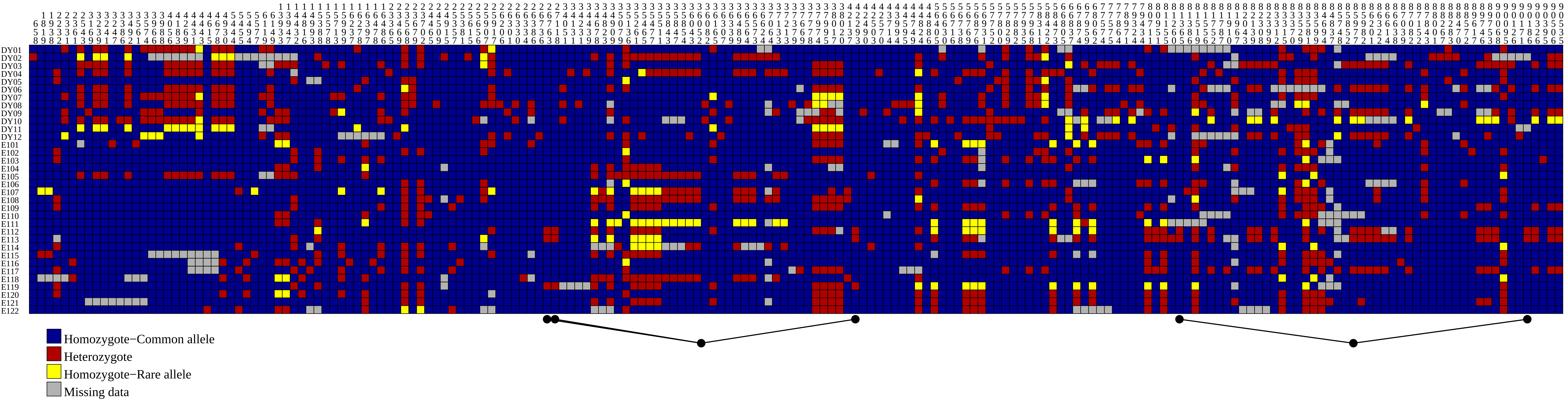

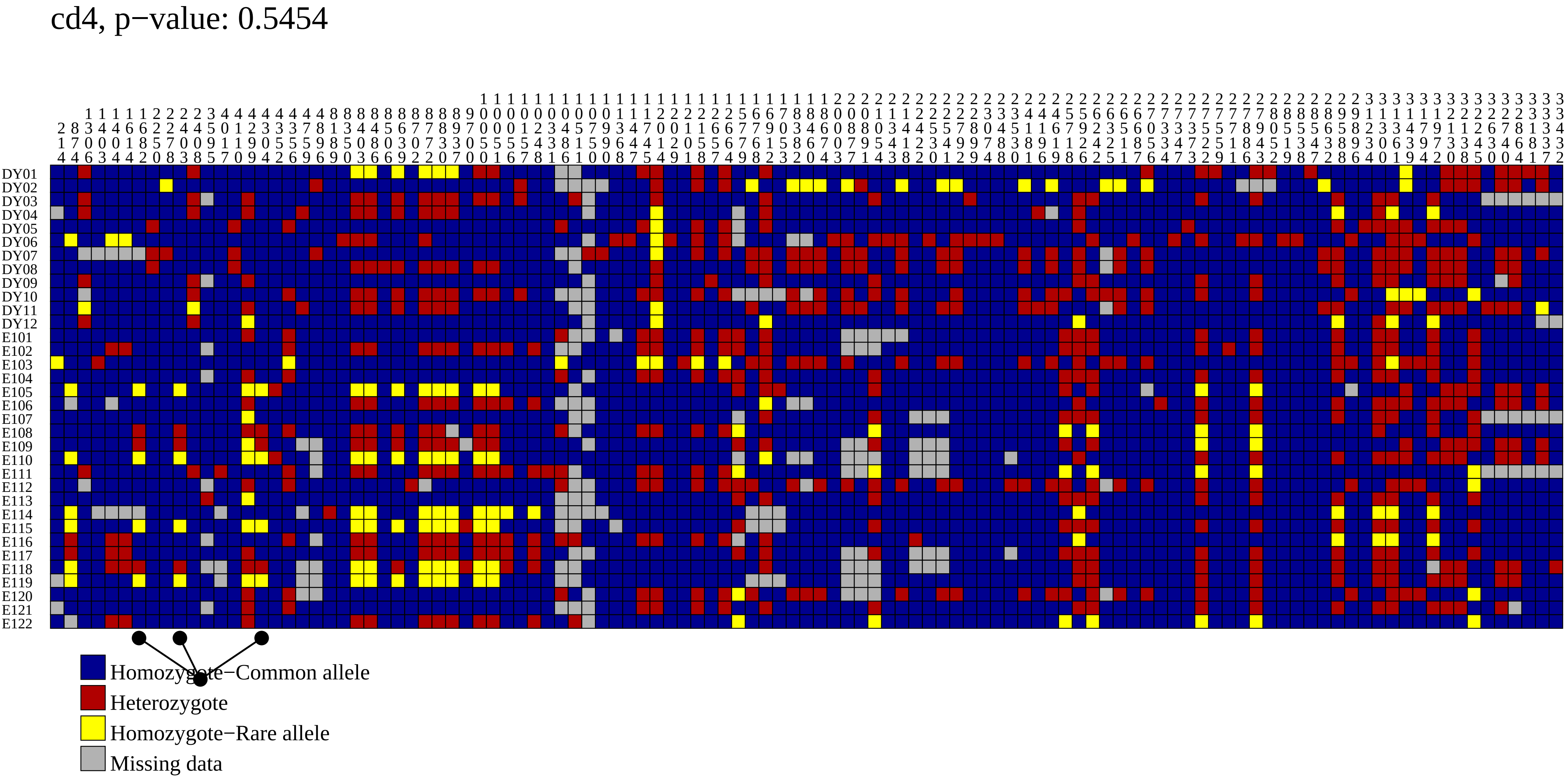
cd4, p−value: 0.5454
DY01
DY02
DY03
DY04
DY05
DY06
DY07
DY08
DY09
DY10
DY11
DY12
E101
E102
E103
E104
E105
E106
E107
E108
E109
E110
E111
E112
E113
E114
E115
E116
E117
E118
E119
E120
E121
E122
Homozygote−Common allele
Heterozygote
Homozygote−Rare allele
Missing data

cdc37, p−value: 0.5508

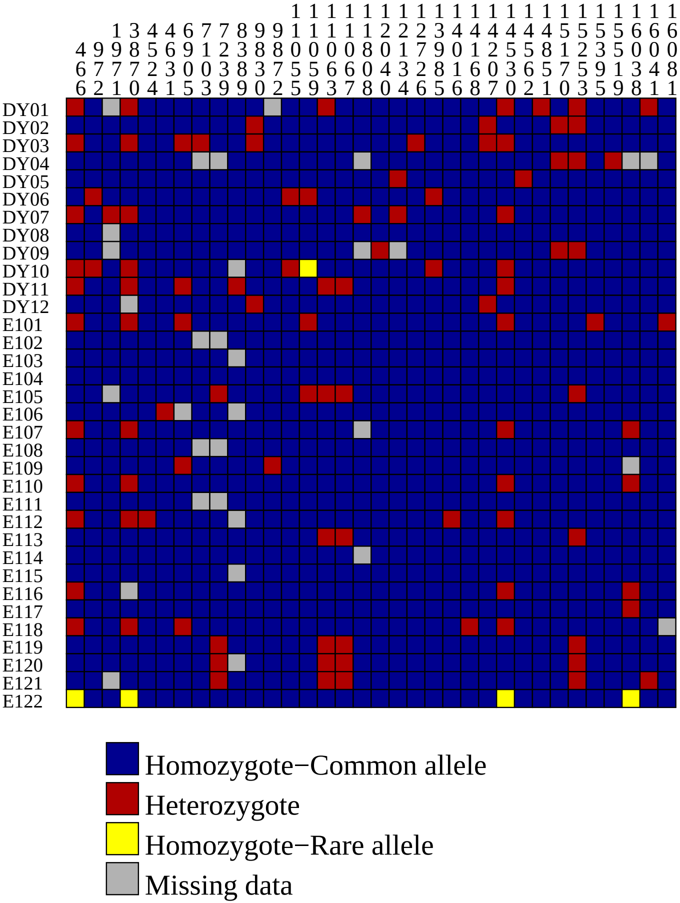

cdh1, p−value: 0.783

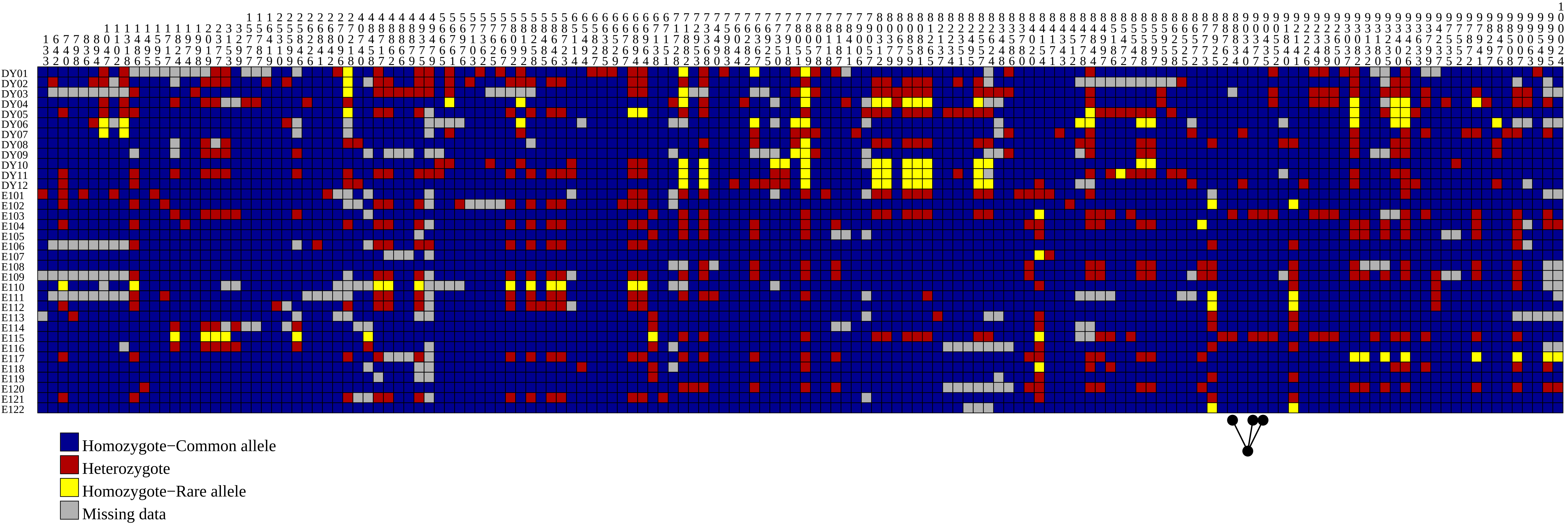

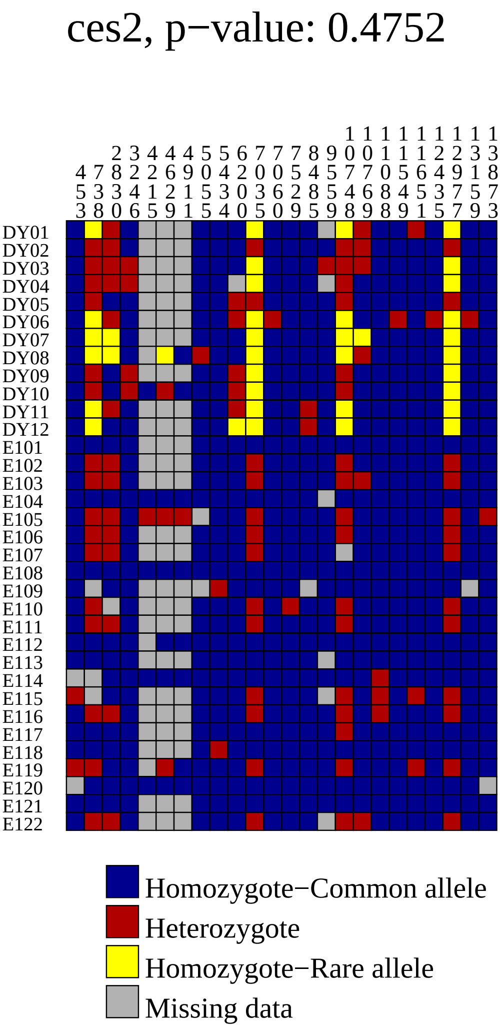
ces2, p−value: 0.4752
453
738
2830
3246
4215
4629
4911
5055
5434
6200
7035
7060
7529
8485
9559
10748
10769
11088
11549
11651
12435
12977
13159
13873
DY01
DY02
DY03
DY04
DY05
DY06
DY07
DY08
DY09
DY10
DY11
DY12
E101
E102
E103
E104
E105
E106
E107
E108
E109
E110
E111
E112
E113
E114
E115
E116
E117
E118
E119
E120
E121
E122
Homozygote−Common allele
Heterozygote
Homozygote−Rare allele
Missing data

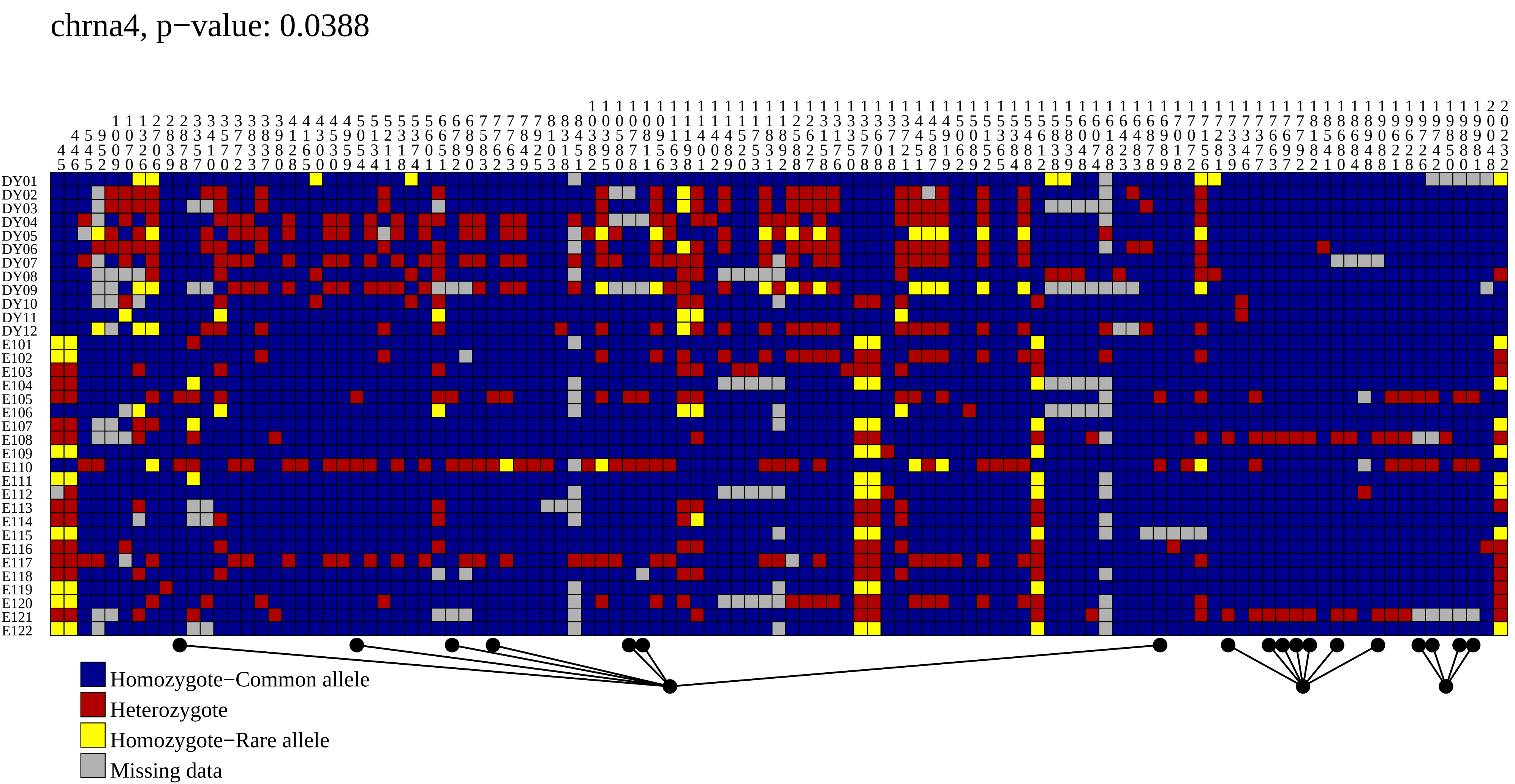

chrna4, p−value: 0.0388
DY01
DY02
DY03
DY04
DY05
DY06
DY07
DY08
DY09
DY10
DY11
DY12
E101
E102
E103
E104
E105
E106
E107
E108
E109
E110
E111
E112
E113
E114
E115
E116
E117
E118
E119
E120
E121
E122
Homozygote−Common allele
Heterozygote
Homozygote−Rare allele
Missing data

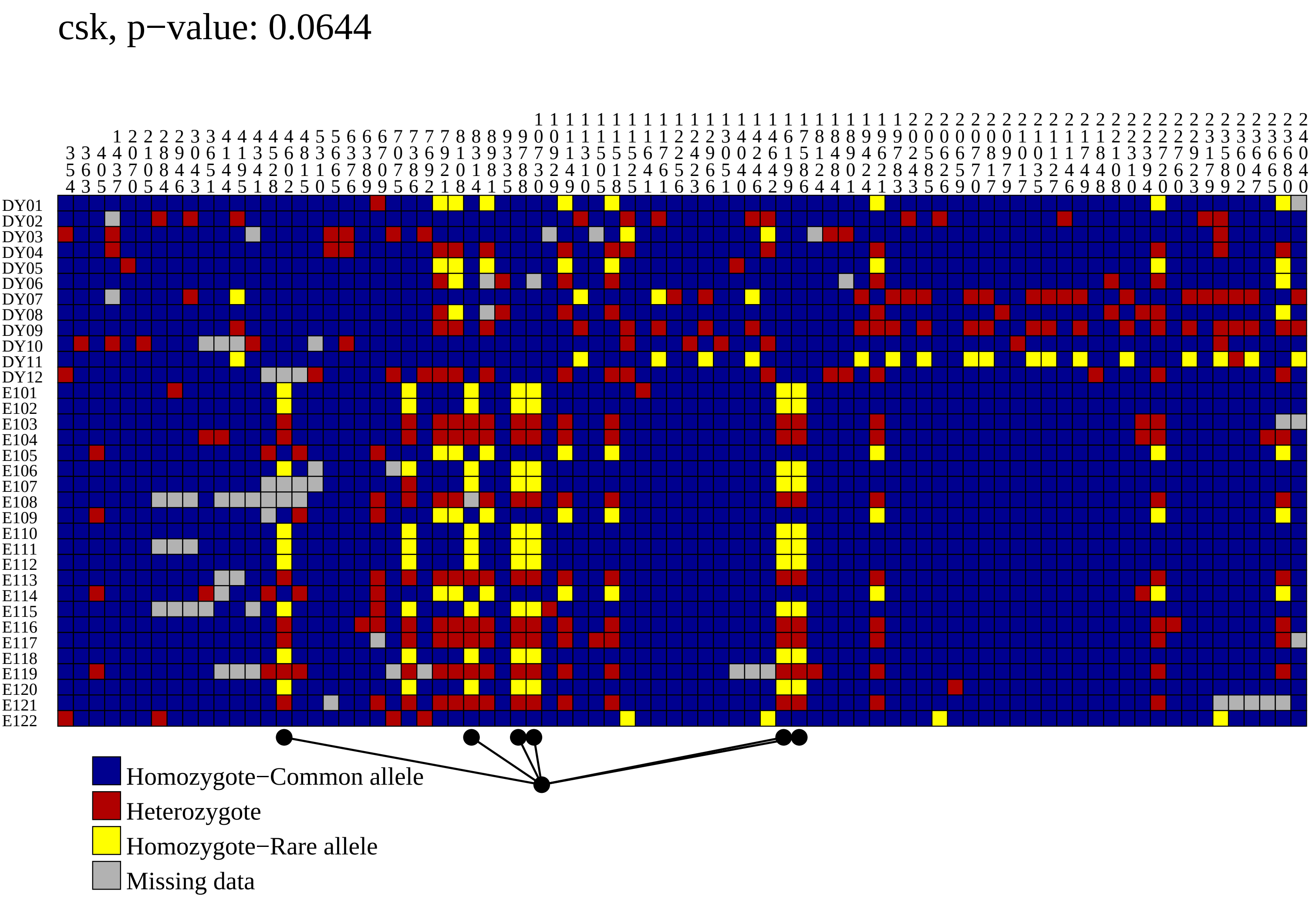
csk, p−value: 0.0644
Homozygote−Common allele
Heterozygote
Homozygote−Rare allele
Missing data

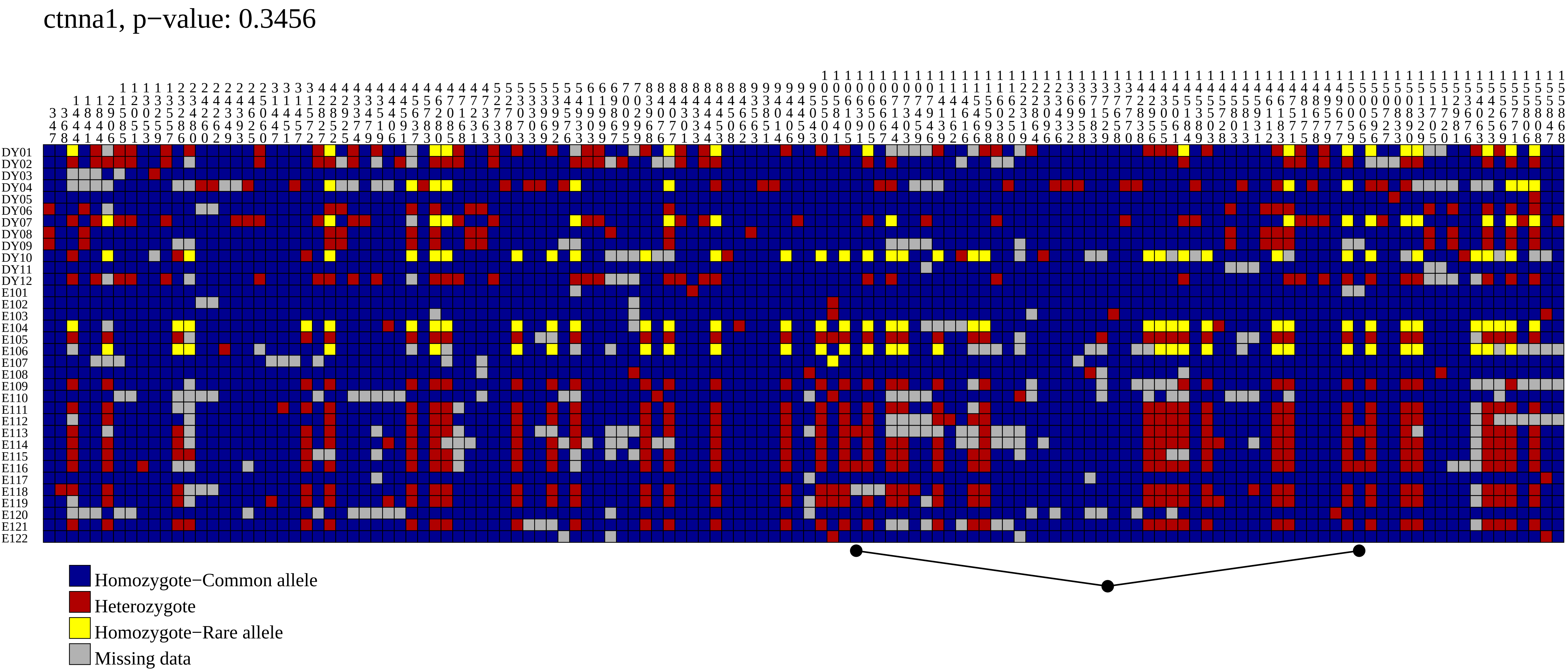
ctnna1, p−value: 0.3456
Homozygote−Common allele
Heterozygote
Homozygote−Rare allele
Missing data

cyp19a1, p−value: 0.7878

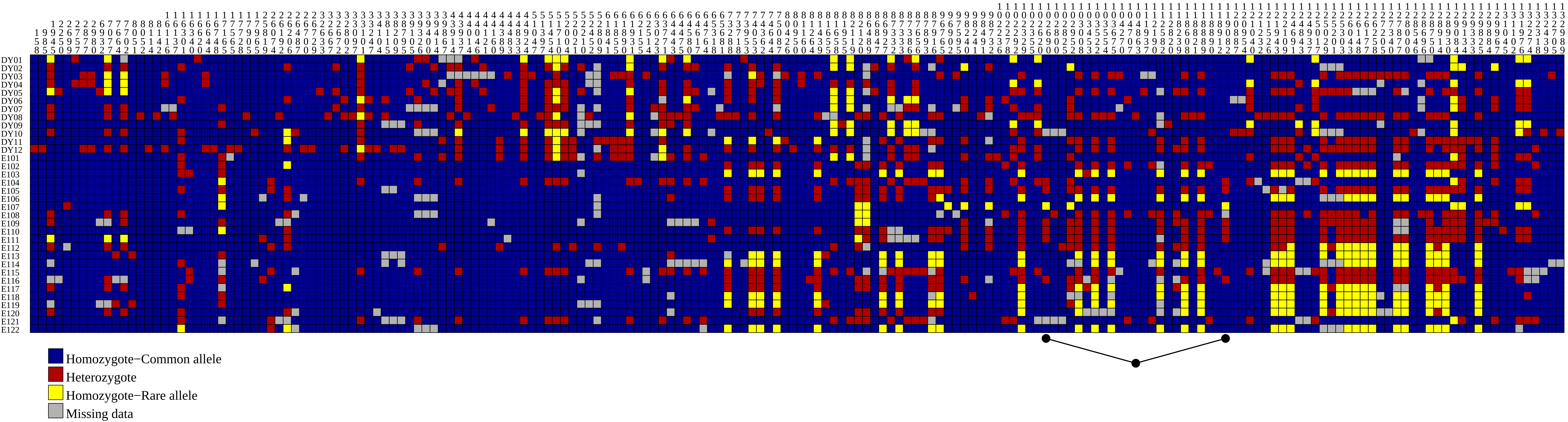

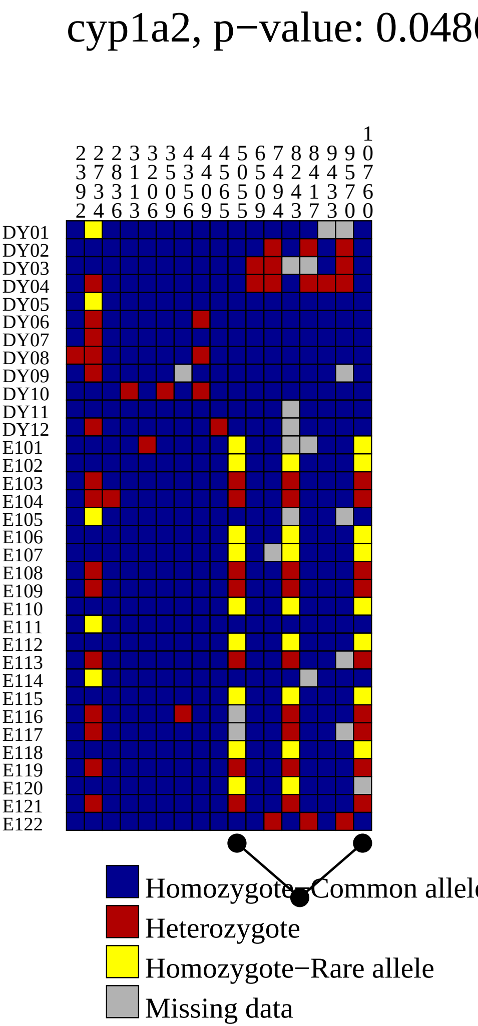
cyp1a2, p−value: 0.048
2392 2734 2836 3113 3206 3509 4356 4409 4565 5055 6509 7494 8243 8417 9433 9570 10760
DY01 DY02 DY03 DY04 DY05 DY06 DY07 DY08 DY09 DY10 DY11 DY12
E101 E102 E103 E104 E105 E106 E107 E108 E109 E110 E111 E112 E113 E114 E115 E116 E117 E118 E119 E120 E121 E122
Homozygote−Common allele
Heterozygote
Homozygote−Rare allele
Missing data

cyp2b6, p−value: 0.797

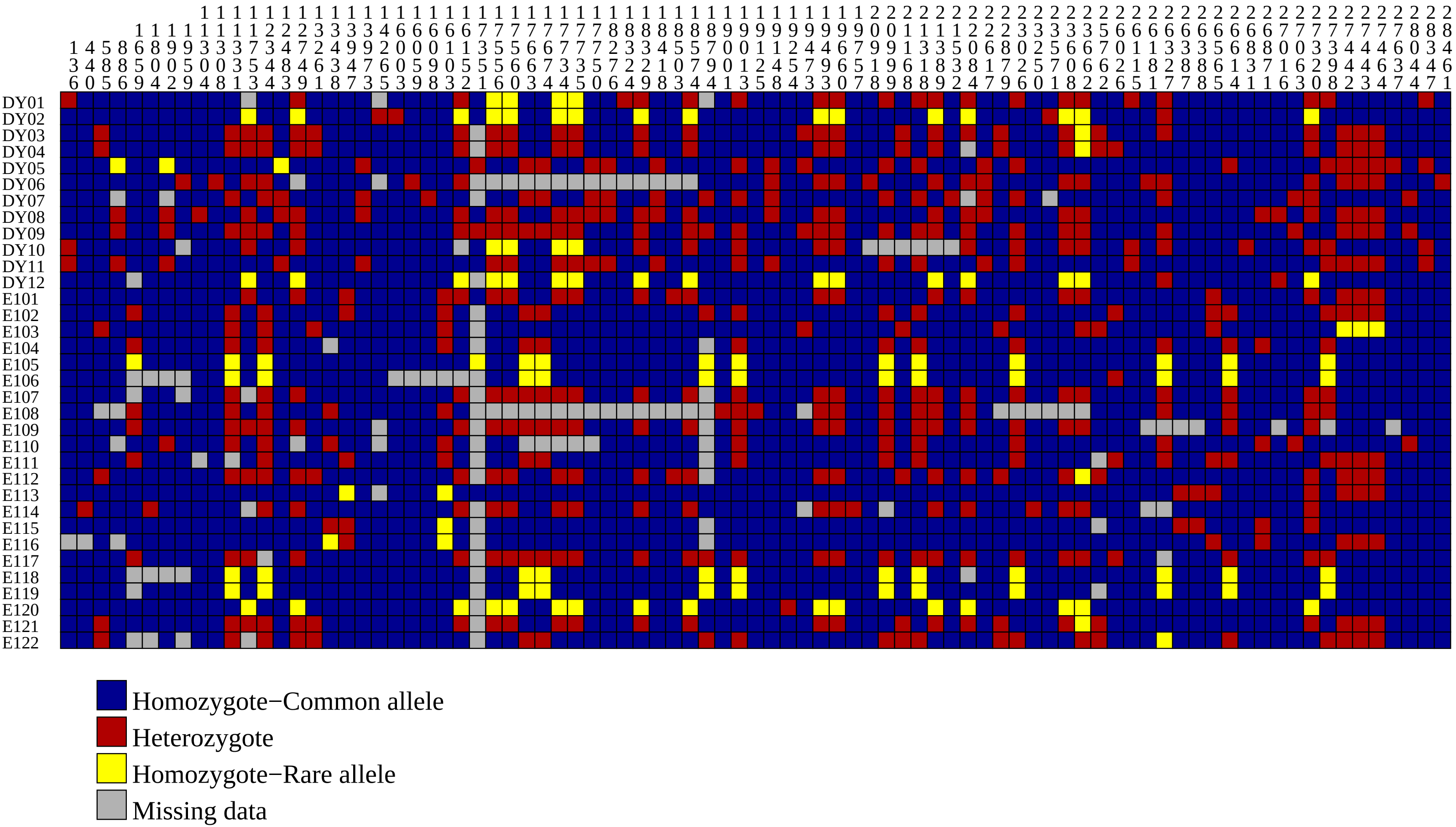

cyp3a4, p−value: 0.5744

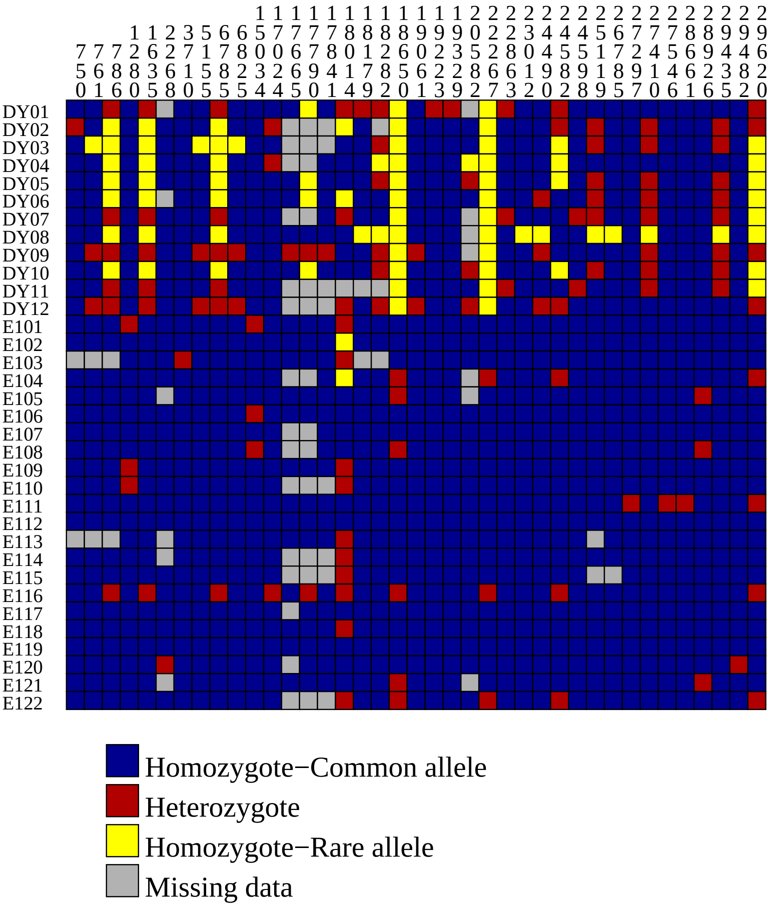

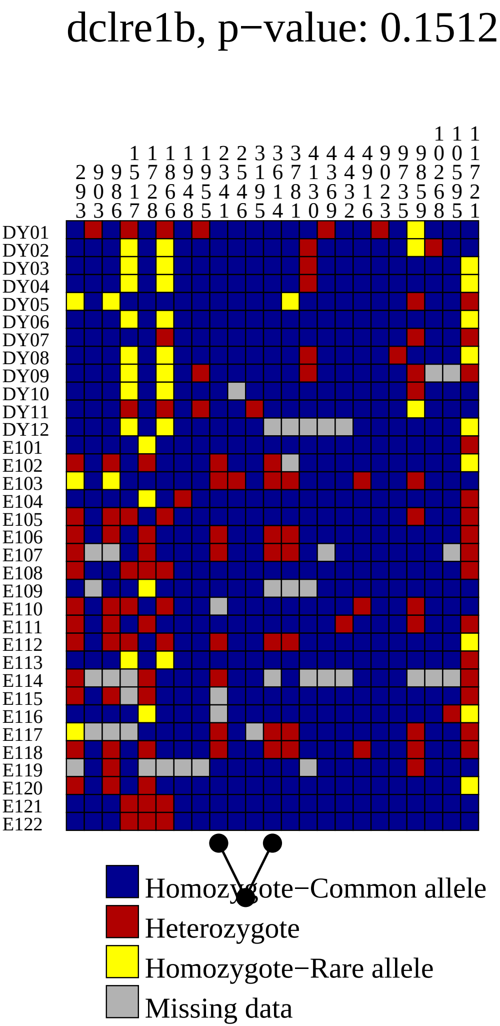
dclre1b, p−value: 0.1512
293
903
986
1517
1728
1866
1948
1955
2341
2546
3195
3614
3781
4130
4369
4432
4916
9023
9735
9859
10268
10595
11721
DY01
DY02
DY03
DY04
DY05
DY06
DY07
DY08
DY09
DY10
DY11
DY12
E101
E102
E103
E104
E105
E106
E107
E108
E109
E110
E111
E112
E113
E114
E115
E116
E117
E118
E119
E120
E121
E122
Homozygote−Common allele
Heterozygote
Homozygote−Rare allele
Missing data

ddb1, p−value: 0.7964

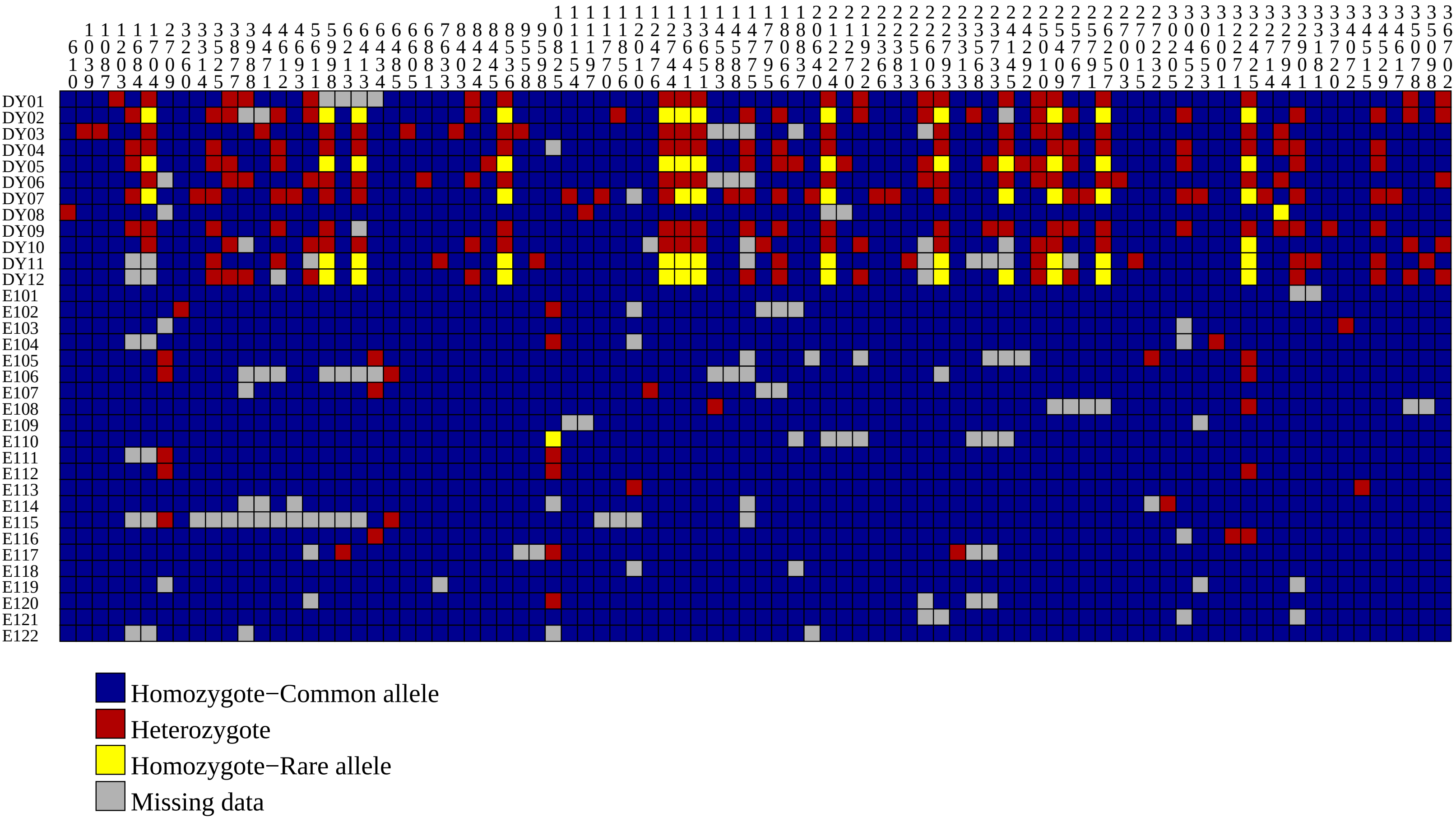

ddit3, p−value: 0.4

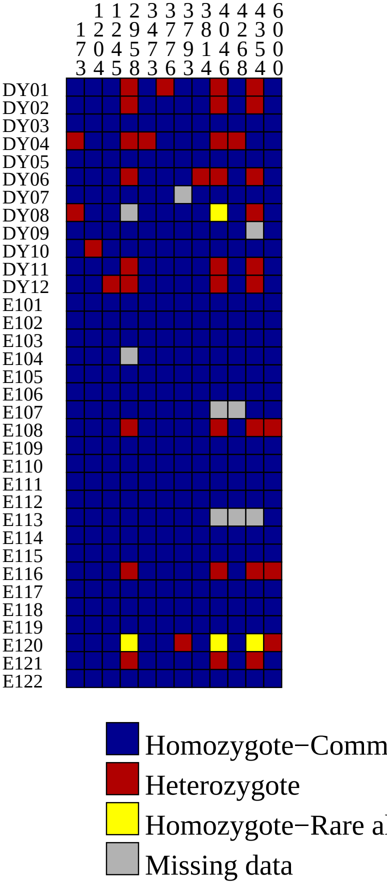

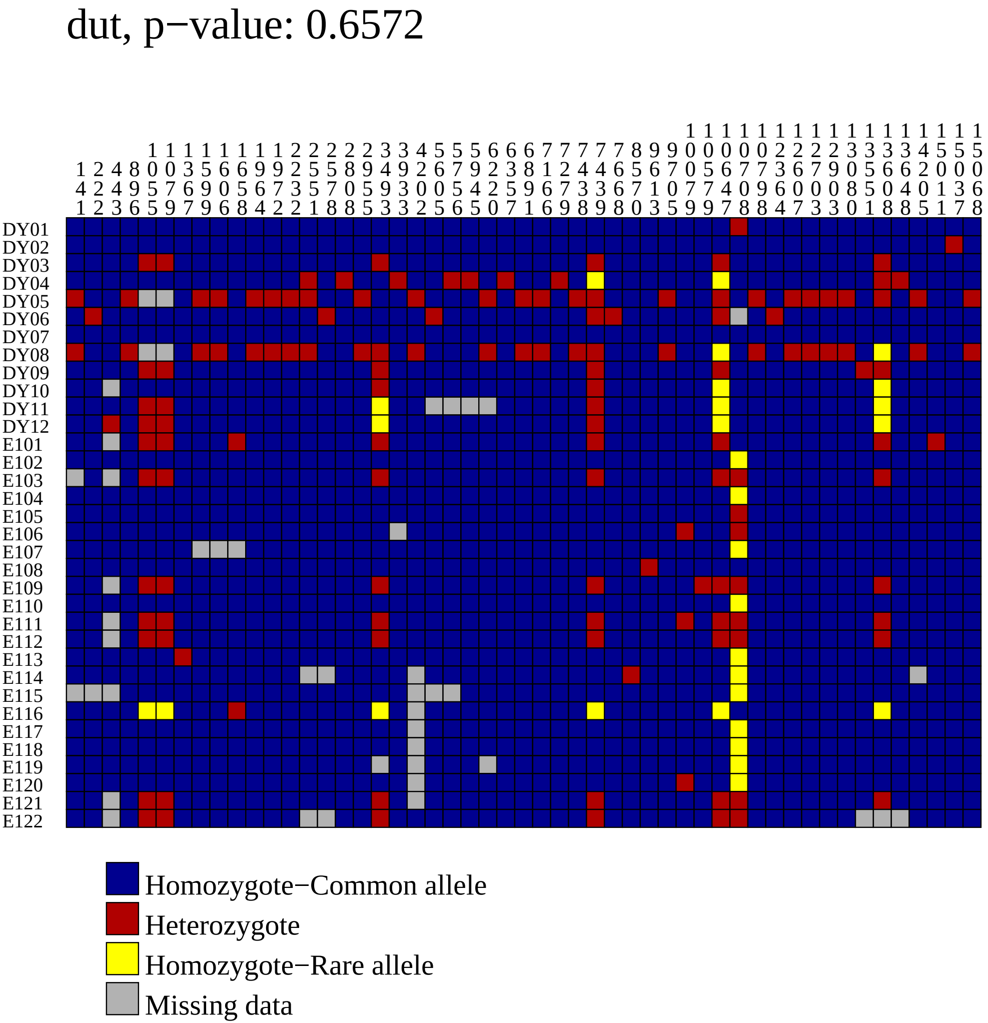
dut, p−value: 0.6572
141 222 443 896 1055 1079 1367 1599 1606 1658 1964 1972 2232 2551 2578 2808 2955 3493 3933 4202 5605 5756 5945 6220 6357 6891 7166 7279 7438 7439 7868 8570 9613 9705 10079 10579 10647 10708 10798 12364 12607 12703 12903 13080 13551 13608 13648 14205 15011 15037 15068
DY01
DY02
DY03
DY04
DY05
DY06
DY07
DY08
DY09
DY10
DY11
DY12
E101
E102
E103
E104
E105
E106
E107
E108
E109
E110
E111
E112
E113
E114
E115
E116
E117
E118
E119
E120
E121
E122
Homozygote−Common allele
Heterozygote
Homozygote−Rare allele
Missing data

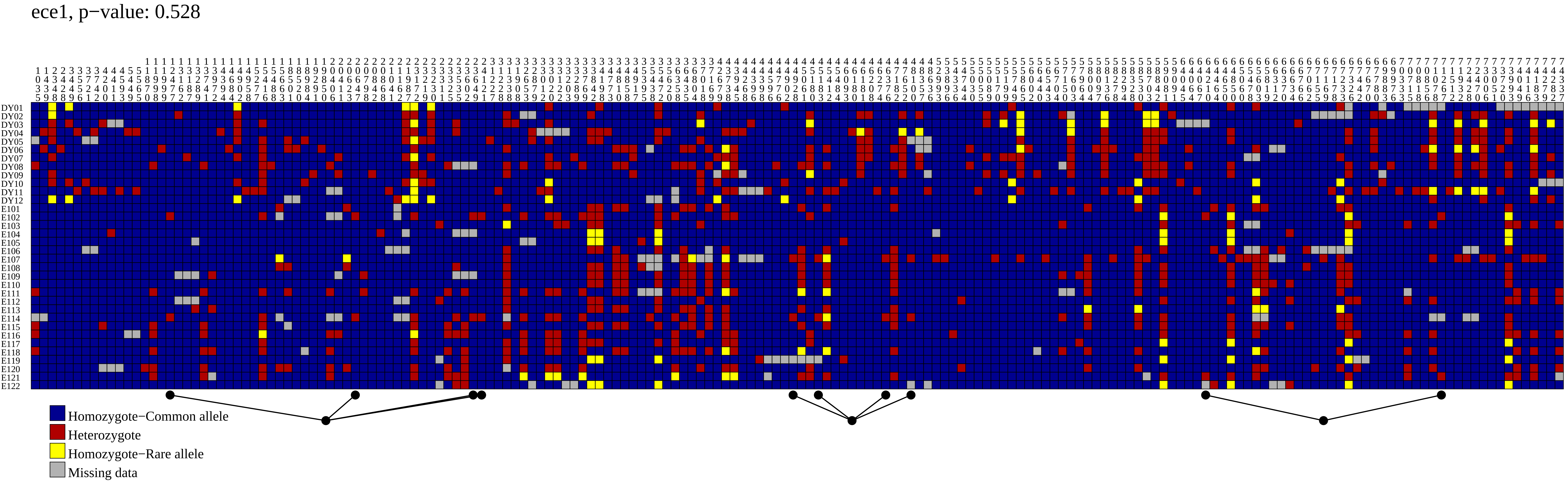
ece1, p−value: 0.528
Homozygote−Common allele
Heterozygote
Homozygote−Rare allele
Missing data

eno1, p−value: 0.7168

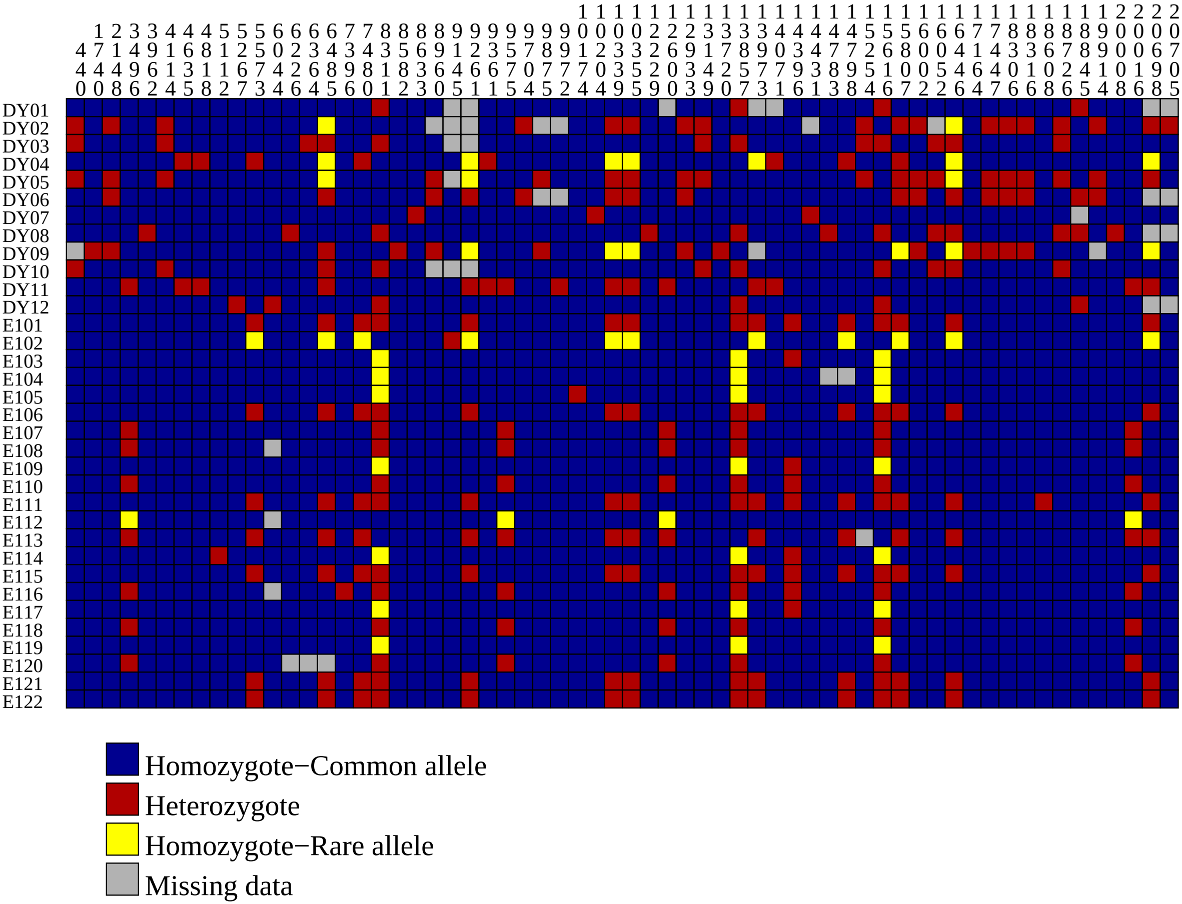

epx, p−value: 0.6962

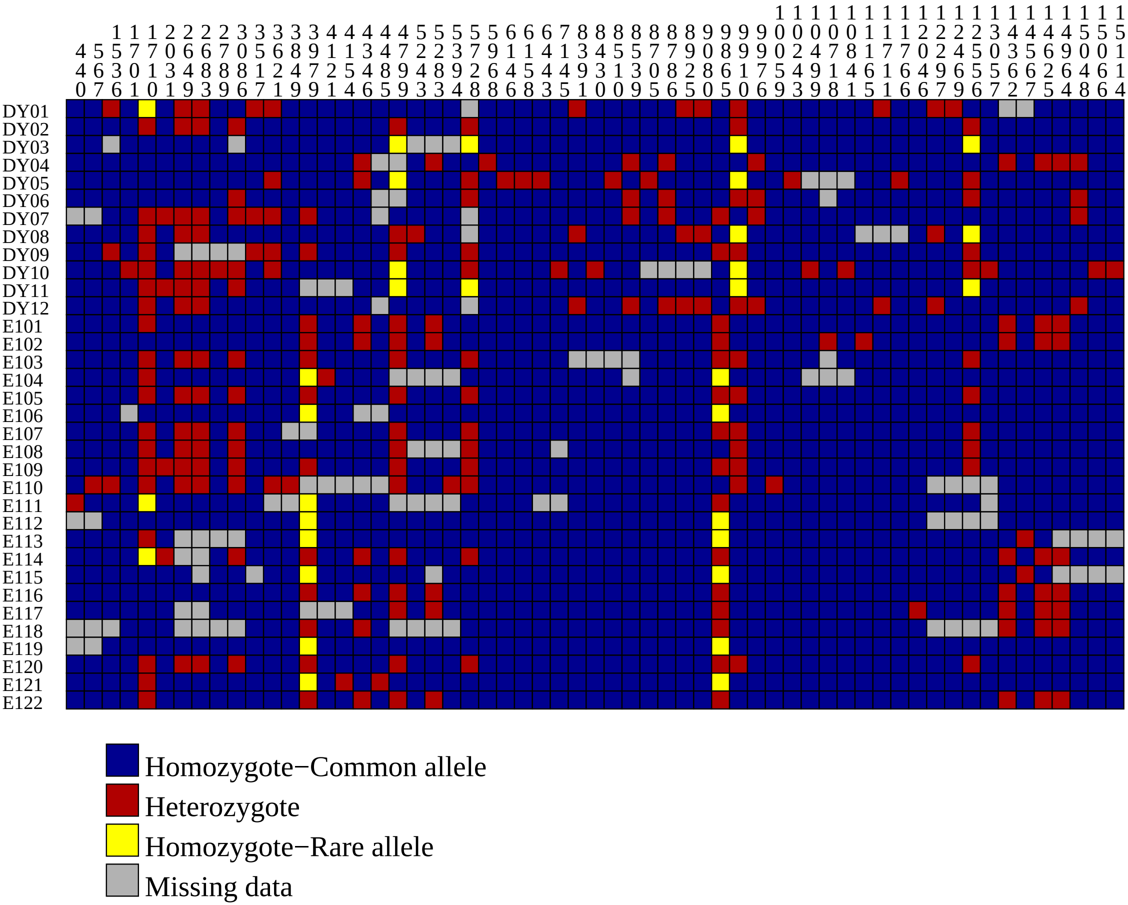

fance, p−value: 0.7212

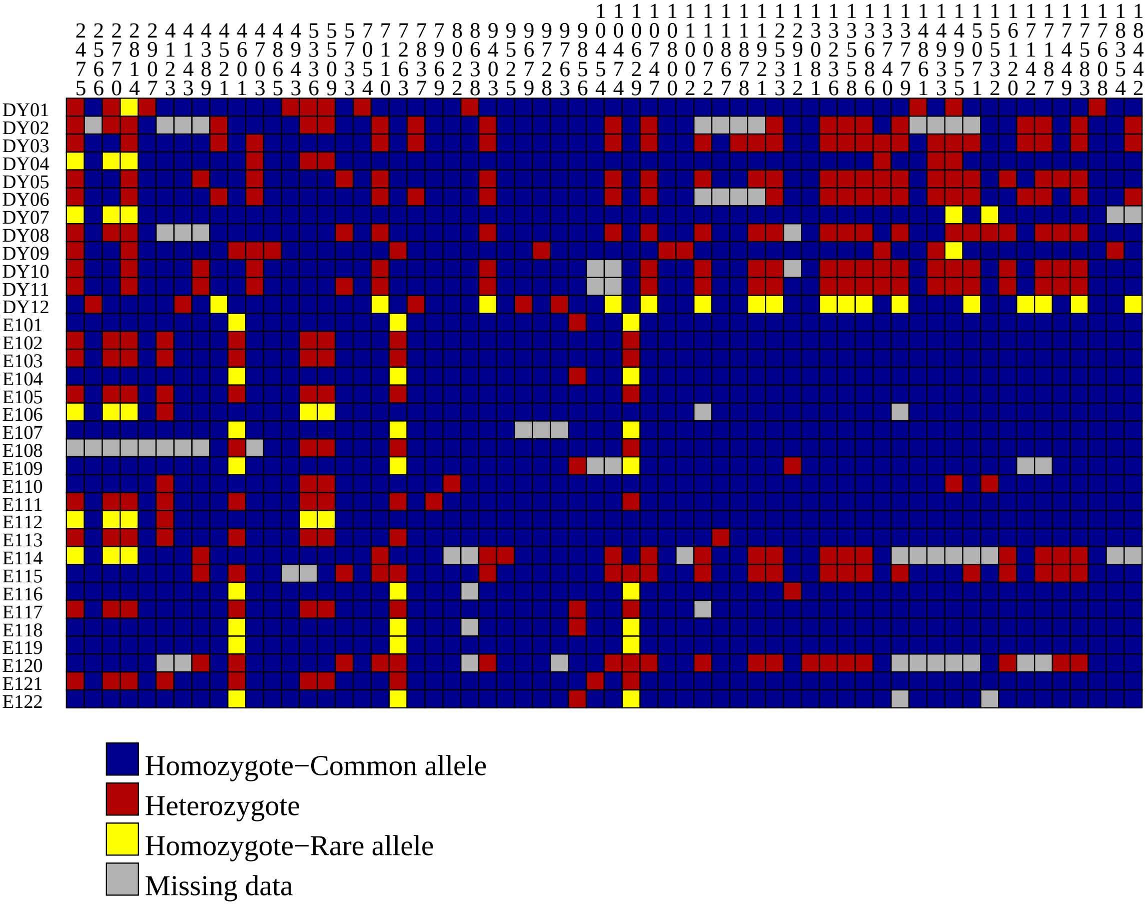

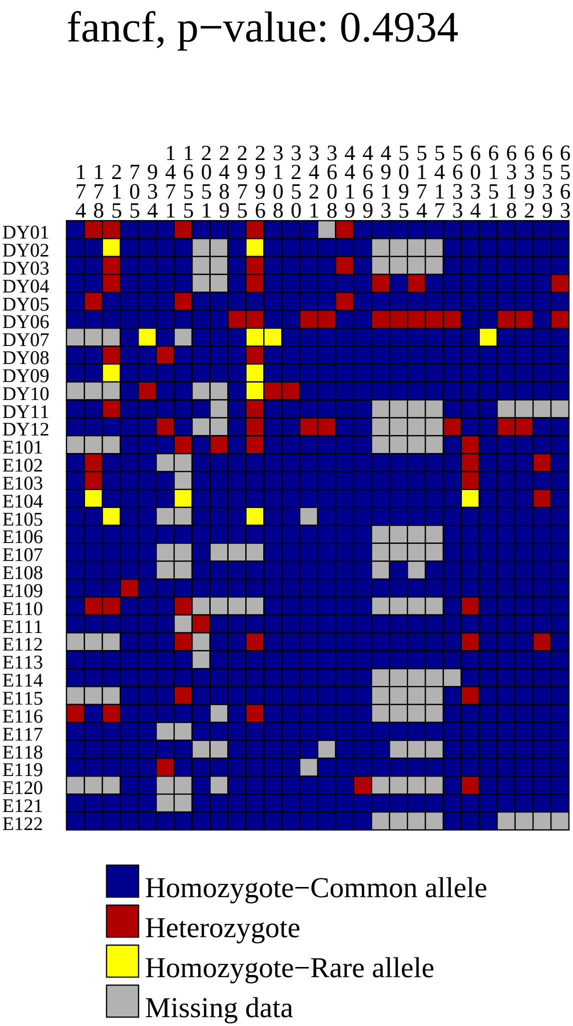
fancf, p−value: 0.4934
174
178
215
705
934
1471
1655
2051
2489
2975
2996
3108
3250
3421
3608
4419
4669
4913
5095
5174
5417
5633
6034
6151
6318
6392
6539
6563
DY01
DY02
DY03
DY04
DY05
DY06
DY07
DY08
DY09
DY10
DY11
DY12
E101
E102
E103
E104
E105
E106
E107
E108
E109
E110
E111
E112
E113
E114
E115
E116
E117
E118
E119
E120
E121
E122
Homozygote−Common allele
Heterozygote
Homozygote−Rare allele
Missing data

fbp1, p−value: 0.1858

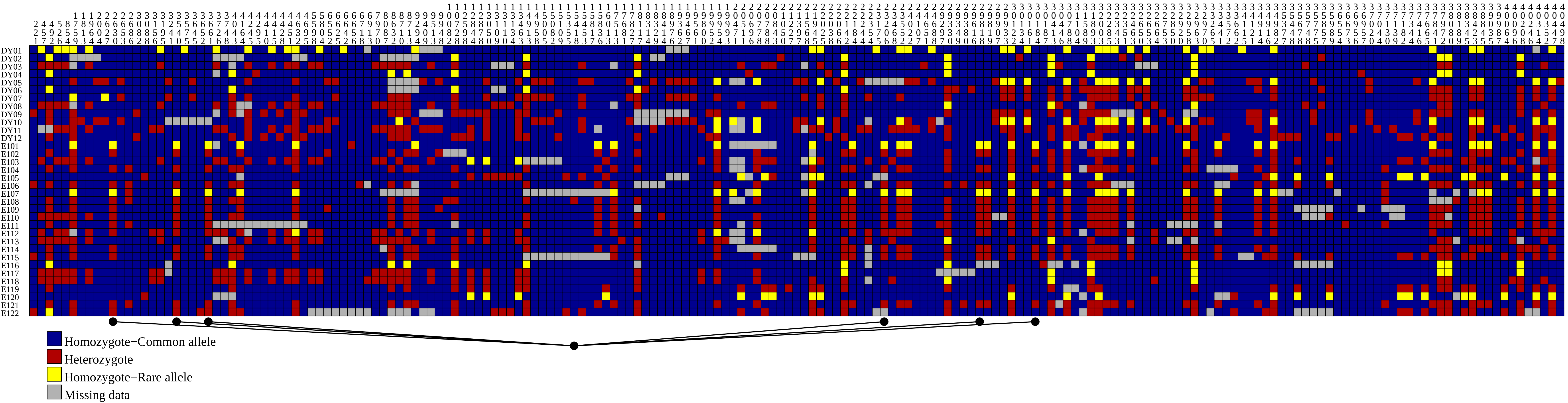

fdxr, p−value: 0.0266

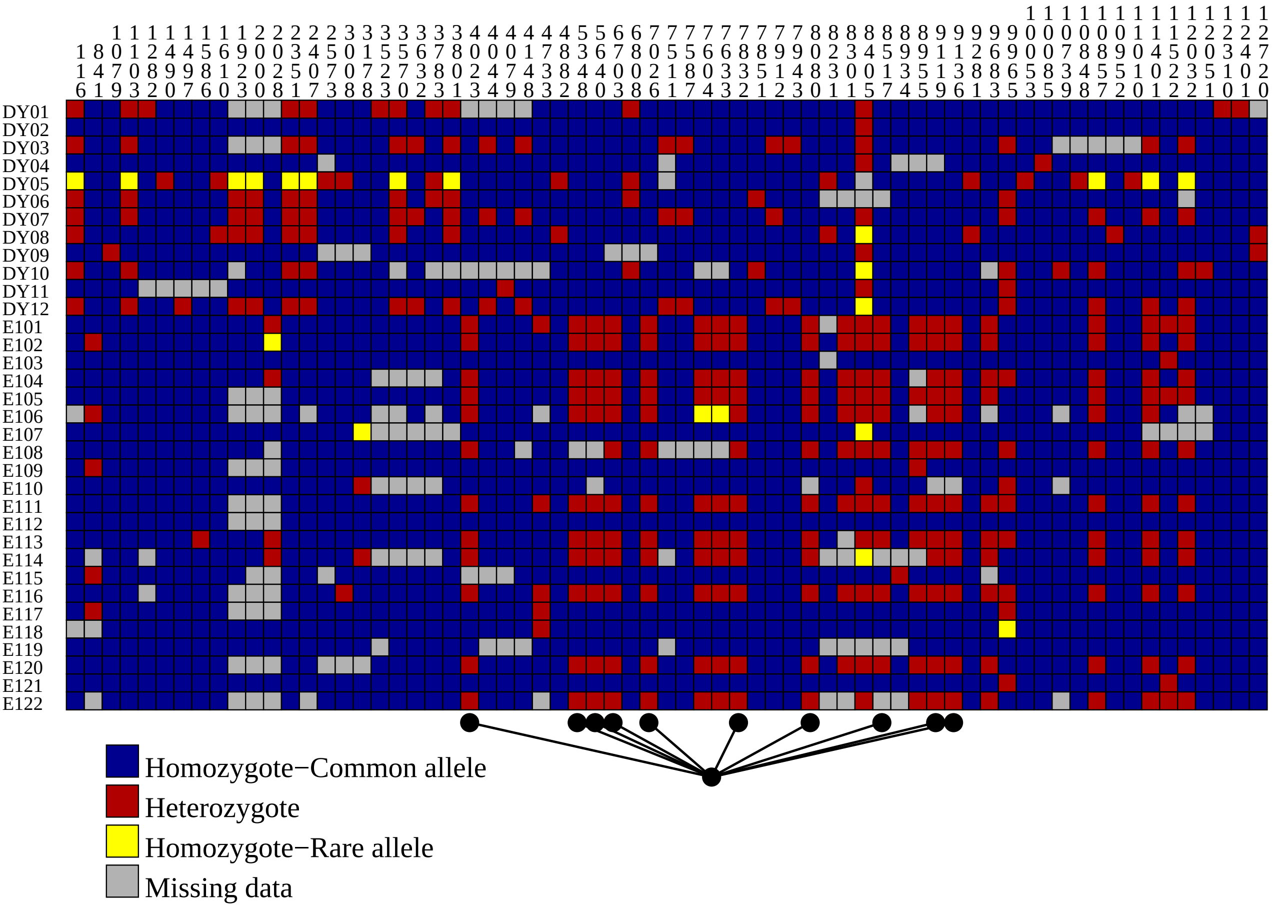

fgf5, p−value: 0.0332

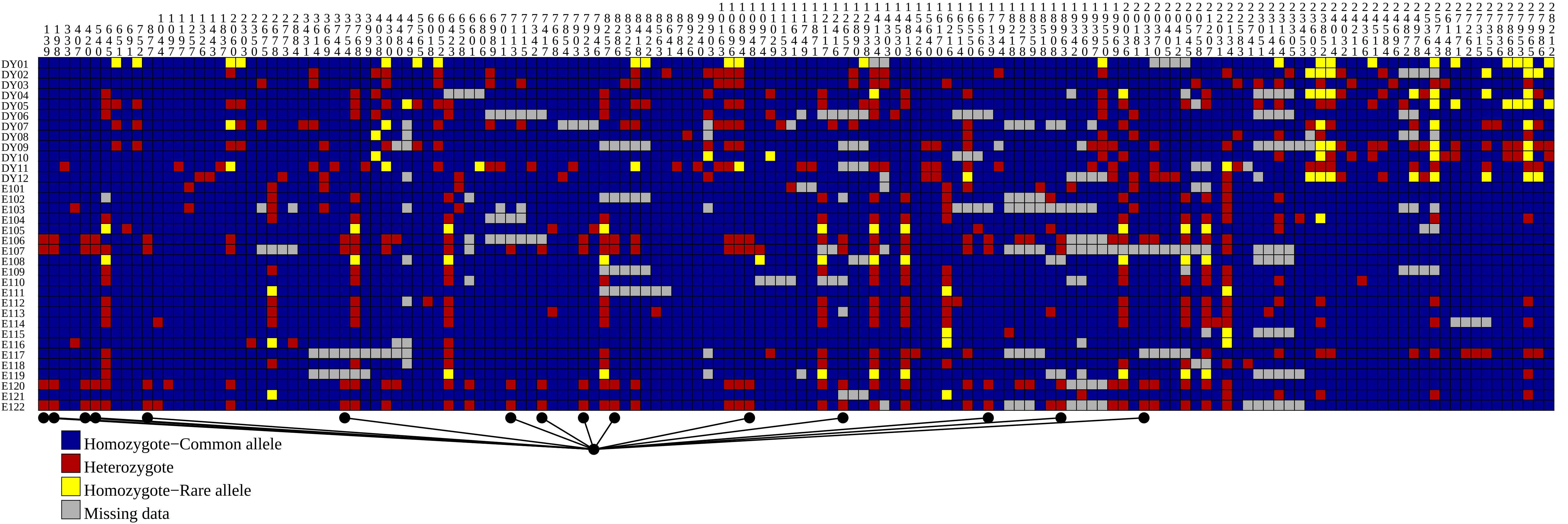

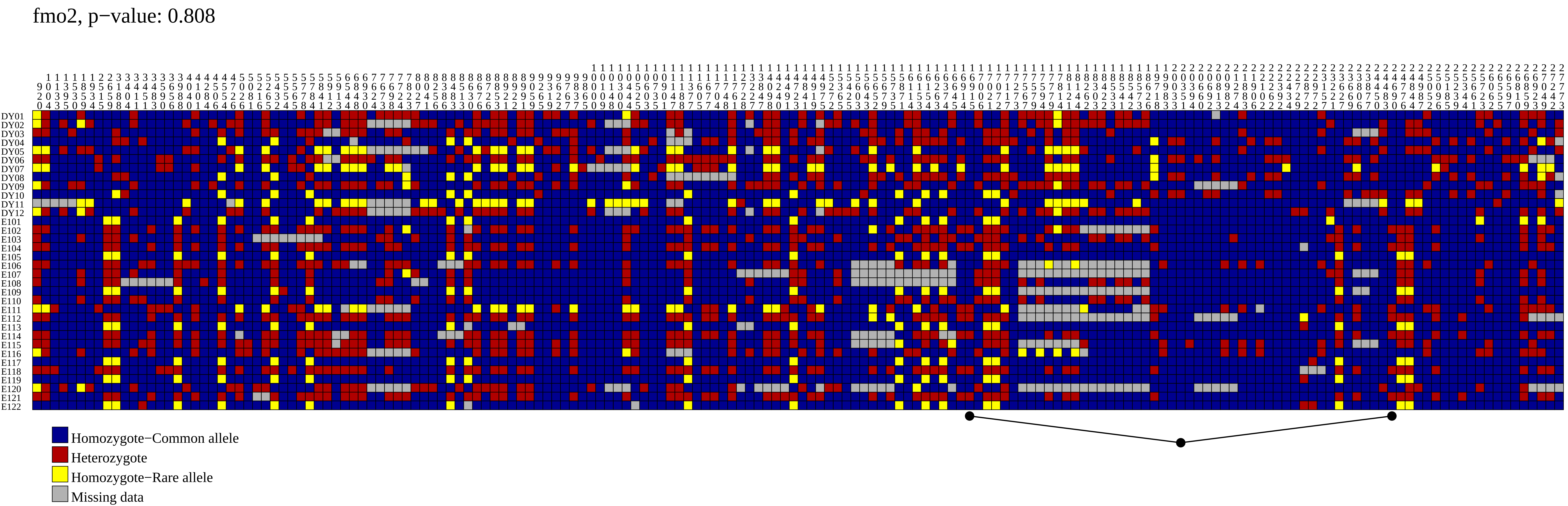
fmo2, p−value: 0.808
DY01
DY02
DY03
DY04
DY05
DY06
DY07
DY08
DY09
DY10
DY11
DY12
E101
E102
E103
E104
E105
E106
E107
E108
E109
E110
E111
E112
E113
E114
E115
E116
E117
E118
E119
E120
E121
E122
Homozygote−Common allele
Heterozygote
Homozygote−Rare allele
Missing data

fmo3, p−value: 0.0946

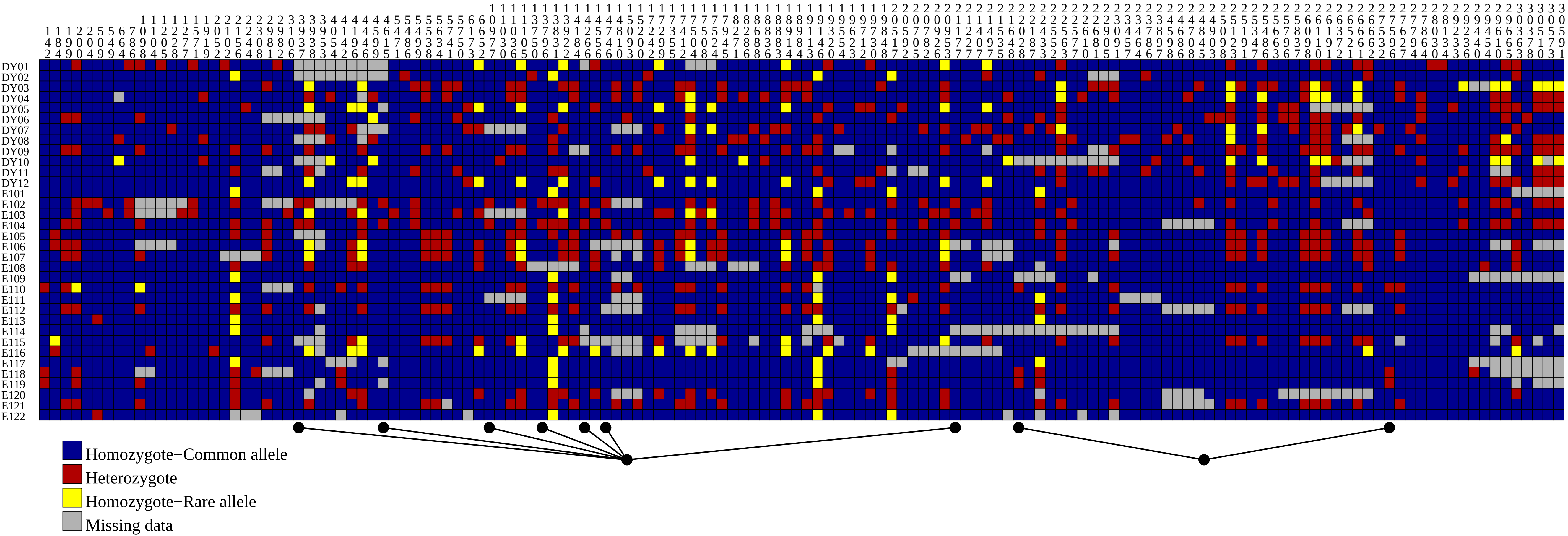

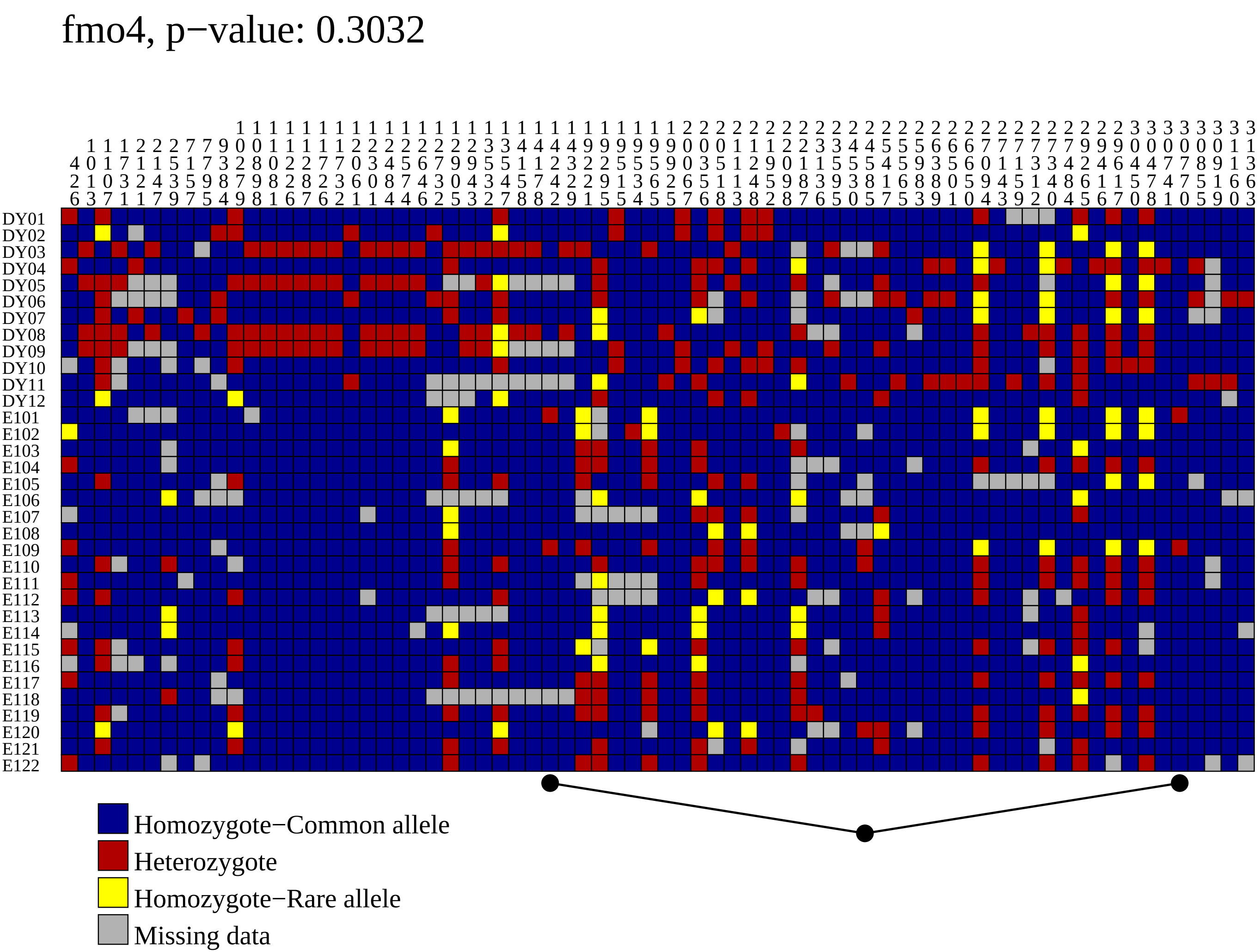
fmo4, p−value: 0.3032
DY01
DY02
DY03
DY04
DY05
DY06
DY07
DY08
DY09
DY10
DY11
DY12
E101
E102
E103
E104
E105
E106
E107
E108
E109
E110
E111
E112
E113
E114
E115
E116
E117
E118
E119
E120
E121
E122
Homozygote−Common allele
Heterozygote
Homozygote−Rare allele
Missing data

fmo5, p−value: 0.0404

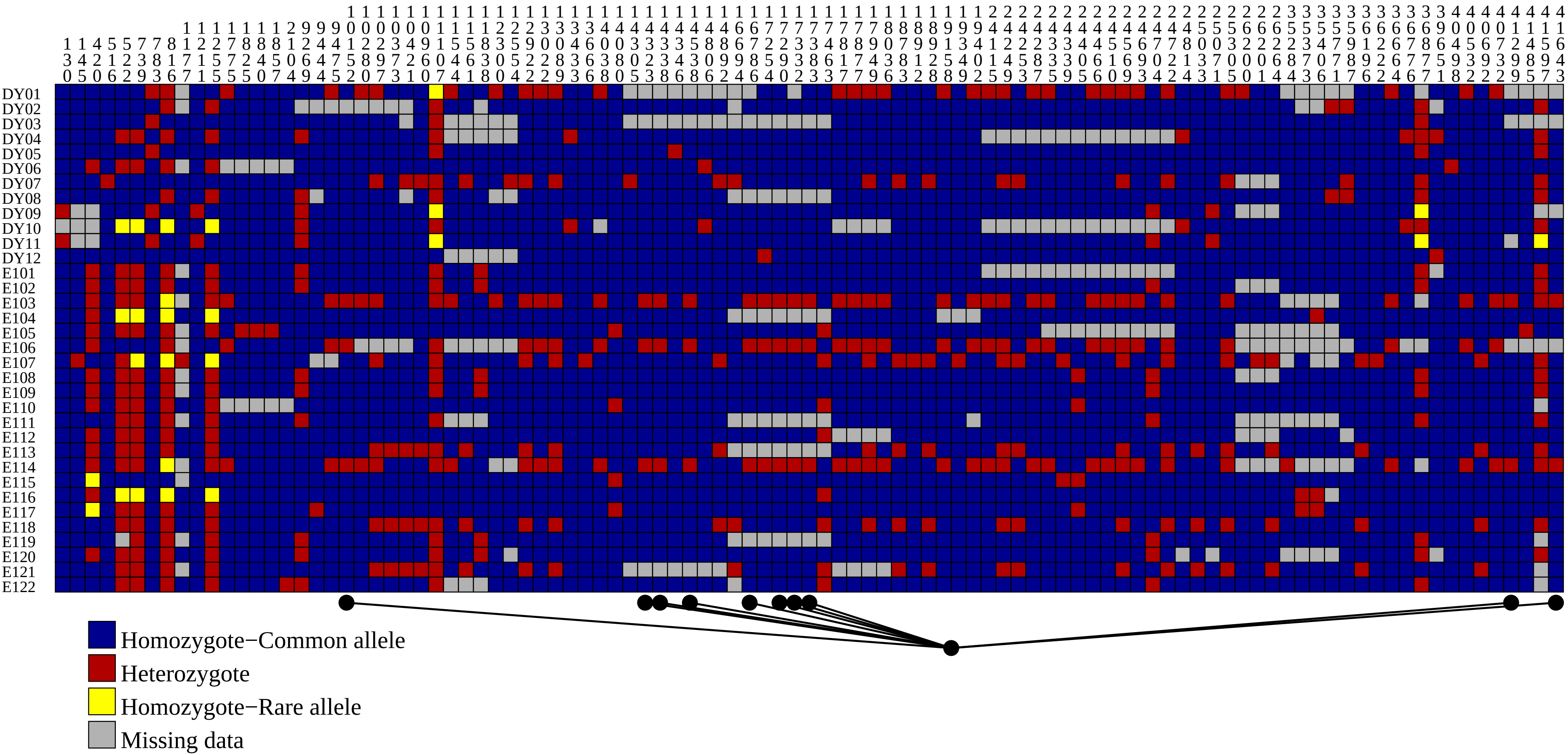

fosb, p−value: 0.5878

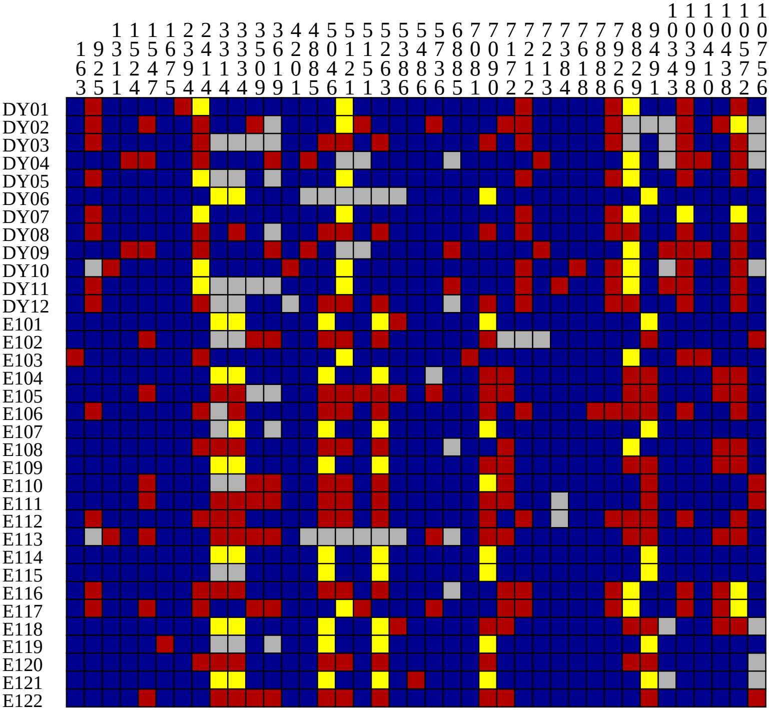

Homozygote−Common allele

Heterozygote

Homozygote−Rare allele

Missing data

foxm1, p−value: 0.1108

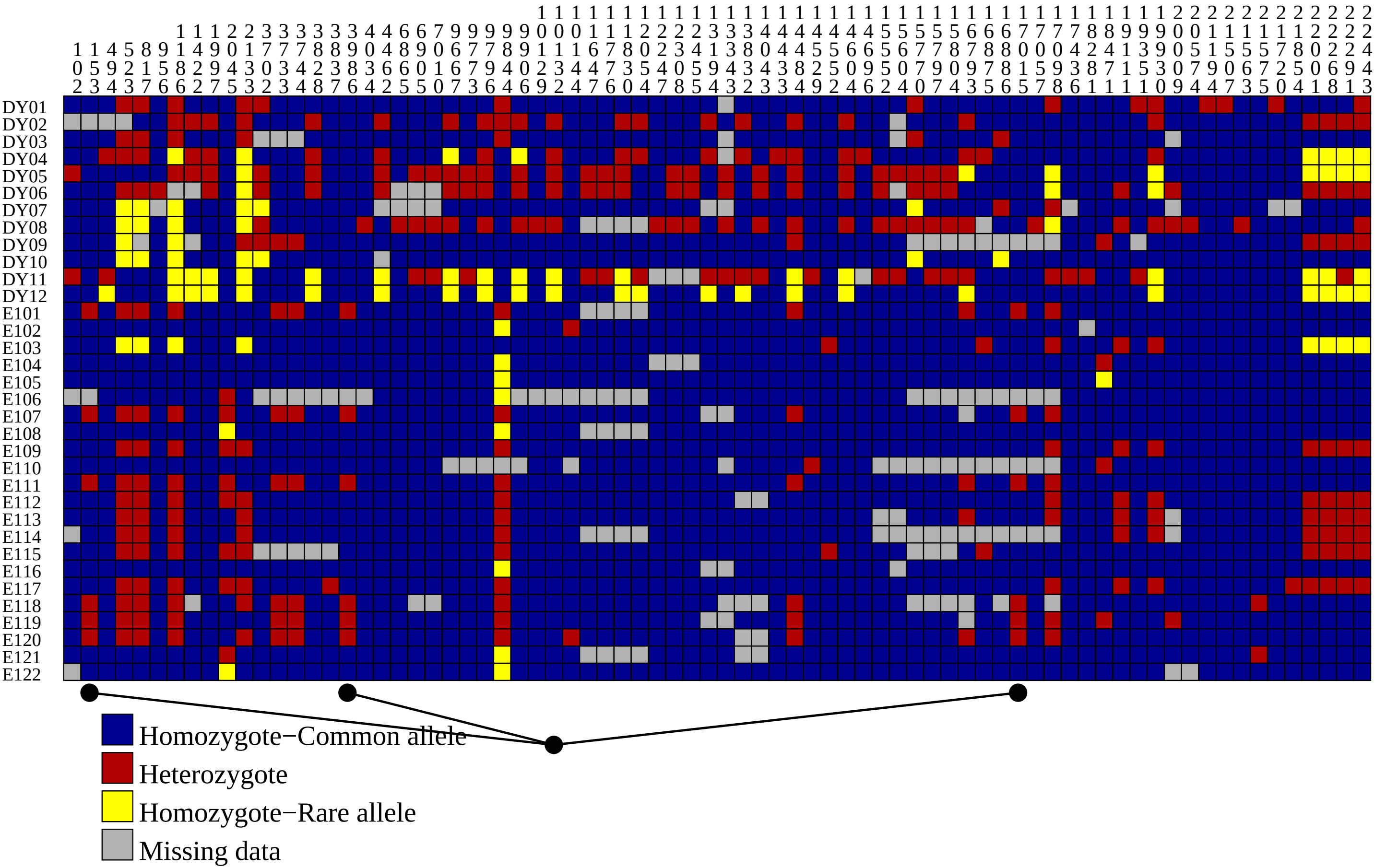

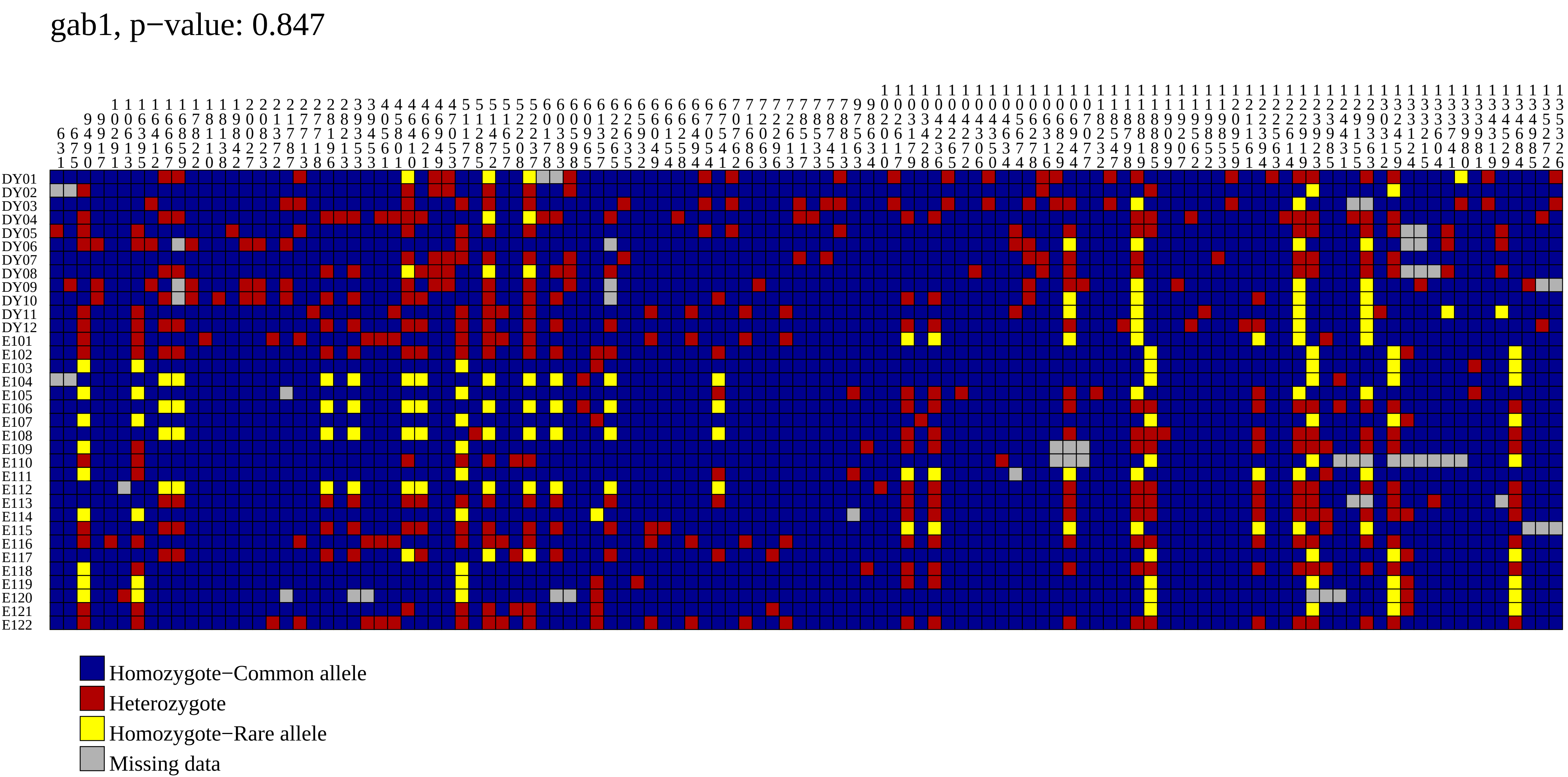
gab1, p−value: 0.847
DY01
DY02
DY03
DY04
DY05
DY06
DY07
DY08
DY09
DY10
DY11
DY12
E101
E102
E103
E104
E105
E106
E107
E108
E109
E110
E111
E112
E113
E114
E115
E116
E117
E118
E119
E120
E121
E122
Homozygote−Common allele
Heterozygote
Homozygote−Rare allele
Missing data

glrx2, p−value: 0.5046

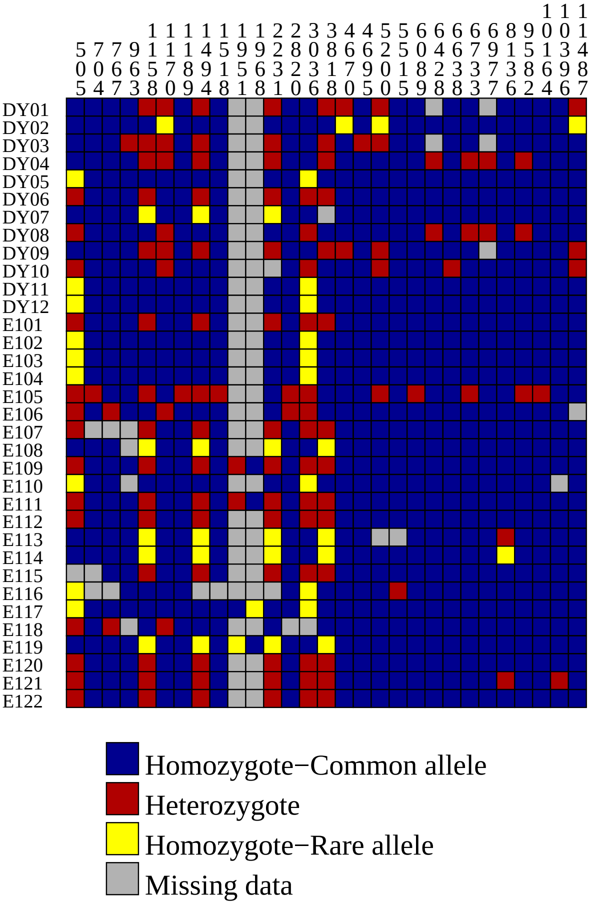

glrx, p−value: 0.6068

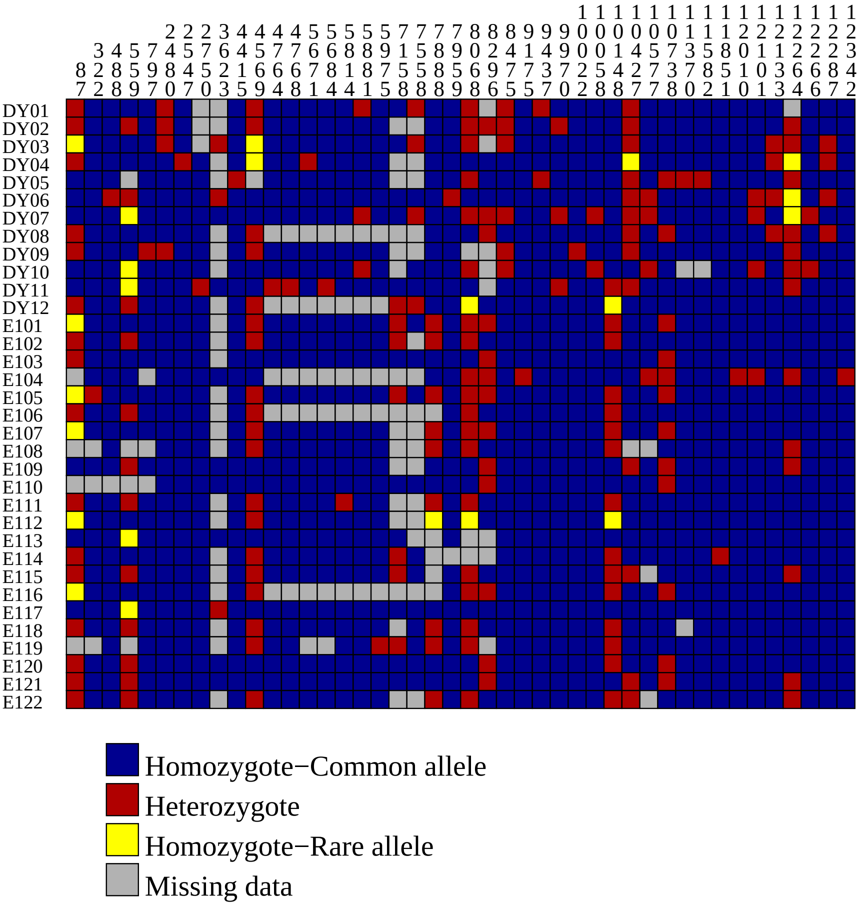

gpx5, p−value: 0.2898

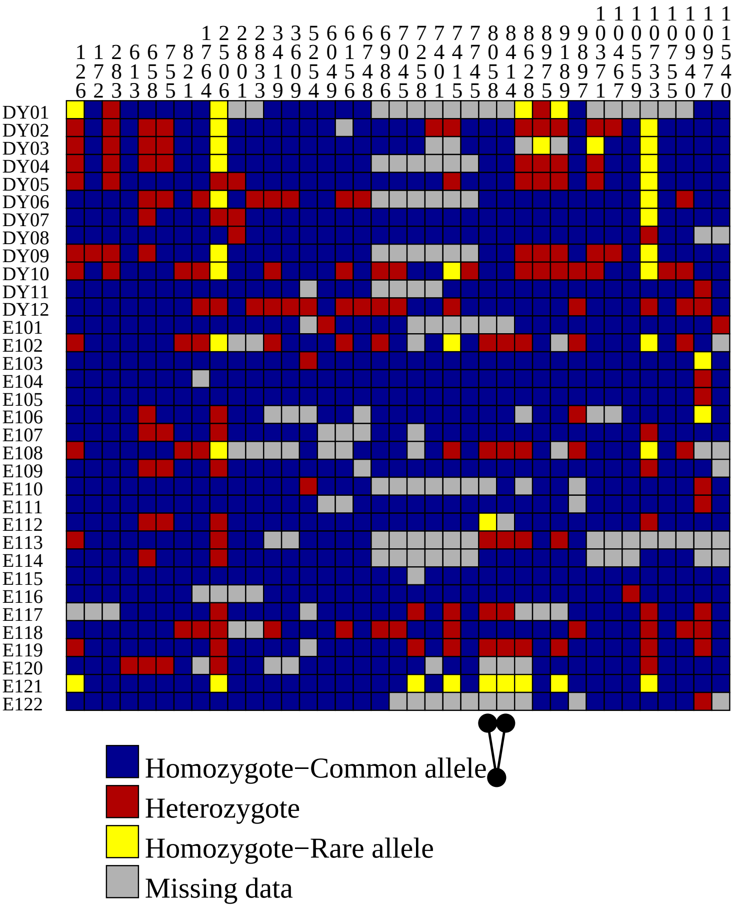

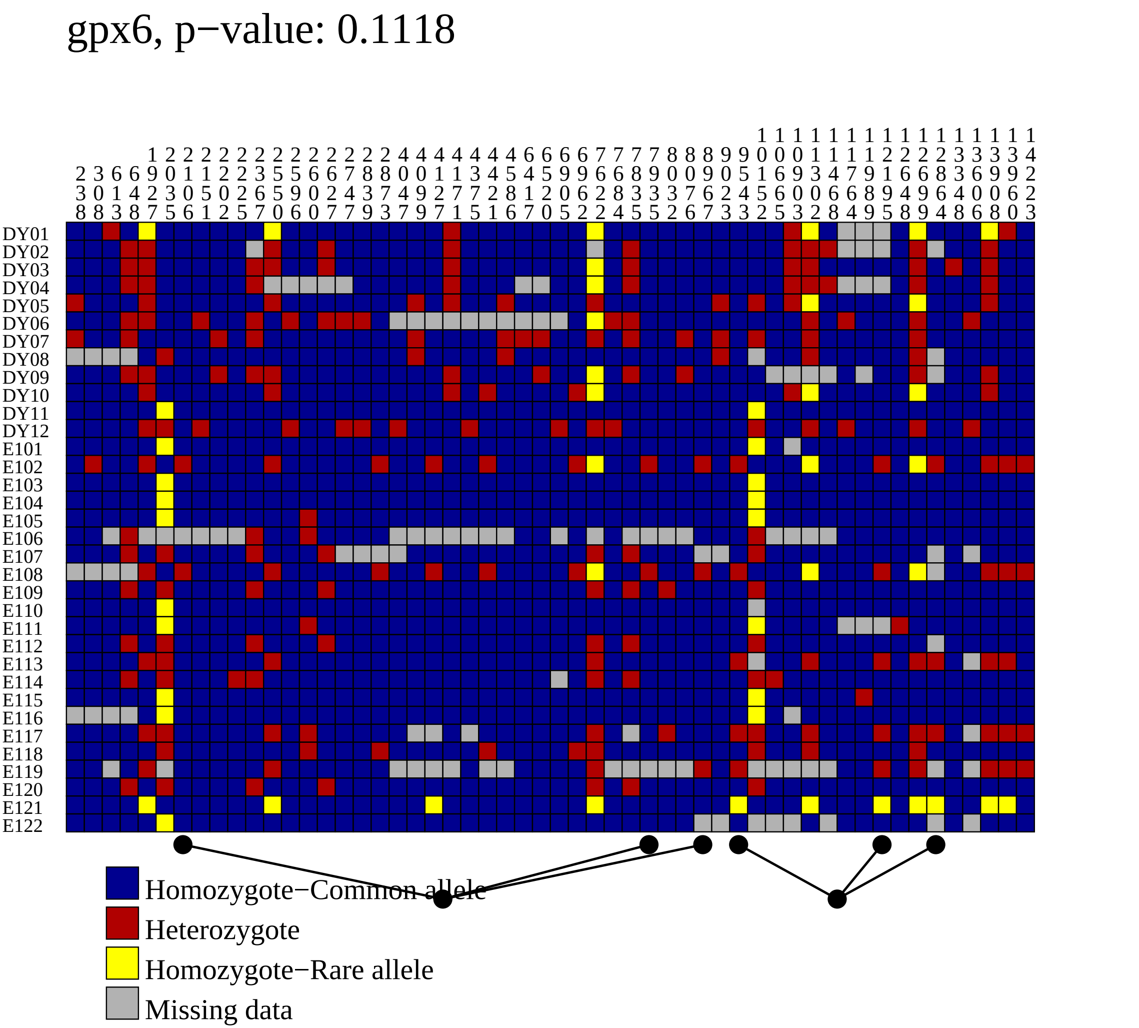
gpx6, p−value: 0.1118
238
308
613
648
1927
2035
2106
2151
2202
2225
2367
2550
2596
2600
2627
2747
2839
2873
4047
4099
4127
4171
4375
4421
4586
6417
6520
6905
6962
7622
7684
7835
7935
8032
8076
8967
9023
9543
10152
10665
10903
11302
11468
11764
11989
12195
12648
12699
12864
13348
13606
13908
13920
14223
DY01
DY02
DY03
DY04
DY05
DY06
DY07
DY08
DY09
DY10
DY11
DY12
E101
E102
E103
E104
E105
E106
E107
E108
E109
E110
E111
E112
E113
E114
E115
E116
E117
E118
E119
E120
E121
E122
Homozygote−Common allele
Heterozygote
Homozygote−Rare allele
Missing data

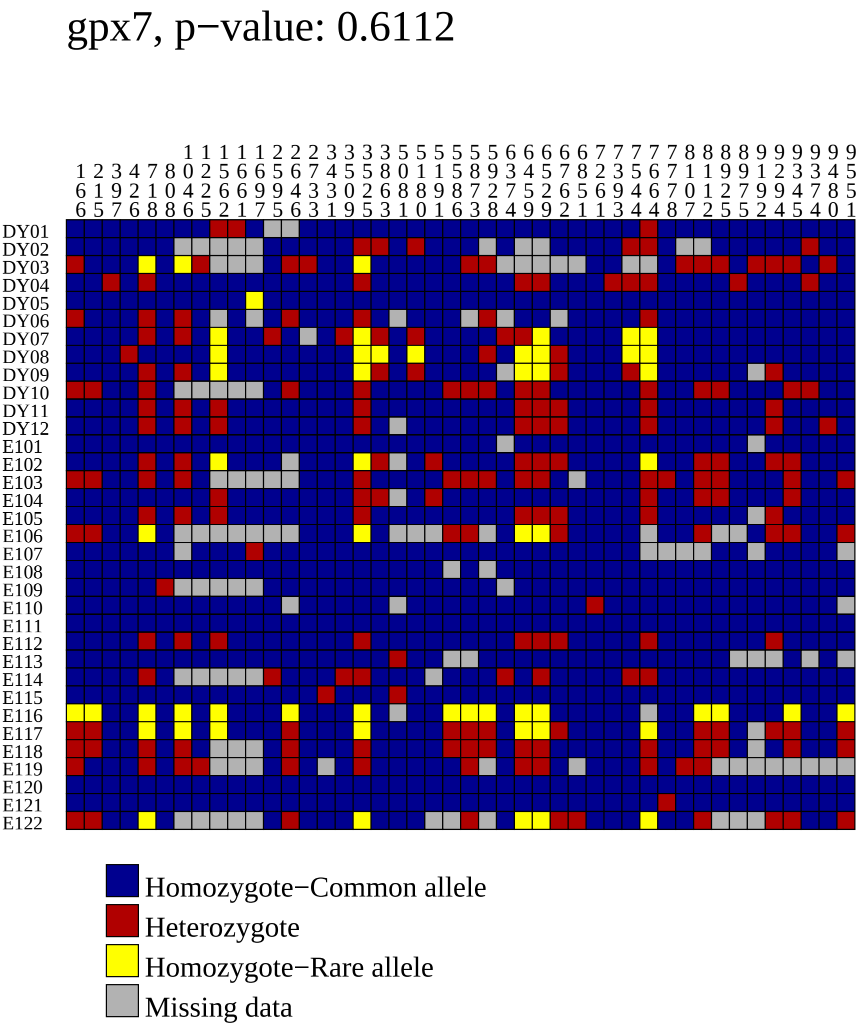
gpx7, p−value: 0.6112
166 215 397 426 718 808 1046 1125 1562 1661 1697 2595 2646 2733 3431 3509 3525 3863 5081 5180 5191 5586 5873 5928 6374 6459 6529 6762 6851 7261 7393 7544 7664 7778 8107 8112 8925 8975 9192 9294 9345 9374 9480 9551
DY01 DY02 DY03 DY04 DY05 DY06 DY07 DY08 DY09 DY10 DY11 DY12 E101 E102 E103 E104 E105 E106 E107 E108 E109 E110 E111 E112 E113 E114 E115 E116 E117 E118 E119 E120 E121 E122
Homozygote−Common allele
Heterozygote
Homozygote−Rare allele
Missing data

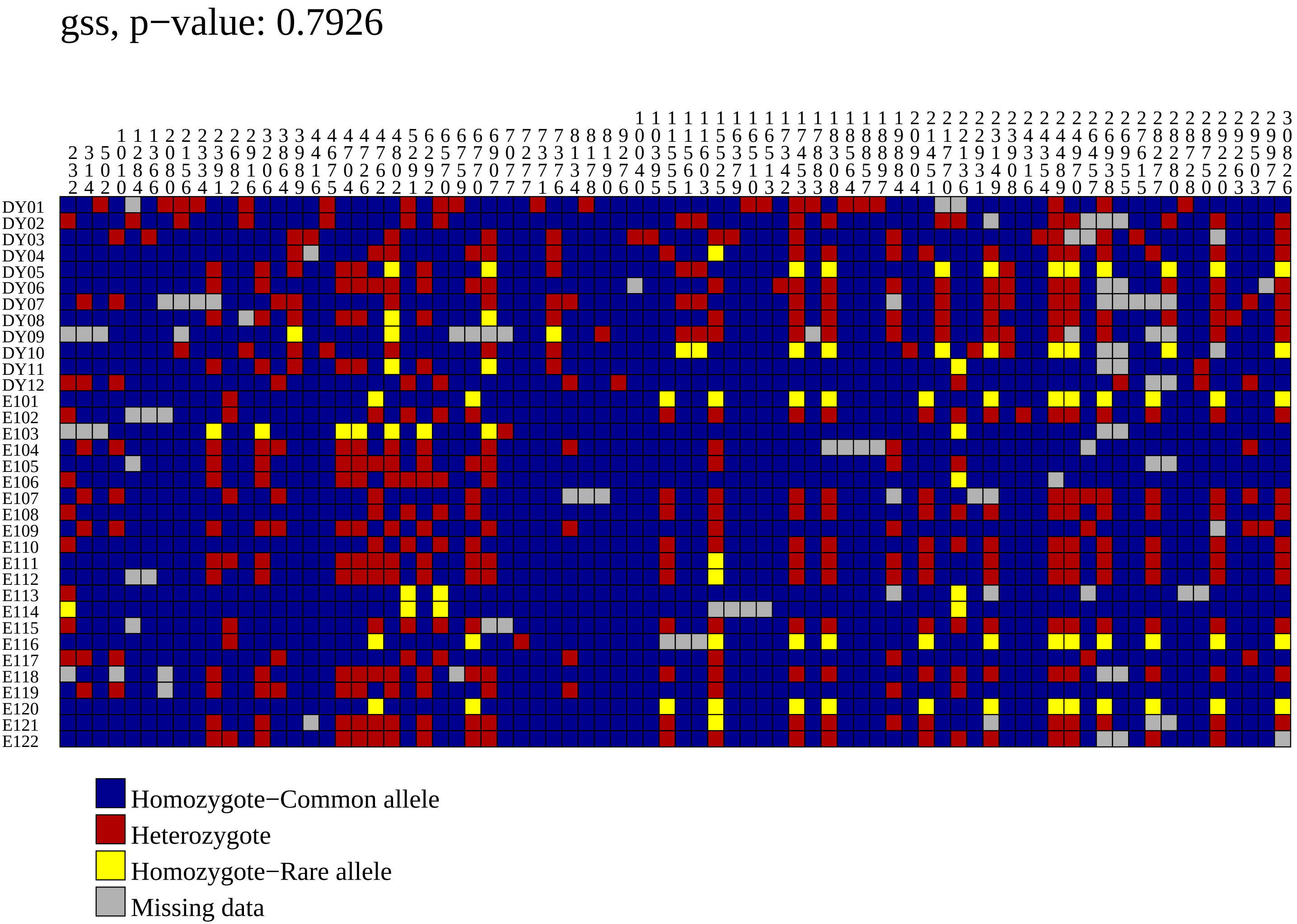
gss, p−value: 0.7926
Homozygote−Common allele
Heterozygote
Homozygote−Rare allele
Missing data

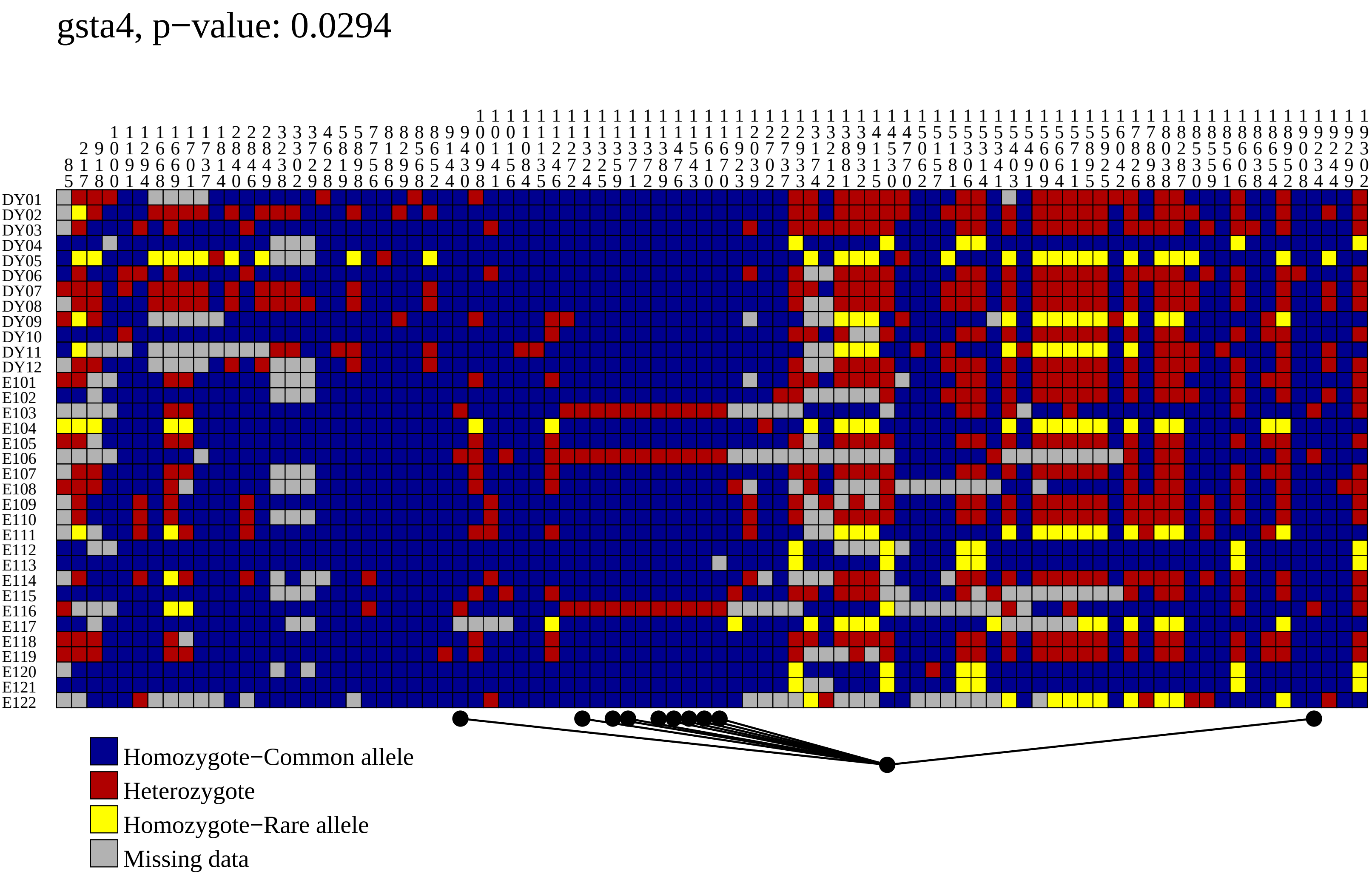
gsta4, p−value: 0.0294
DY01
DY02
DY03
DY04
DY05
DY06
DY07
DY08
DY09
DY10
DY11
DY12
E101
E102
E103
E104
E105
E106
E107
E108
E109
E110
E111
E112
E113
E114
E115
E116
E117
E118
E119
E120
E121
E122
Homozygote−Common allele
Heterozygote
Homozygote−Rare allele
Missing data

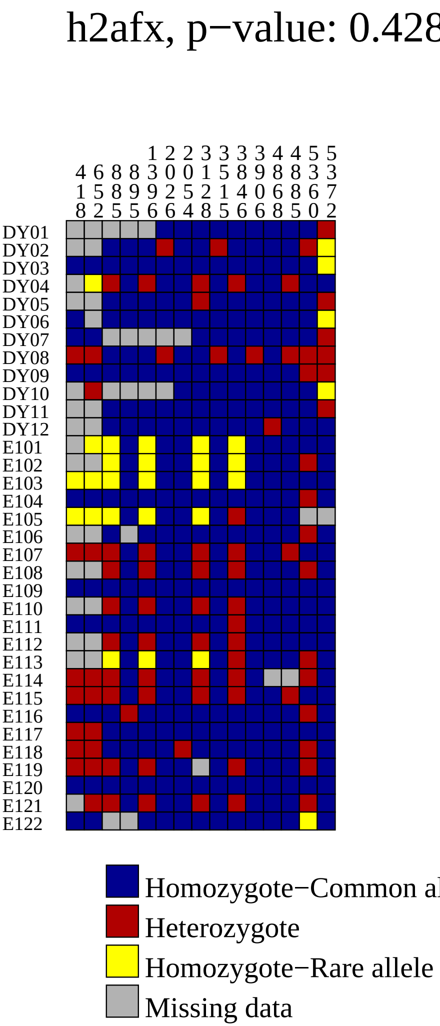
h2afx, p−value: 0.428
418
652
885
895
1396
2026
2054
3128
3515
3846
3906
4868
4885
5360
5372
DY01
DY02
DY03
DY04
DY05
DY06
DY07
DY08
DY09
DY10
DY11
DY12
E101
E102
E103
E104
E105
E106
E107
E108
E109
E110
E111
E112
E113
E114
E115
E116
E117
E118
E119
E120
E121
E122
Homozygote−Common al
Heterozygote
Homozygote−Rare allele
Missing data

ifna1, p−value: 0.5156

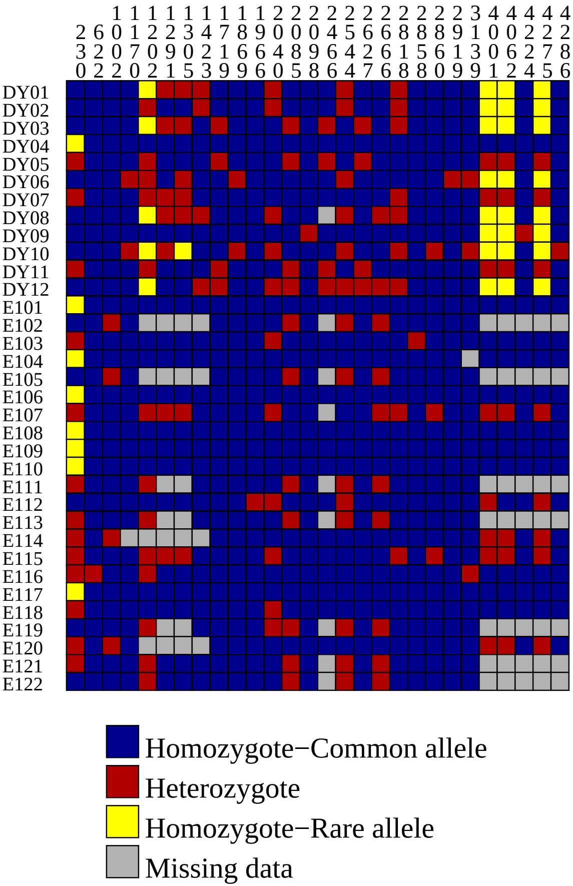

itgal, p−value: 0.3874

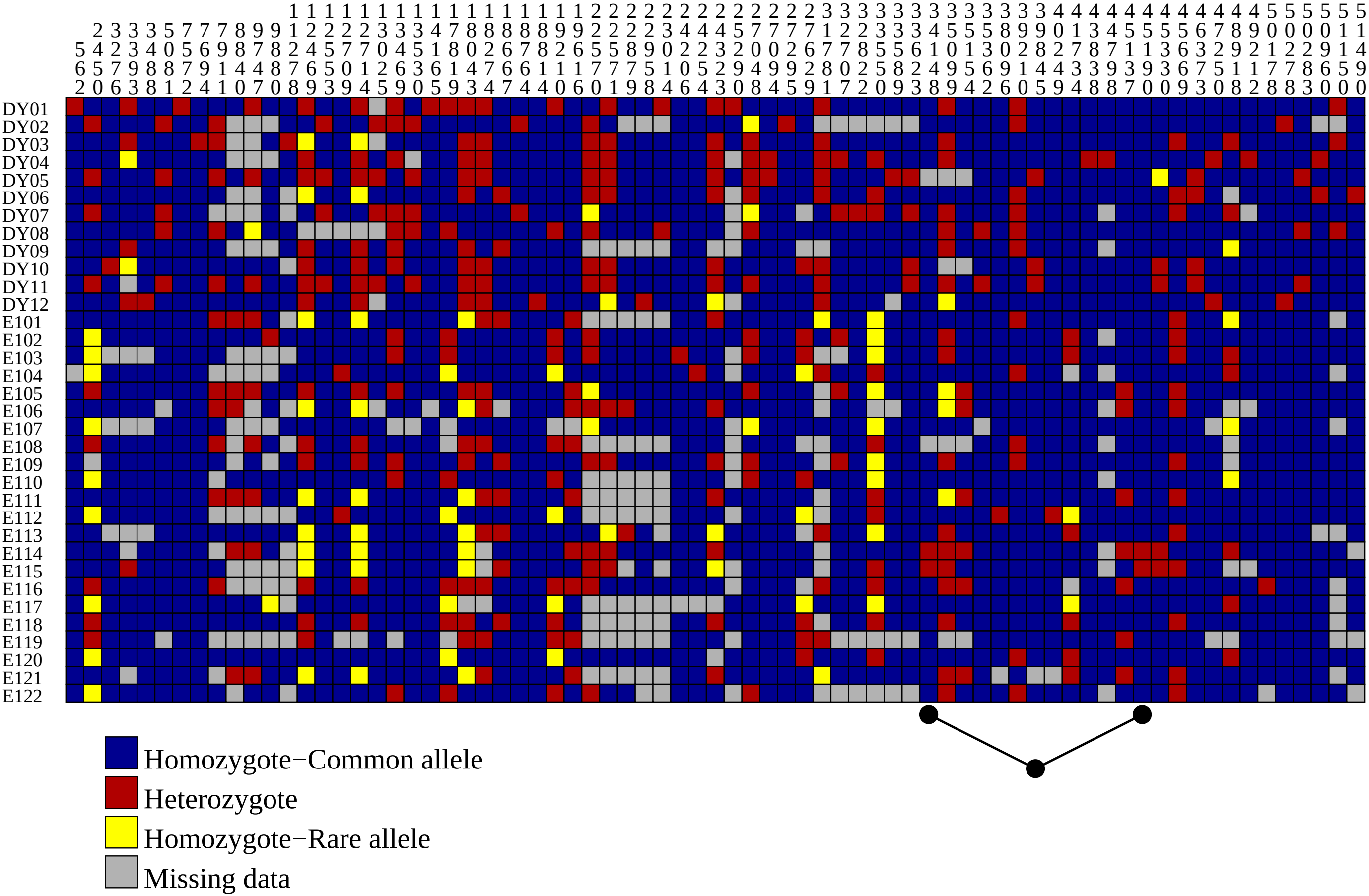

lcmt2, p−value: 0.4486

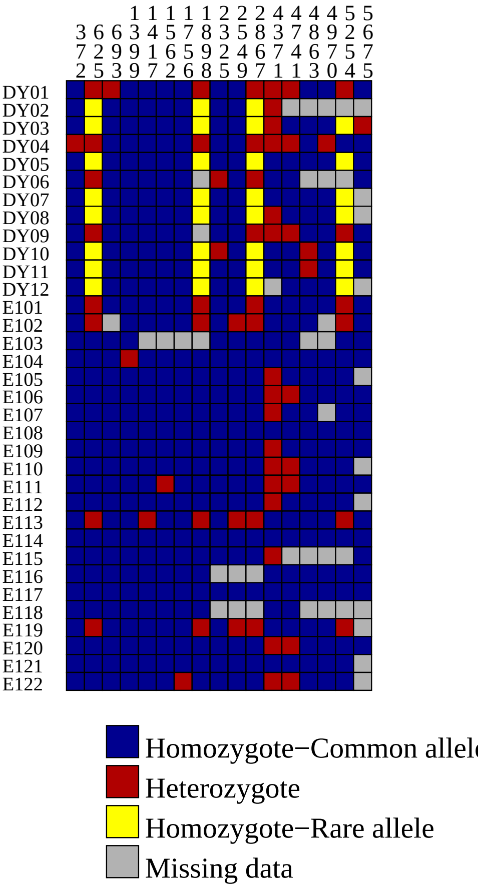

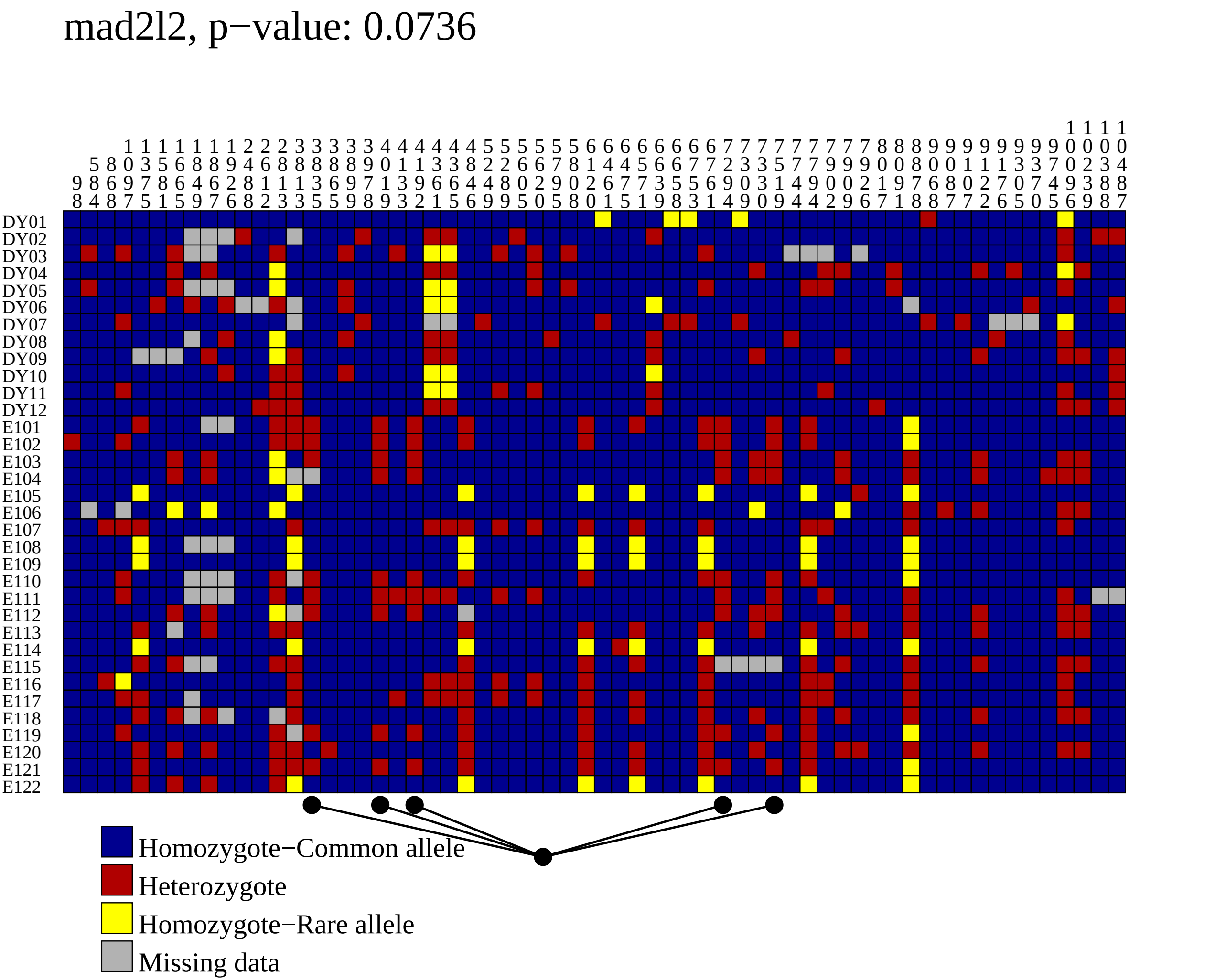
mad2l2, p−value: 0.0736
Homozygote−Common allele
Heterozygote
Homozygote−Rare allele
Missing data

map2k4, p−value: 0.8604

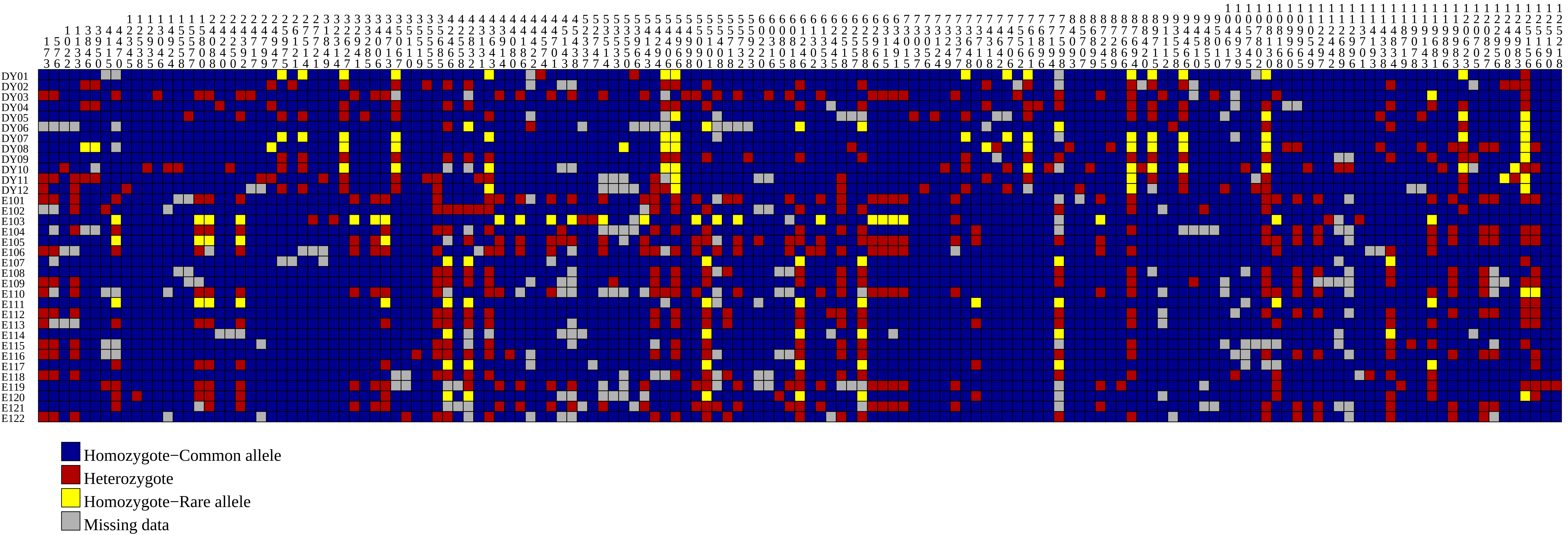

mapk9, p−value: 0.243

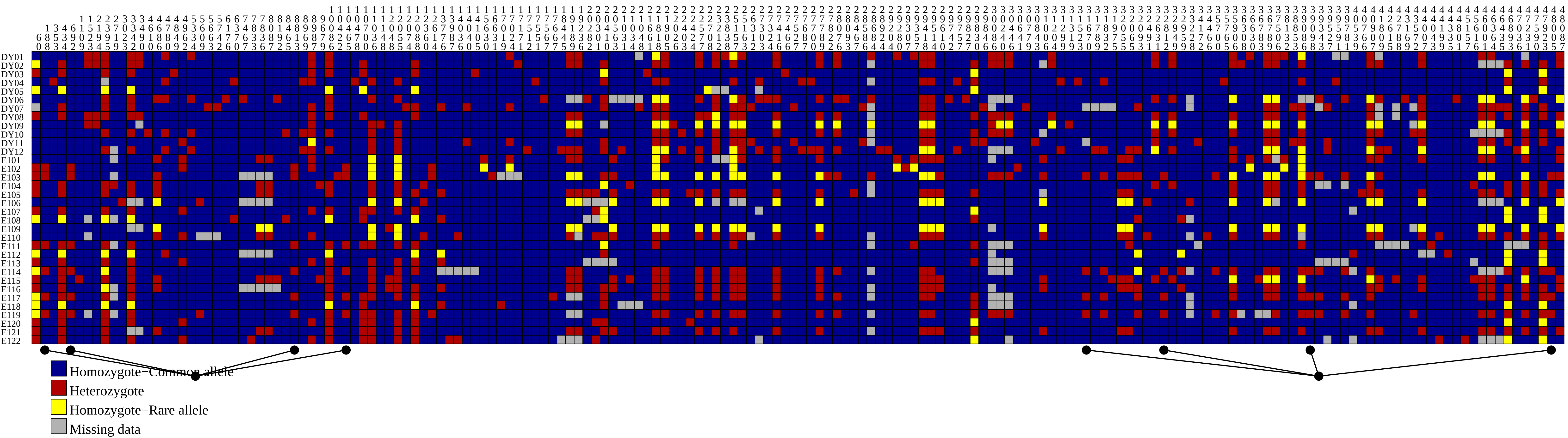

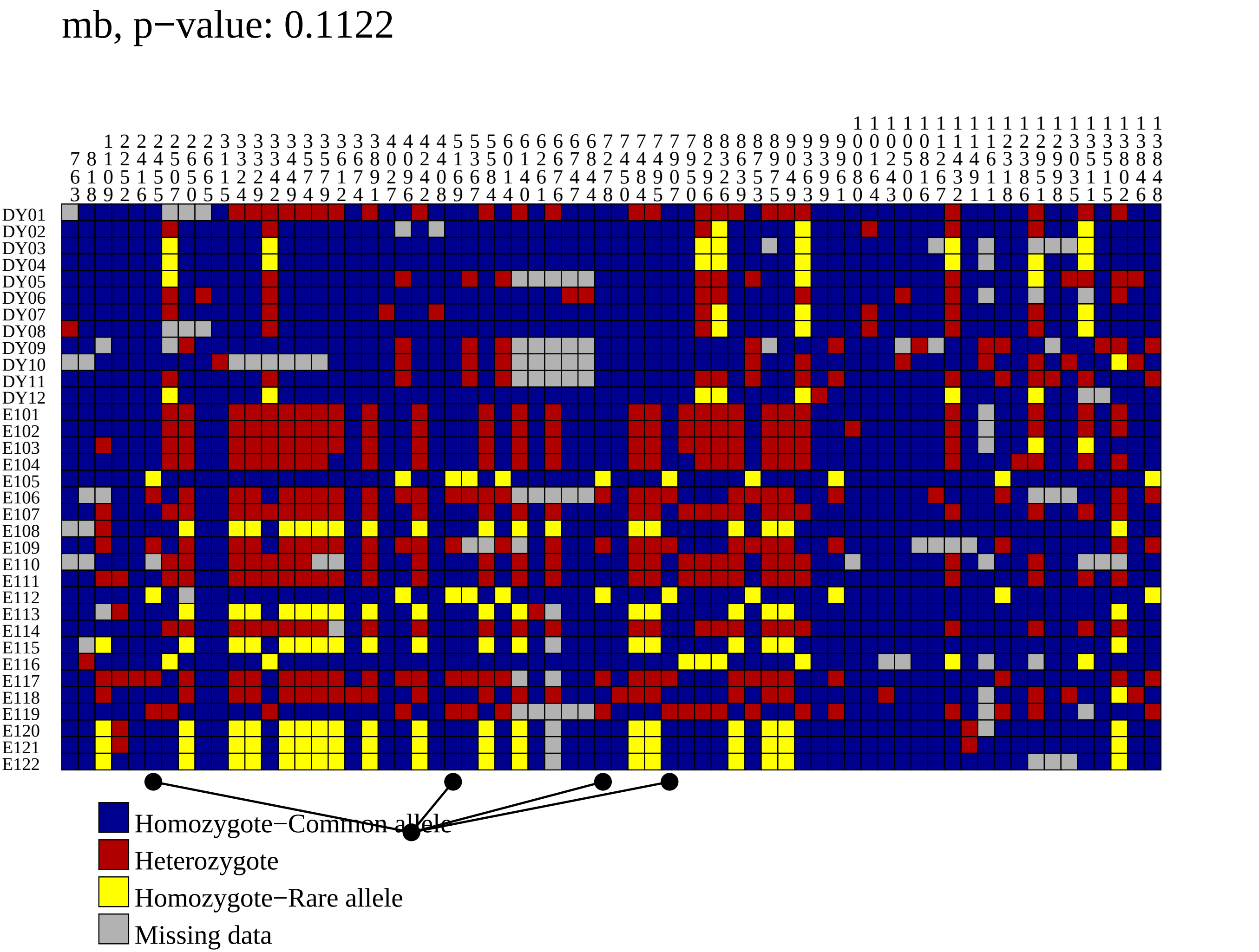
mb, p−value: 0.1122
Homozygote−Common allele
Heterozygote
Homozygote−Rare allele
Missing data

mcl1, p−value: 0.4808

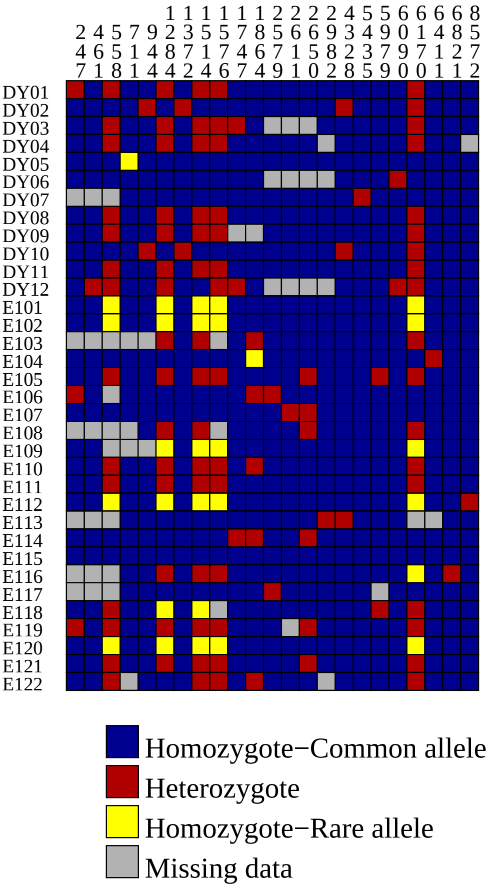

mlh3, p−value: 0.8542

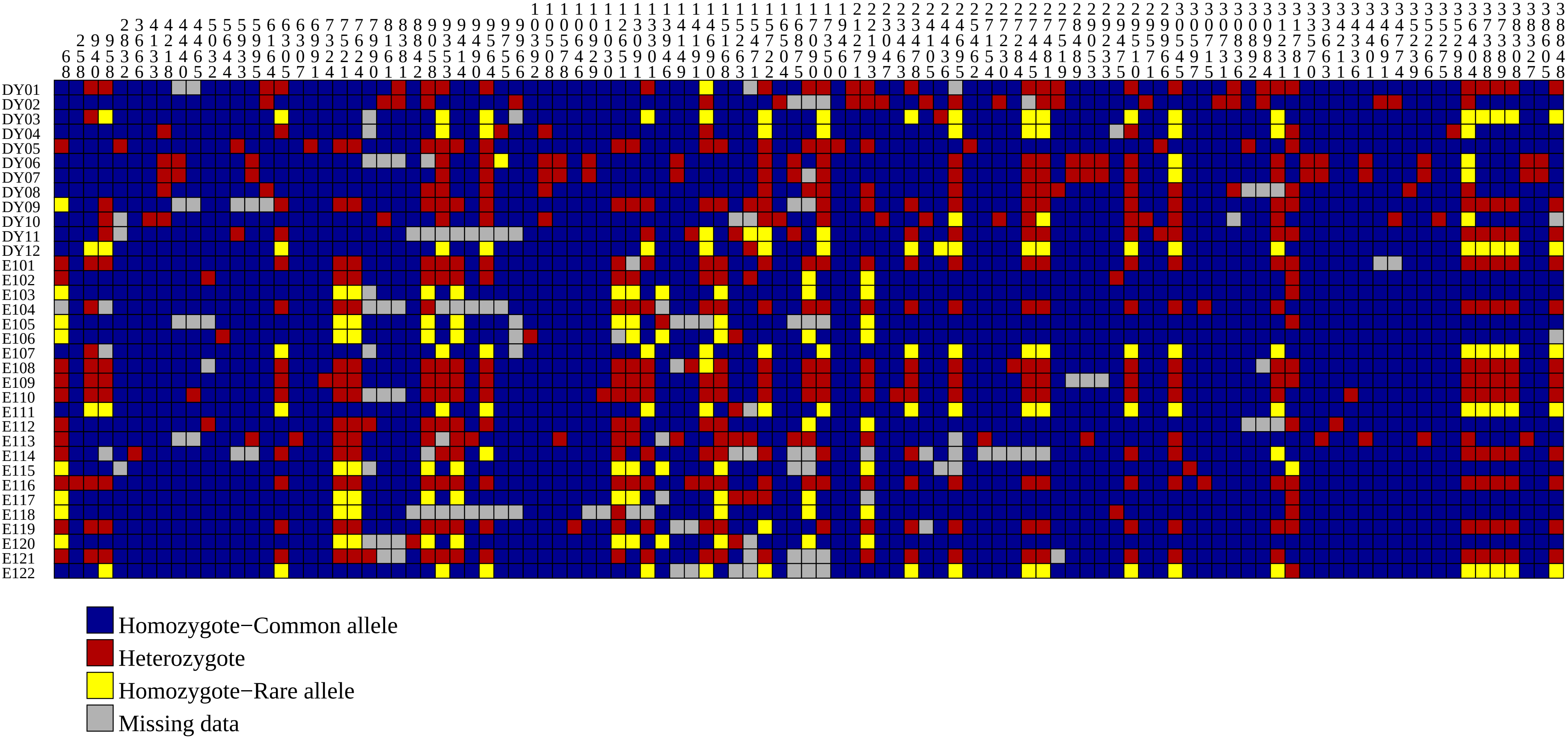

mmp11, p−value: 0.3752

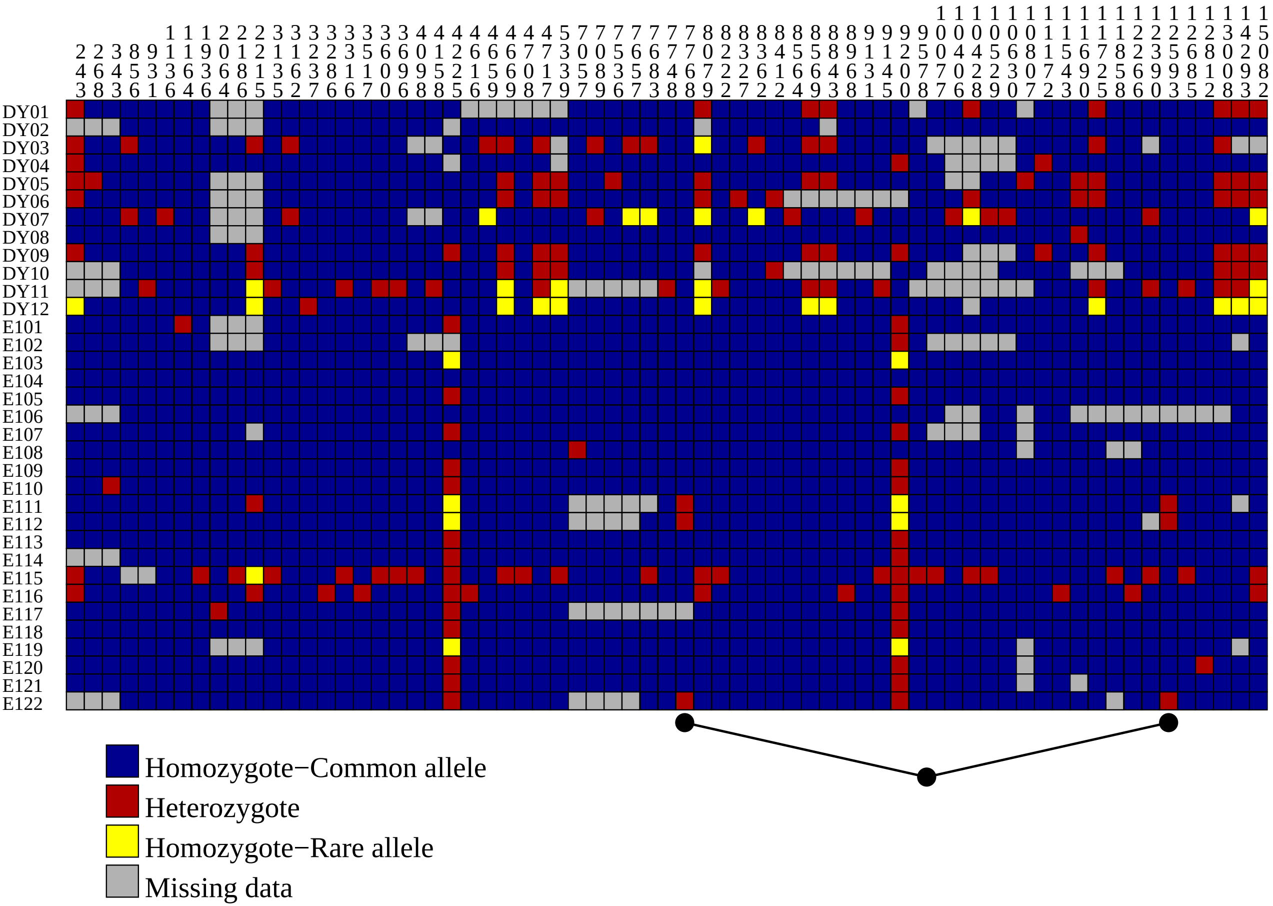

mmp12, p−value: 0.0508

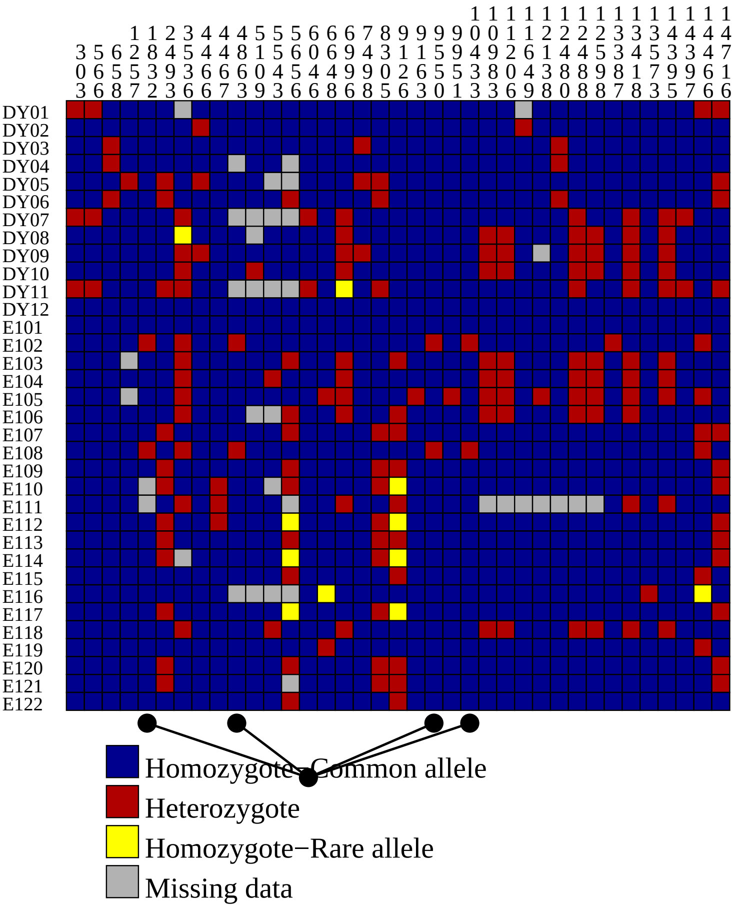

mmp16, p−value: 0.144

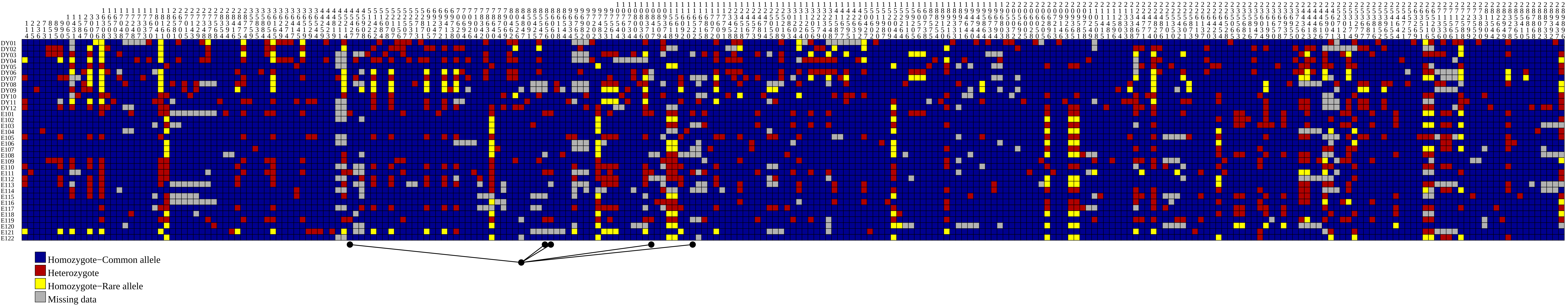

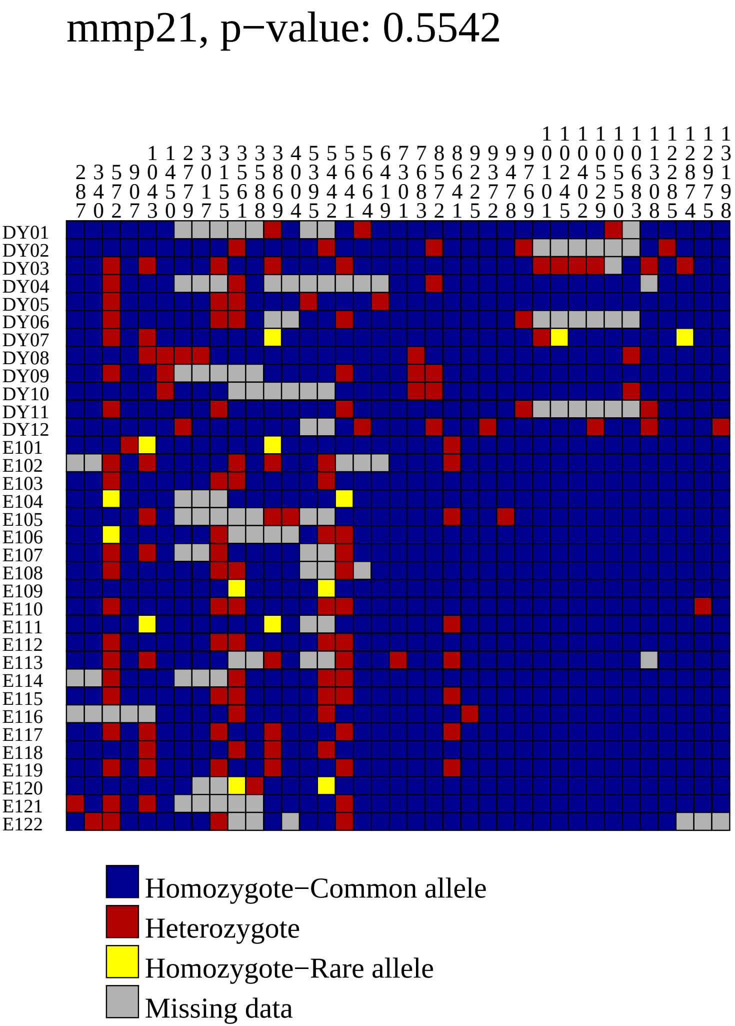
mmp21, p−value: 0.5542
Homozygote−Common allele
Heterozygote
Homozygote−Rare allele
Missing data

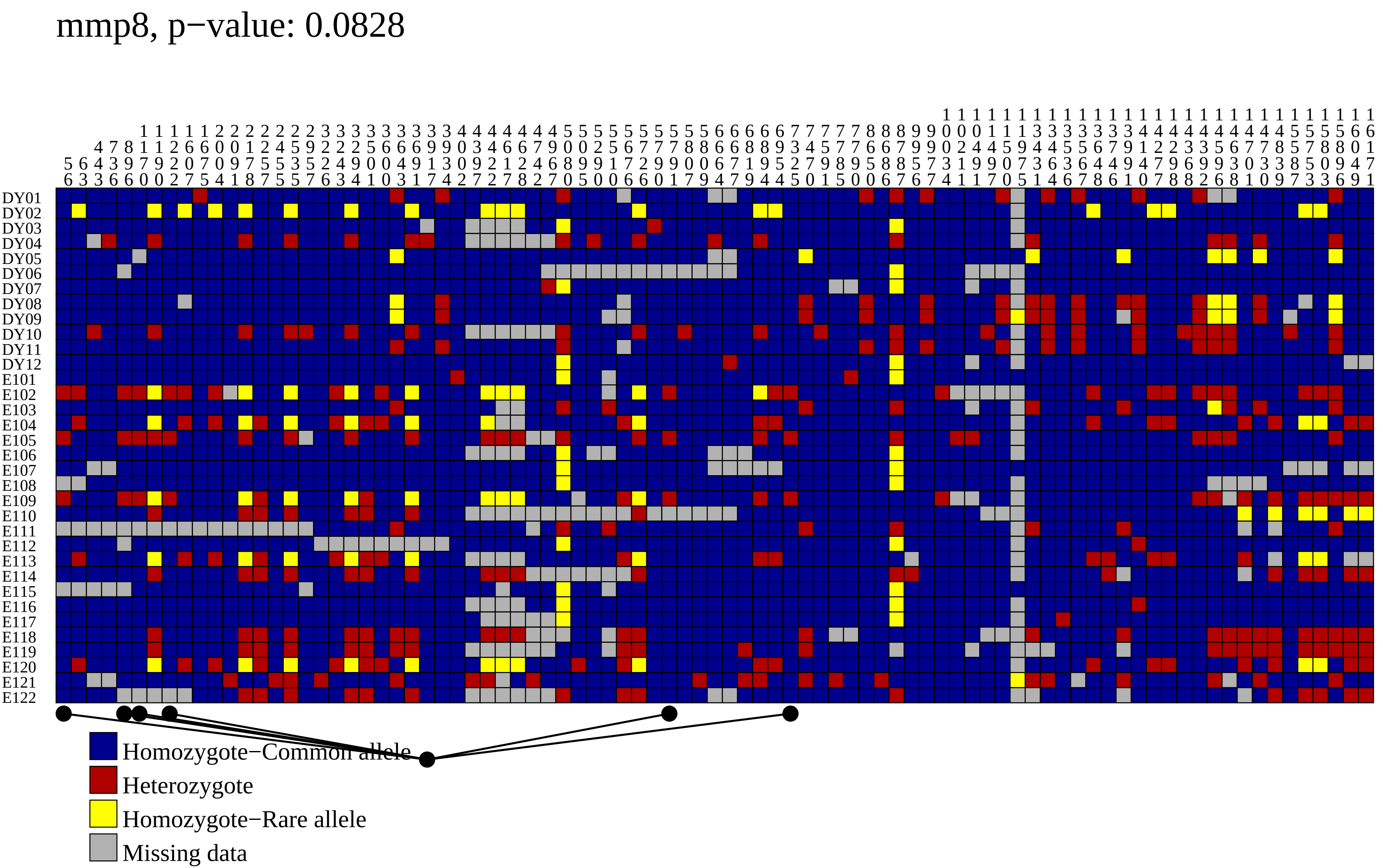
mmp8, p−value: 0.0828
DY01
DY02
DY03
DY04
DY05
DY06
DY07
DY08
DY09
DY10
DY11
DY12
E101
E102
E103
E104
E105
E106
E107
E108
E109
E110
E111
E112
E113
E114
E115
E116
E117
E118
E119
E120
E121
E122
Homozygote−Common allele
Heterozygote
Homozygote−Rare allele
Missing data

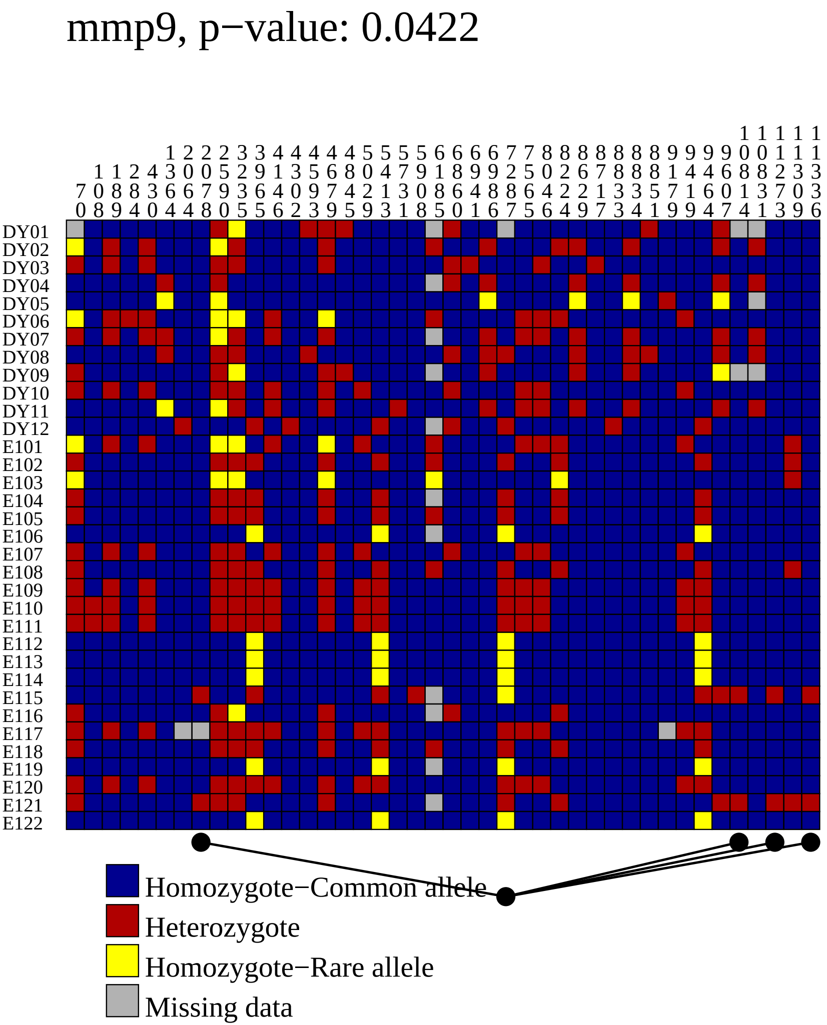
mmp9, p−value: 0.0422
70 108 189 284 430 1364 2064 2078 2590 3235 3965 4146 4302 4593 4679 4845 5029 5413 5731 5908 6185 6860 6941 6986 7287 7565 8046 8224 8629 8717 8833 8834 8851 9179 9419 9464 9607 10814 10831 11273 11309 11336
DY01 DY02 DY03 DY04 DY05 DY06 DY07 DY08 DY09 DY10 DY11 DY12
E101 E102 E103 E104 E105 E106 E107 E108 E109 E110 E111 E112 E113 E114 E115 E116 E117 E118 E119 E120 E121 E122
Homozygote−Common allele
Heterozygote
Homozygote−Rare allele
Missing data

mms19l, p−value: 0.3118

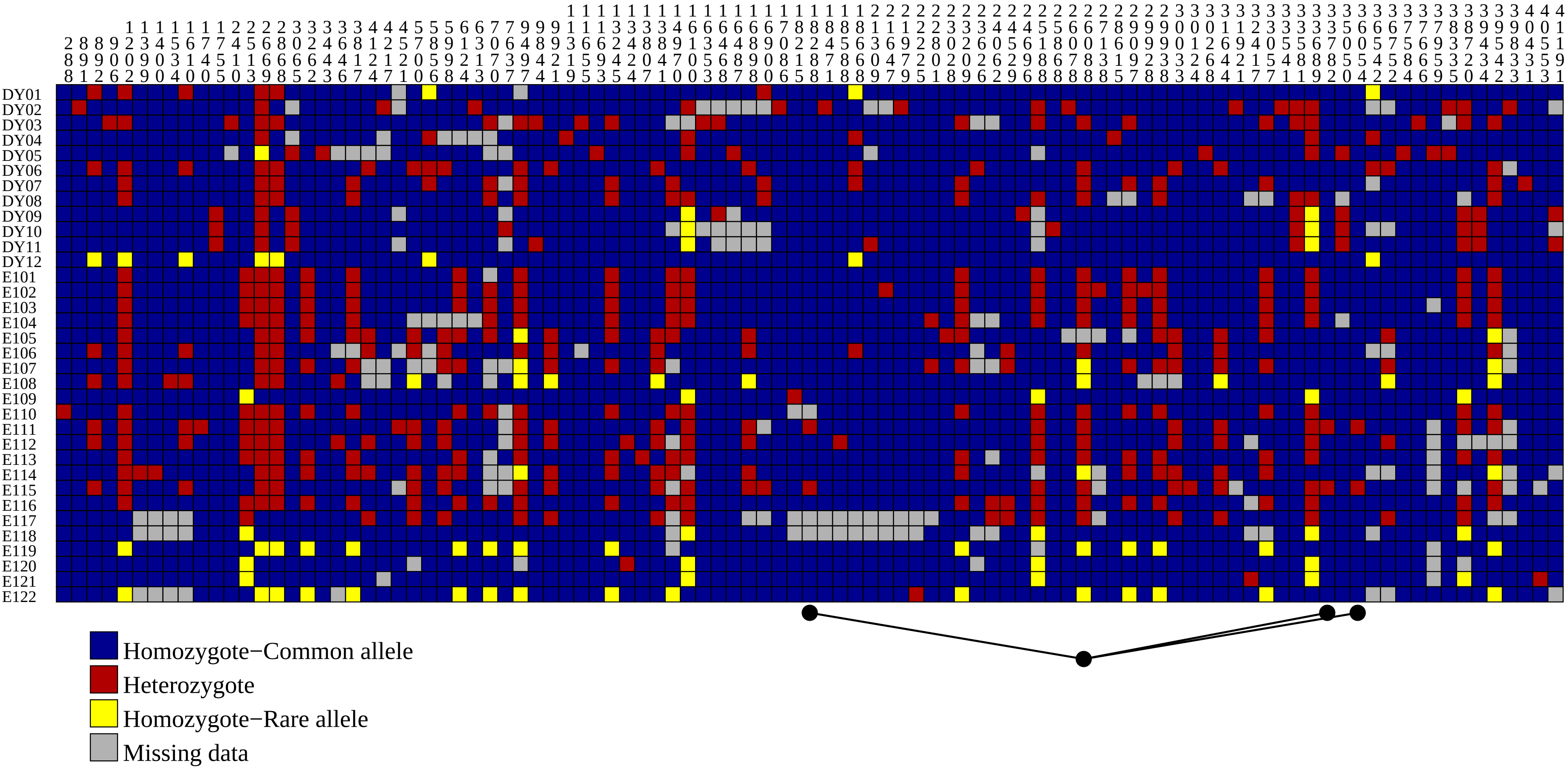

mpo, p−value: 0.581

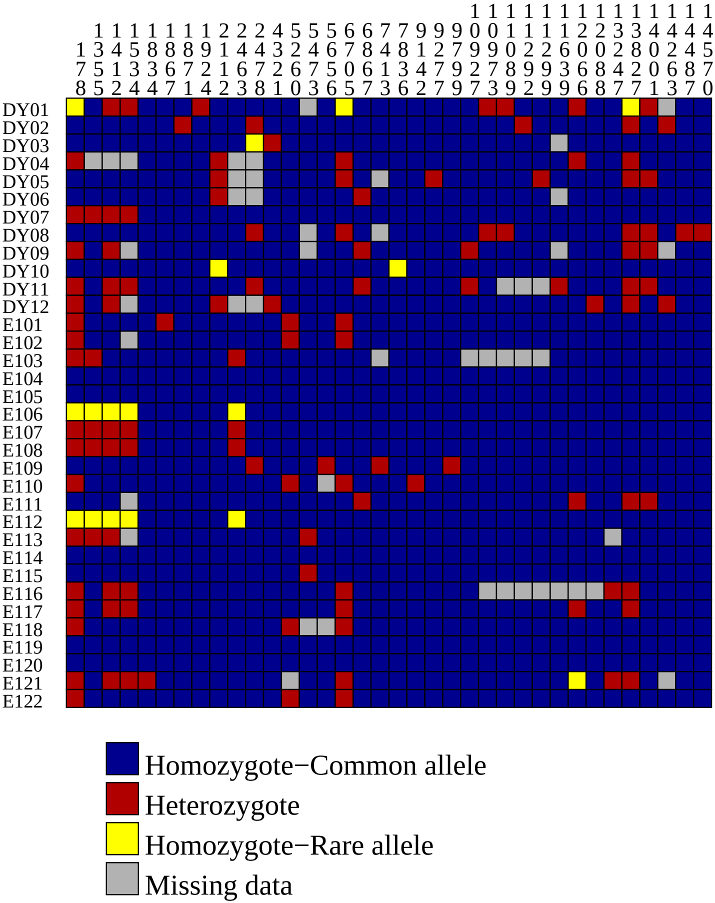

msh5, p−value: 0.7106

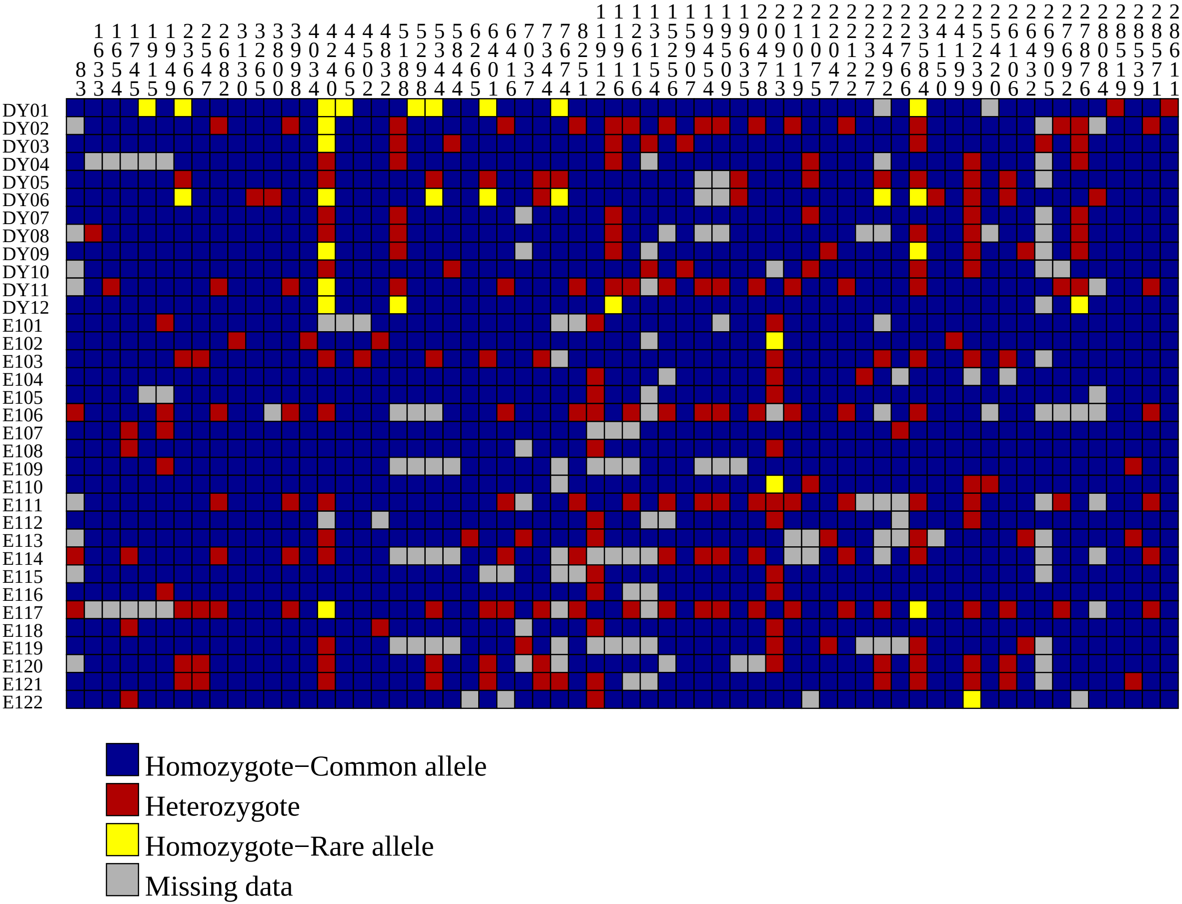

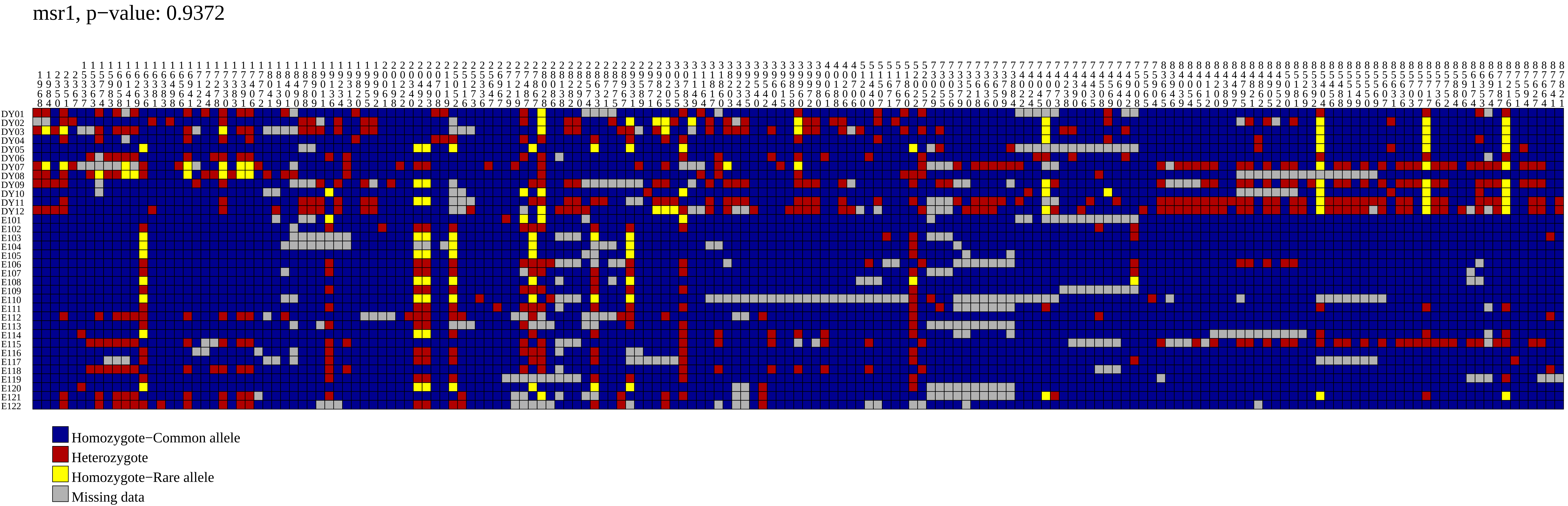
msr1, p−value: 0.9372
DY01
DY02
DY03
DY04
DY05
DY06
DY07
DY08
DY09
DY10
DY11
DY12
E101
E102
E103
E104
E105
E106
E107
E108
E109
E110
E111
E112
E113
E114
E115
E116
E117
E118
E119
E120
E121
E122
Homozygote−Common allele
Heterozygote
Homozygote−Rare allele
Missing data

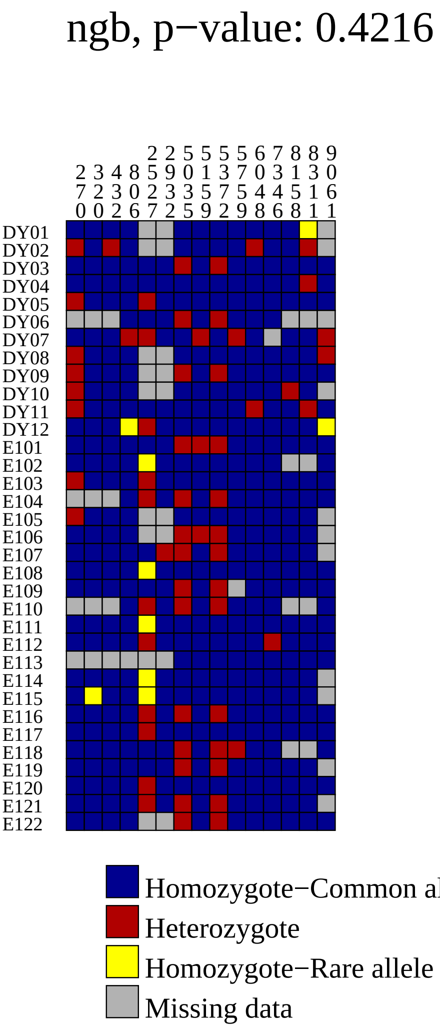
ngb, p−value: 0.4216
270
320
432
806
2527
2932
5035
5159
5372
5759
6048
7346
8158
8311
9061
DY01
DY02
DY03
DY04
DY05
DY06
DY07
DY08
DY09
DY10
DY11
DY12
E101
E102
E103
E104
E105
E106
E107
E108
E109
E110
E111
E112
E113
E114
E115
E116
E117
E118
E119
E120
E121
E122
Homozygote−Common al
Heterozygote
Homozygote−Rare allele
Missing data

nos2a, p−value: 0.8476

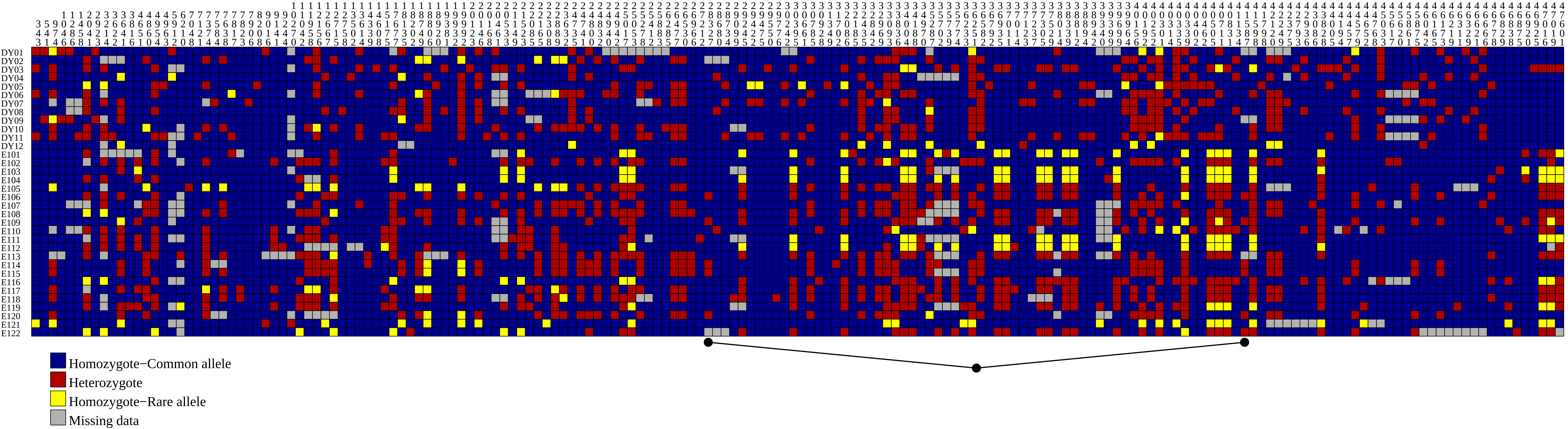

nudt1, p−value: 0.11

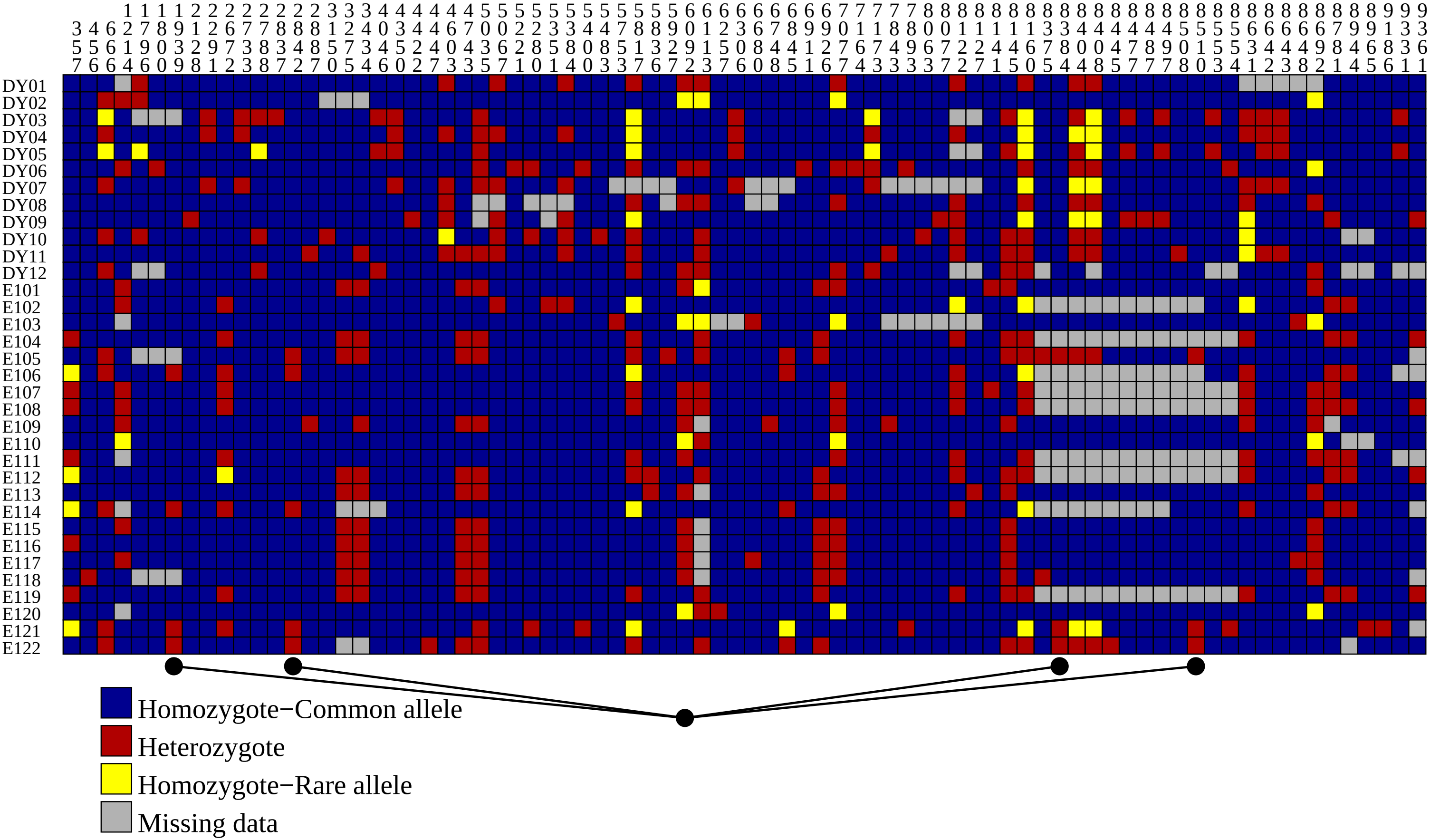

odc1, p−value: 0.0416

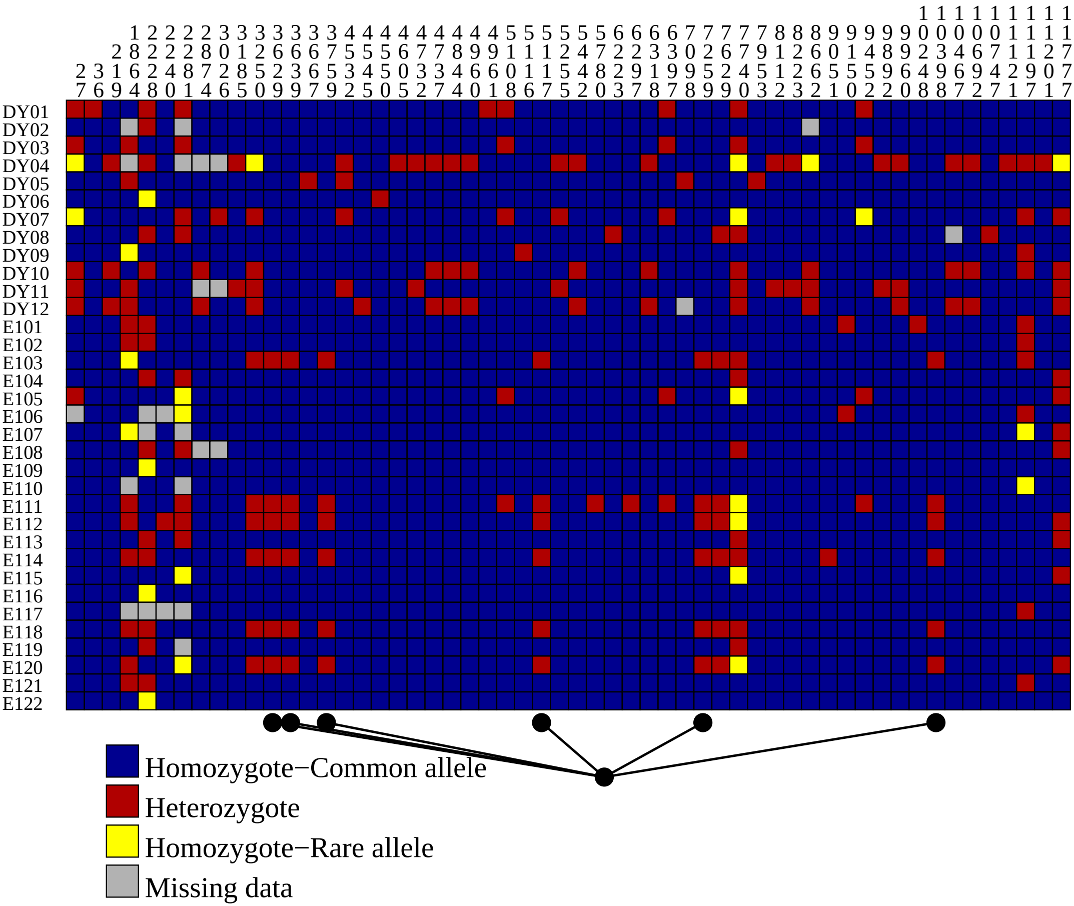

osr1, p−value: 0.5938

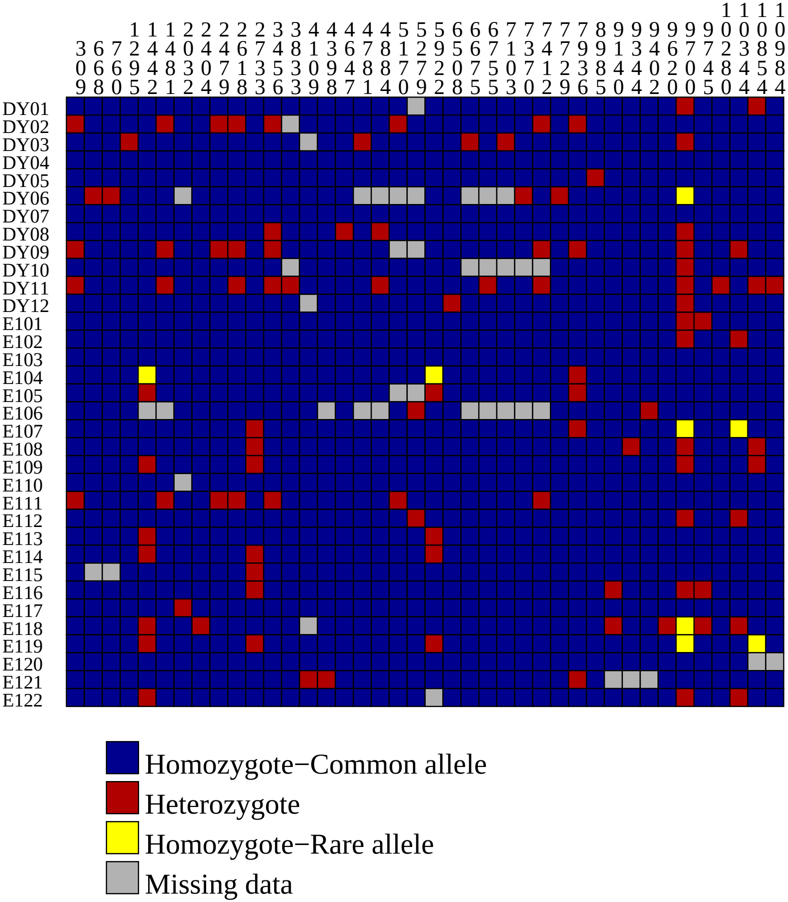

oxr1, p−value: 0.0094

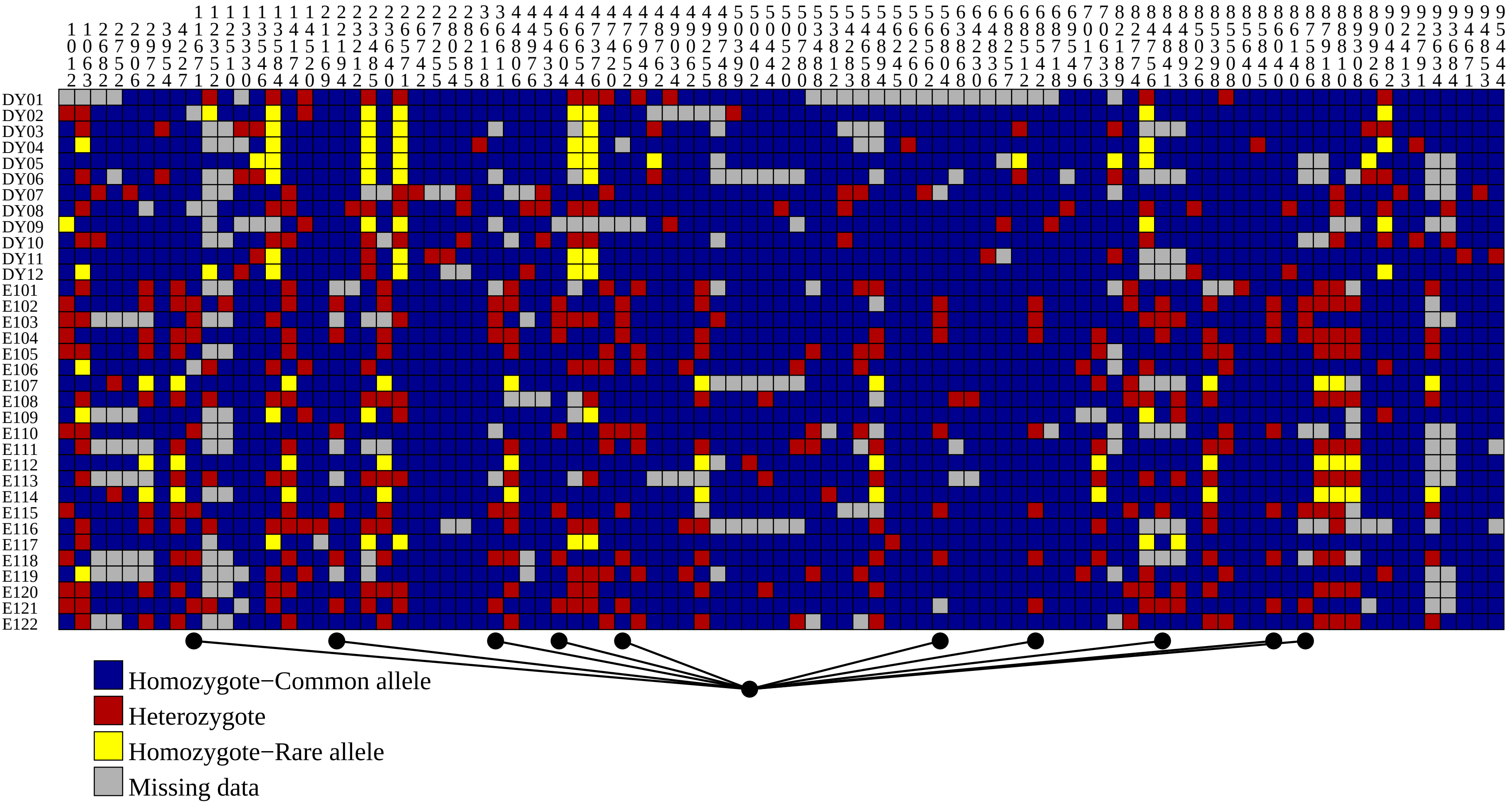

oxsr1, p−value: 0.2496

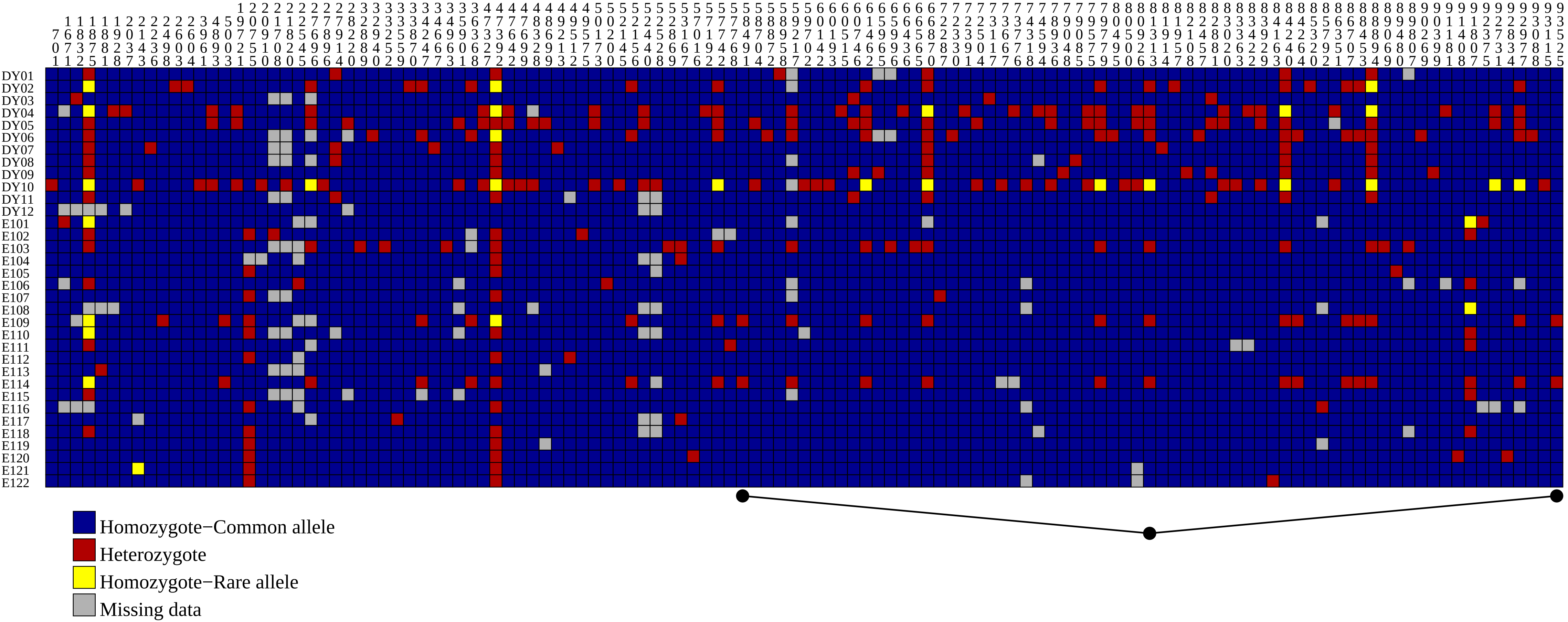

pax3, p−value: 0.1876

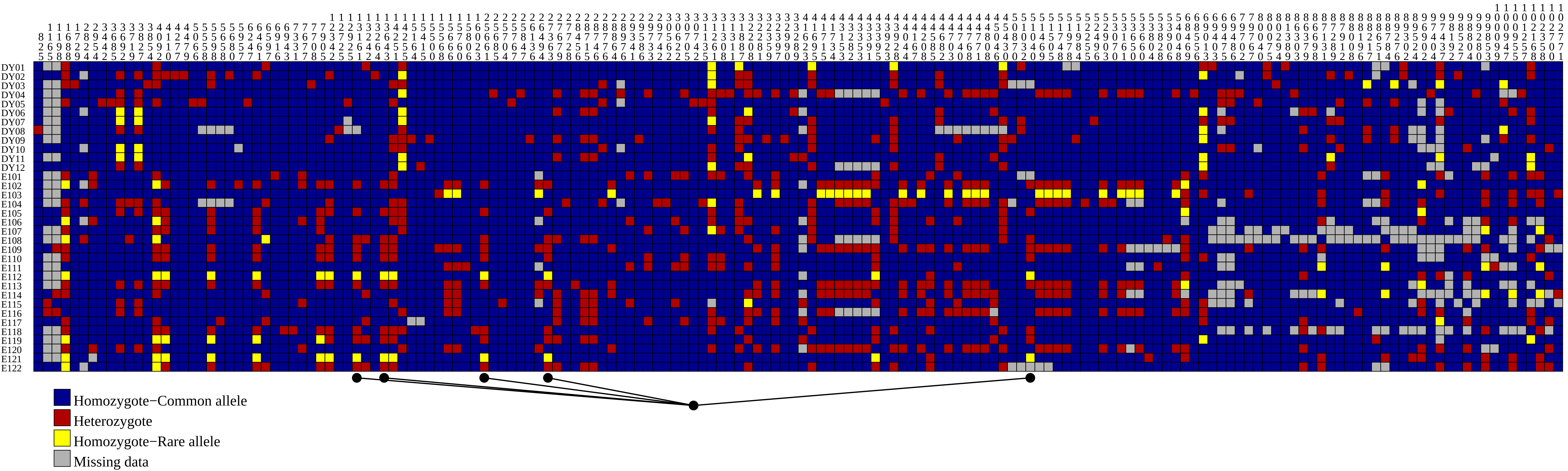

pdlim1, p−value: 0.2652

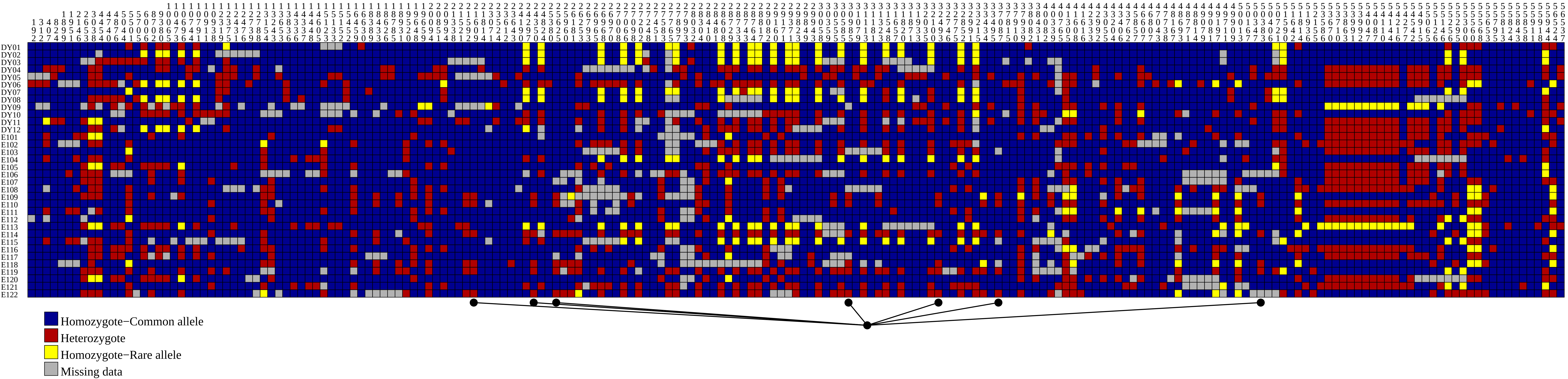

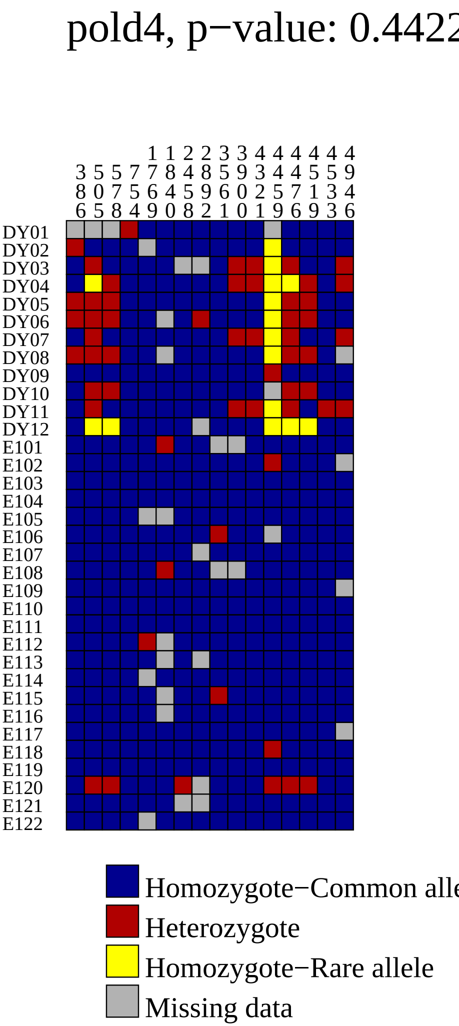
pold4, p−value: 0.4422
386
505
578
754
1769
1840
2458
2892
3561
3900
4321
4459
4476
4519
4533
4946
DY01
DY02
DY03
DY04
DY05
DY06
DY07
DY08
DY09
DY10
DY11
DY12
E101
E102
E103
E104
E105
E106
E107
E108
E109
E110
E111
E112
E113
E114
E115
E116
E117
E118
E119
E120
E121
E122
Homozygote−Common alle
Heterozygote
Homozygote−Rare allele
Missing data

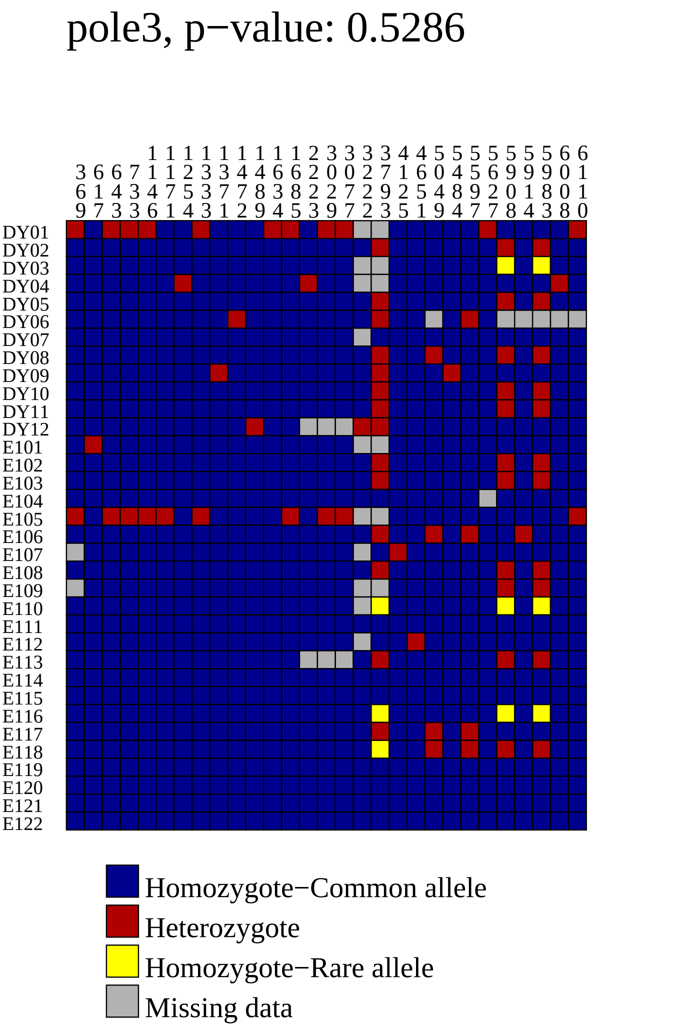
pole3, p−value: 0.5286
369 617 643 733 1146 1171 1254 1333 1371 1472 1489 1634 1685 2223 3029 3077 3222 3793 4125 4651 5049 5484 5597 5627 5908 5914 5983 6008 6110
DY01
DY02
DY03
DY04
DY05
DY06
DY07
DY08
DY09
DY10
DY11
DY12
E101
E102
E103
E104
E105
E106
E107
E108
E109
E110
E111
E112
E113
E114
E115
E116
E117
E118
E119
E120
E121
E122
Homozygote−Common allele
Heterozygote
Homozygote−Rare allele
Missing data

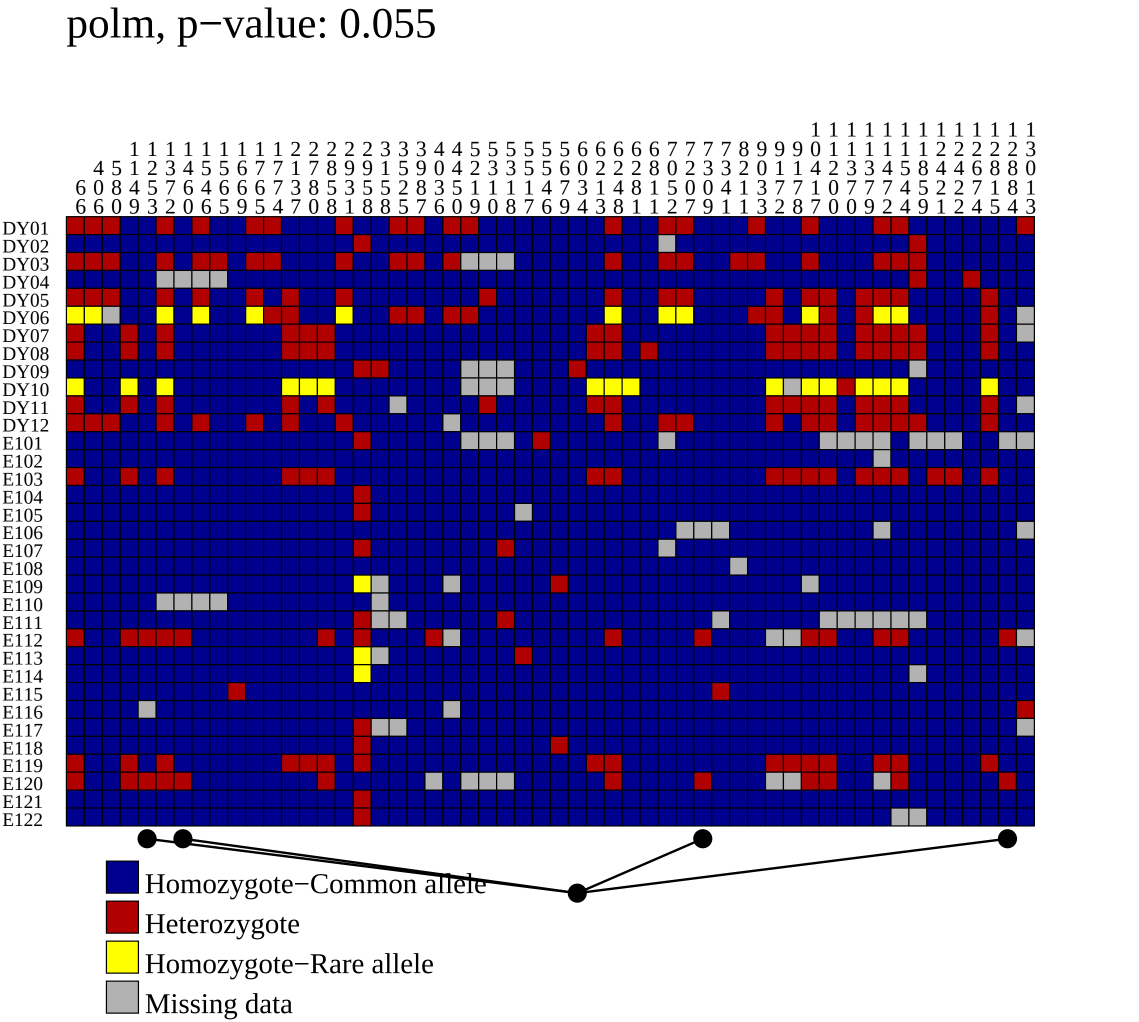
polm, p−value: 0.055
66
406
580
1149
1253
1372
1460
1546
1565
1669
1675
1774
2137
2780
2858
2931
2958
3158
3525
3987
4036
4450
5219
5310
5318
5517
5546
5679
6034
6213
6248
6281
6812
7052
7207
7309
7341
8211
9033
9172
9178
10417
11020
11370
11379
11472
11544
11859
12421
12422
12674
12815
12884
13013
DY01
DY02
DY03
DY04
DY05
DY06
DY07
DY08
DY09
DY10
DY11
DY12
E101
E102
E103
E104
E105
E106
E107
E108
E109
E110
E111
E112
E113
E114
E115
E116
E117
E118
E119
E120
E121
E122
Homozygote−Common allele
Heterozygote
Homozygote−Rare allele
Missing data

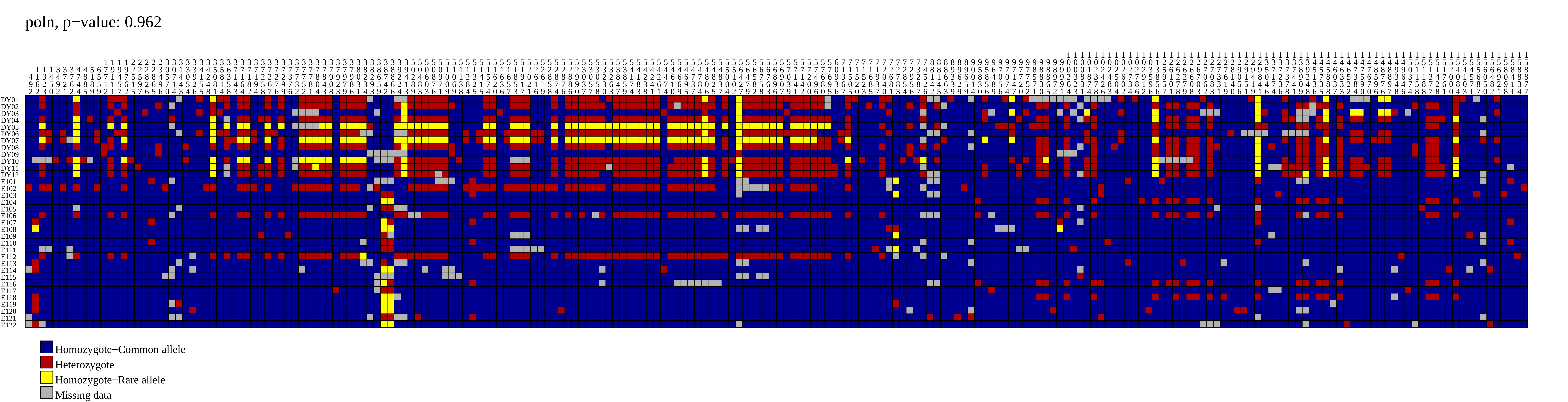
poln, p−value: 0.962
DY01
DY02
DY03
DY04
DY05
DY06
DY07
DY08
DY09
DY10
DY11
DY12
E101
E102
E103
E104
E105
E106
E107
E108
E109
E110
E111
E112
E113
E114
E115
E116
E117
E118
E119
E120
E121
E122
Homozygote−Common allele
Heterozygote
Homozygote−Rare allele
Missing data

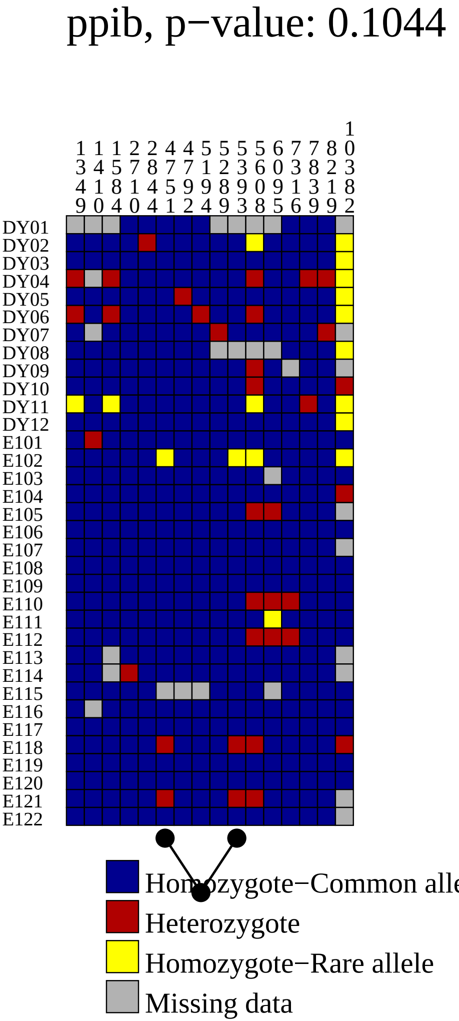
ppib, p−value: 0.1044
1349 1410 1584 2710 2844 4751 4792 5194 5289 5393 5608 6095 7316 7839 8219 10382
DY01
DY02
DY03
DY04
DY05
DY06
DY07
DY08
DY09
DY10
DY11
DY12
E101
E102
E103
E104
E105
E106
E107
E108
E109
E110
E111
E112
E113
E114
E115
E116
E117
E118
E119
E120
E121
E122
Homozygote−Common alle
Heterozygote
Homozygote−Rare allele
Missing data

prdx2, p−value: 0.4446

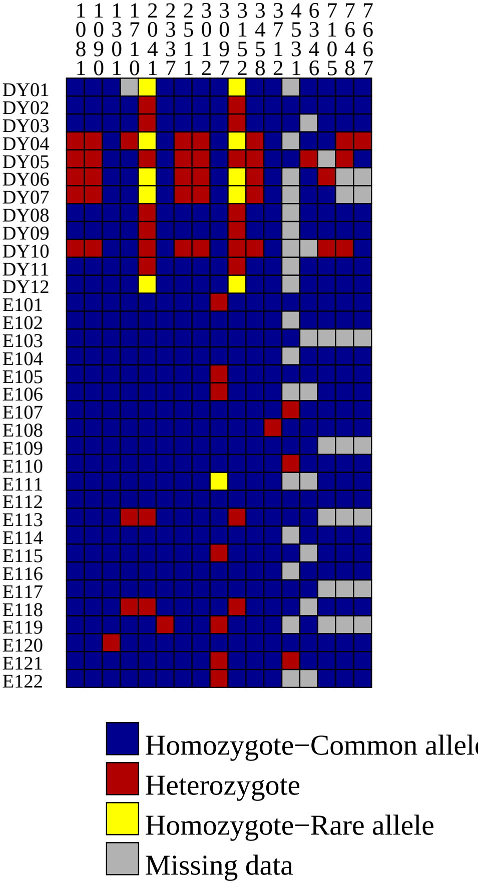

prdx5, p−value: 0.1878

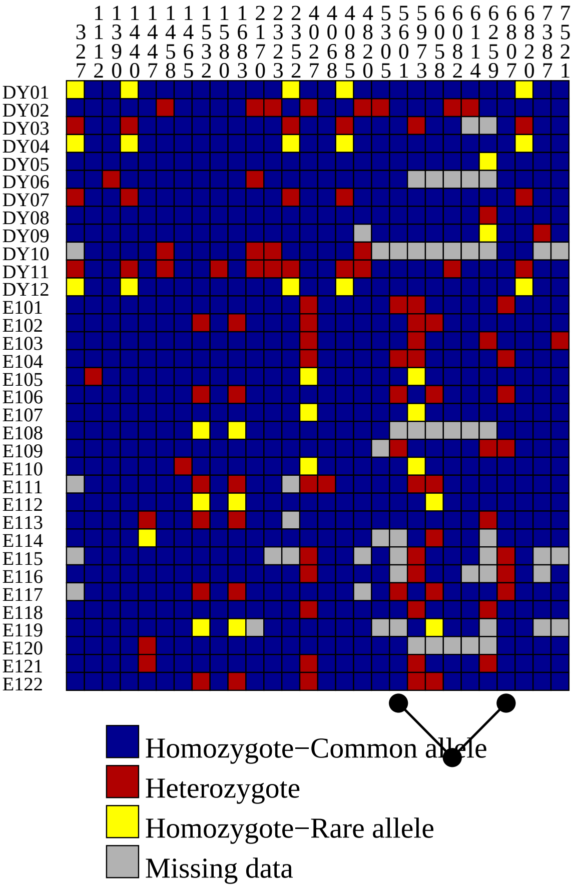

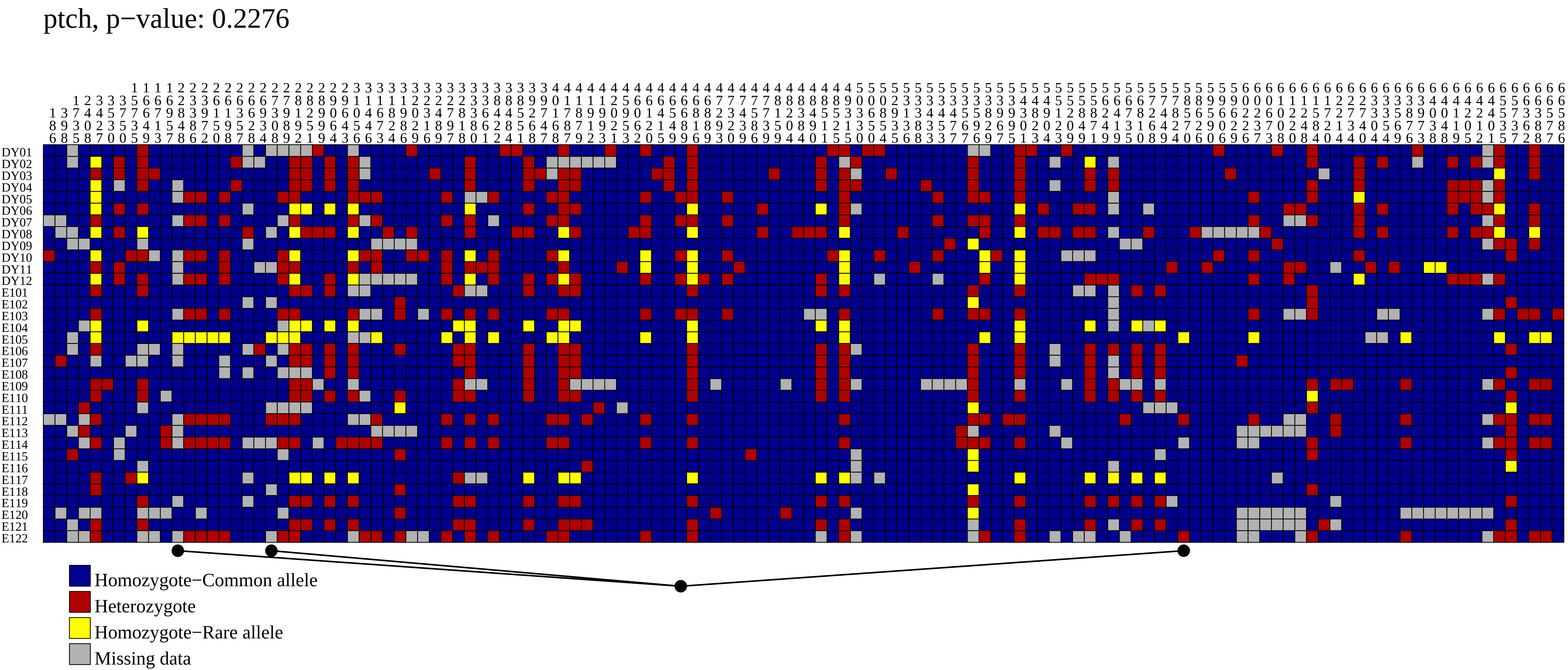
ptch, p−value: 0.2276
DY01
DY02
DY03
DY04
DY05
DY06
DY07
DY08
DY09
DY10
DY11
DY12
E101
E102
E103
E104
E105
E106
E107
E108
E109
E110
E111
E112
E113
E114
E115
E116
E117
E118
E119
E120
E121
E122
Homozygote−Common allele
Heterozygote
Homozygote−Rare allele
Missing data

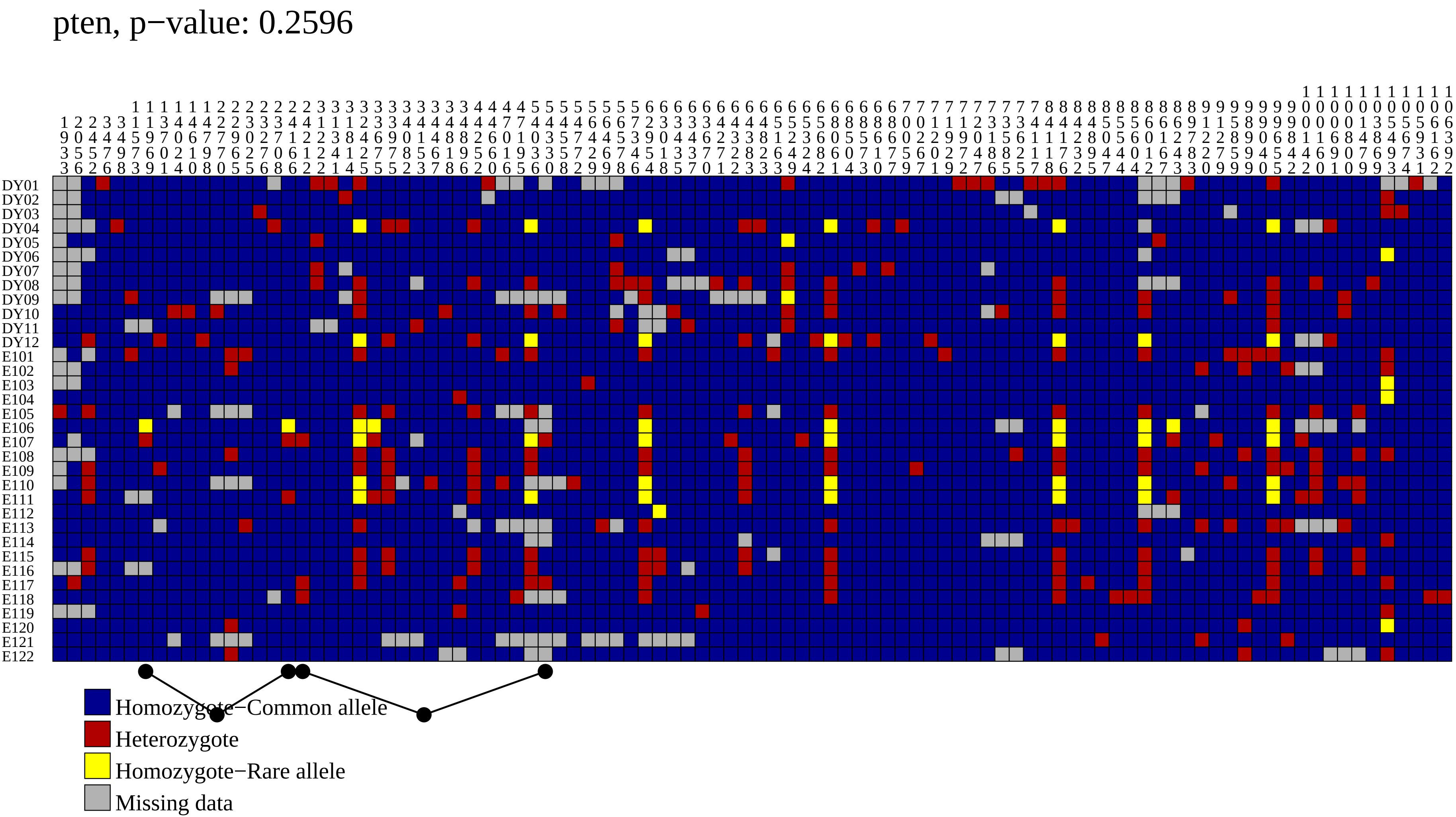
pten, p−value: 0.2596
DY01
DY02
DY03
DY04
DY05
DY06
DY07
DY08
DY09
DY10
DY11
DY12
E101
E102
E103
E104
E105
E106
E107
E108
E109
E110
E111
E112
E113
E114
E115
E116
E117
E118
E119
E120
E121
E122
Homozygote−Common allele
Heterozygote
Homozygote−Rare allele
Missing data

rac1, p−value: 0.8914

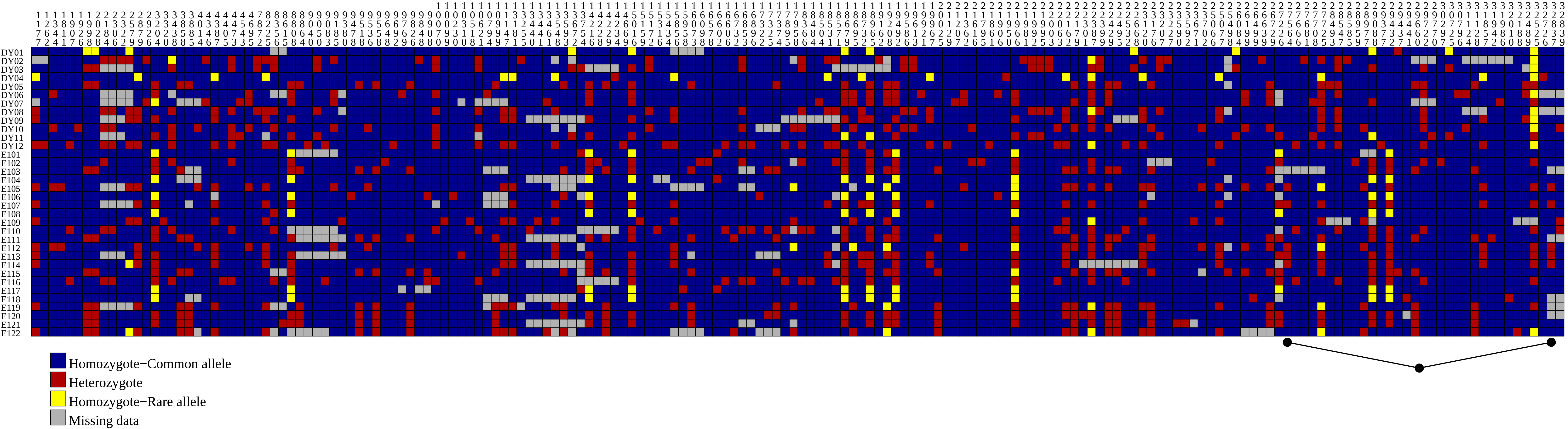

rad18, p−value: 0.0362

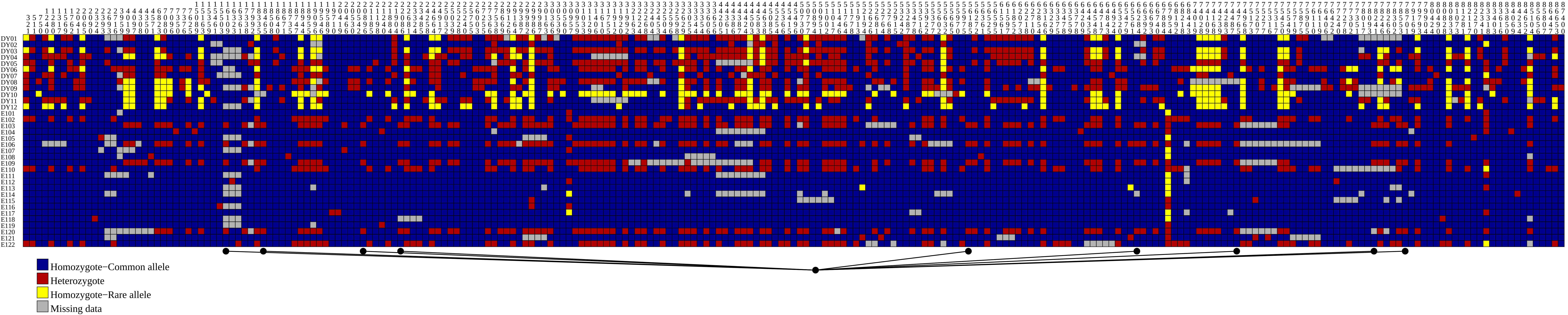

rad51l1, p−value: 0.4884

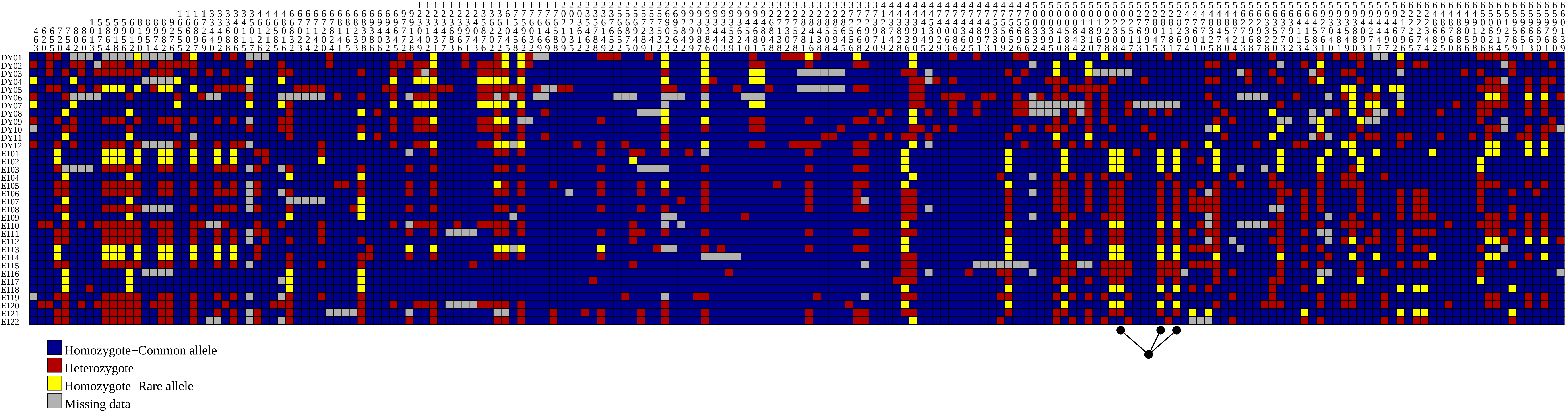

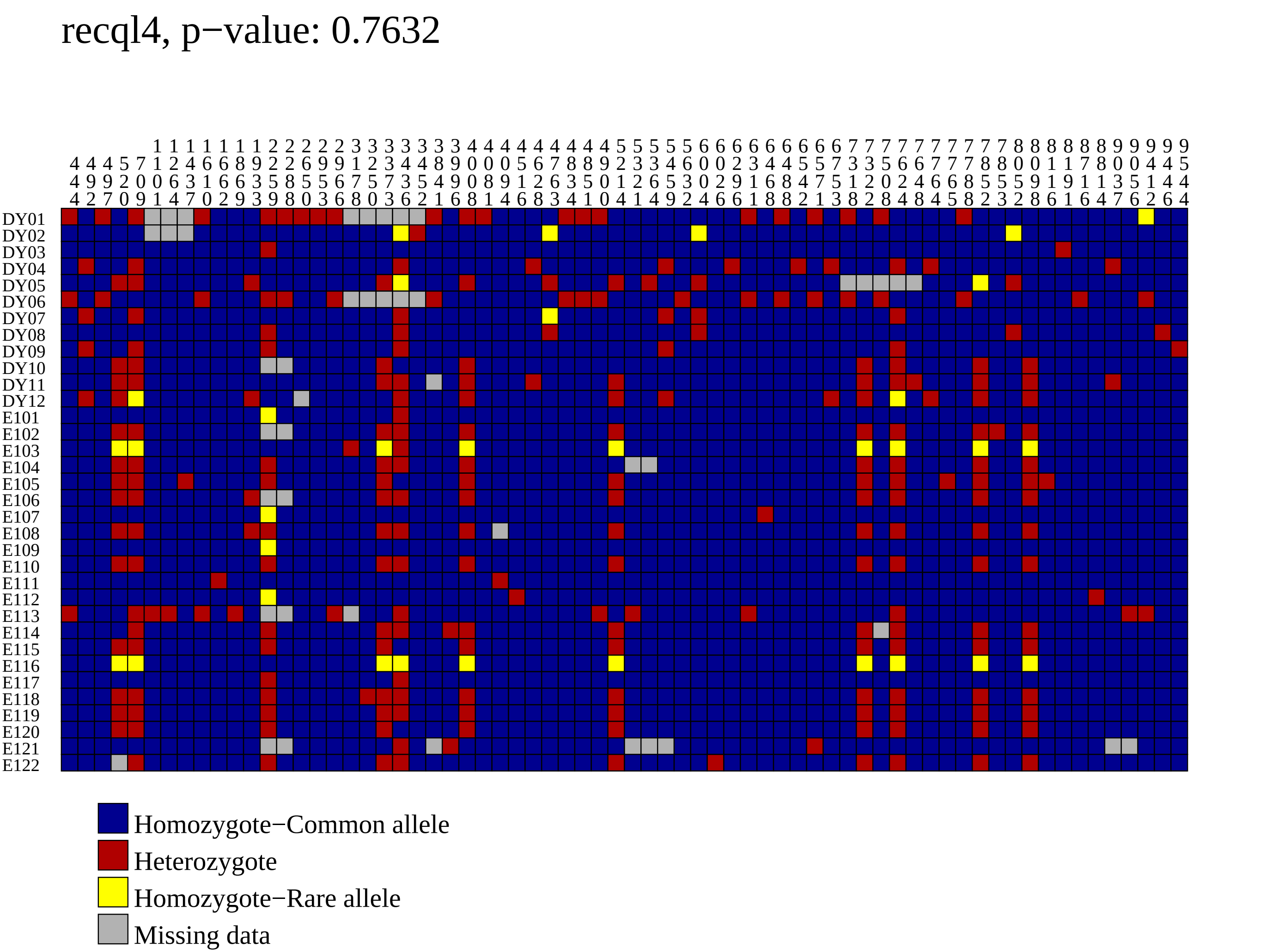
recql4, p−value: 0.7632
Homozygote−Common allele
Heterozygote
Homozygote−Rare allele
Missing data

rev1l, p−value: 0.8328

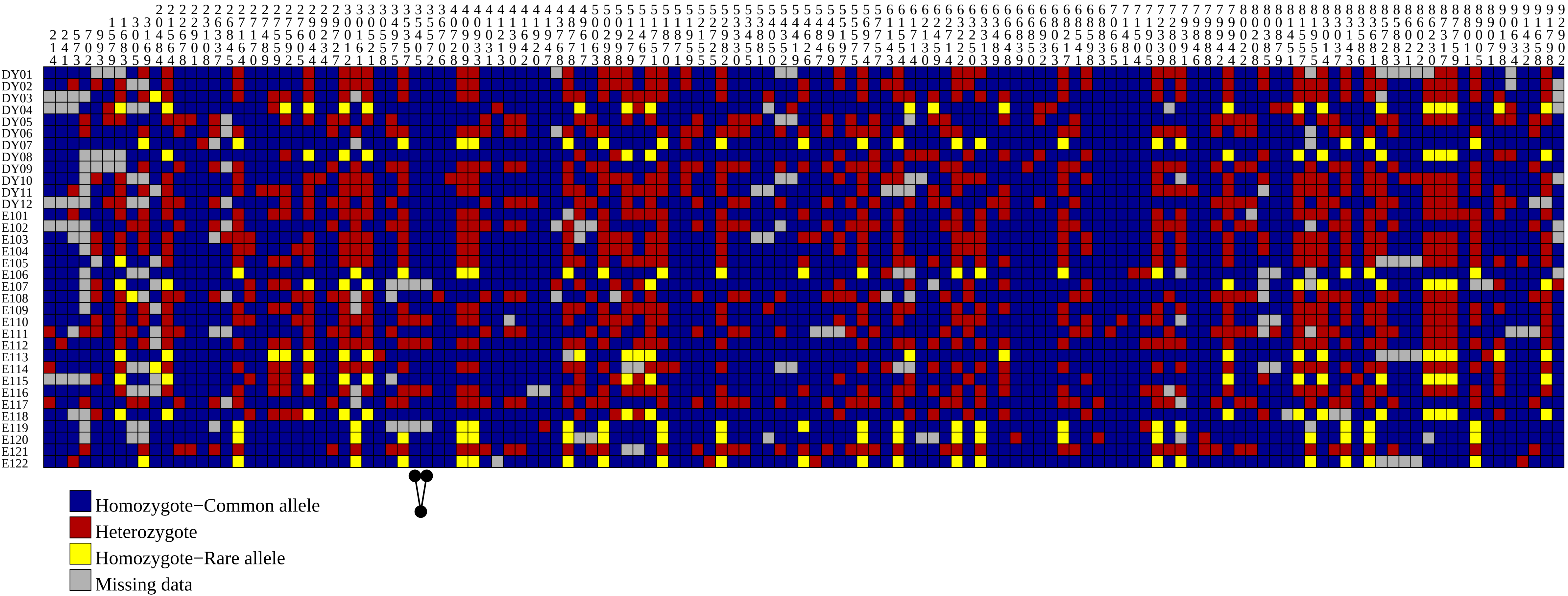

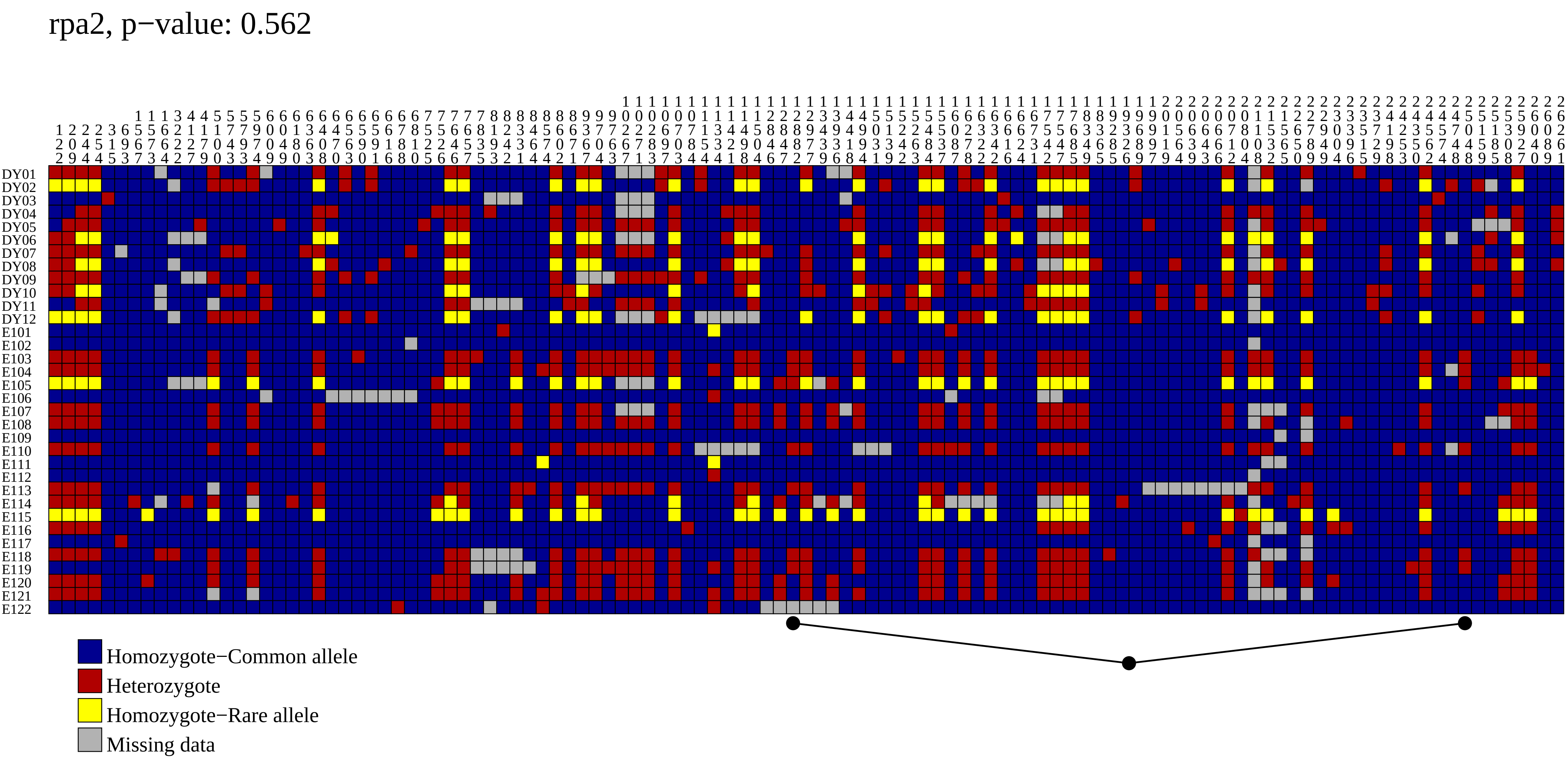
rpa2, p−value: 0.562
DY01
DY02
DY03
DY04
DY05
DY06
DY07
DY08
DY09
DY10
DY11
DY12
E101
E102
E103
E104
E105
E106
E107
E108
E109
E110
E111
E112
E113
E114
E115
E116
E117
E118
E119
E120
E121
E122
Homozygote−Common allele
Heterozygote
Homozygote−Rare allele
Missing data

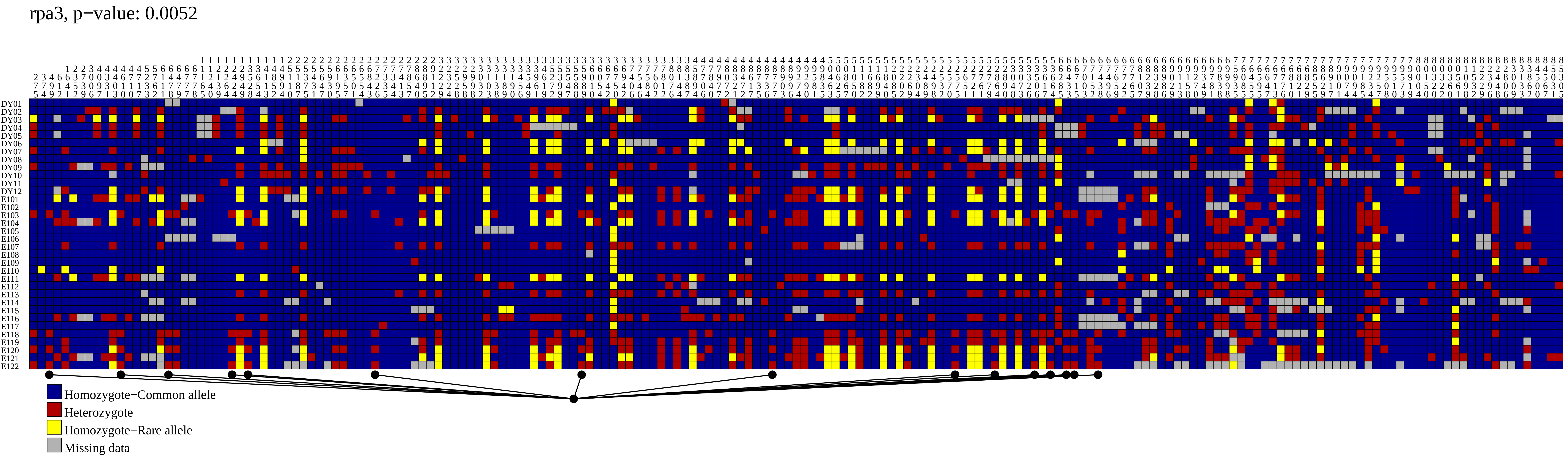
rpa3, p−value: 0.0052
Homozygote−Common allele
Heterozygote
Homozygote−Rare allele
Missing data

rrm2b, p−value: 0.0018

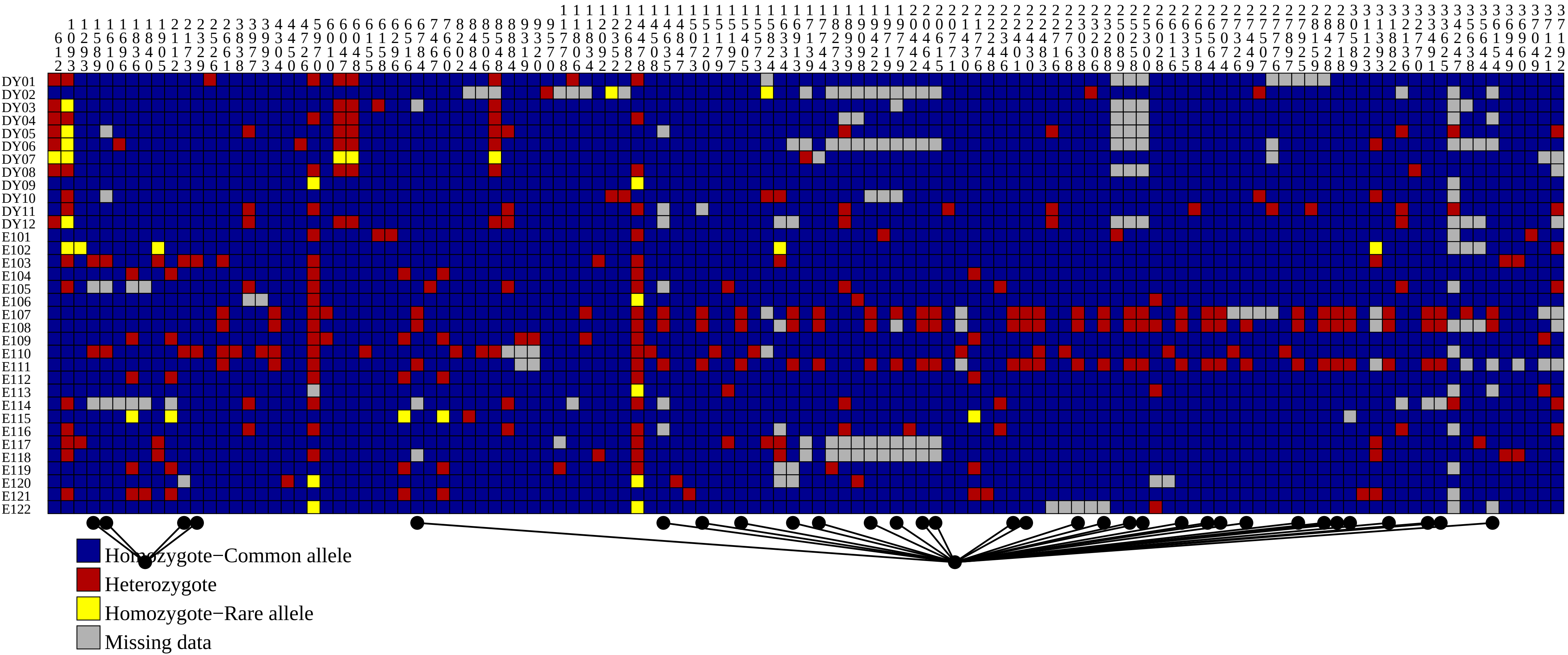

scara3, p−value: 0.0322

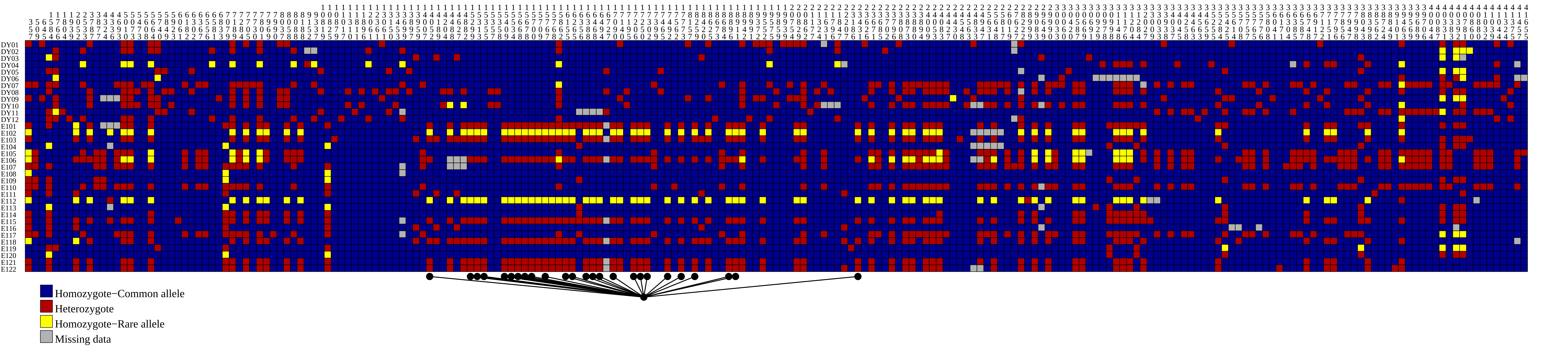

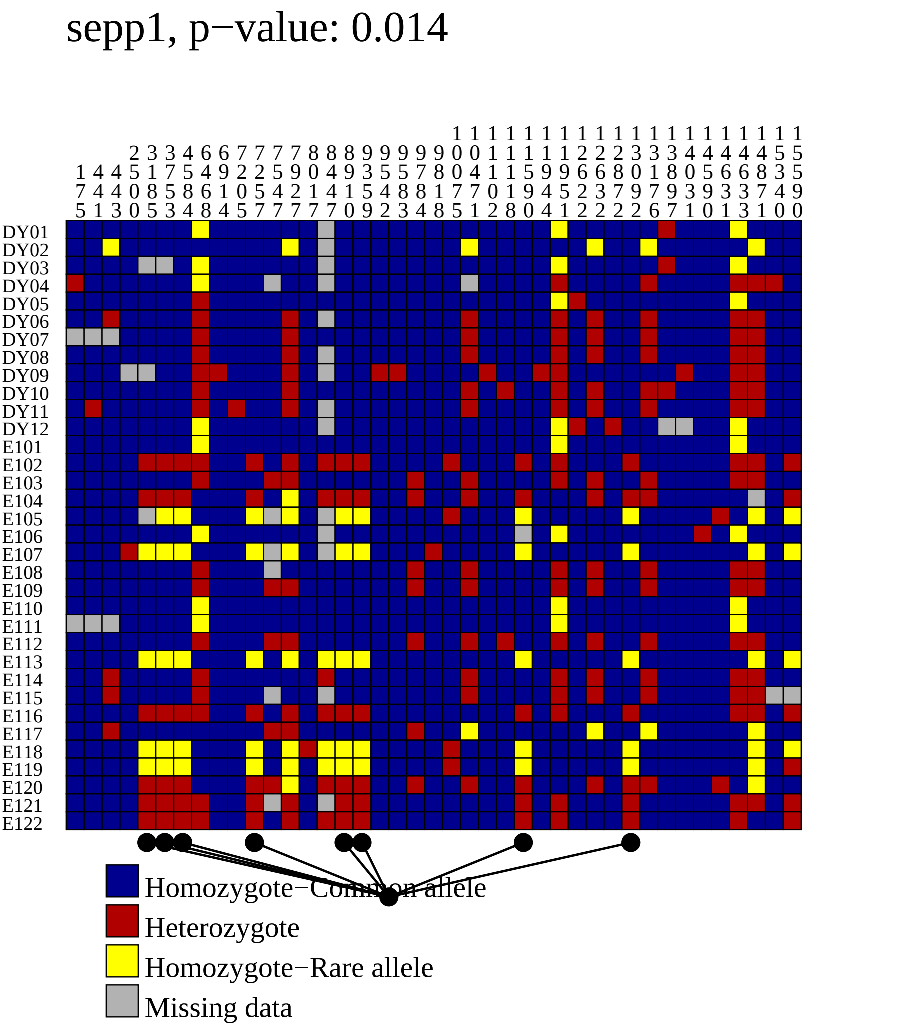
sepp1, p−value: 0.014
175 441 443 250 3185 3753 4584 6468 6914 7205 7257 7547 7927 8017 8447 8910 9359 9542 9583 9784 9818 10075 10471 11102 11118 11590 11944 11951 12622 12632 12872 13092 13176 13897 14031 14590 14631 14633 14871 15340 15590
DY01
DY02
DY03
DY04
DY05
DY06
DY07
DY08
DY09
DY10
DY11
DY12
E101
E102
E103
E104
E105
E106
E107
E108
E109
E110
E111
E112
E113
E114
E115
E116
E117
E118
E119
E120
E121
E122
Homozygote−Common allele
Heterozygote
Homozygote−Rare allele
Missing data

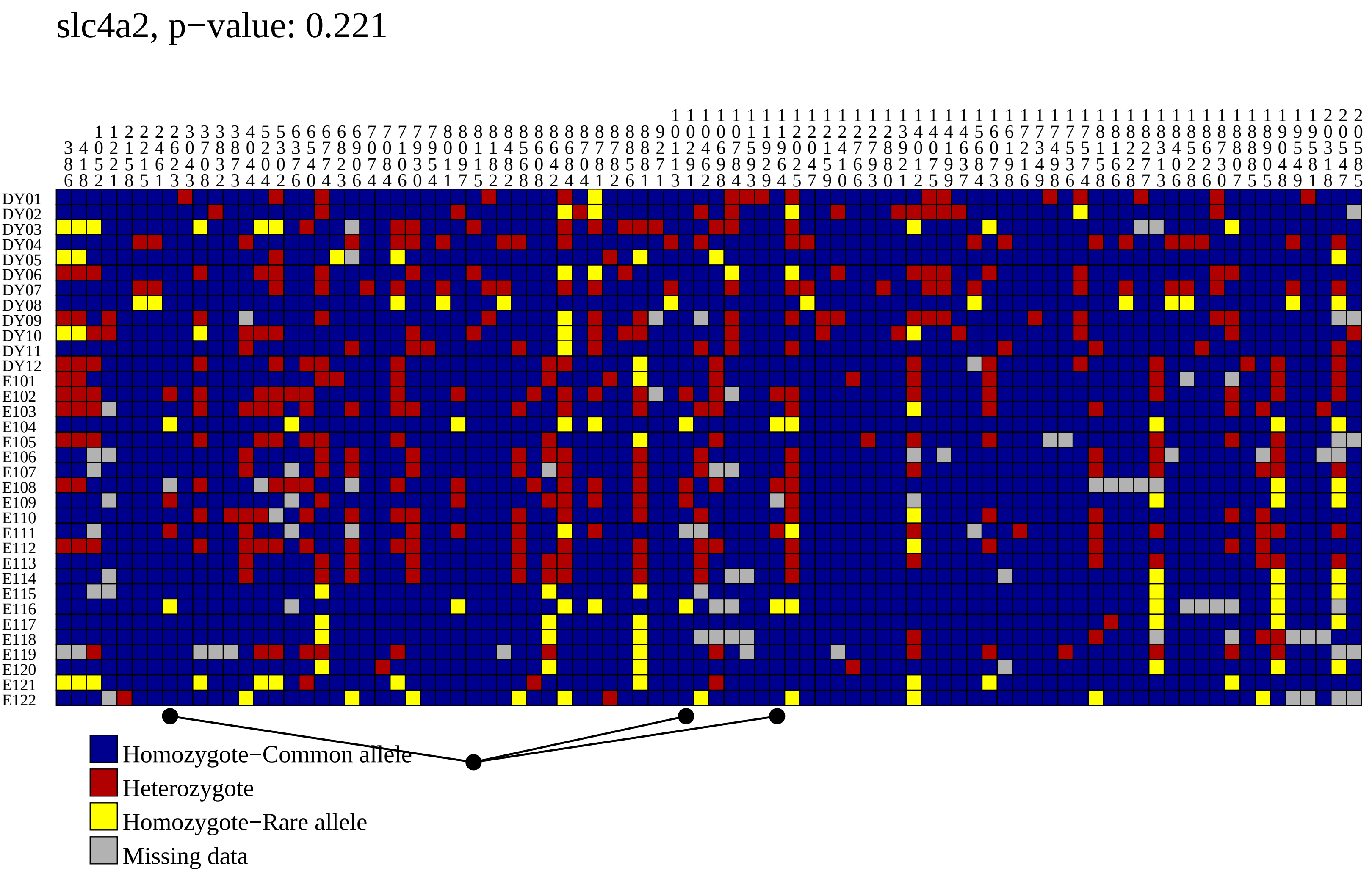
slc4a2, p−value: 0.221
DY01
DY02
DY03
DY04
DY05
DY06
DY07
DY08
DY09
DY10
DY11
DY12
E101
E102
E103
E104
E105
E106
E107
E108
E109
E110
E111
E112
E113
E114
E115
E116
E117
E118
E119
E120
E121
E122
Homozygote−Common allele
Heterozygote
Homozygote−Rare allele
Missing data

smarcb1, p−value: 0.5342

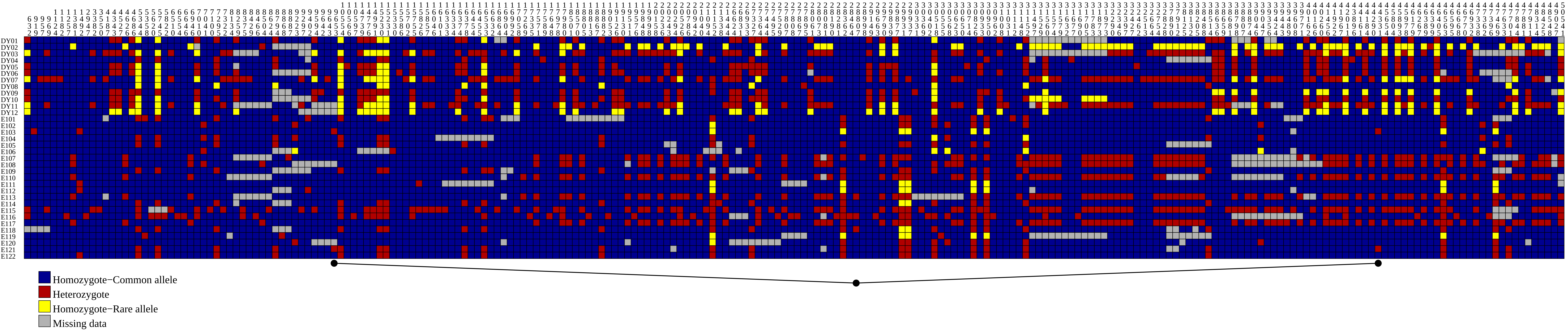

snca, p−value: 0.9632

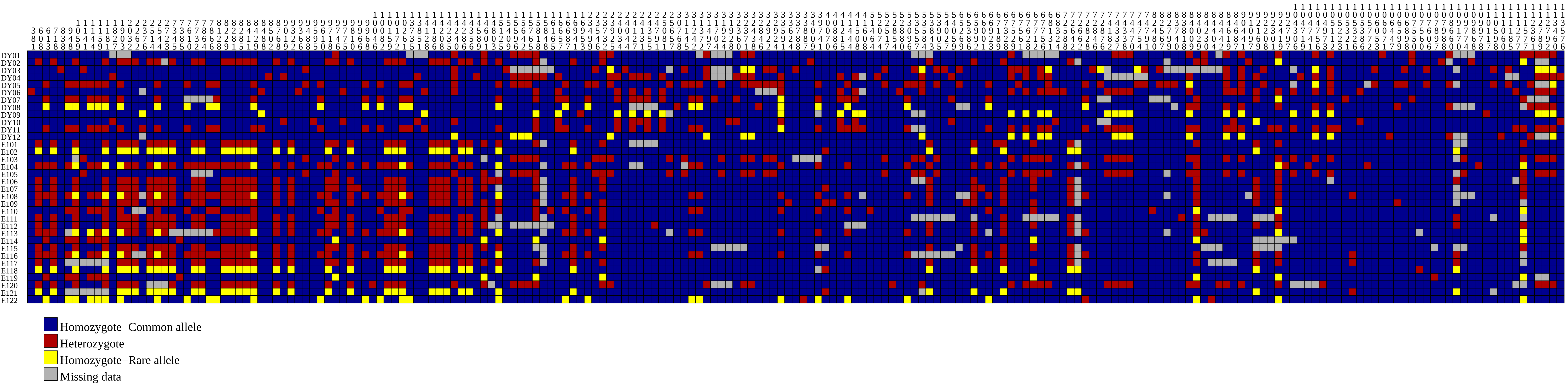

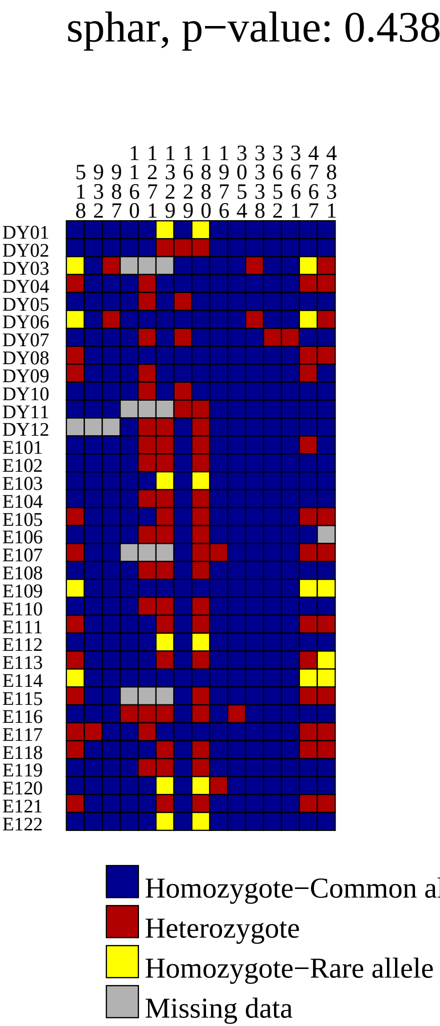
sphar, p−value: 0.438
518
932
987
1160
1271
1329
1629
1880
1976
3054
3338
3652
3661
4767
4831
DY01
DY02
DY03
DY04
DY05
DY06
DY07
DY08
DY09
DY10
DY11
DY12
E101
E102
E103
E104
E105
E106
E107
E108
E109
E110
E111
E112
E113
E114
E115
E116
E117
E118
E119
E120
E121
E122
Homozygote−Common al
Heterozygote
Homozygote−Rare allele
Missing data

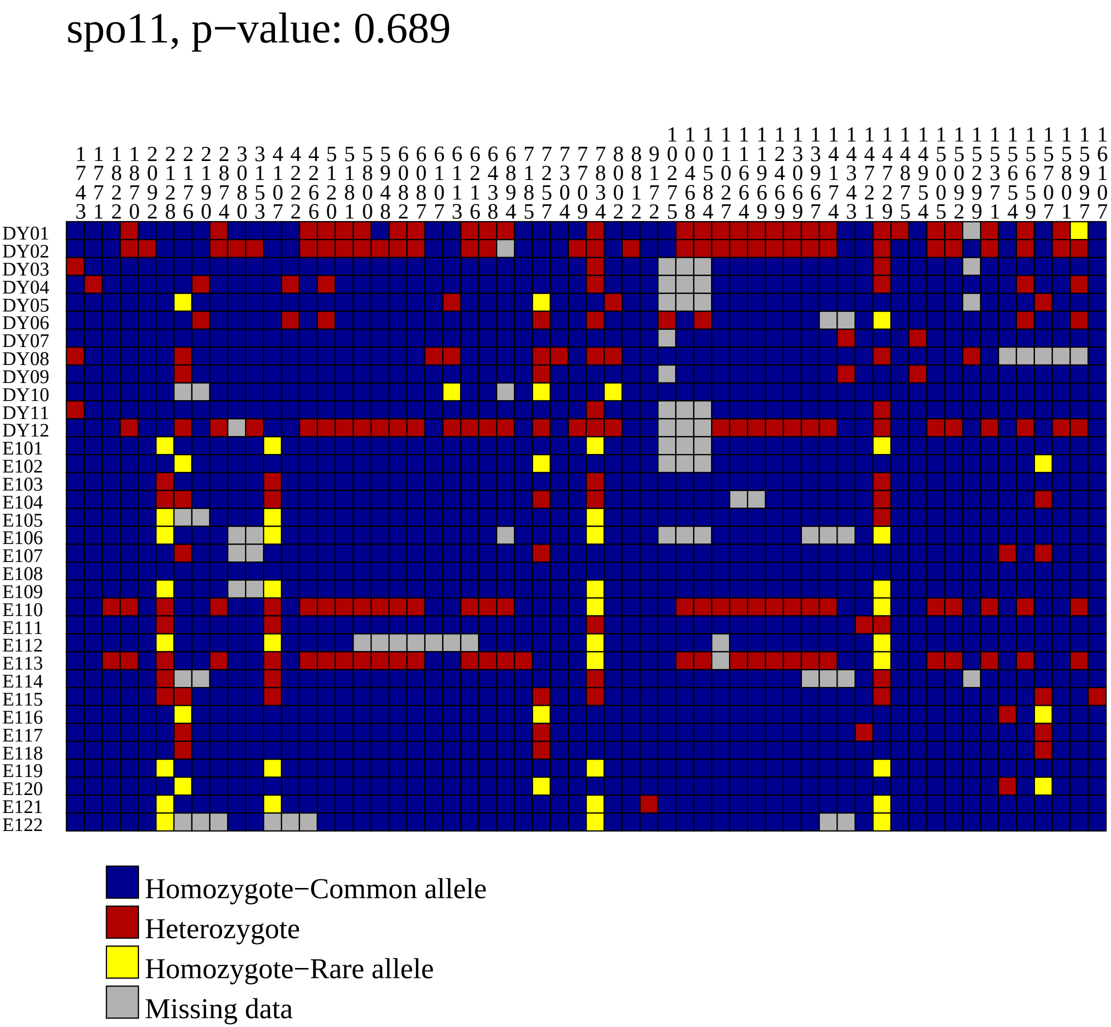
spo11, p−value: 0.689
1743 1771 1822 1870 2092 2128 2176 2190 2874 3080 3153 4107 4222 4266 5120 5181 5800 5948 6082 6087 6107 6113 6216 6438 6894 7185 7257 7304 7709 7834 8002 8812 9172 10275 10468 10584 11027 11664 11969 12469 13069 13967 14174 14743 14721 14729 14875 14954 15005 15092 15299 15371 15654 15659 15707 15801 15917 16107
DY01
DY02
DY03
DY04
DY05
DY06
DY07
DY08
DY09
DY10
DY11
DY12
E101
E102
E103
E104
E105
E106
E107
E108
E109
E110
E111
E112
E113
E114
E115
E116
E117
E118
E119
E120
E121
E122
Homozygote−Common allele
Heterozygote
Homozygote−Rare allele
Missing data

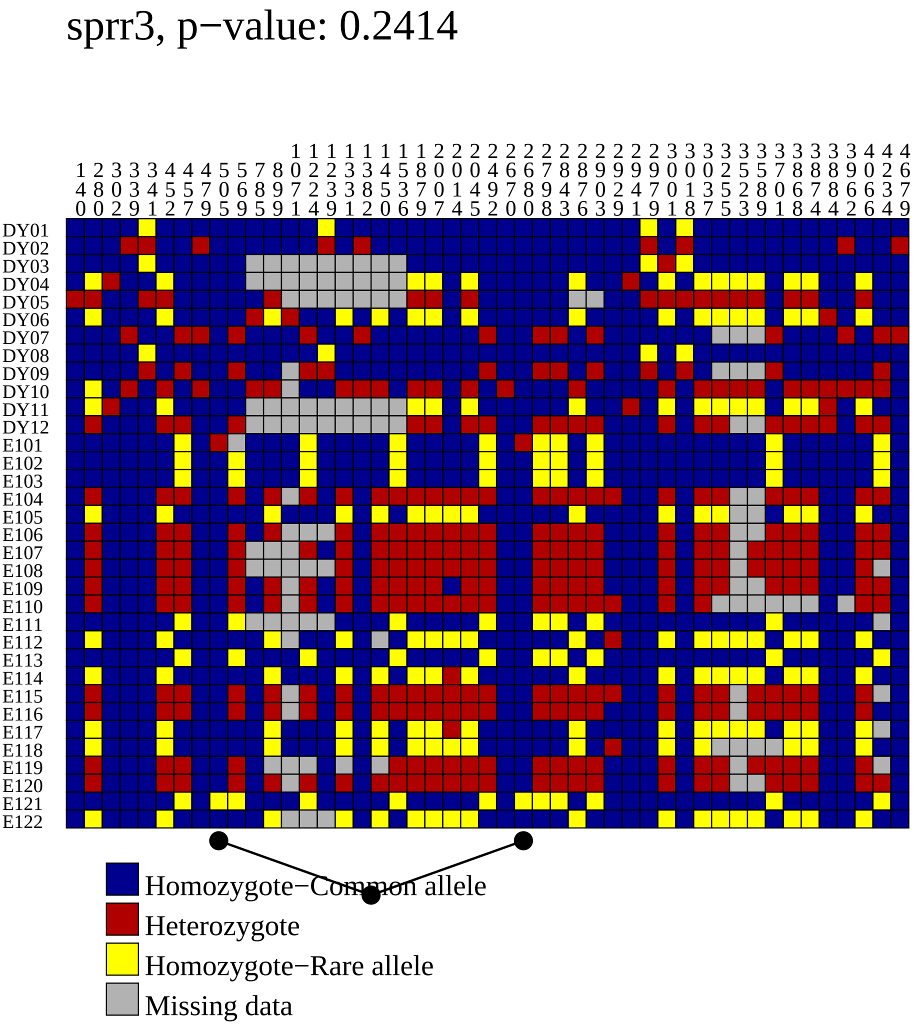
sprr3, p−value: 0.2414
140 280 302 339 341 452 457 479 505 569 785 899 1071 1224 1239 1331 1382 1450 1536 1879 2007 2014 2045 2492 2670 2680 2798 2843 2876 2903 2929 2941 2979 3001 3018 3037 3253 3523 3589 3701 3868 3874 3884 3962 4026 4234 4679
DY01 DY02 DY03 DY04 DY05 DY06 DY07 DY08 DY09 DY10 DY11 DY12
E101 E102 E103 E104 E105 E106 E107 E108 E109 E110 E111 E112 E113 E114 E115 E116 E117 E118 E119 E120 E121 E122
Homozygote−Common allele
Heterozygote
Homozygote−Rare allele
Missing data

srd5a2, p−value: 0.3952

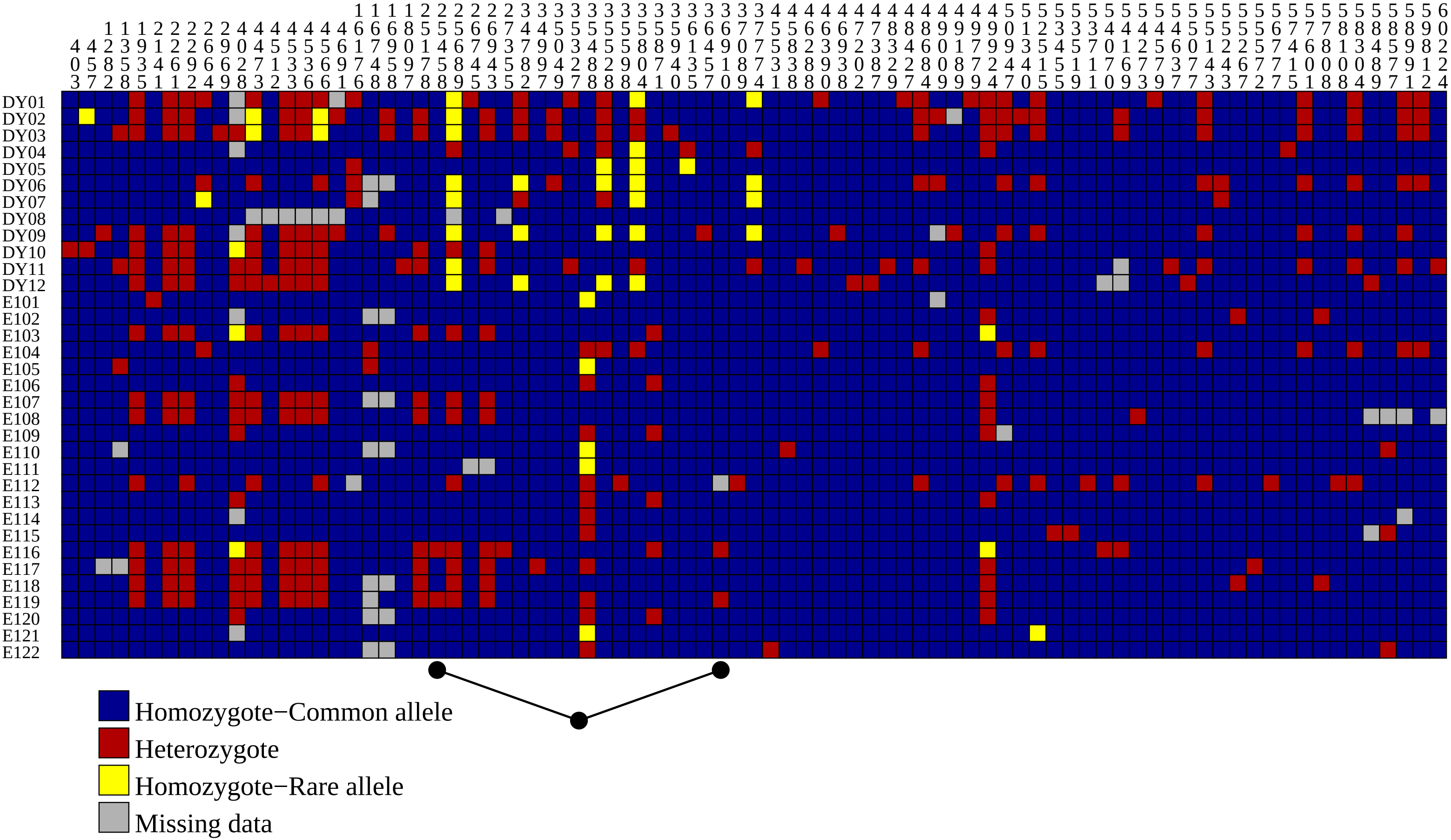

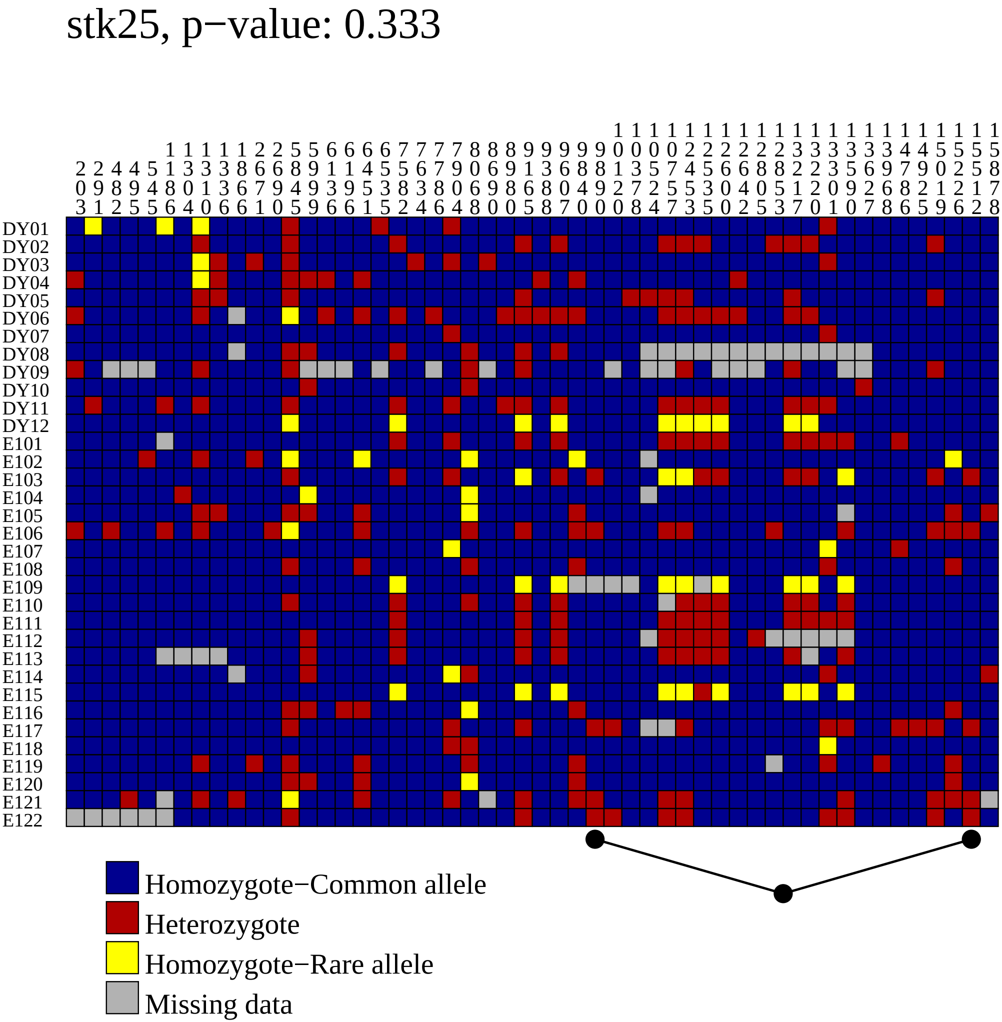
stk25, p−value: 0.333
203 291 482 495 545 1186 1304 1310 1336 1866 2671 2690 5845 5999 6136 6196 6451 6535 7582 7634 7786 7904 8068 8690 8980 9165 9388 9607 9840 9890 10120 10378 10524 10757 12453 12535 12600 12642 12805 12853 13217 13220 13301 13590 13627 13968 14786 14925 15019 15226 15512 15878
DY01
DY02
DY03
DY04
DY05
DY06
DY07
DY08
DY09
DY10
DY11
DY12
E101
E102
E103
E104
E105
E106
E107
E108
E109
E110
E111
E112
E113
E114
E115
E116
E117
E118
E119
E120
E121
E122
Homozygote−Common allele
Heterozygote
Homozygote−Rare allele
Missing data

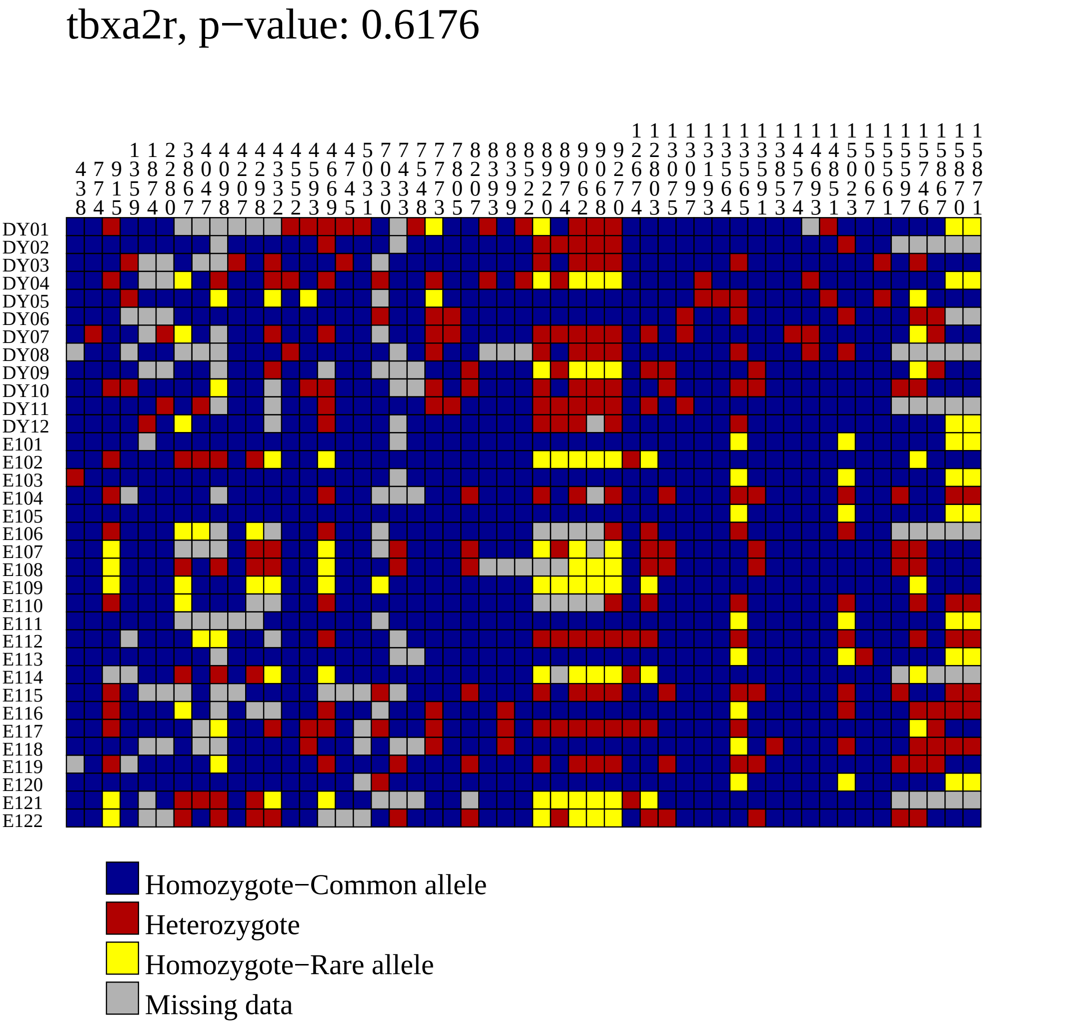

tbxa2r, p−value: 0.6176
438
774
915
1359
1874
2280
3867
4047
4098
4207
4298
4332
4552
4593
4669
4745
5031
7030
7433
7548
7773
7805
8207
8393
8399
8522
8920
8974
9062
9068
9270
12674
12803
13075
13097
13193
13564
13565
13591
13853
14574
14693
14851
15023
15067
15061
15597
15746
15867
15870
15871
DY01
DY02
DY03
DY04
DY05
DY06
DY07
DY08
DY09
DY10
DY11
DY12
E101
E102
E103
E104
E105
E106
E107
E108
E109
E110
E111
E112
E113
E114
E115
E116
E117
E118
E119
E120
E121
E122
Homozygote−Common allele
Heterozygote
Homozygote−Rare allele
Missing data

tert, p−value: 0.8844

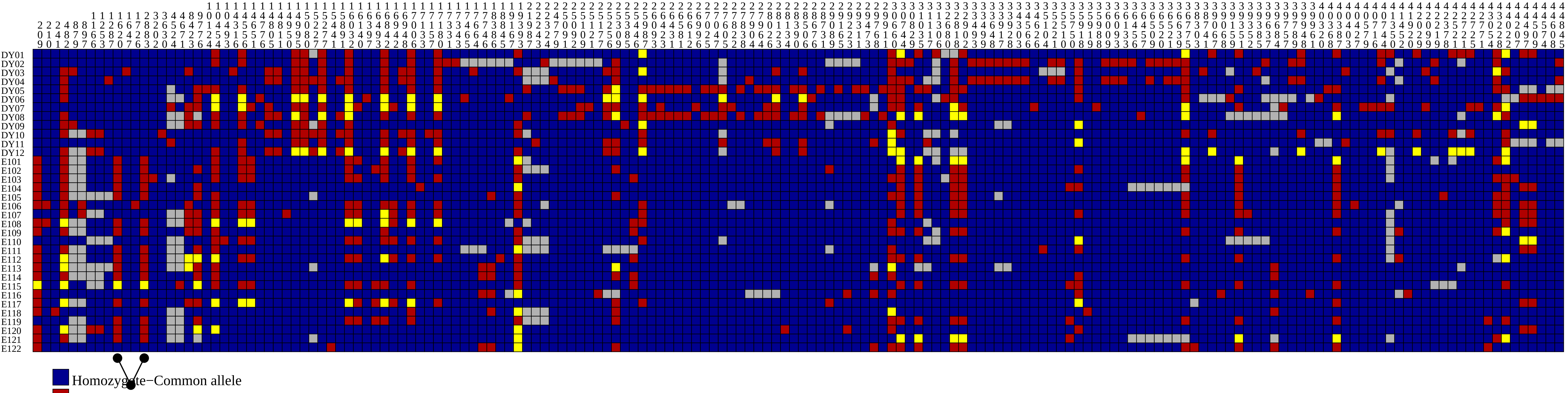

Homozygote−Common allele
Heterozygote
Homozygote−Rare allele
Missing data

tjp1, p−value: 0.3208

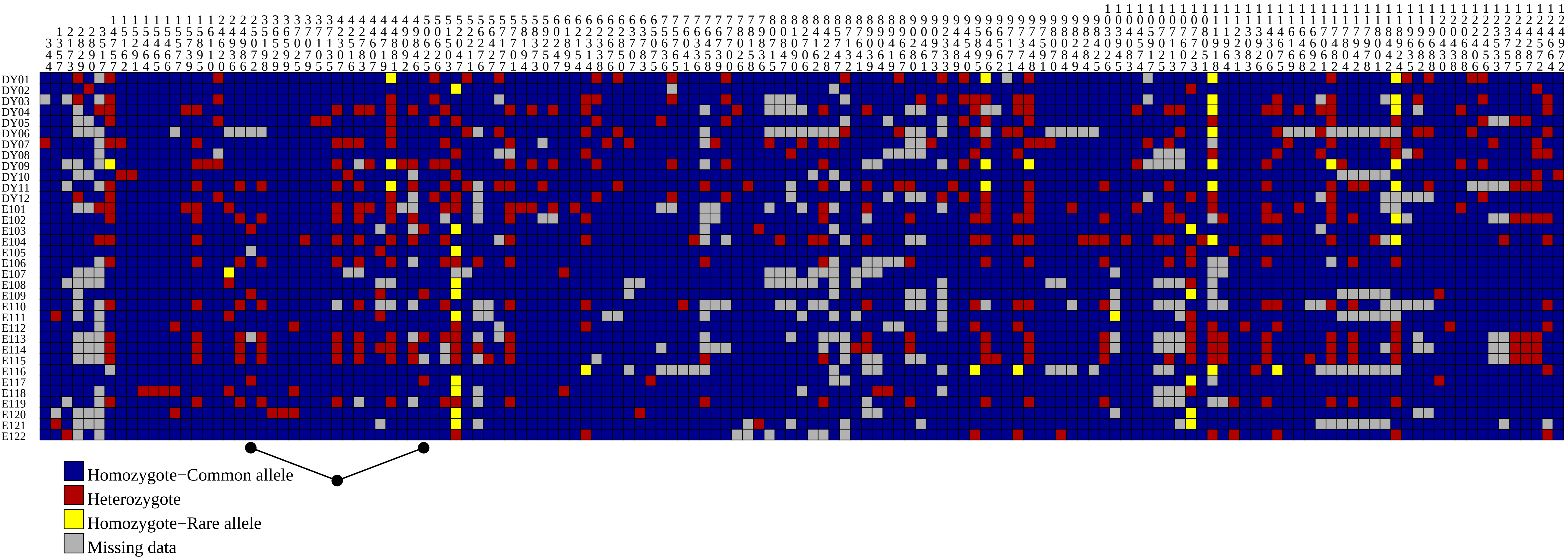

tnfrsf4, p−value: 0.5824

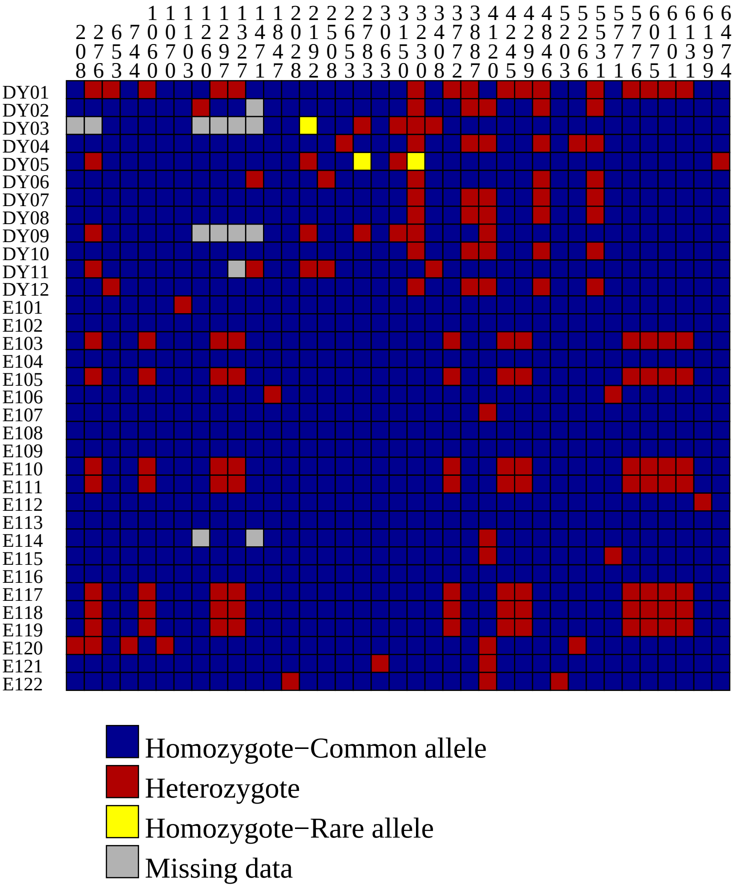

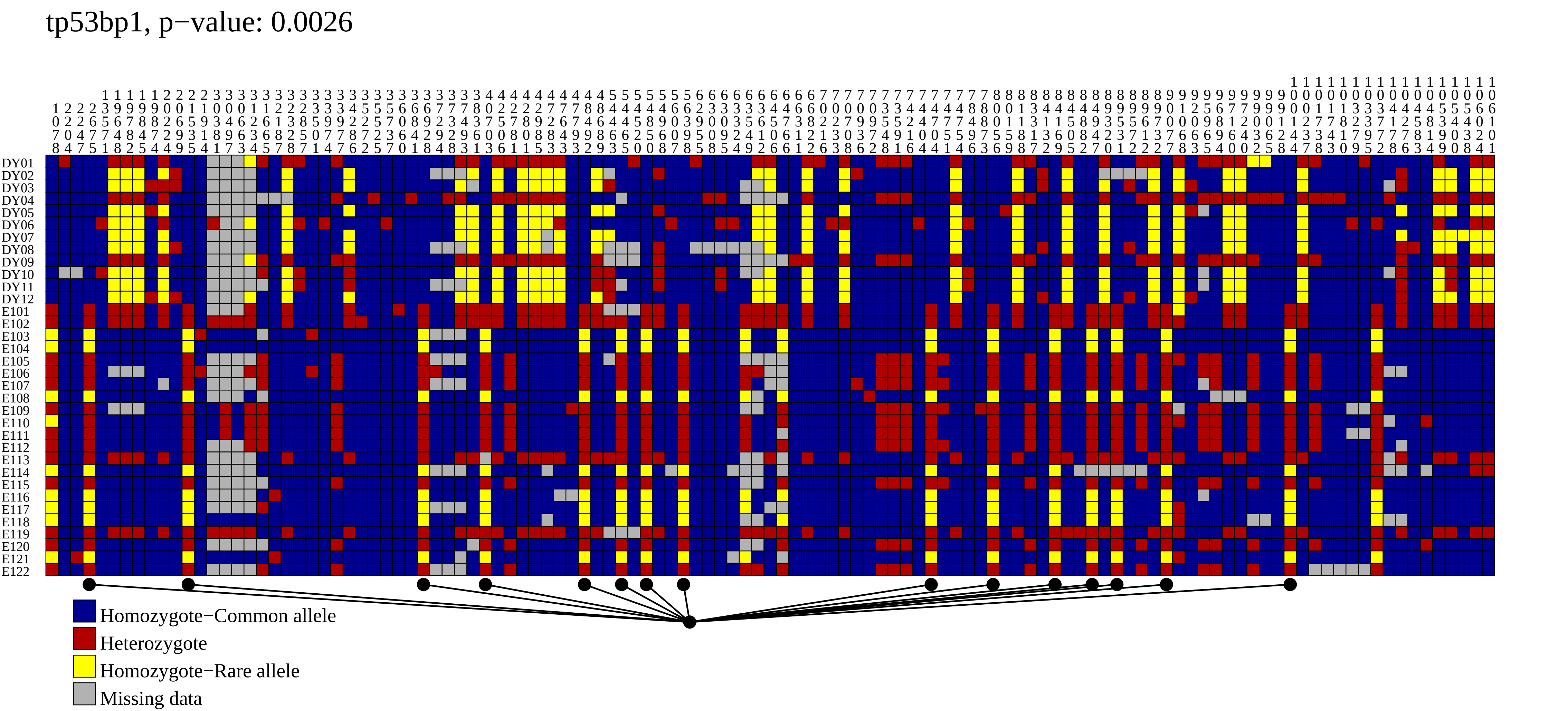
tp53bp1, p−value: 0.0026
DY01
DY02
DY03
DY04
DY05
DY06
DY07
DY08
DY09
DY10
DY11
DY12
E101
E102
E103
E104
E105
E106
E107
E108
E109
E110
E111
E112
E113
E114
E115
E116
E117
E118
E119
E120
E121
E122
Homozygote−Common allele
Heterozygote
Homozygote−Rare allele
Missing data

tp53i3, p−value: 0.0132

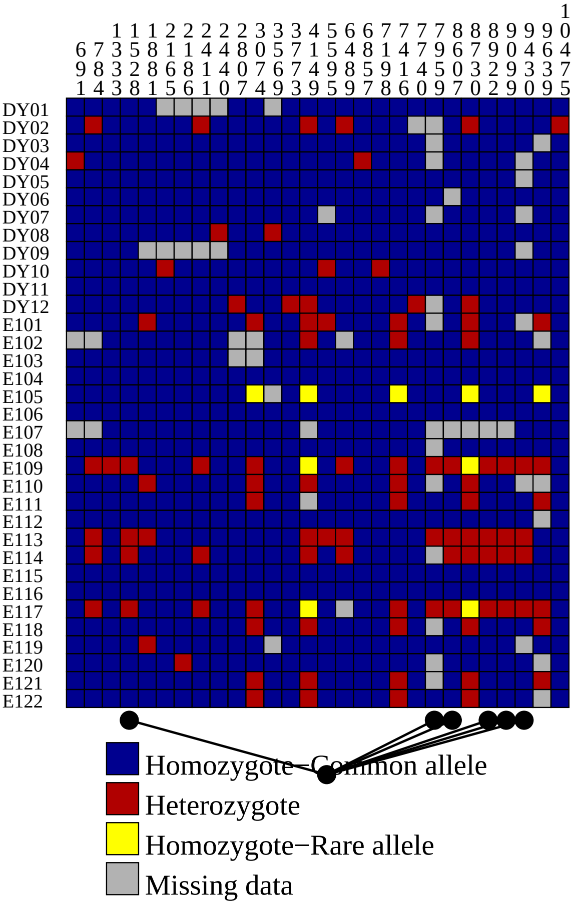

tpo, p−value: 0.1076

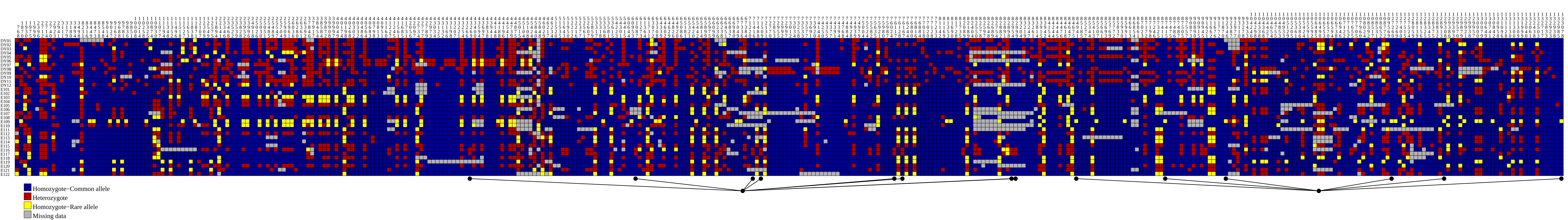

trpm2, p−value: 0.9546

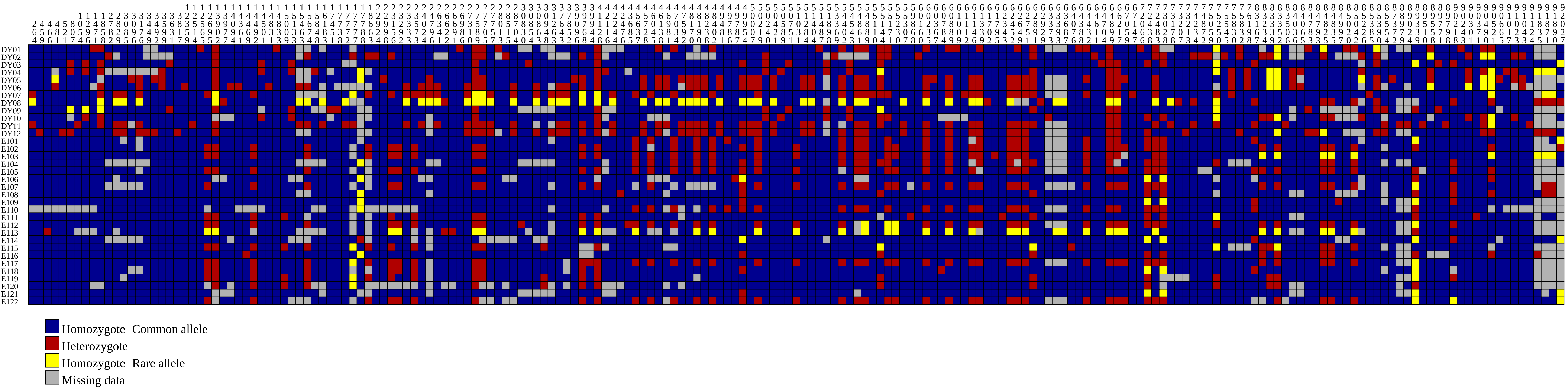

tuba1, p−value: 0.4312

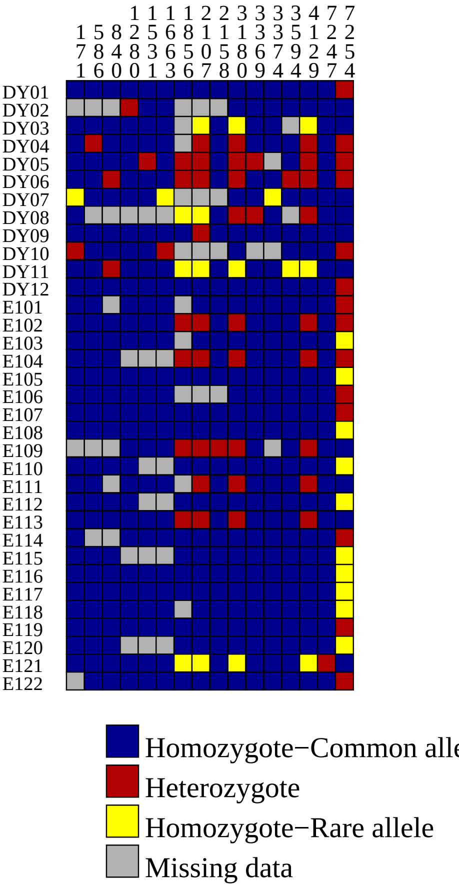

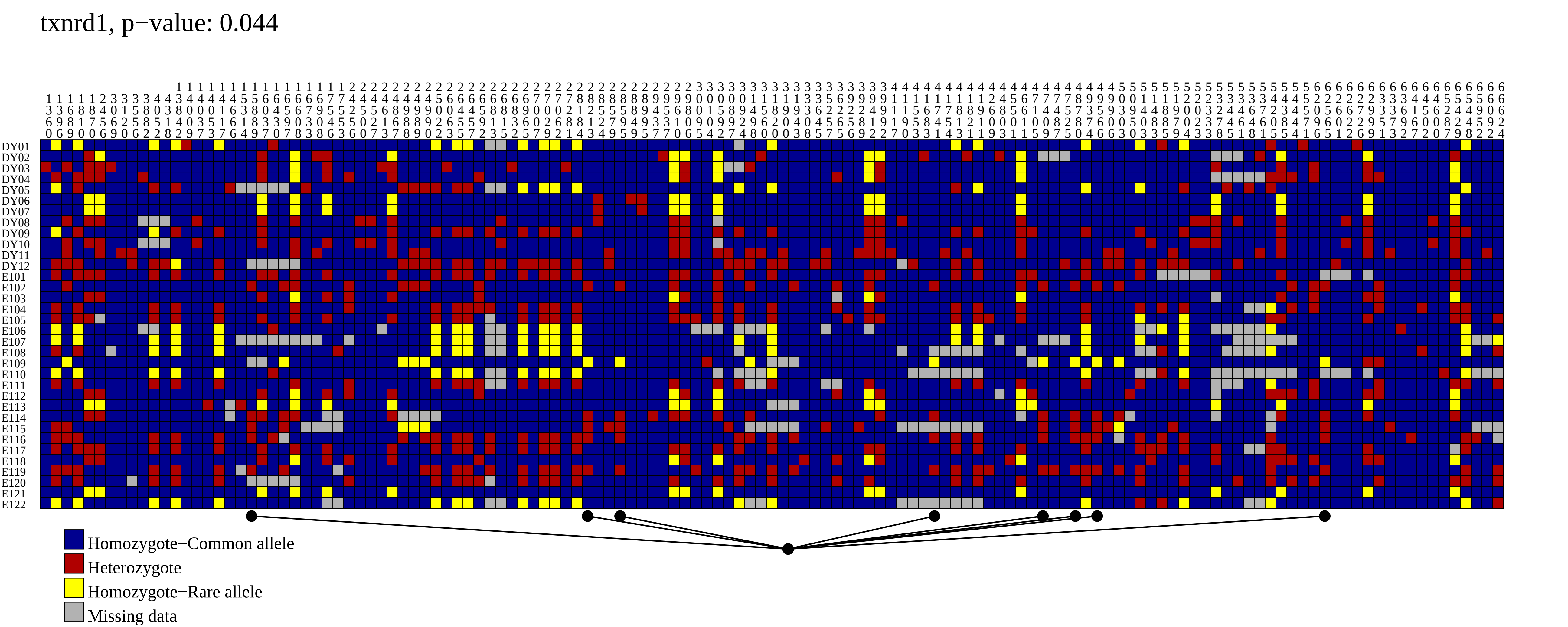
txnrd1, p−value: 0.044
DY01
DY02
DY03
DY04
DY05
DY06
DY07
DY08
DY09
DY10
DY11
DY12
E101
E102
E103
E104
E105
E106
E107
E108
E109
E110
E111
E112
E113
E114
E115
E116
E117
E118
E119
E120
E121
E122
Homozygote−Common allele
Heterozygote
Homozygote−Rare allele
Missing data

ube2b, p−value: 0.6018

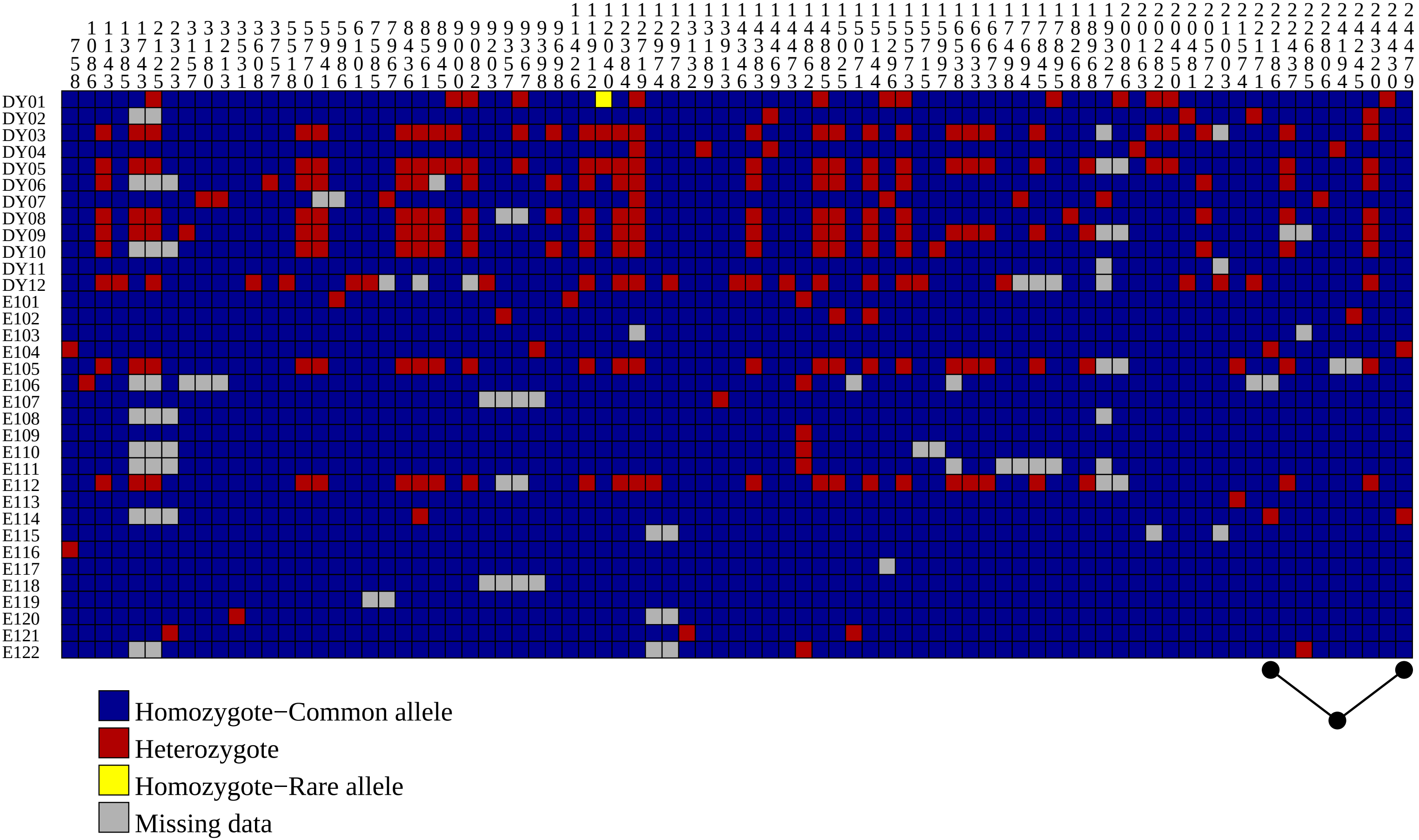

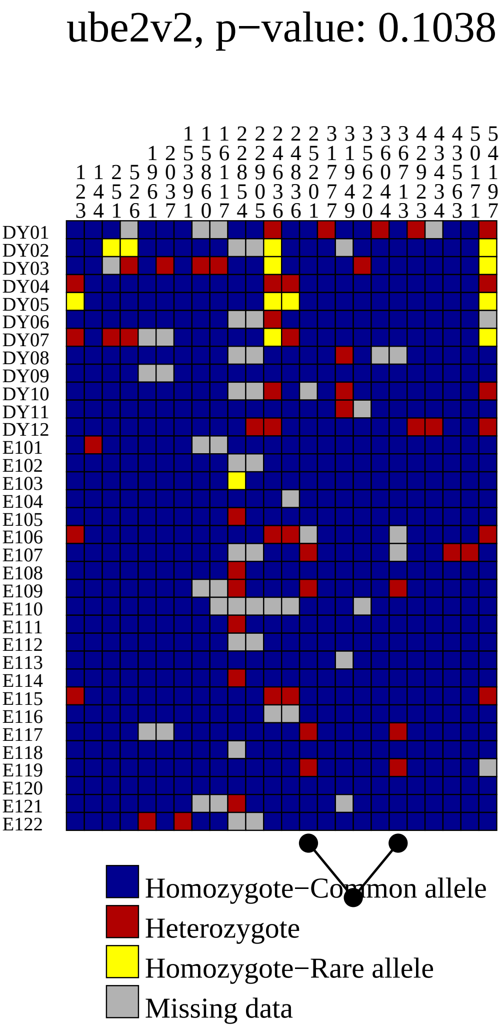
ube2v2, p−value: 0.1038
123 144 251 526 1961 2037 15391 15860 16117 22854 22905 24636 24836 25201 31777 31949 35620 36044 36713 42923 43434 43563 50171 54197
DY01
DY02
DY03
DY04
DY05
DY06
DY07
DY08
DY09
DY10
DY11
DY12
E101
E102
E103
E104
E105
E106
E107
E108
E109
E110
E111
E112
E113
E114
E115
E116
E117
E118
E119
E120
E121
E122
Homozygote−Common allele
Heterozygote
Homozygote−Rare allele
Missing data

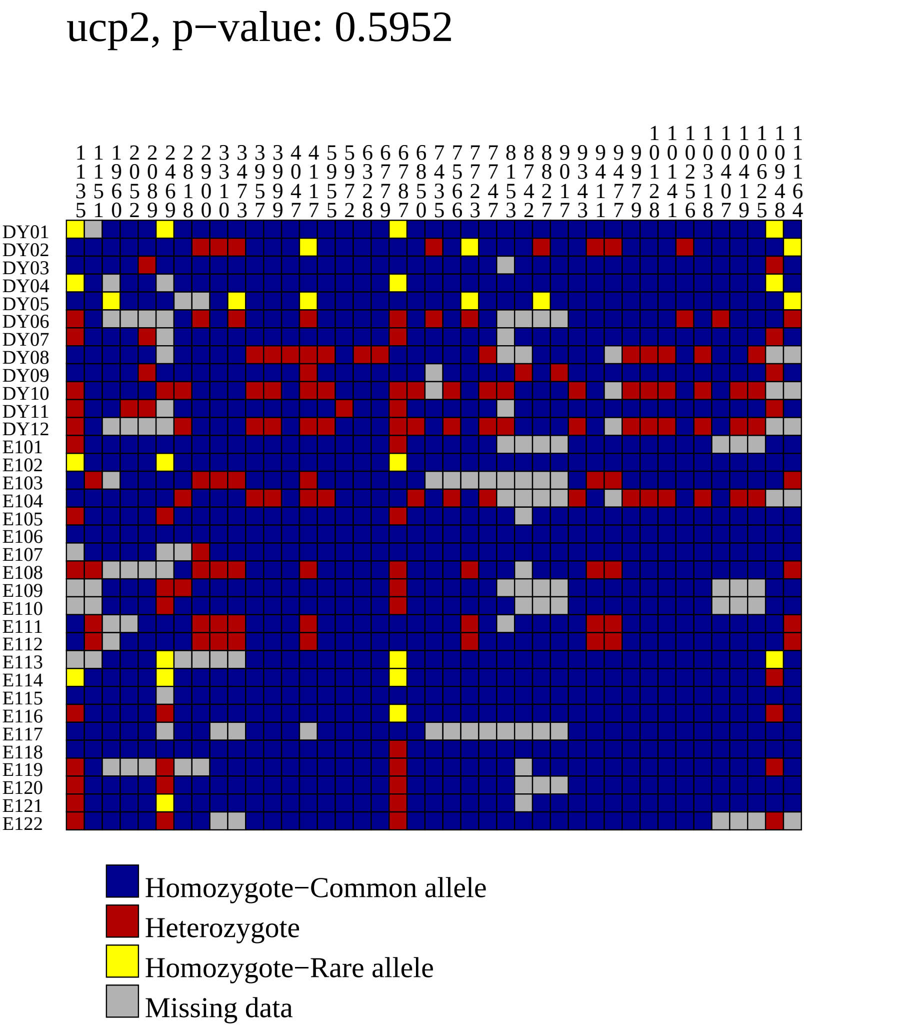
ucp2, p−value: 0.5952
1135
1151
1960
2052
2089
2469
2818
2900
3310
3473
3957
3999
4047
4117
5955
5972
6328
6779
6787
6850
7435
7566
7723
7747
8153
8742
8827
9017
9343
9411
9477
9979
10128
10141
10256
10318
10407
10419
10625
10948
11164
DY01
DY02
DY03
DY04
DY05
DY06
DY07
DY08
DY09
DY10
DY11
DY12
E101
E102
E103
E104
E105
E106
E107
E108
E109
E110
E111
E112
E113
E114
E115
E116
E117
E118
E119
E120
E121
E122
Homozygote−Common allele
Heterozygote
Homozygote−Rare allele
Missing data

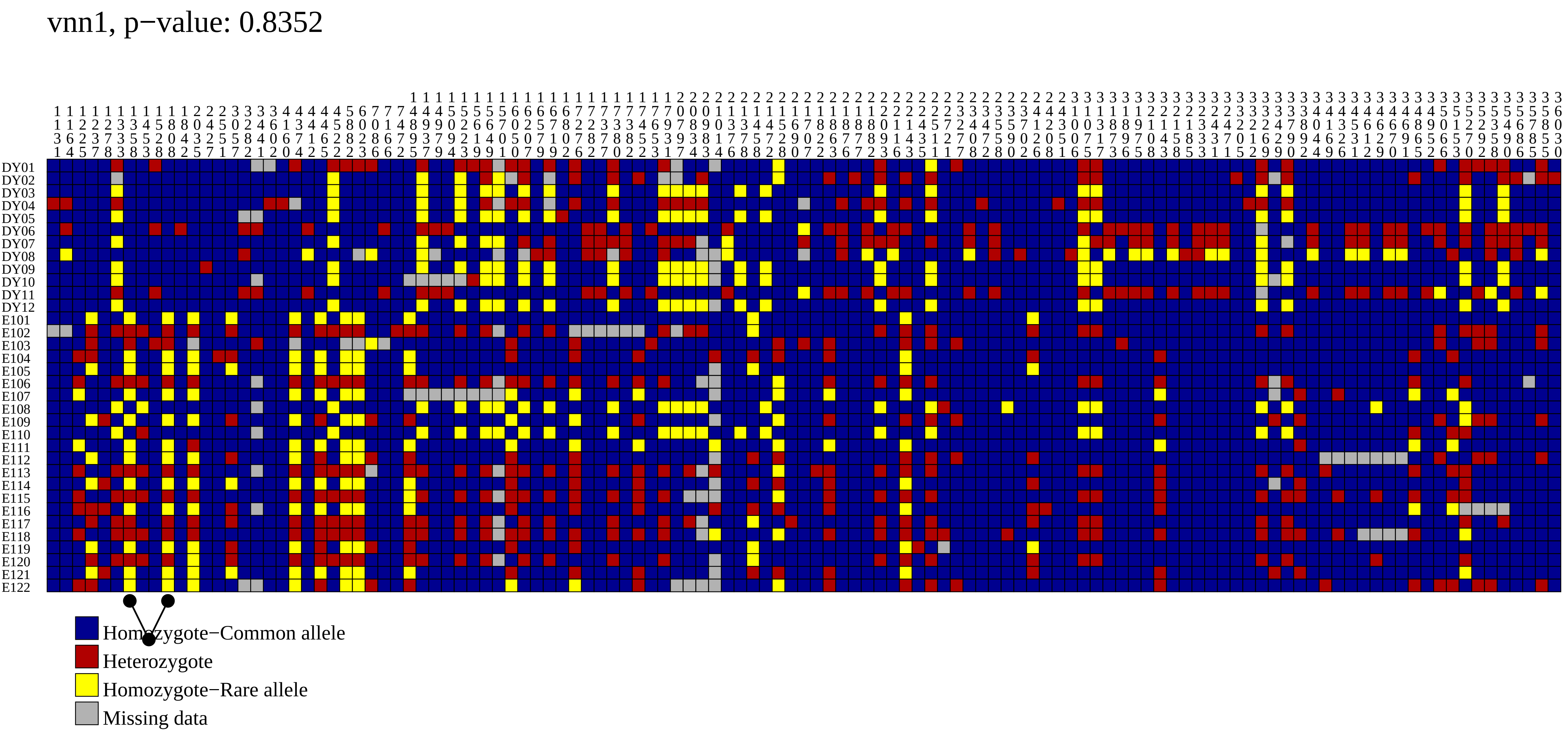
vnn1, p−value: 0.8352
DY01
DY02
DY03
DY04
DY05
DY06
DY07
DY08
DY09
DY10
DY11
DY12
E101
E102
E103
E104
E105
E106
E107
E108
E109
E110
E111
E112
E113
E114
E115
E116
E117
E118
E119
E120
E121
E122
Homozygote−Common allele
Heterozygote
Homozygote−Rare allele
Missing data

vnn2, p−value: 0.0162

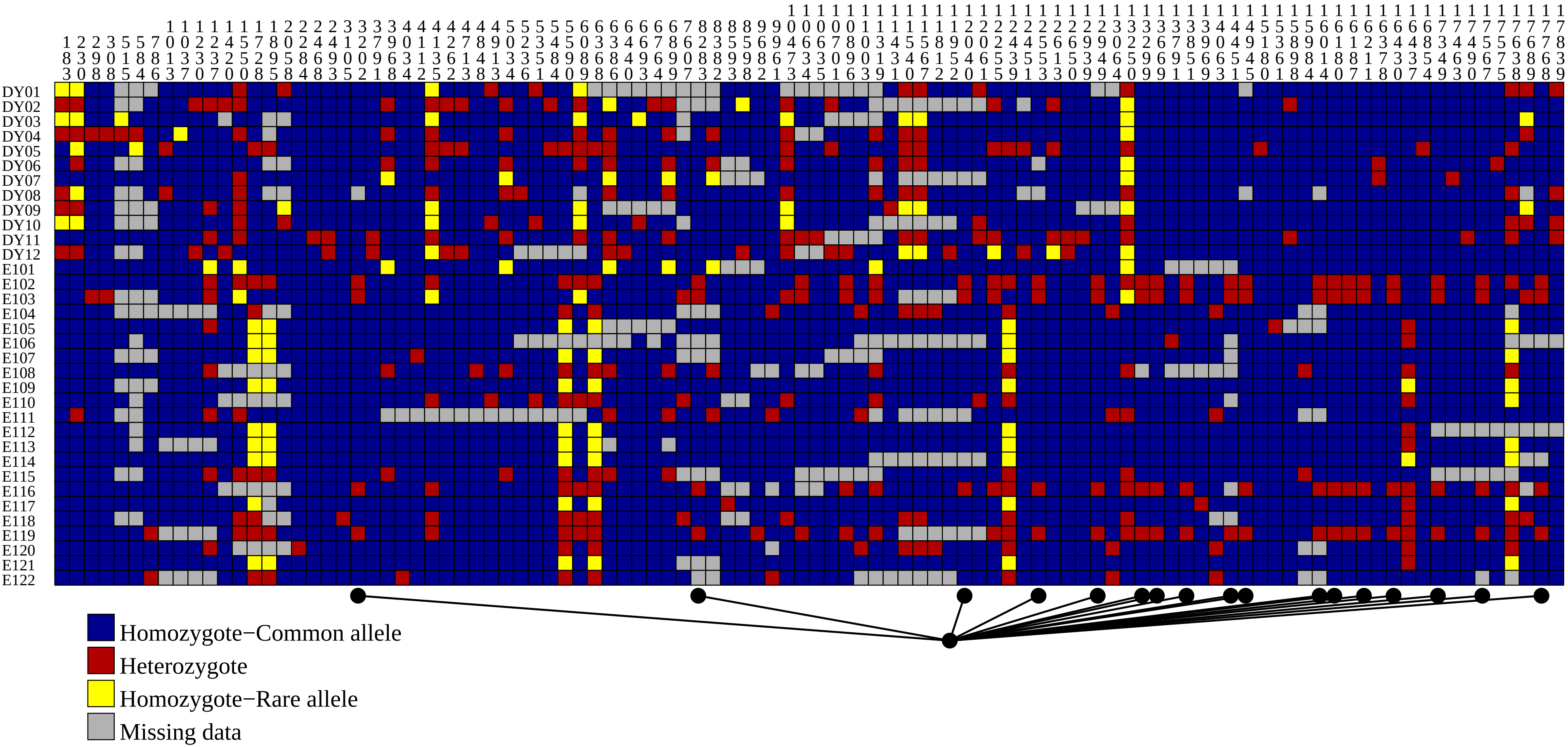

vnn3, p−value: 0.06

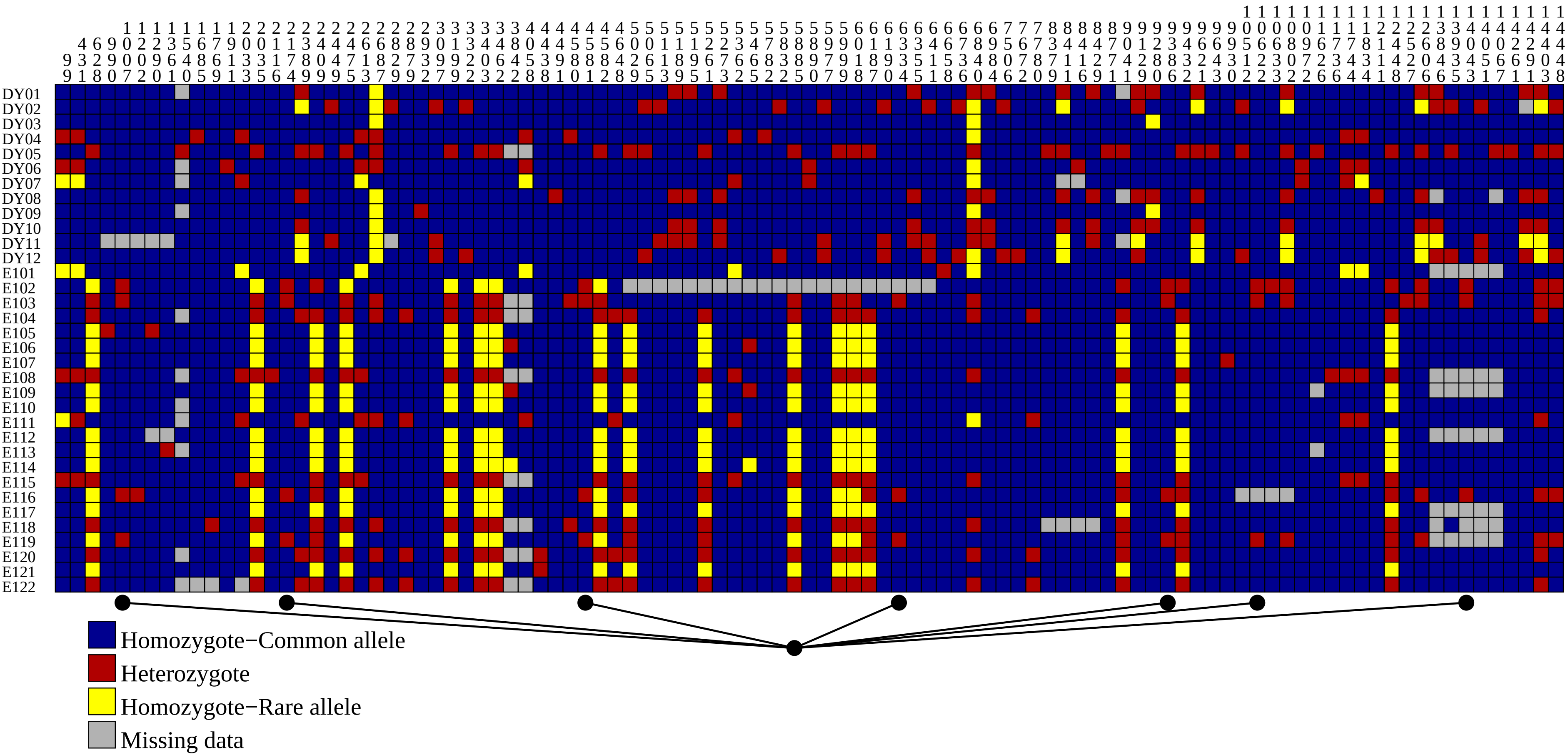

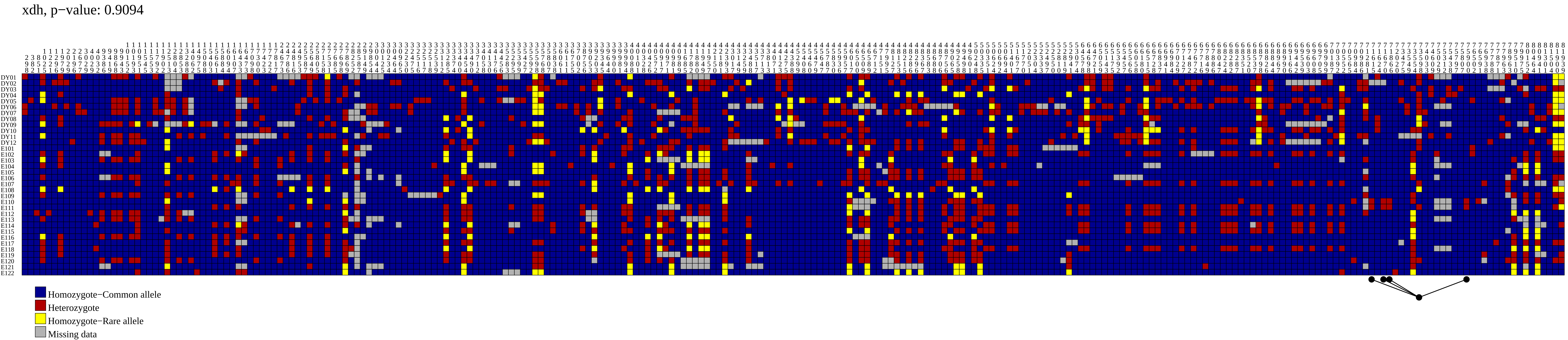
xdh, p−value: 0.9094
Homozygote−Common allele
Heterozygote
Homozygote−Rare allele
Missing data

xrcc4, p−value: 0.2016

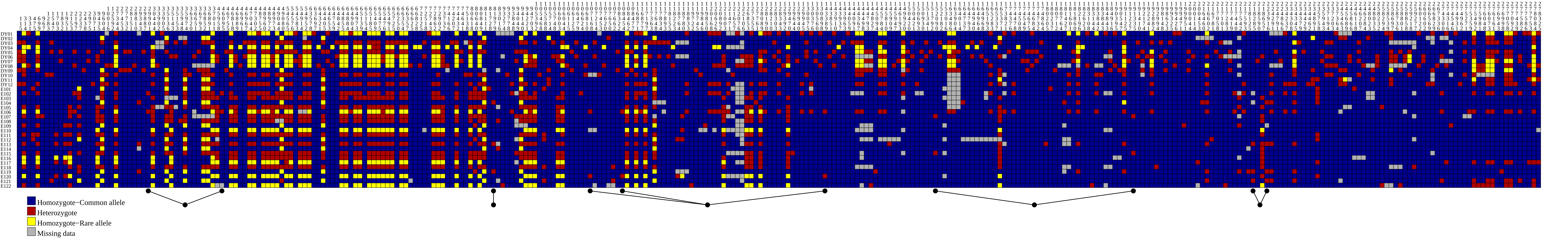

Supplement: Figure S5 — (2.8 MB PDF) [file pgen.0020105.sg005.pdf]
