# Supplementary material for: Possible Ancestral Structure in Human Populations
Source: PLoS Genet. 2006 Jul 28;2(7):e105. doi: 10.1371/journal.pgen.0020105 (PMC1523253; doi:10.1371/journal.pgen.0020105)

abcb1, p−value: 0.04

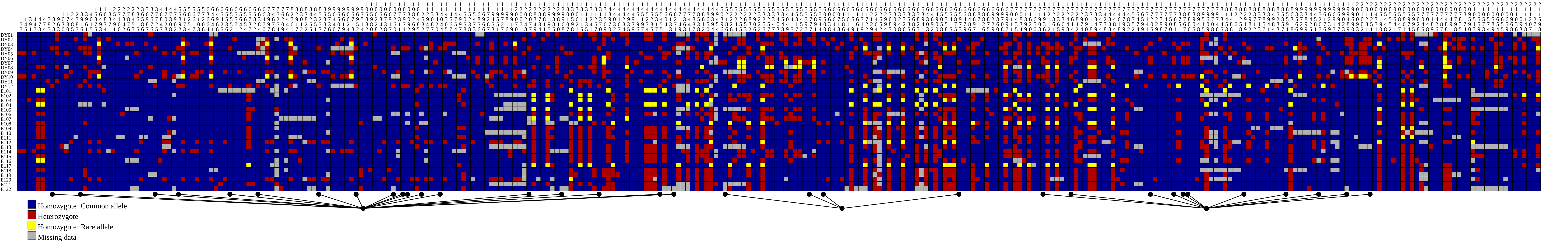

abl1, p−value: 0.0832

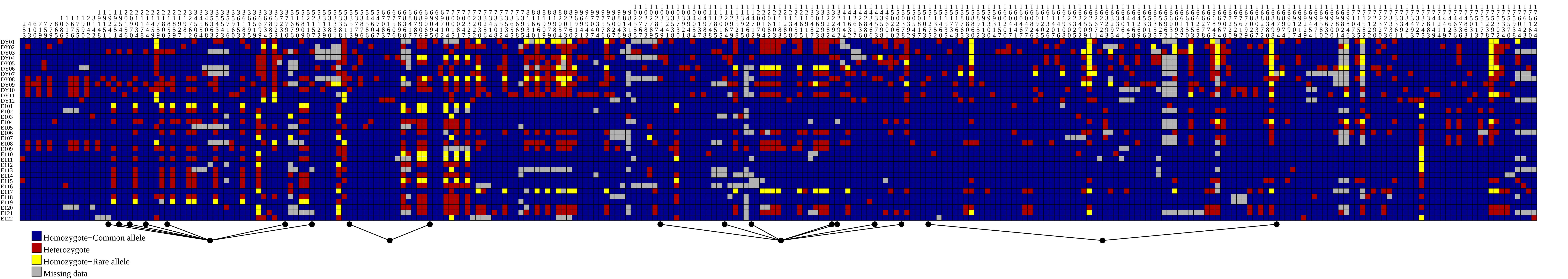

abl2, p−value: 0.1898

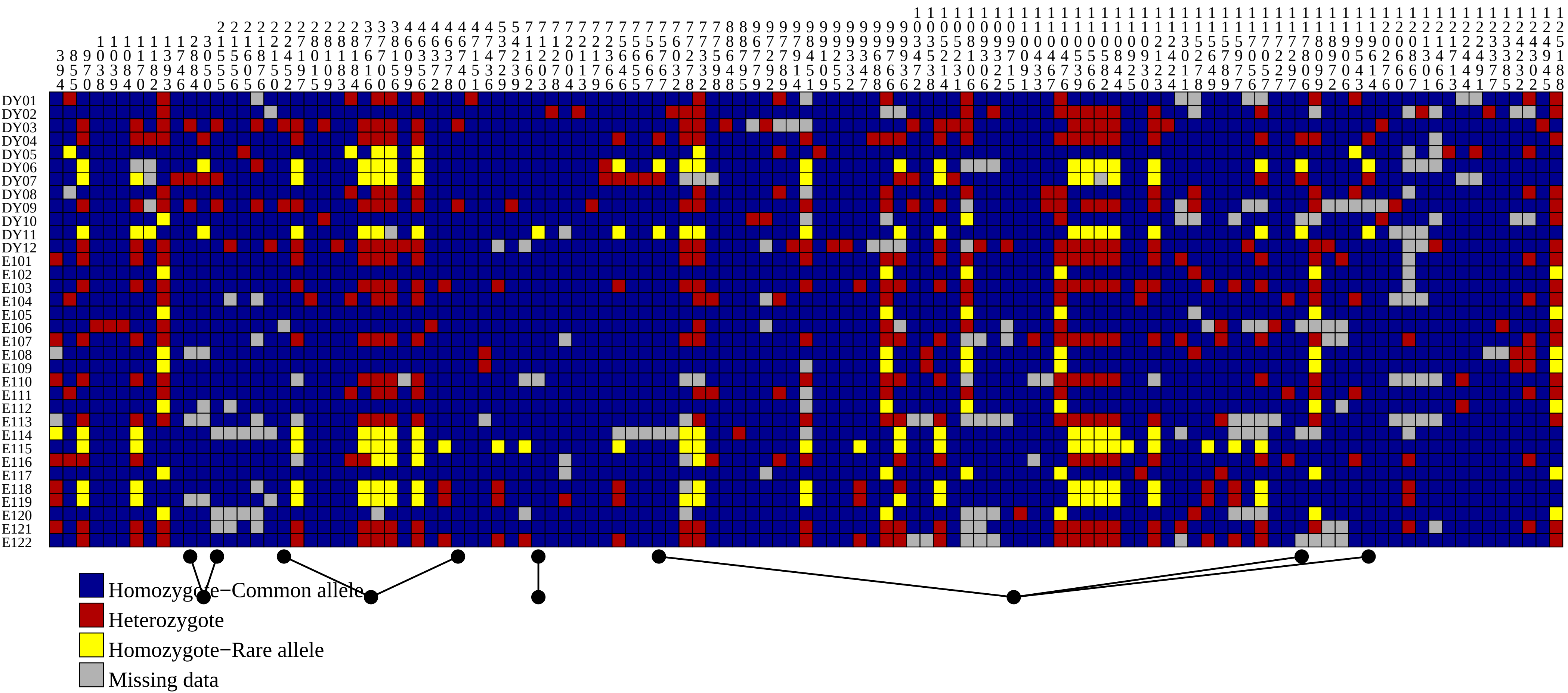

abp1, p−value: 0.516

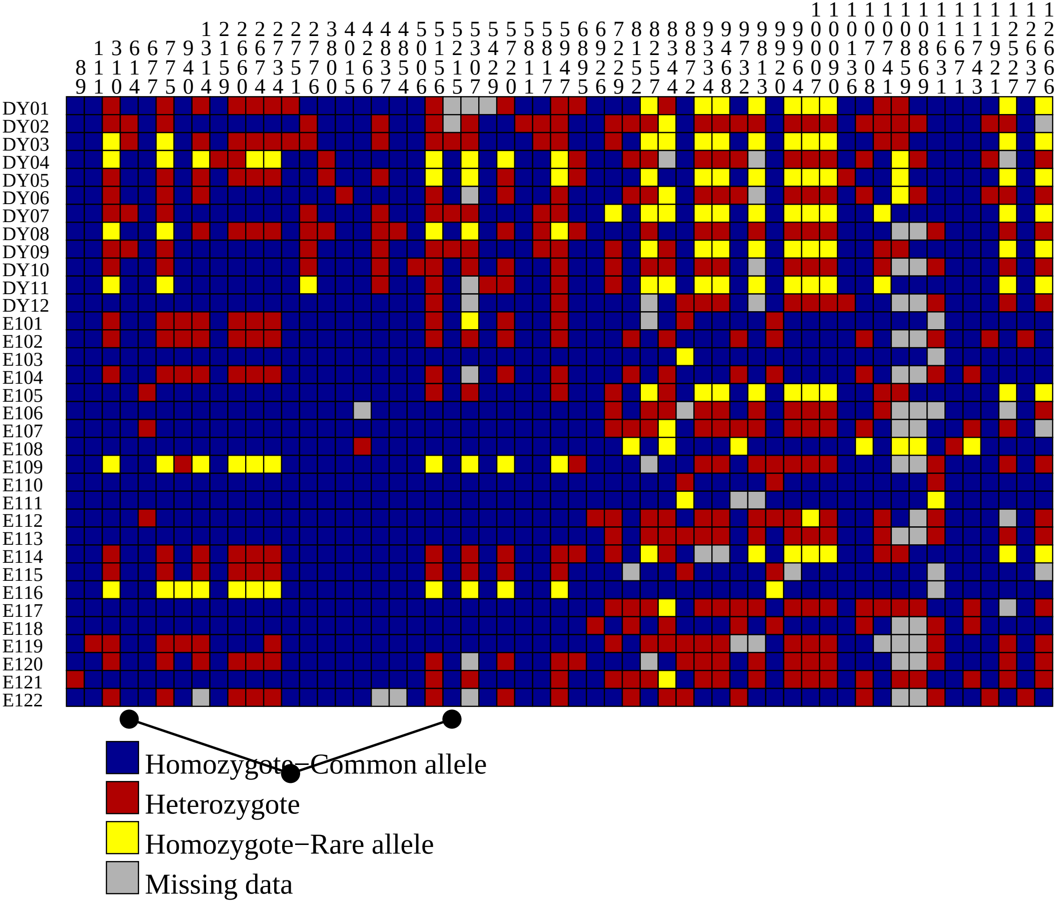

adh1a, p−value: 0.2892

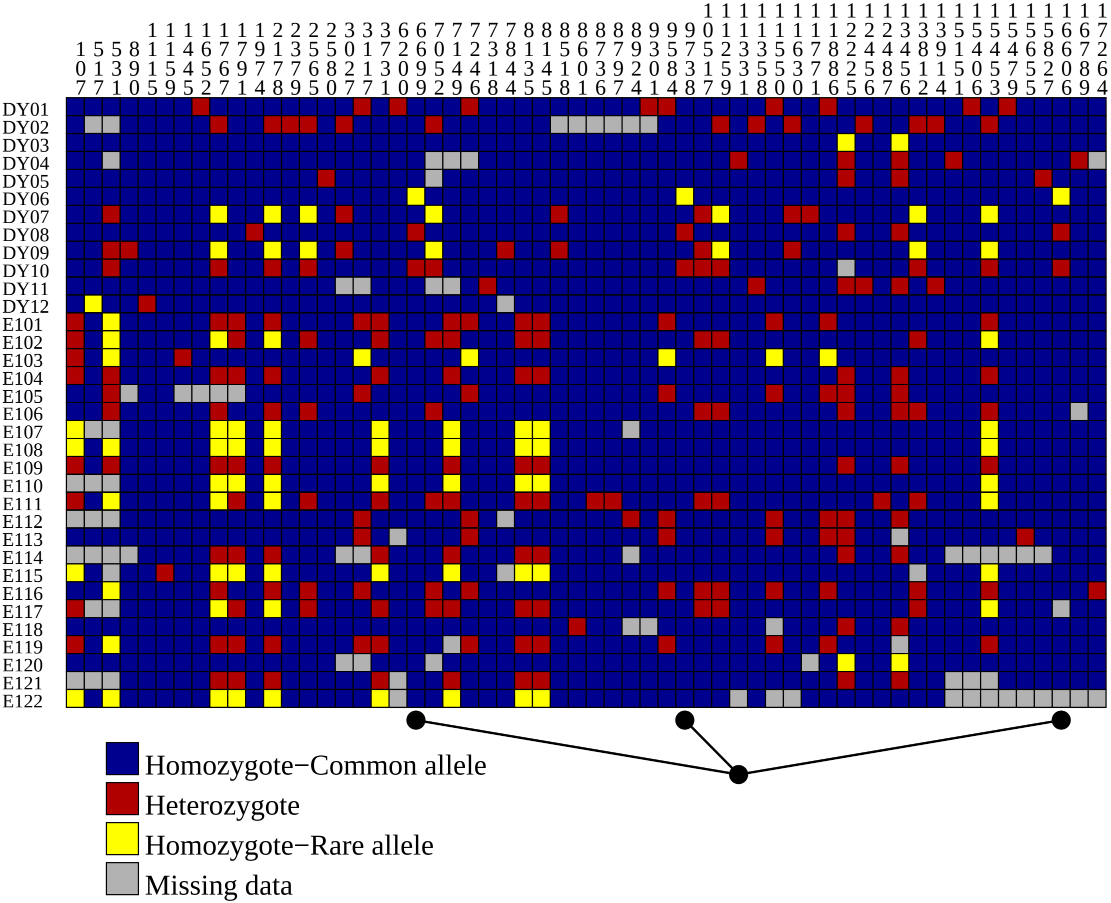

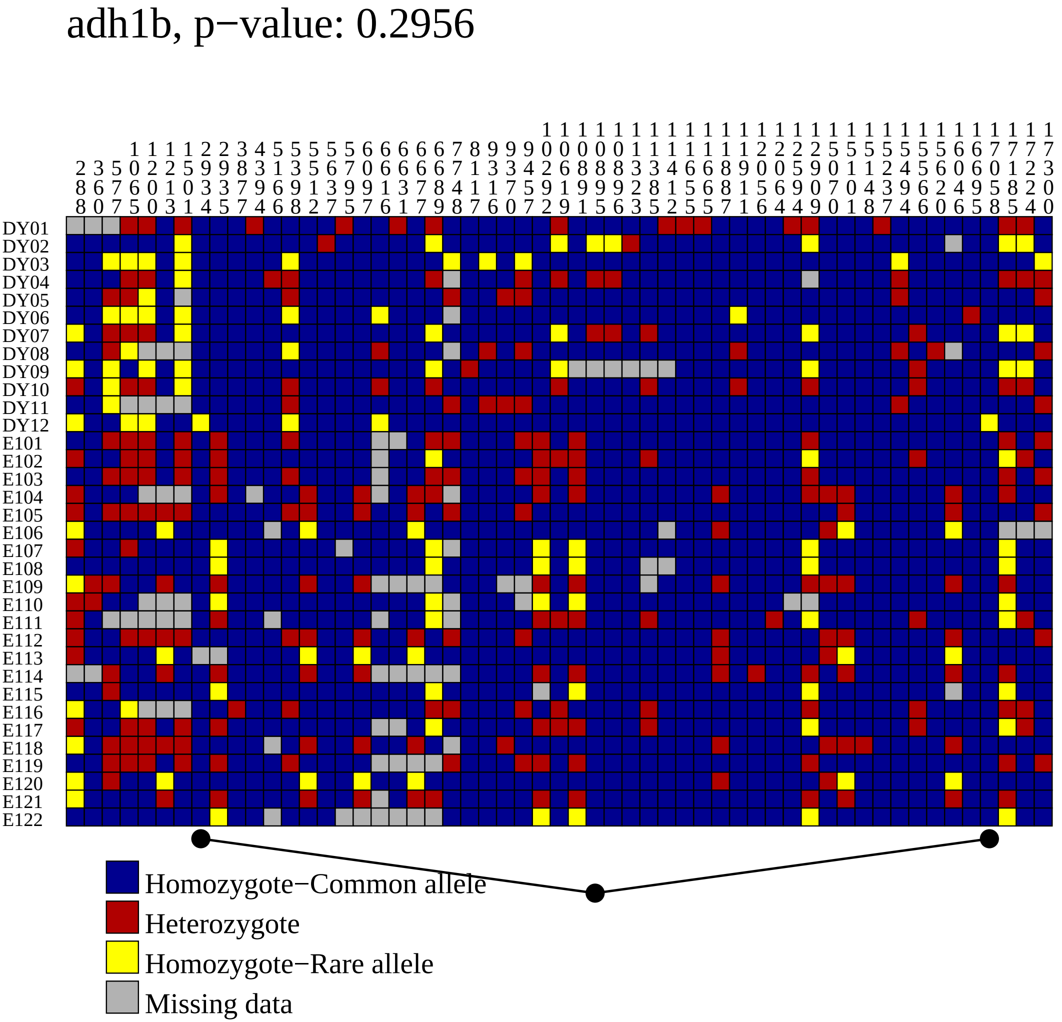
adh1b, p−value: 0.2956
Homozygote−Common allele
Heterozygote
Homozygote−Rare allele
Missing data

adh1c, p−value: 0.8998

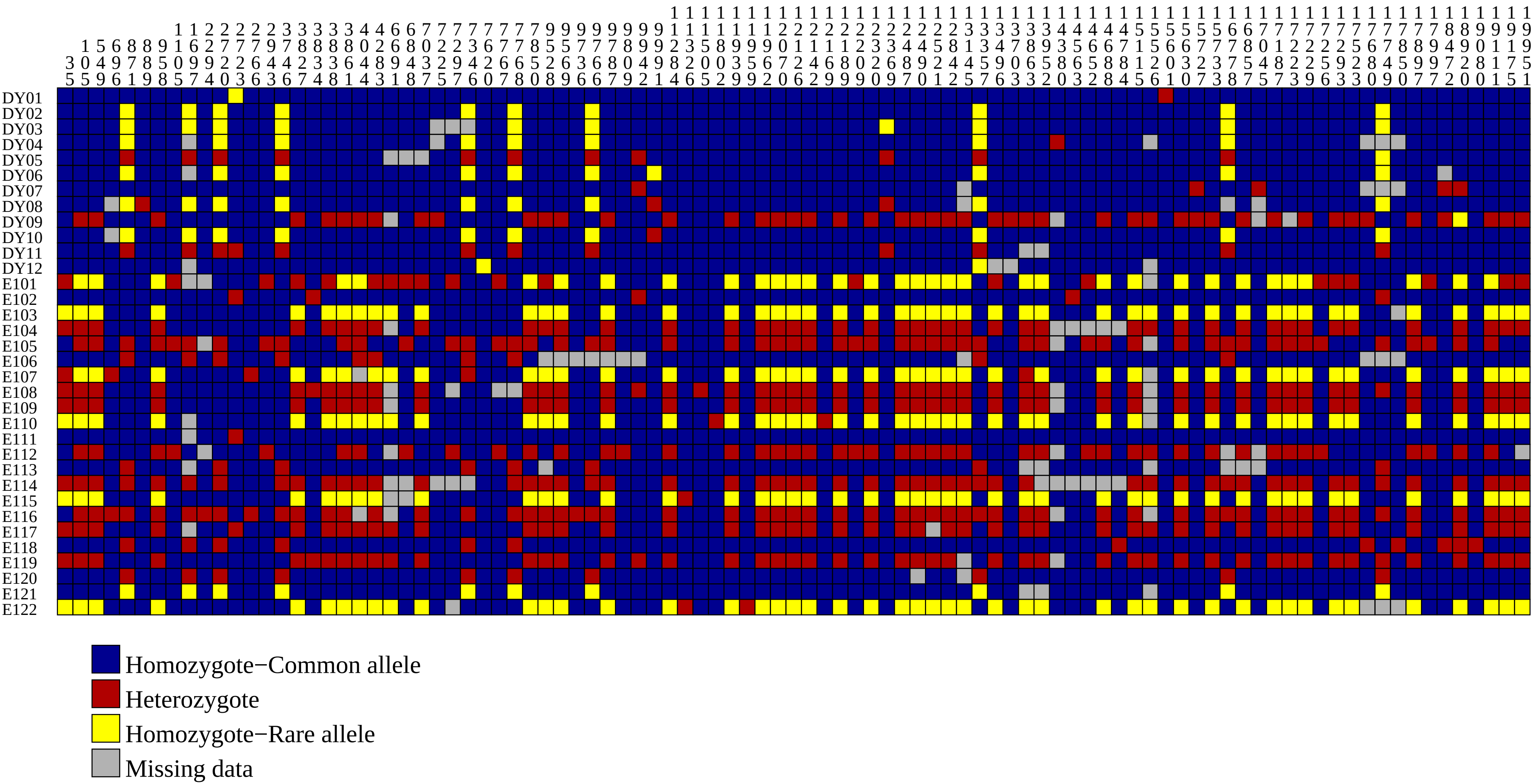

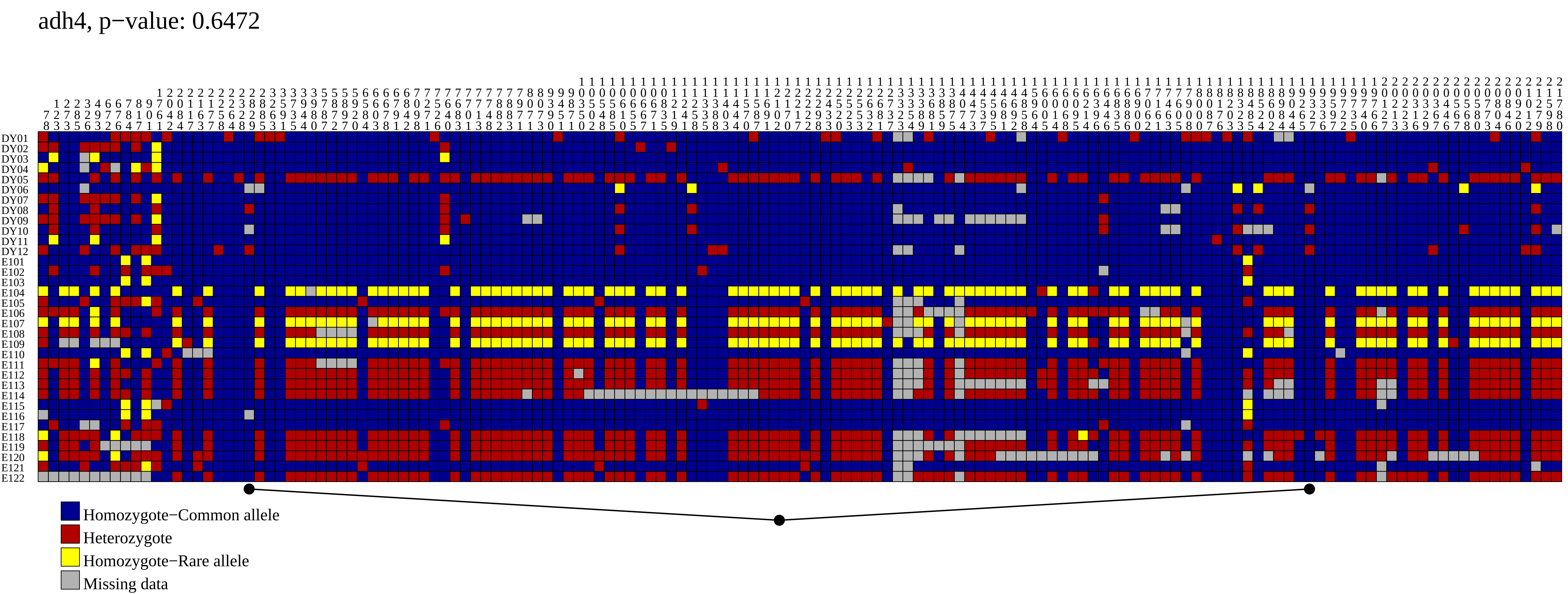
adh4, p−value: 0.6472
Homozygote−Common allele
Heterozygote
Homozygote−Rare allele
Missing data

adh5, p−value: 0.2132

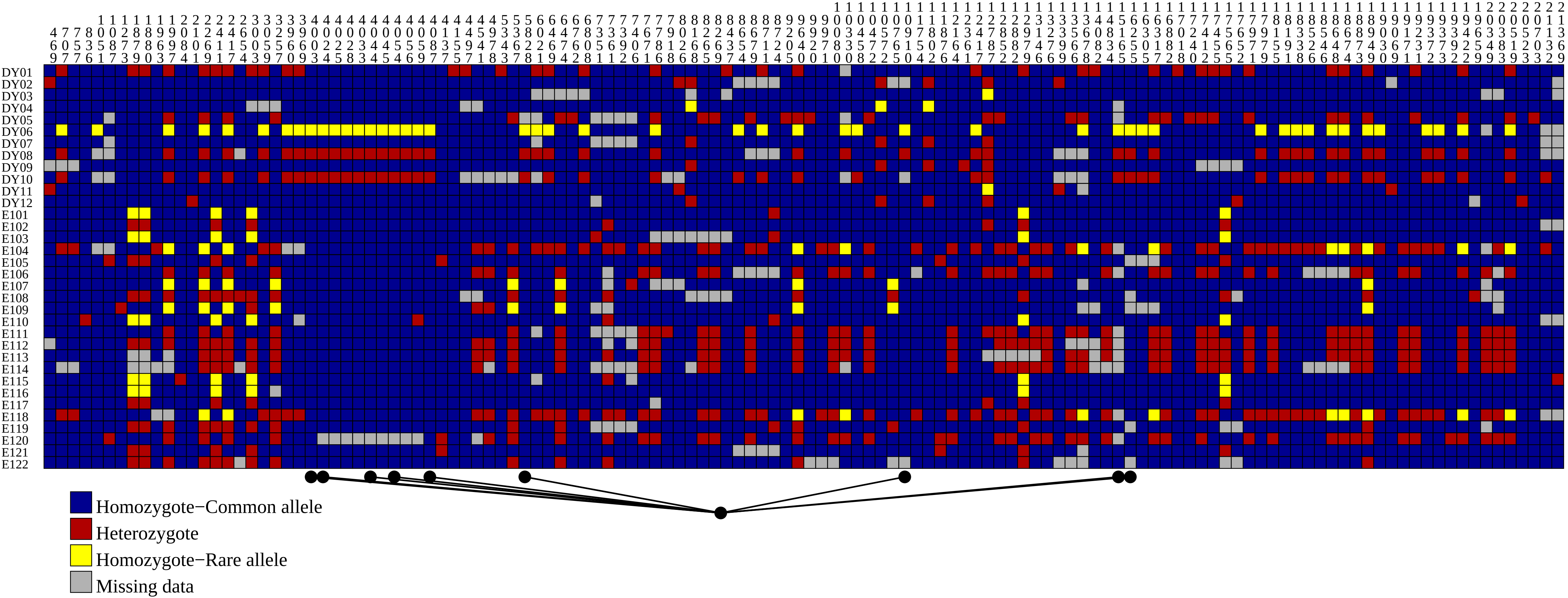

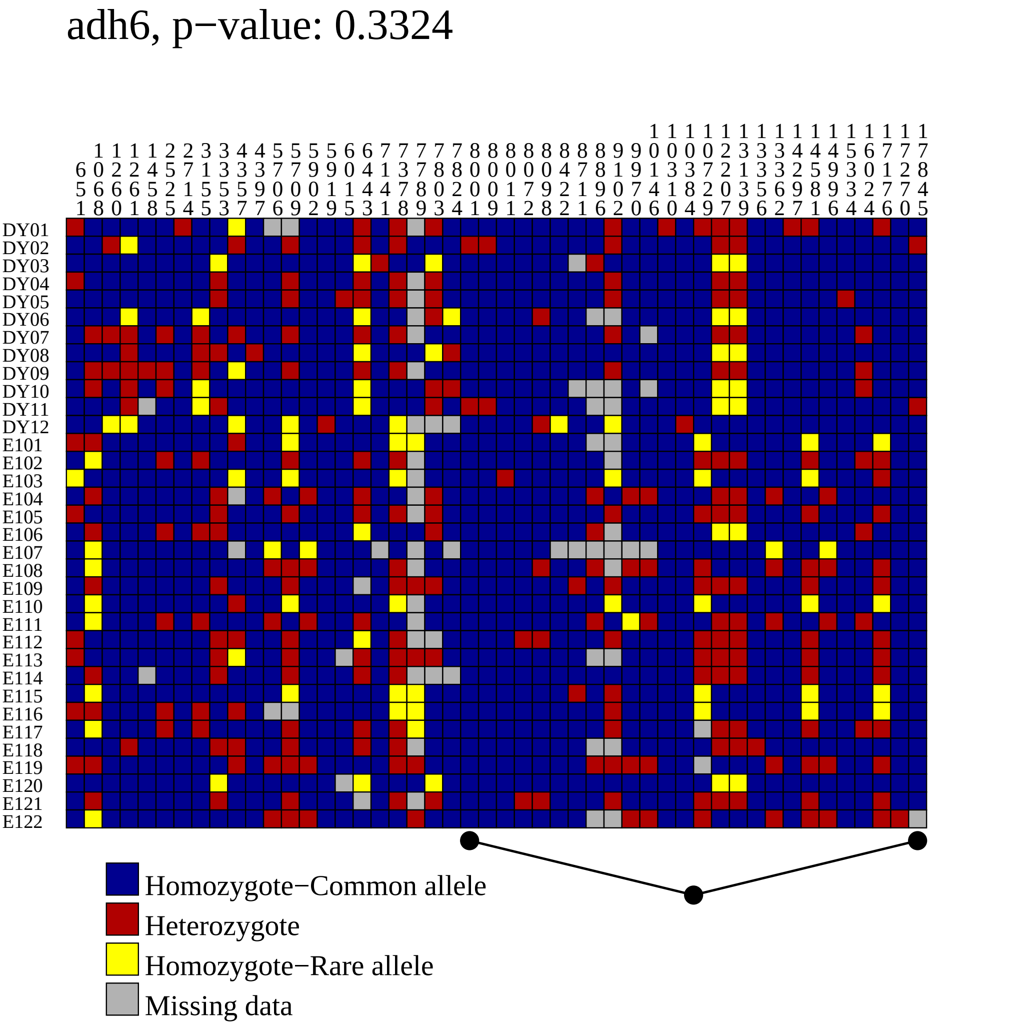
adh6, p−value: 0.3324
651
1068
1260
1261
1458
2525
2714
3155
3353
4357
4397
5706
5709
5902
5919
6015
6443
7141
7378
7789
7803
7824
8001
8009
8011
8072
8098
8422
8721
8896
9102
9970
10146
10310
10384
10729
11207
11339
13356
13362
14297
14581
14996
15334
16024
17176
17270
17845
DY01
DY02
DY03
DY04
DY05
DY06
DY07
DY08
DY09
DY10
DY11
DY12
E101
E102
E103
E104
E105
E106
E107
E108
E109
E110
E111
E112
E113
E114
E115
E116
E117
E118
E119
E120
E121
E122
Homozygote−Common allele
Heterozygote
Homozygote−Rare allele
Missing data

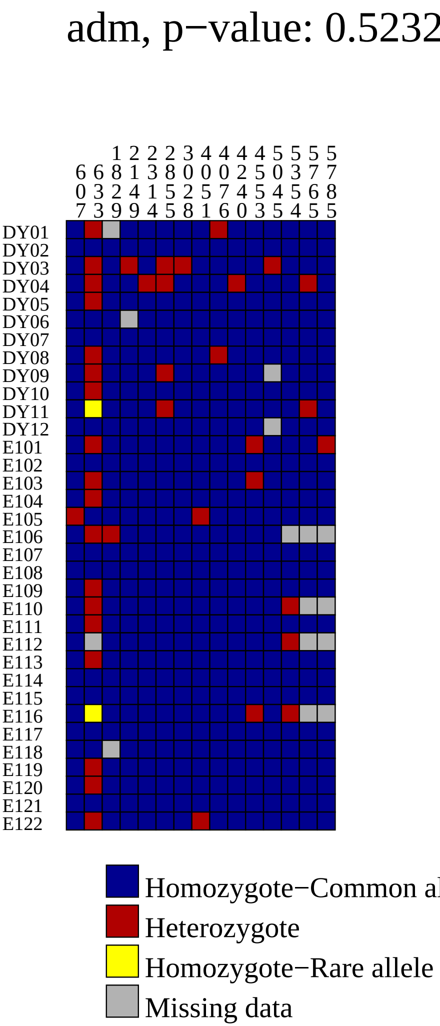
adm, p−value: 0.5232
607
633
1829
2149
2314
2855
3028
4051
4076
4240
4553
5045
5354
5765
5785
DY01
DY02
DY03
DY04
DY05
DY06
DY07
DY08
DY09
DY10
DY11
DY12
E101
E102
E103
E104
E105
E106
E107
E108
E109
E110
E111
E112
E113
E114
E115
E116
E117
E118
E119
E120
E121
E122
Homozygote−Common al
Heterozygote
Homozygote−Rare allele
Missing data

angptl7, p−value: 0.046

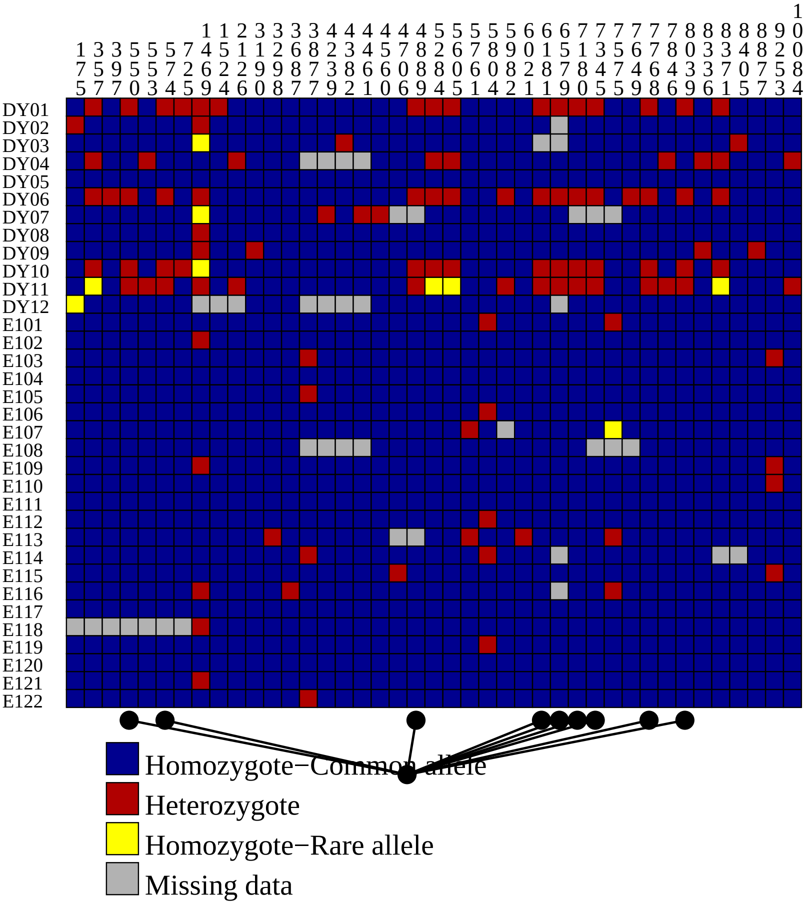

aoc2, p−value: 0.2106

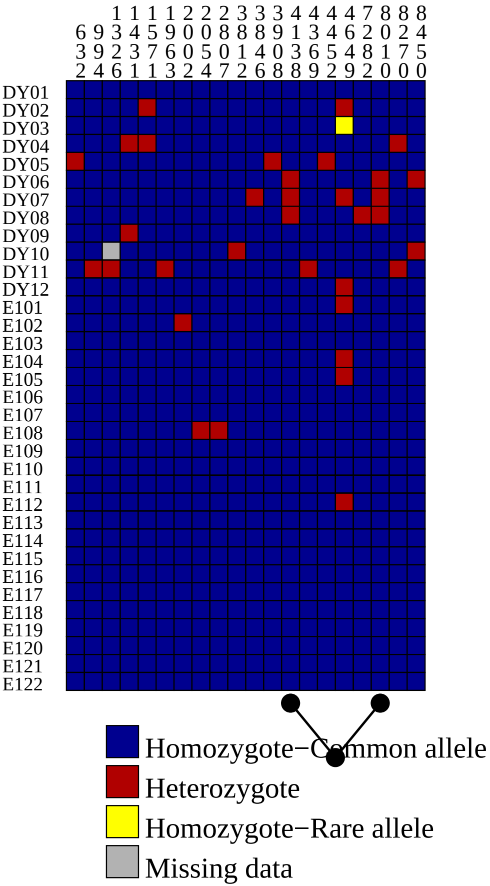

aoc3, p−value: 0.1488

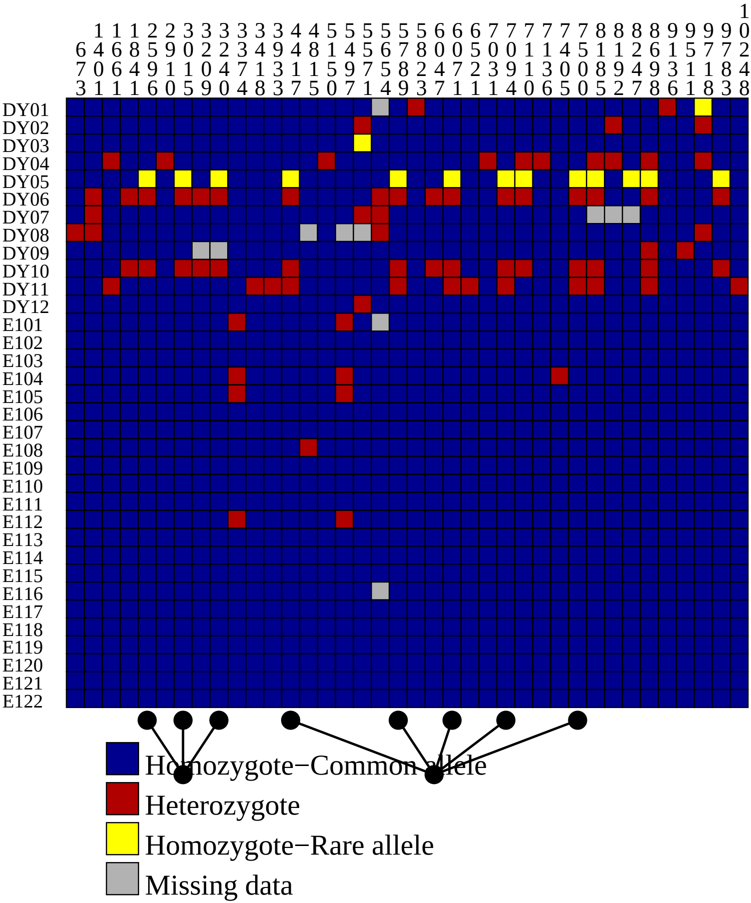

app, p−value: 0.0804

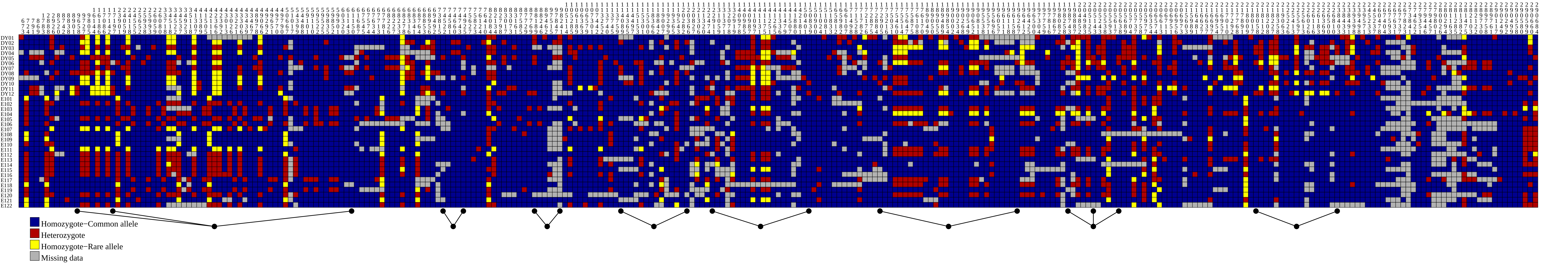

atox1, p−value: 0.2204

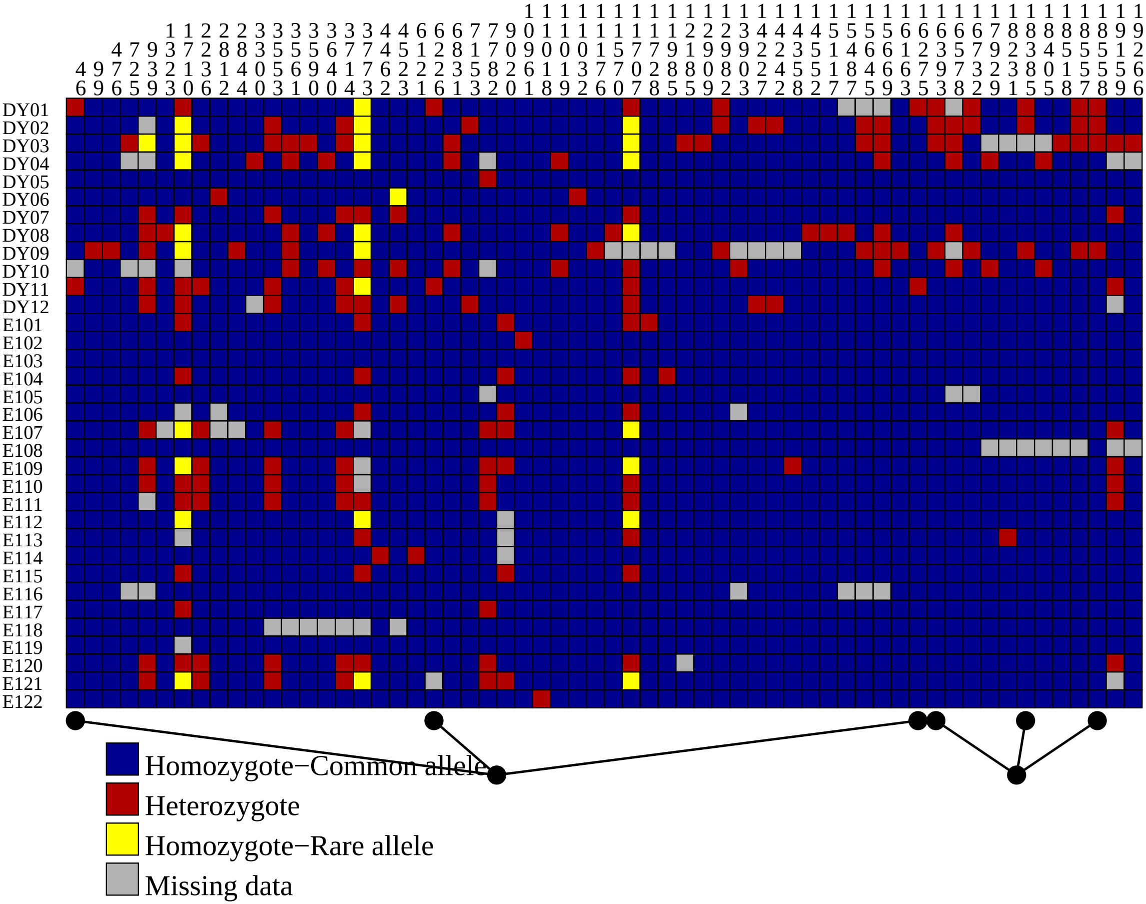

bace1, p−value: 0.3992

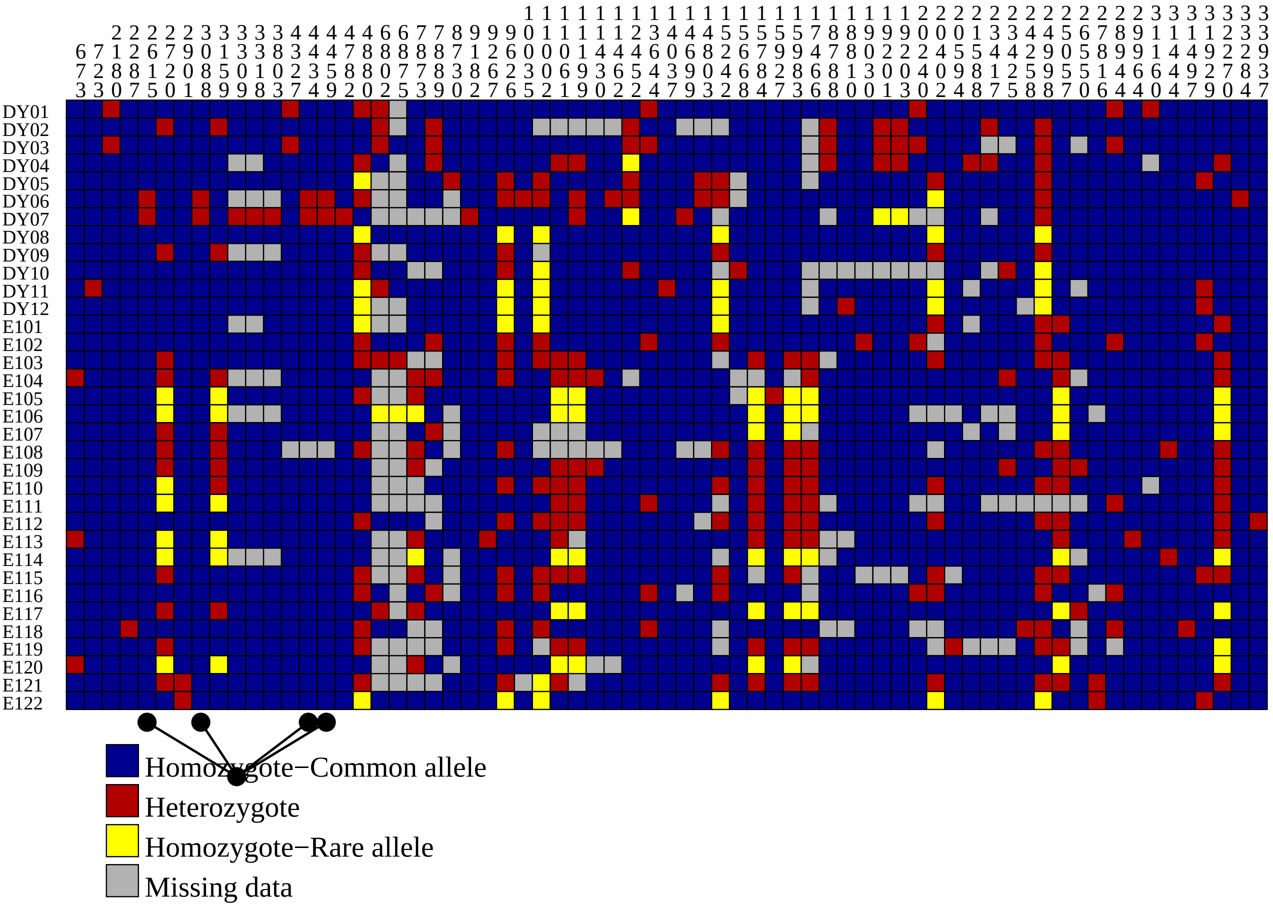

birc2, p−value: 0.2594

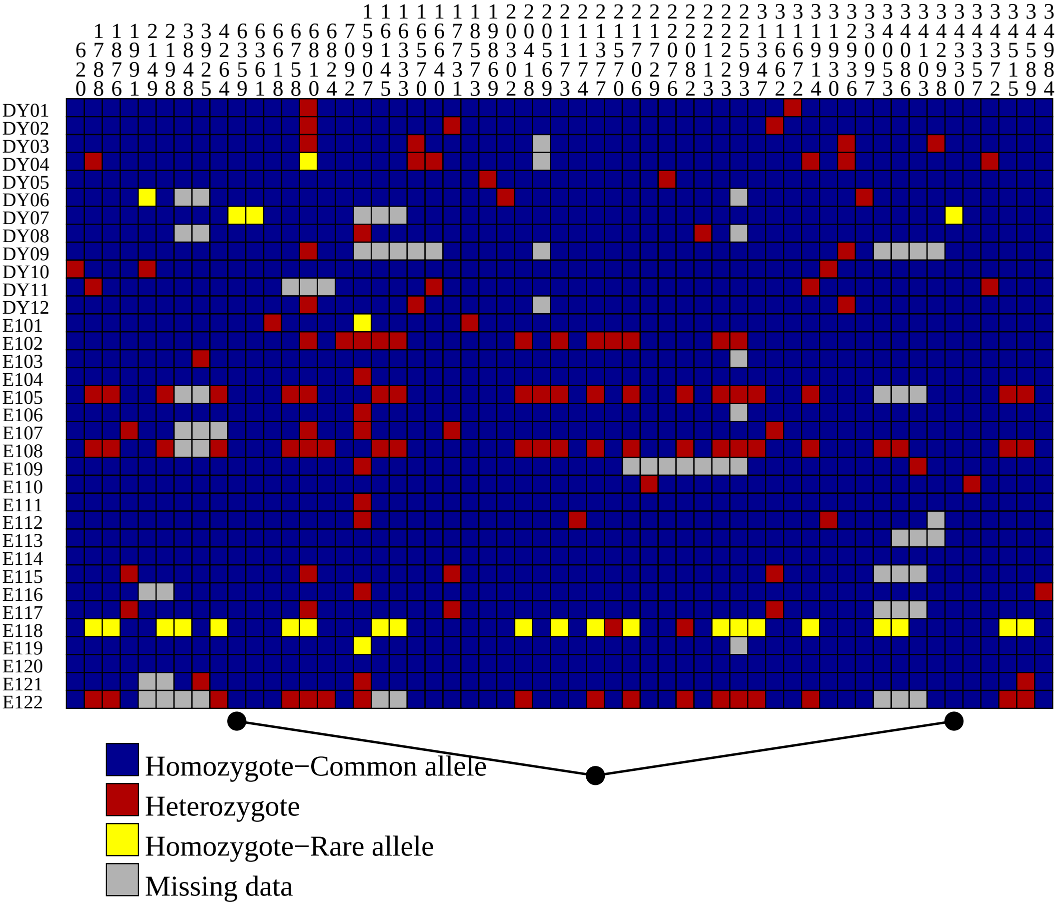

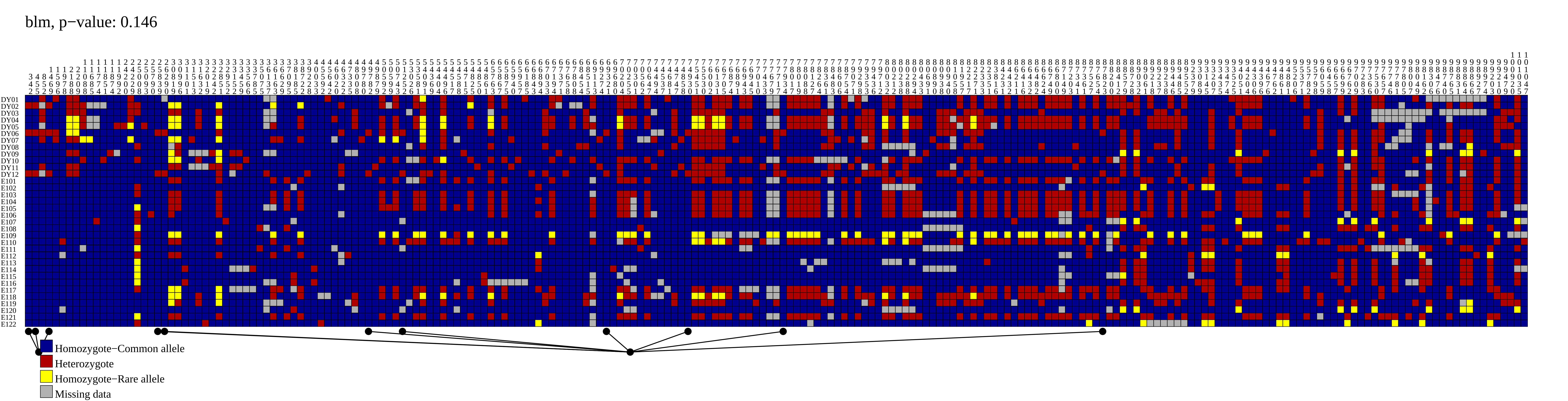

blm, p−value: 0.146
Homozygote−Common allele
Heterozygote
Homozygote−Rare allele
Missing data

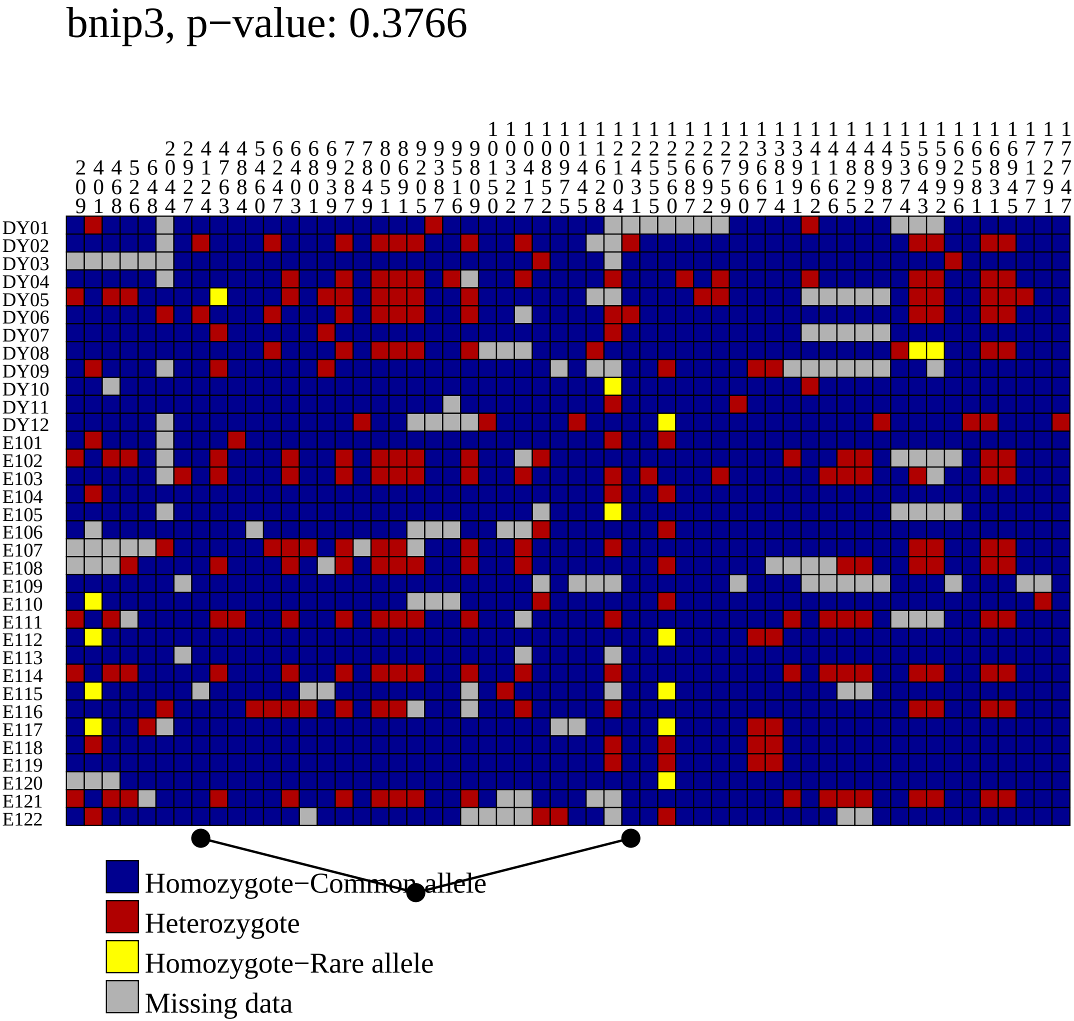
bnip3, p−value: 0.3766
209 401 468 526 648 2044 2927 4124 4763 4884 5460 6247 6403 6801 6939 7287 7849 8051 8691 9205 9387 9516 9809 10150 10322 10417 10852 10975 11445 11628 12104 12431 12555 12560 12687 12692 12759 12960 13667 13814 13991 14162 14166 14825 14892 14987 15374 15643 15992 16296 16581 16831 16945 17177 17291 17747
DY01
DY02
DY03
DY04
DY05
DY06
DY07
DY08
DY09
DY10
DY11
DY12
E101
E102
E103
E104
E105
E106
E107
E108
E109
E110
E111
E112
E113
E114
E115
E116
E117
E118
E119
E120
E121
E122
Homozygote−Common allele
Heterozygote
Homozygote−Rare allele
Missing data

calca, p−value: 0.1004

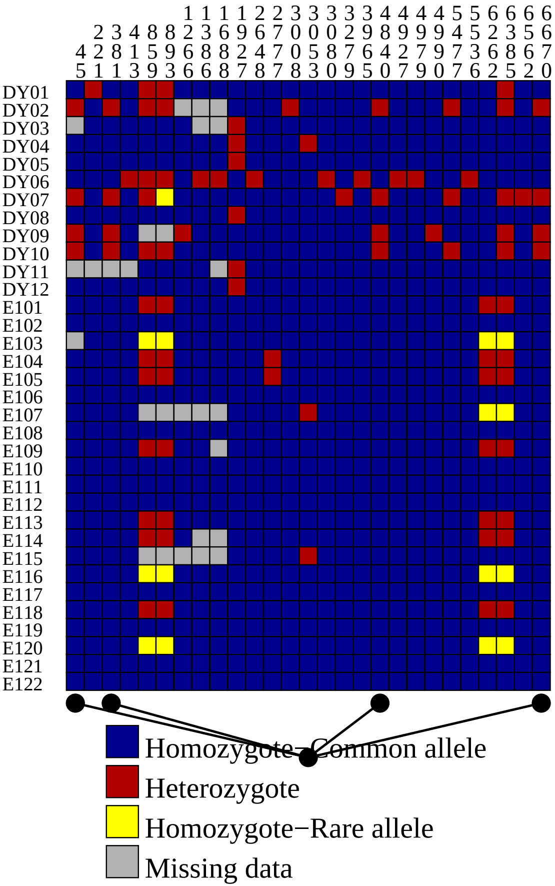

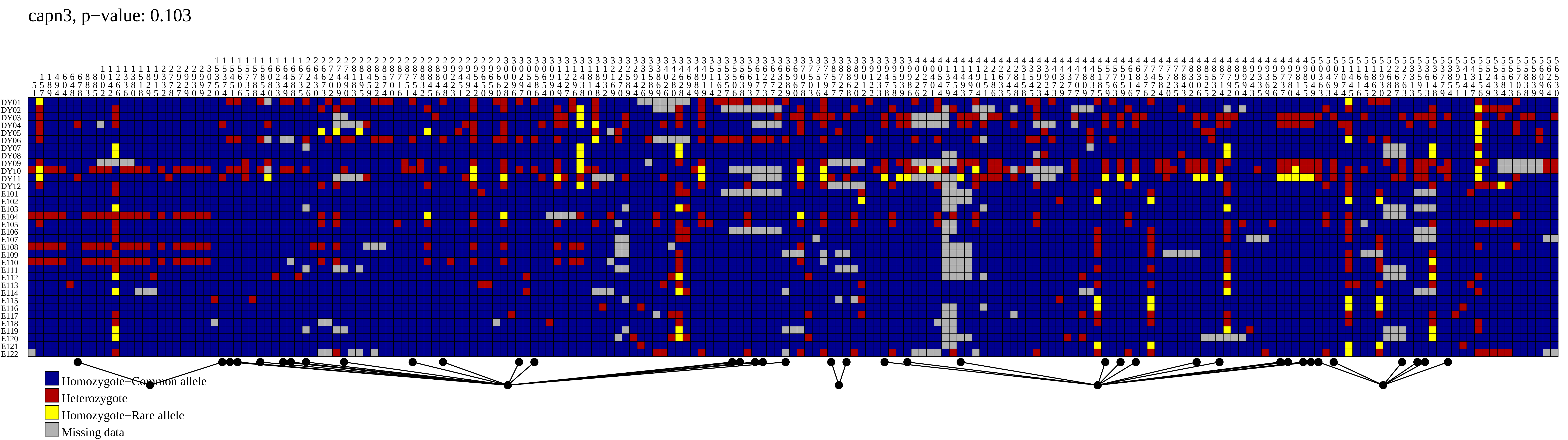
capn3, p−value: 0.103
Homozygote−Common allele
Heterozygote
Homozygote−Rare allele
Missing data

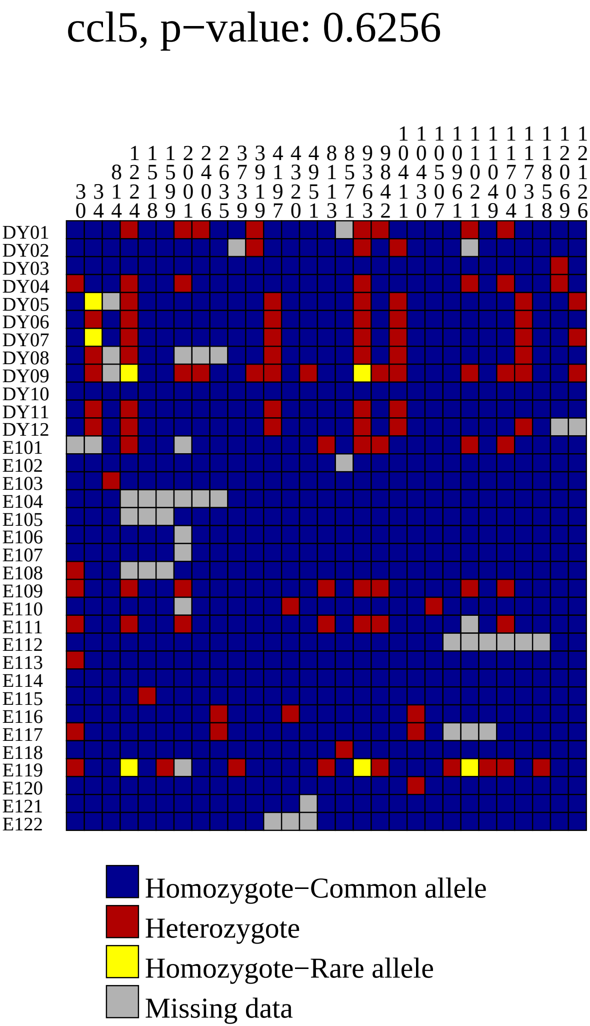

ccl5, p−value: 0.6256
DY01
DY02
DY03
DY04
DY05
DY06
DY07
DY08
DY09
DY10
DY11
DY12
E101
E102
E103
E104
E105
E106
E107
E108
E109
E110
E111
E112
E113
E114
E115
E116
E117
E118
E119
E120
E121
E122
Homozygote−Common allele
Heterozygote
Homozygote−Rare allele
Missing data

cd3z, p−value: 0.5752

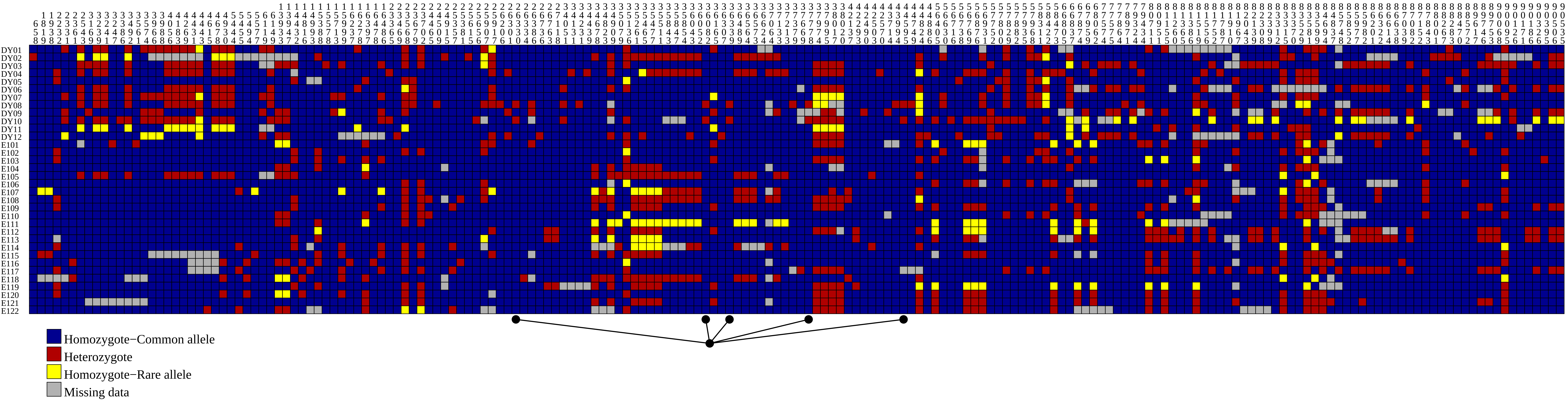

cd4, p−value: 0.4368

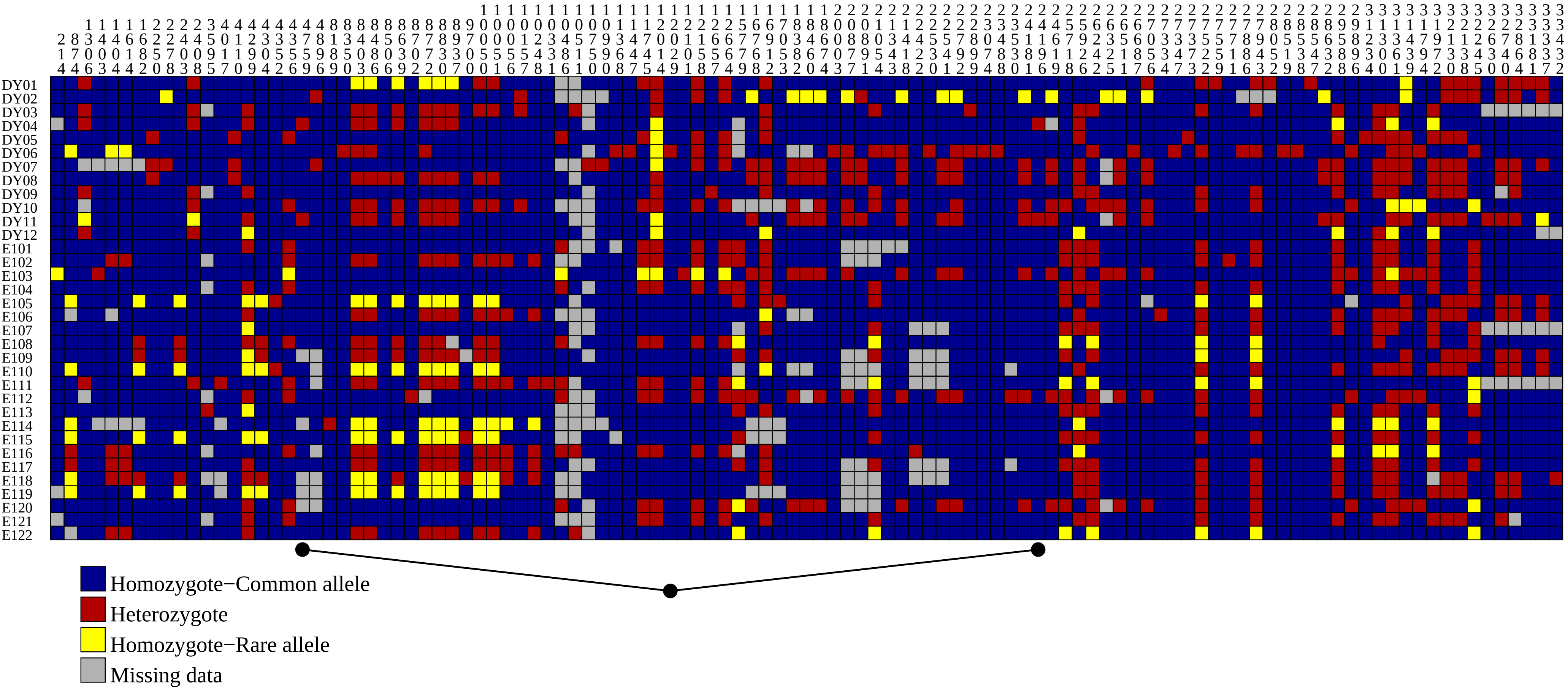

cdc37, p−value: 0.1266

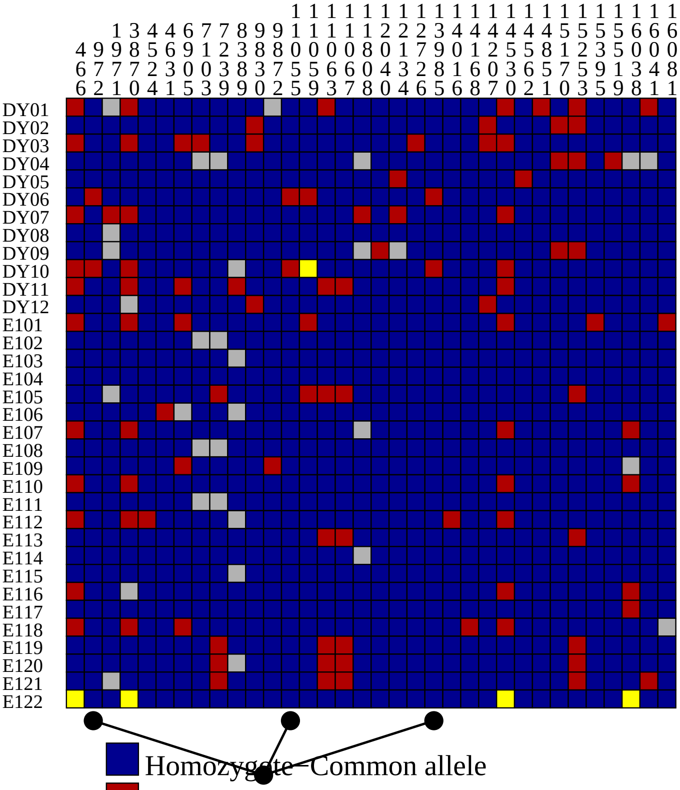

Homozygote−Common allele

Heterozygote

Homozygote−Rare allele

Missing data

cdh1, p−value: 0.3846

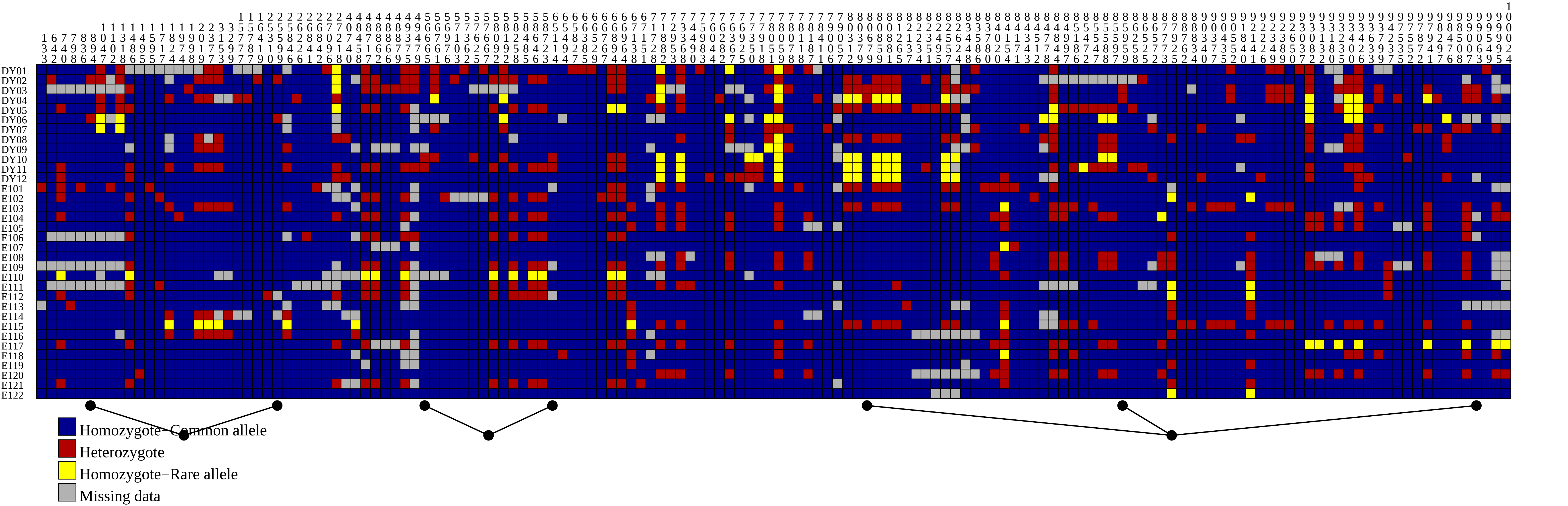

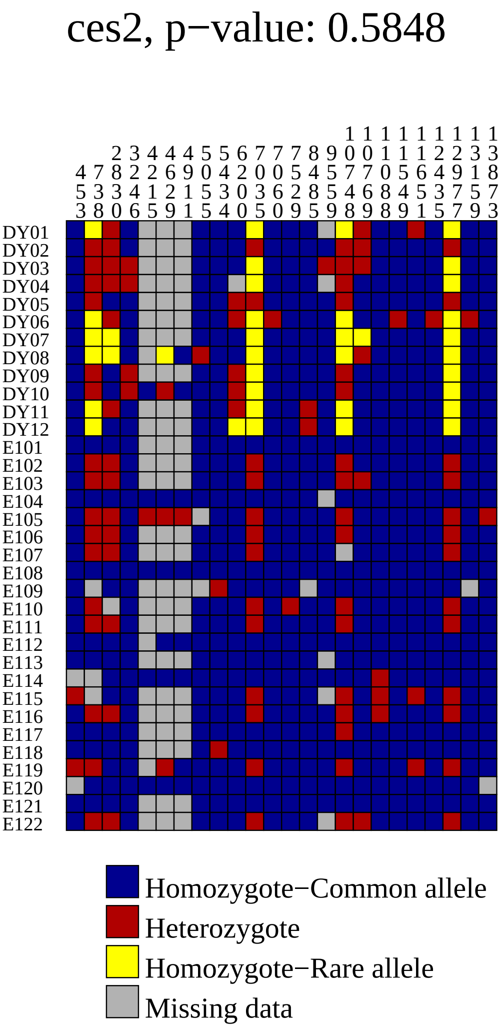
ces2, p−value: 0.5848
453
738
2830
3246
4215
4629
4911
5055
5434
6200
7035
7060
7529
8485
9559
10748
10769
11088
11549
11651
12435
12977
13159
13873
DY01
DY02
DY03
DY04
DY05
DY06
DY07
DY08
DY09
DY10
DY11
DY12
E101
E102
E103
E104
E105
E106
E107
E108
E109
E110
E111
E112
E113
E114
E115
E116
E117
E118
E119
E120
E121
E122
Homozygote−Common allele
Heterozygote
Homozygote−Rare allele
Missing data

chrna4, p−value: 0.8434

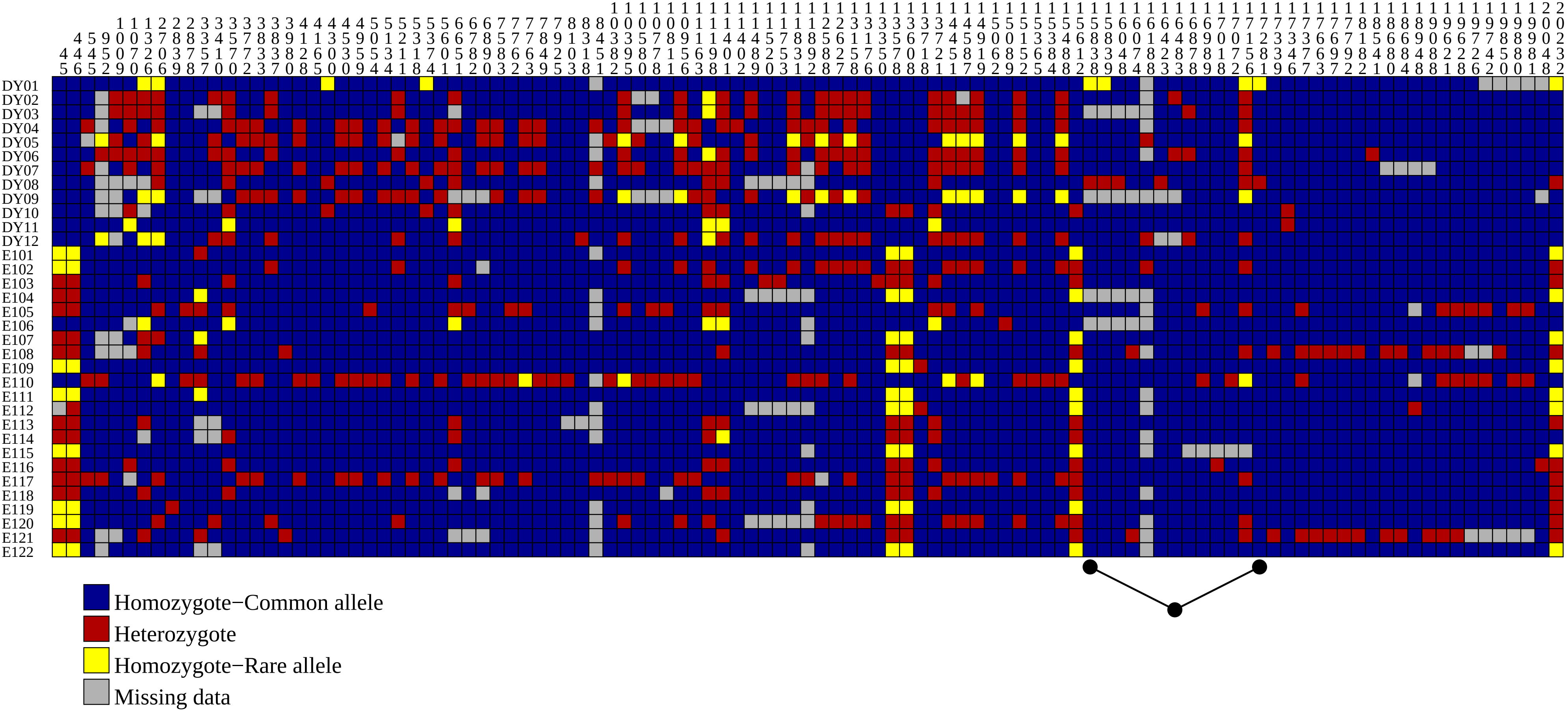

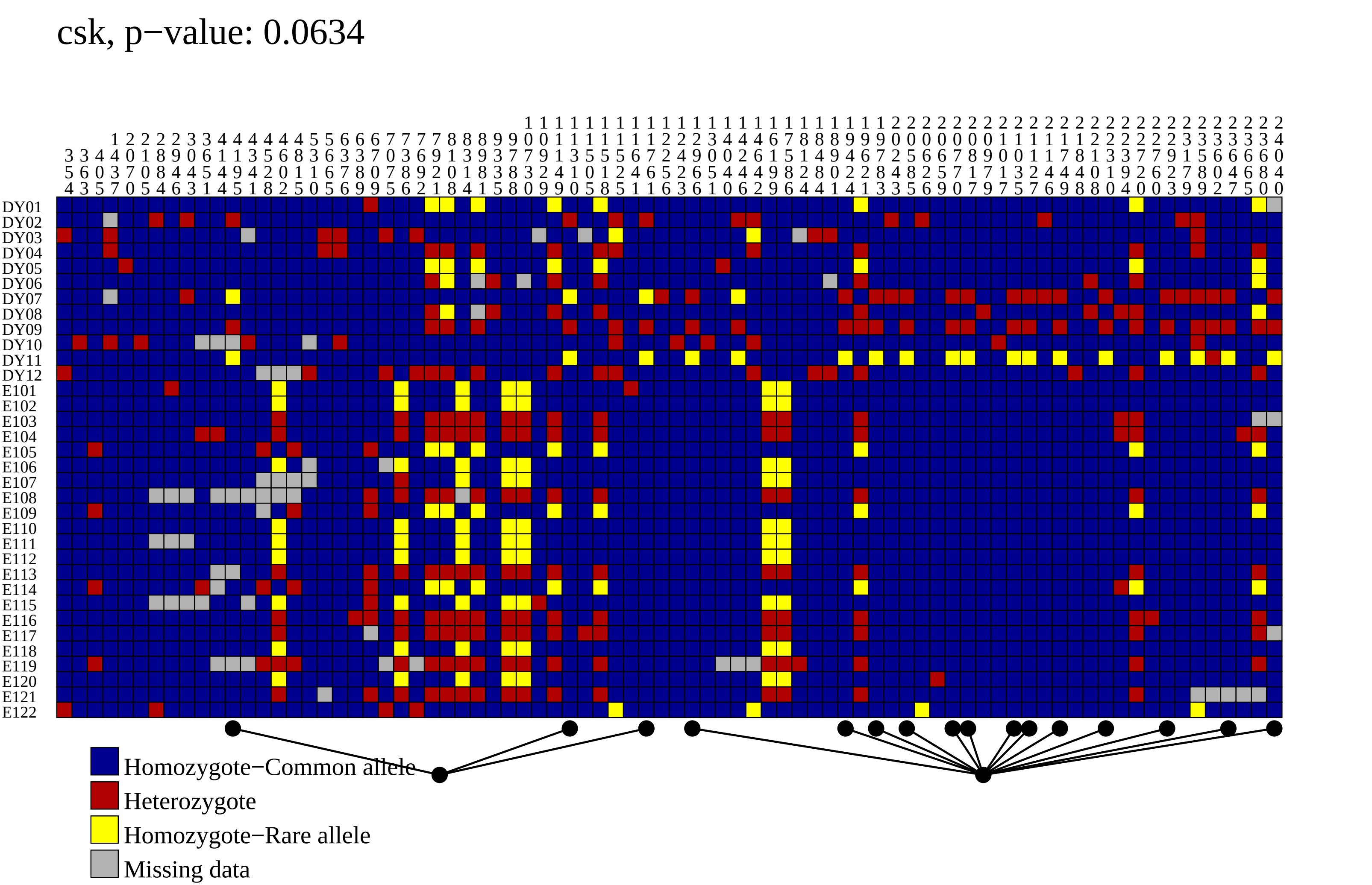
csk, p−value: 0.0634
DY01
DY02
DY03
DY04
DY05
DY06
DY07
DY08
DY09
DY10
DY11
DY12
E101
E102
E103
E104
E105
E106
E107
E108
E109
E110
E111
E112
E113
E114
E115
E116
E117
E118
E119
E120
E121
E122
Homozygote−Common allele
Heterozygote
Homozygote−Rare allele
Missing data

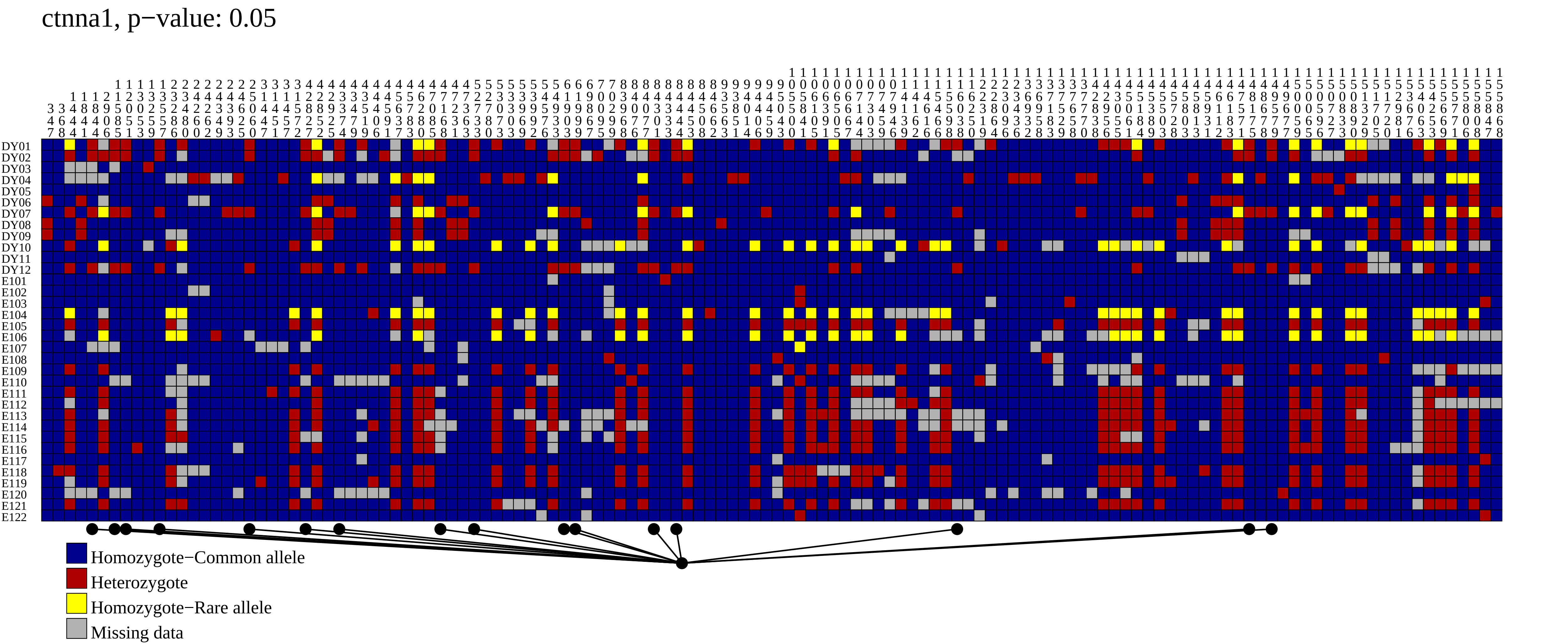
ctnna1, p−value: 0.05
DY01
DY02
DY03
DY04
DY05
DY06
DY07
DY08
DY09
DY10
DY11
DY12
E101
E102
E103
E104
E105
E106
E107
E108
E109
E110
E111
E112
E113
E114
E115
E116
E117
E118
E119
E120
E121
E122
Homozygote−Common allele
Heterozygote
Homozygote−Rare allele
Missing data

cyp19a1, p−value: 0.3276

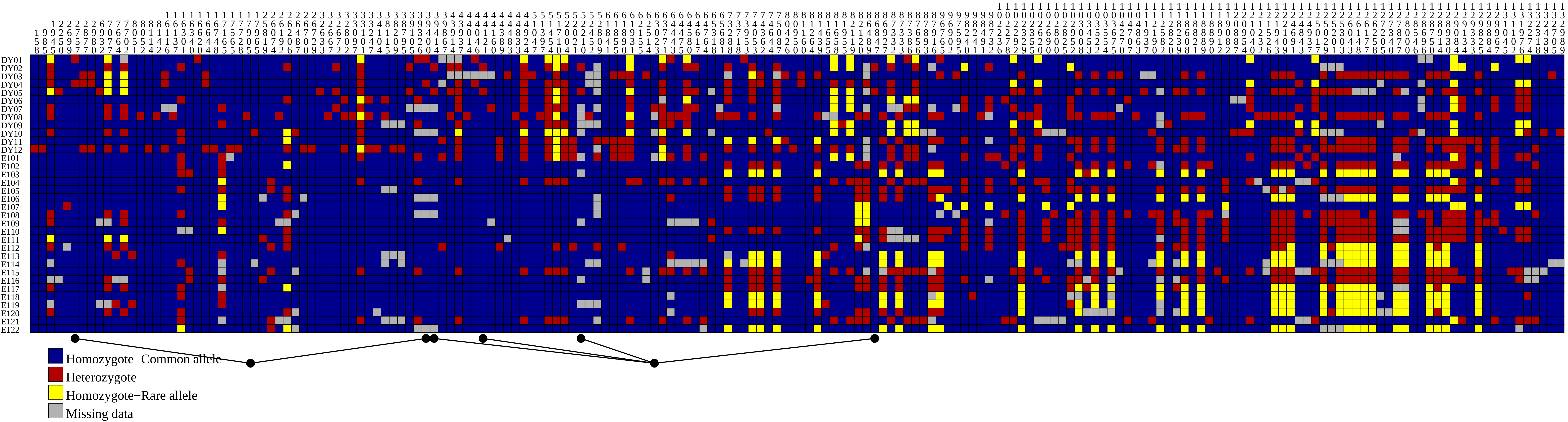

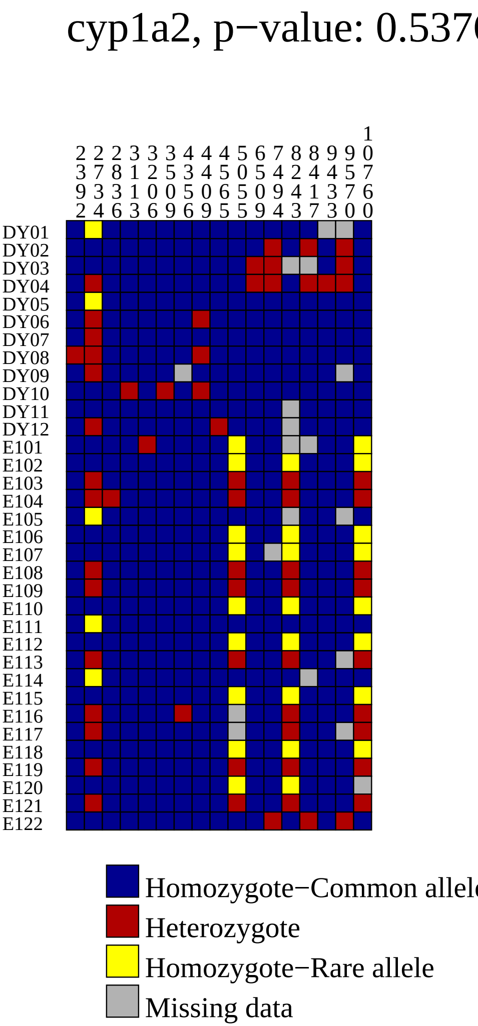
cyp1a2, p−value: 0.537
2392
2734
2836
3113
3206
3509
4356
4409
4565
5055
6509
7494
8243
8417
9433
9570
10760
DY01
DY02
DY03
DY04
DY05
DY06
DY07
DY08
DY09
DY10
DY11
DY12
E101
E102
E103
E104
E105
E106
E107
E108
E109
E110
E111
E112
E113
E114
E115
E116
E117
E118
E119
E120
E121
E122
Homozygote−Common allel
Heterozygote
Homozygote−Rare allele
Missing data

cyp2b6, p−value: 0.2506

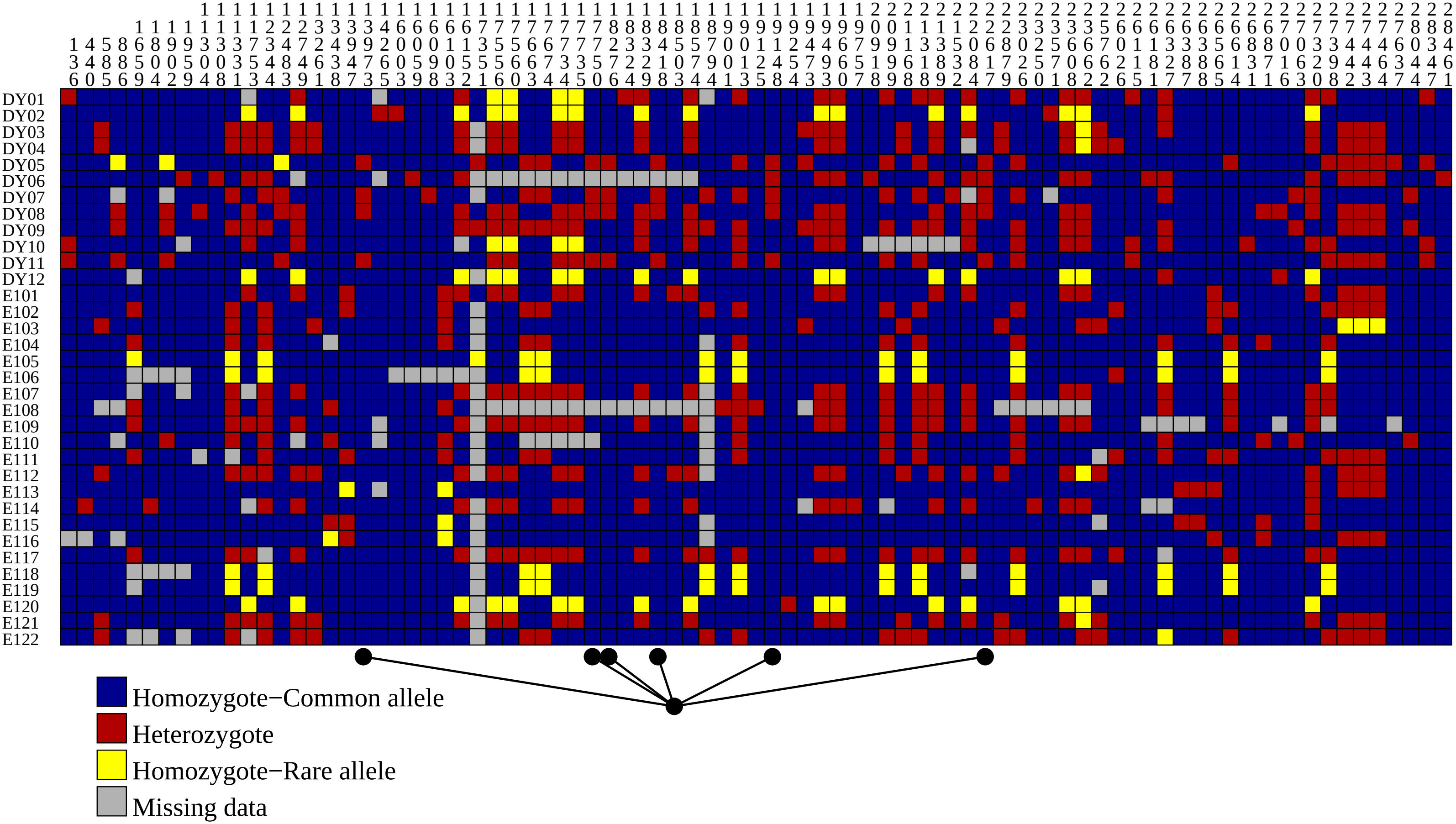

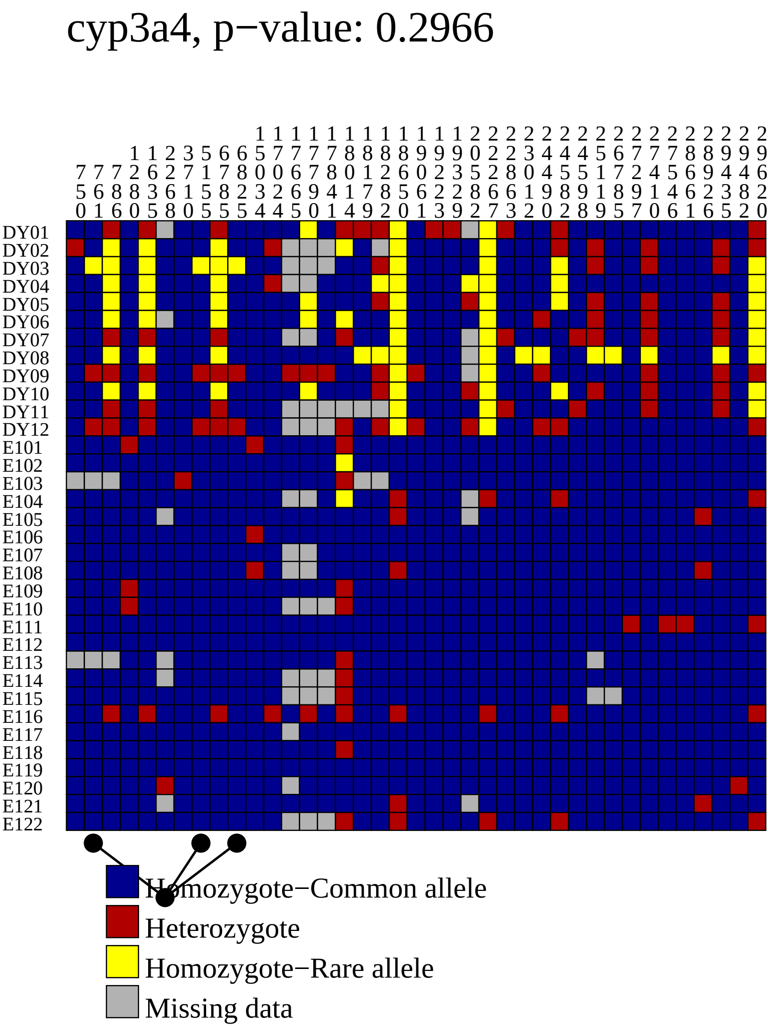
cyp3a4, p−value: 0.2966
750 761 786 1280 1635 2268 3710 5155 6785 6825 15034 17024 17665 17790 17841 18014 18179 18282 18650 19061 19223 19329 20582 22267 22863 23012 24490 24582 24598 25119 26785 27297 27410 27546 28261 28926 29435 29482 29620
DY01 DY02 DY03 DY04 DY05 DY06 DY07 DY08 DY09 DY10 DY11 DY12
E101 E102 E103 E104 E105 E106 E107 E108 E109 E110 E111 E112 E113 E114 E115 E116 E117 E118 E119 E120 E121 E122
Homozygote−Common allele
Heterozygote
Homozygote−Rare allele
Missing data

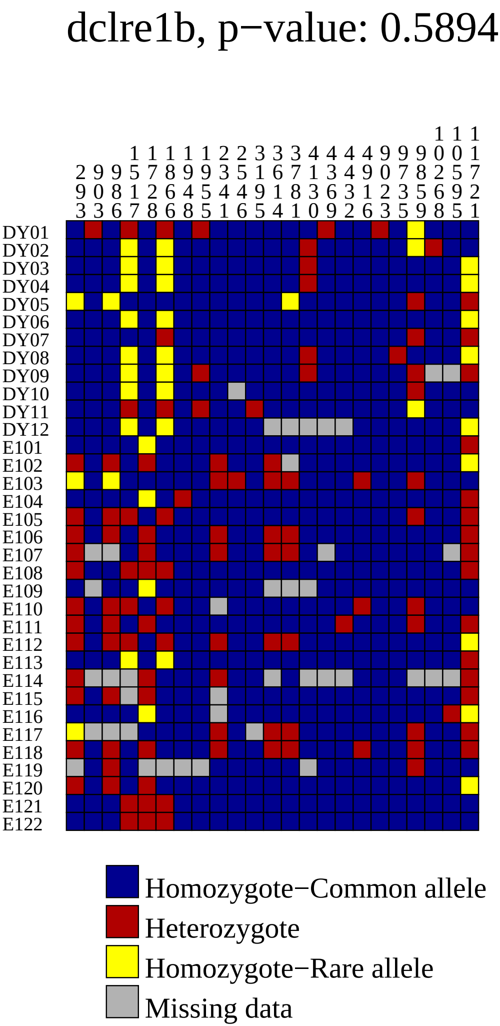
dclre1b, p−value: 0.5894
293 903 986 1517 1728 1866 1948 1955 2341 2546 3195 3614 3781 4130 4369 4432 4916 9023 9735 9859 10268 10595 11721
DY01
DY02
DY03
DY04
DY05
DY06
DY07
DY08
DY09
DY10
DY11
DY12
E101
E102
E103
E104
E105
E106
E107
E108
E109
E110
E111
E112
E113
E114
E115
E116
E117
E118
E119
E120
E121
E122
Homozygote−Common allele
Heterozygote
Homozygote−Rare allele
Missing data

ddb1, p−value: 0.086

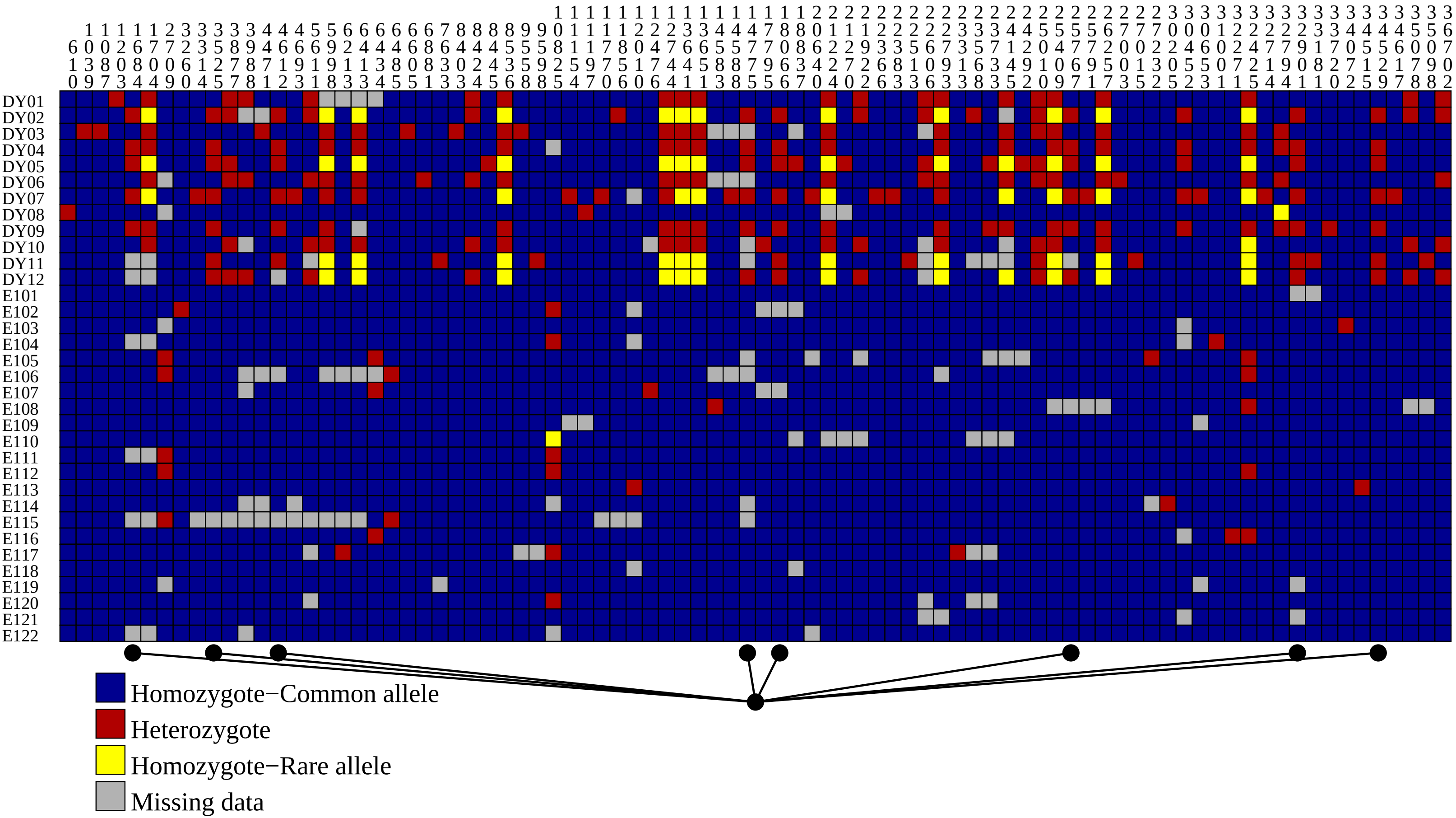

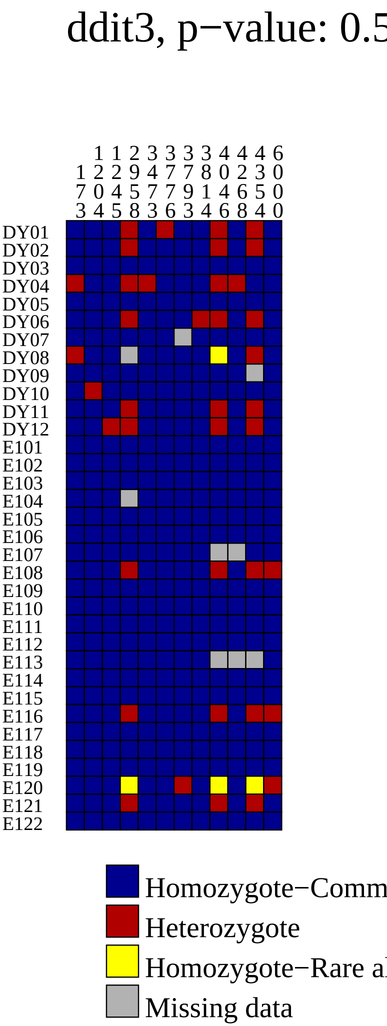
ddit3, p−value: 0.5
173
1204
1245
2958
3473
3776
3793
3814
4046
4268
4354
6000
DY01
DY02
DY03
DY04
DY05
DY06
DY07
DY08
DY09
DY10
DY11
DY12
E101
E102
E103
E104
E105
E106
E107
E108
E109
E110
E111
E112
E113
E114
E115
E116
E117
E118
E119
E120
E121
E122
Homozygote−Comm
Heterozygote
Homozygote−Rare a
Missing data

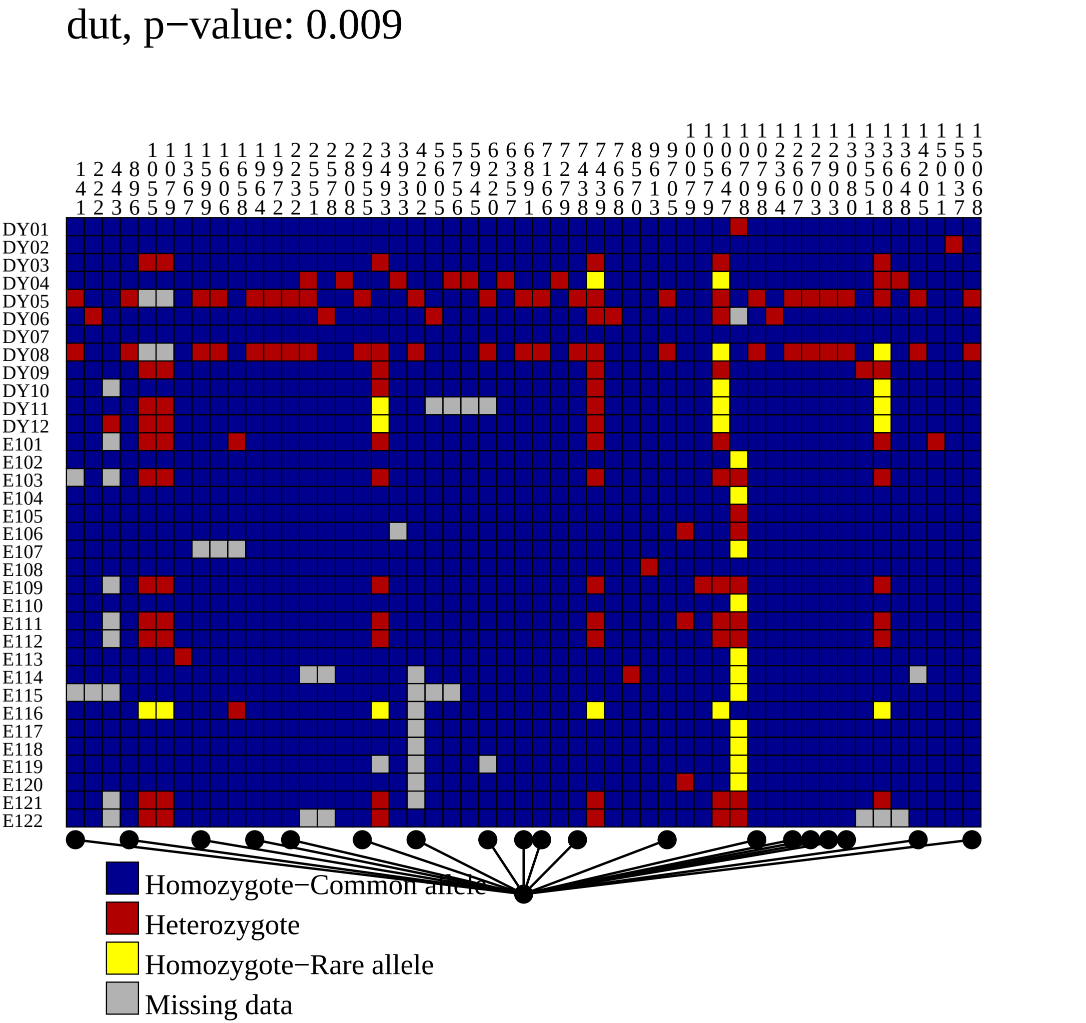
dut, p−value: 0.009
Homozygote−Common allele
Heterozygote
Homozygote−Rare allele
Missing data

ece1, p−value: 0.8506

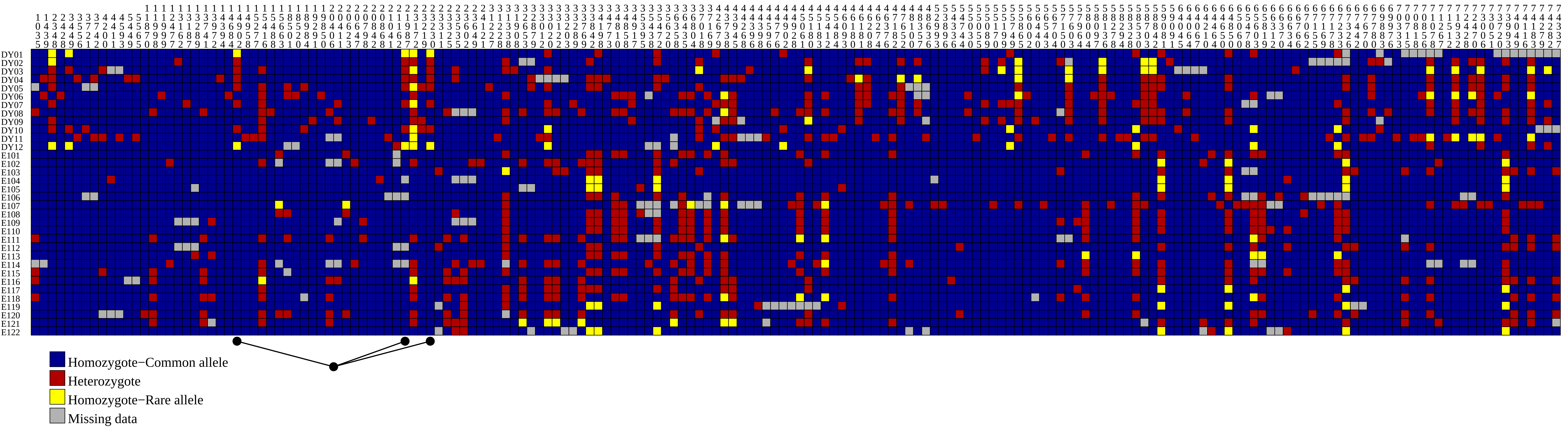

eno1, p−value: 0.0882

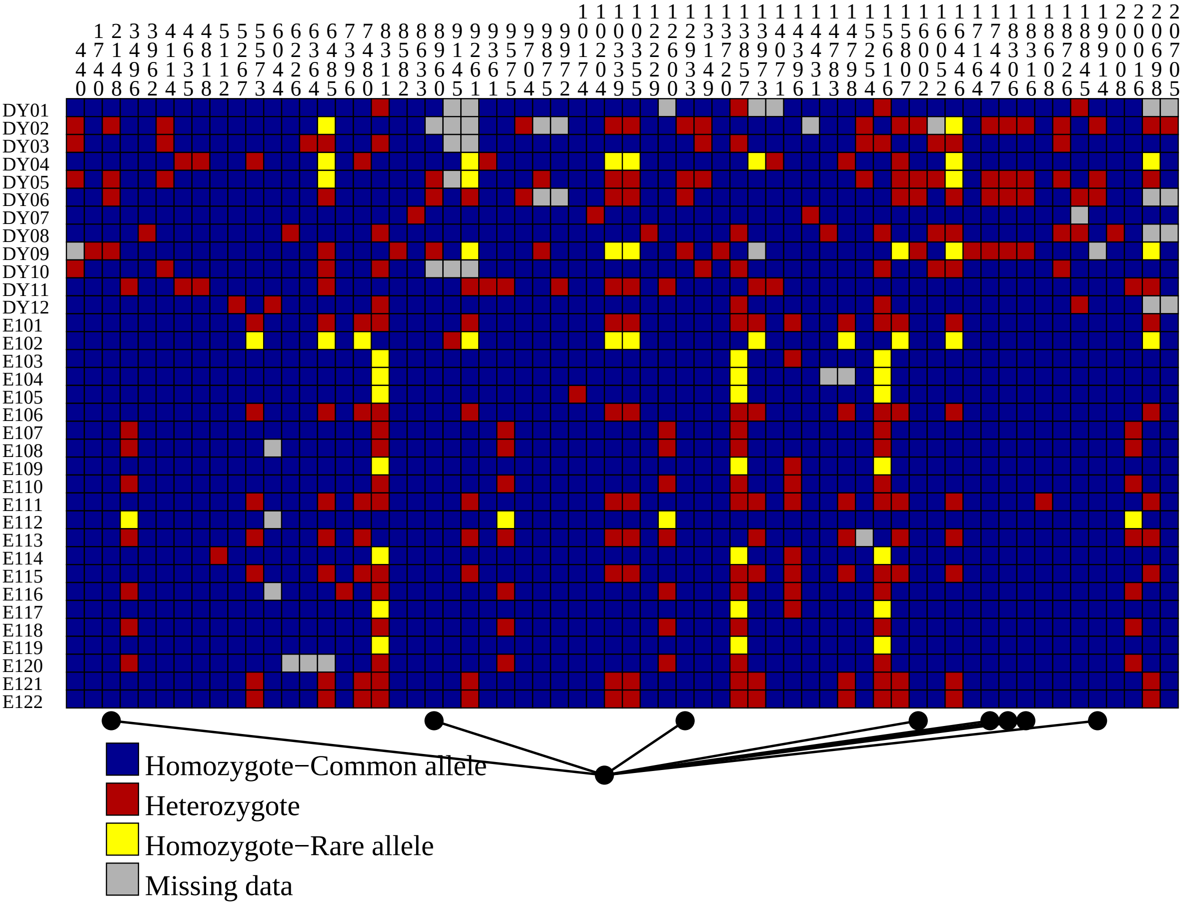

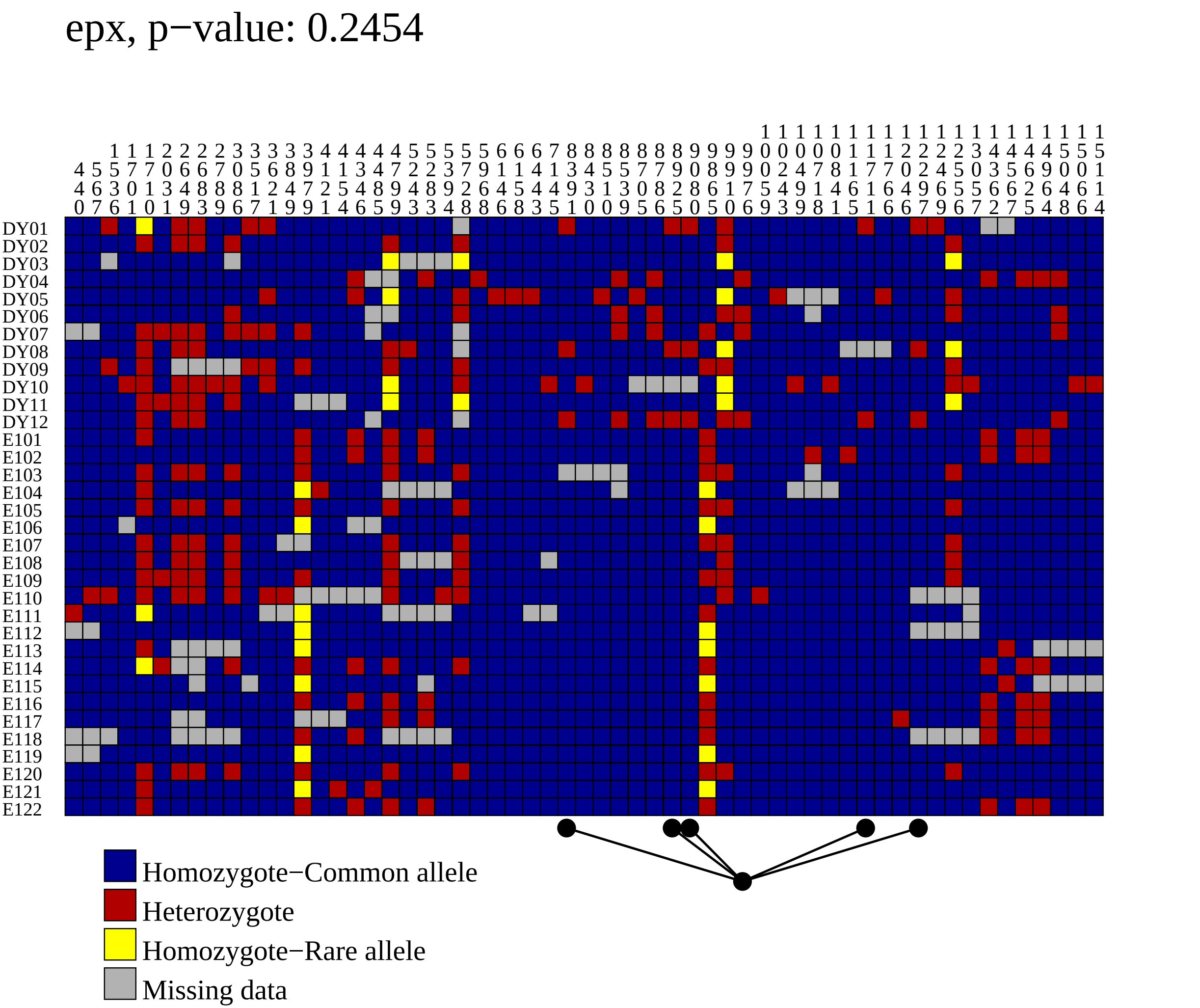
epx, p−value: 0.2454
440 567 1536 1701 1710 2031 2649 2683 2789 3086 3517 3621 3849 3979 4121 4154 4346 4485 4799 5243 5283 5394 5728 5968 6146 6158 6443 7145 8391 8430 8510 8539 8705 8786 8925 9080 9865 9910 9976 10059 10243 10499 10718 10841 11165 11711 11766 12046 12297 12469 12556 13057 14362 14567 14625 14964 15048 15066 15114
DY01
DY02
DY03
DY04
DY05
DY06
DY07
DY08
DY09
DY10
DY11
DY12
E101
E102
E103
E104
E105
E106
E107
E108
E109
E110
E111
E112
E113
E114
E115
E116
E117
E118
E119
E120
E121
E122
Homozygote−Common allele
Heterozygote
Homozygote−Rare allele
Missing data

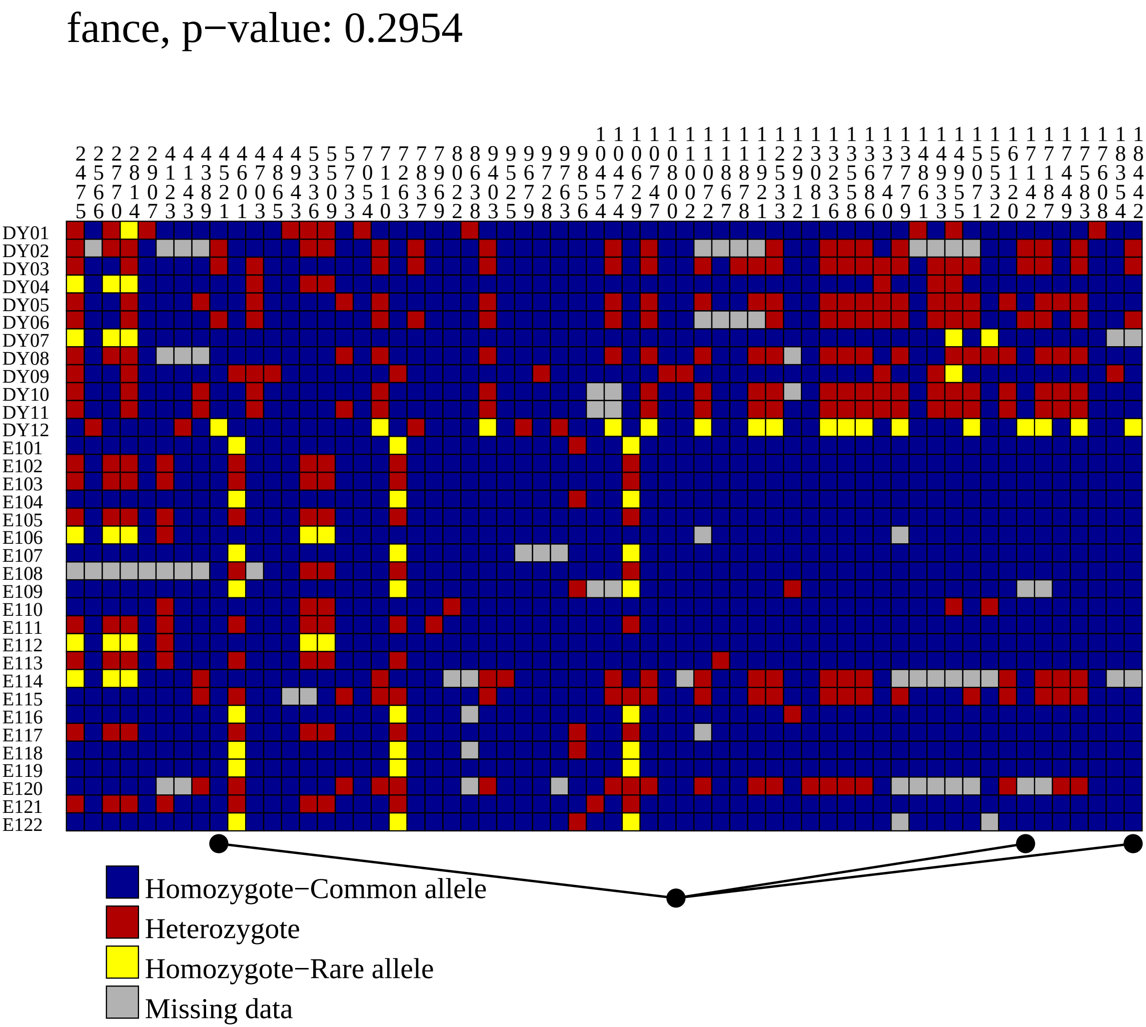
fance, p−value: 0.2954
Homozygote−Common allele
Heterozygote
Homozygote−Rare allele
Missing data

fancf, p−value: 0.0778

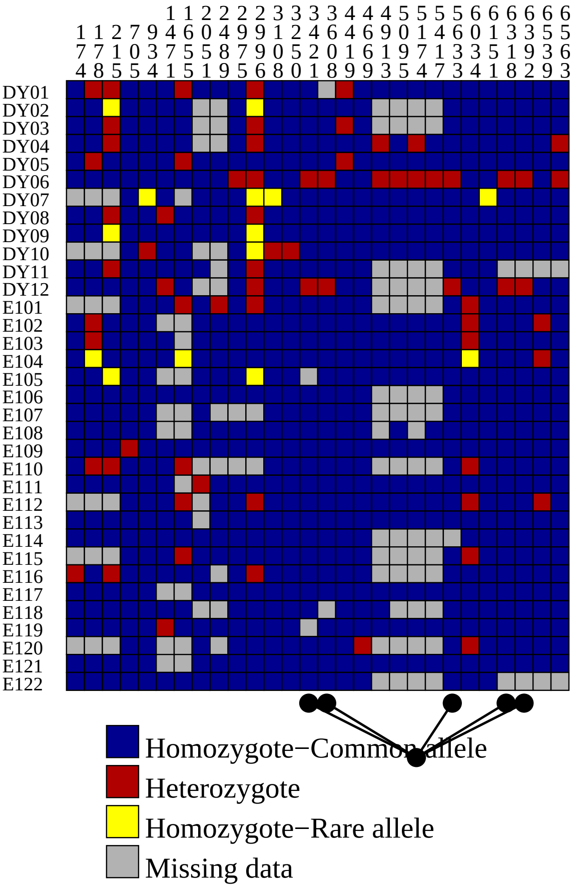

fbp1, p−value: 0.1952

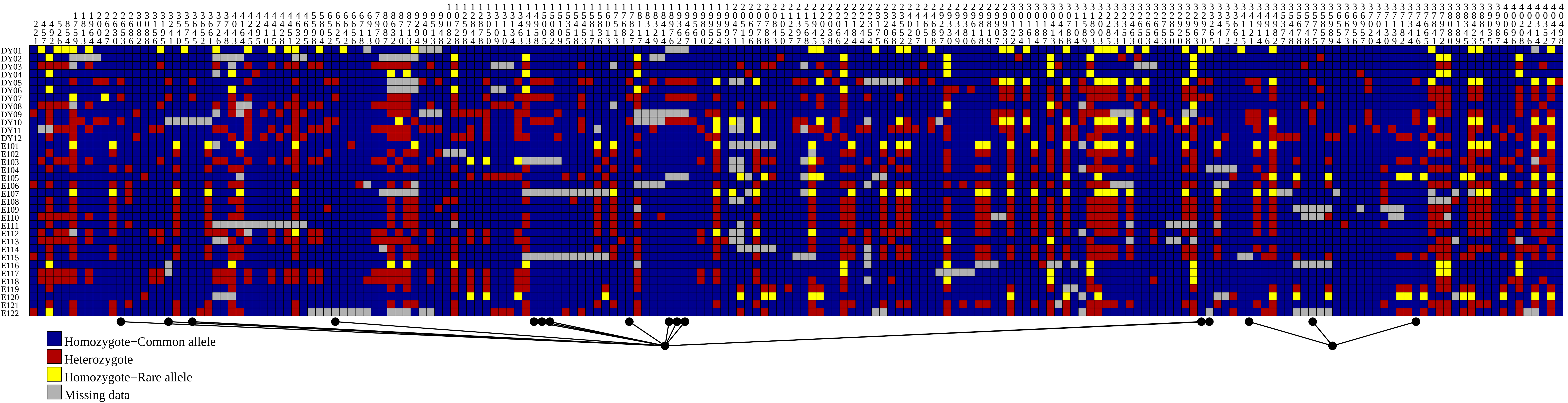

fdxr, p−value: 0.1546

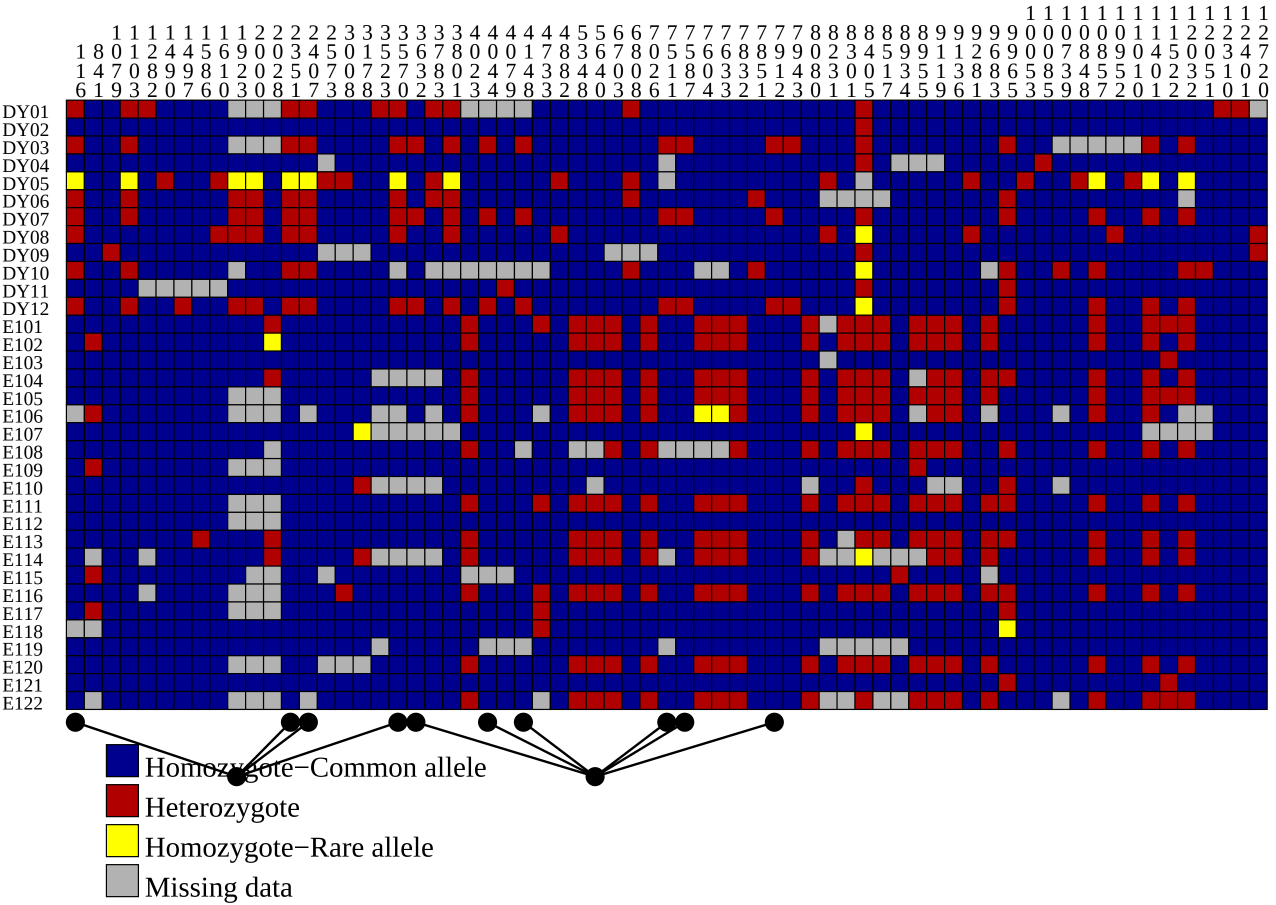

fgf5, p−value: 0.5746

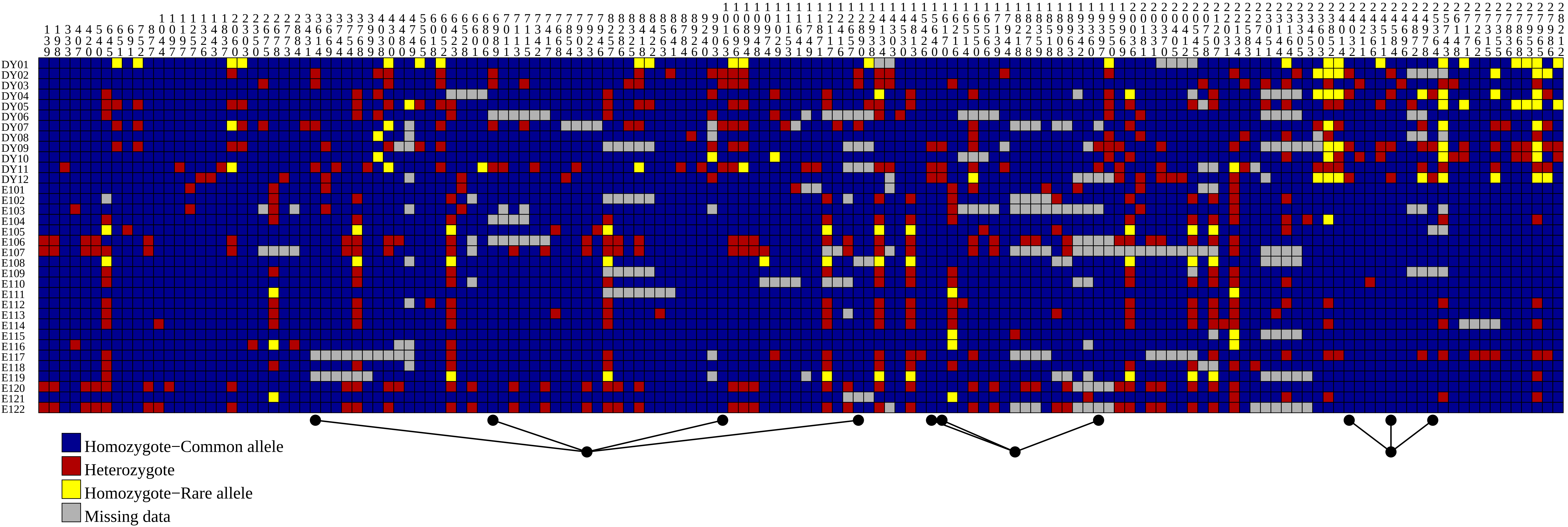

fmo2, p−value: 0.2464

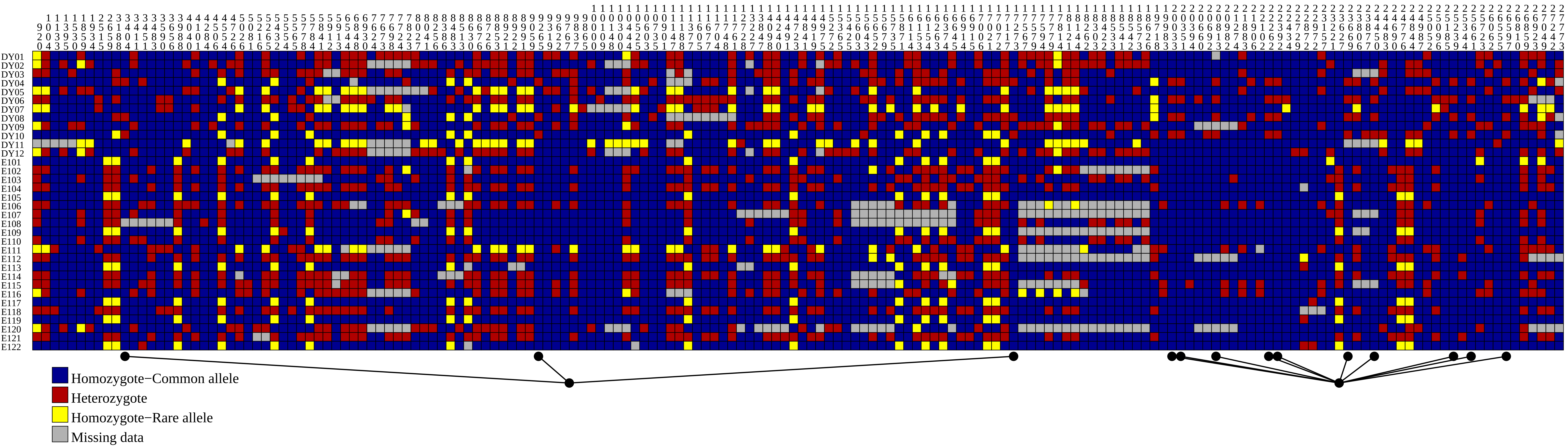

fmo3, p−value: 0.477

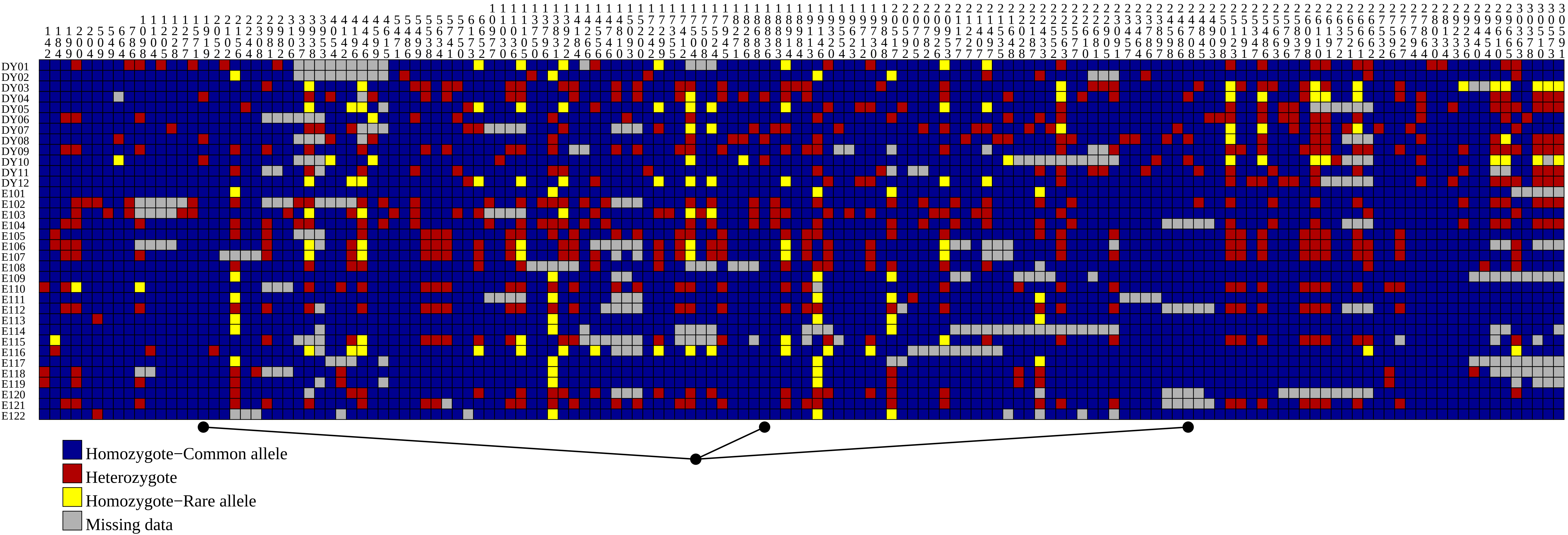

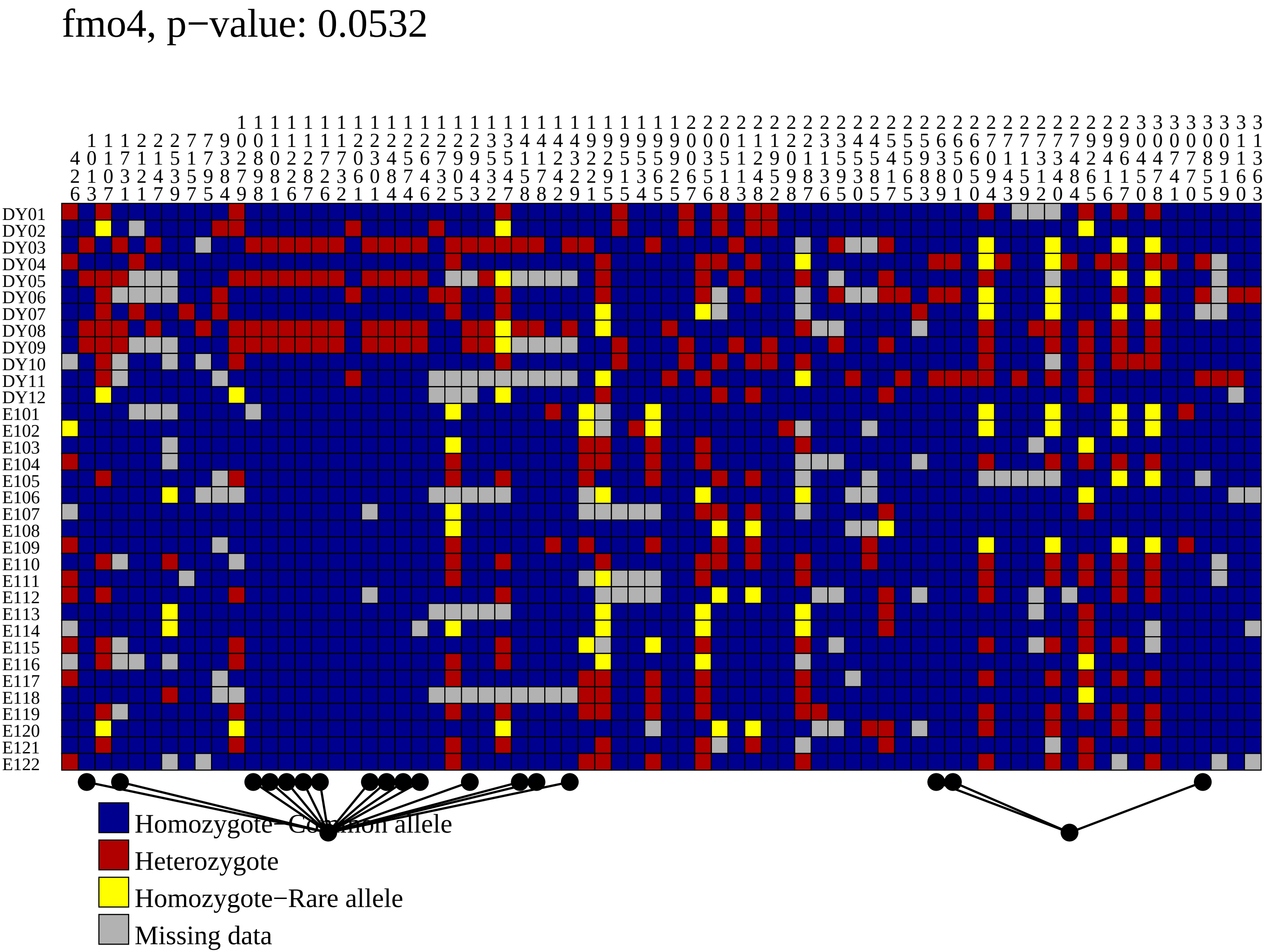

fmo4, p−value: 0.0532
DY01
DY02
DY03
DY04
DY05
DY06
DY07
DY08
DY09
DY10
DY11
DY12
E101
E102
E103
E104
E105
E106
E107
E108
E109
E110
E111
E112
E113
E114
E115
E116
E117
E118
E119
E120
E121
E122
Homozygote−Common allele
Heterozygote
Homozygote−Rare allele
Missing data

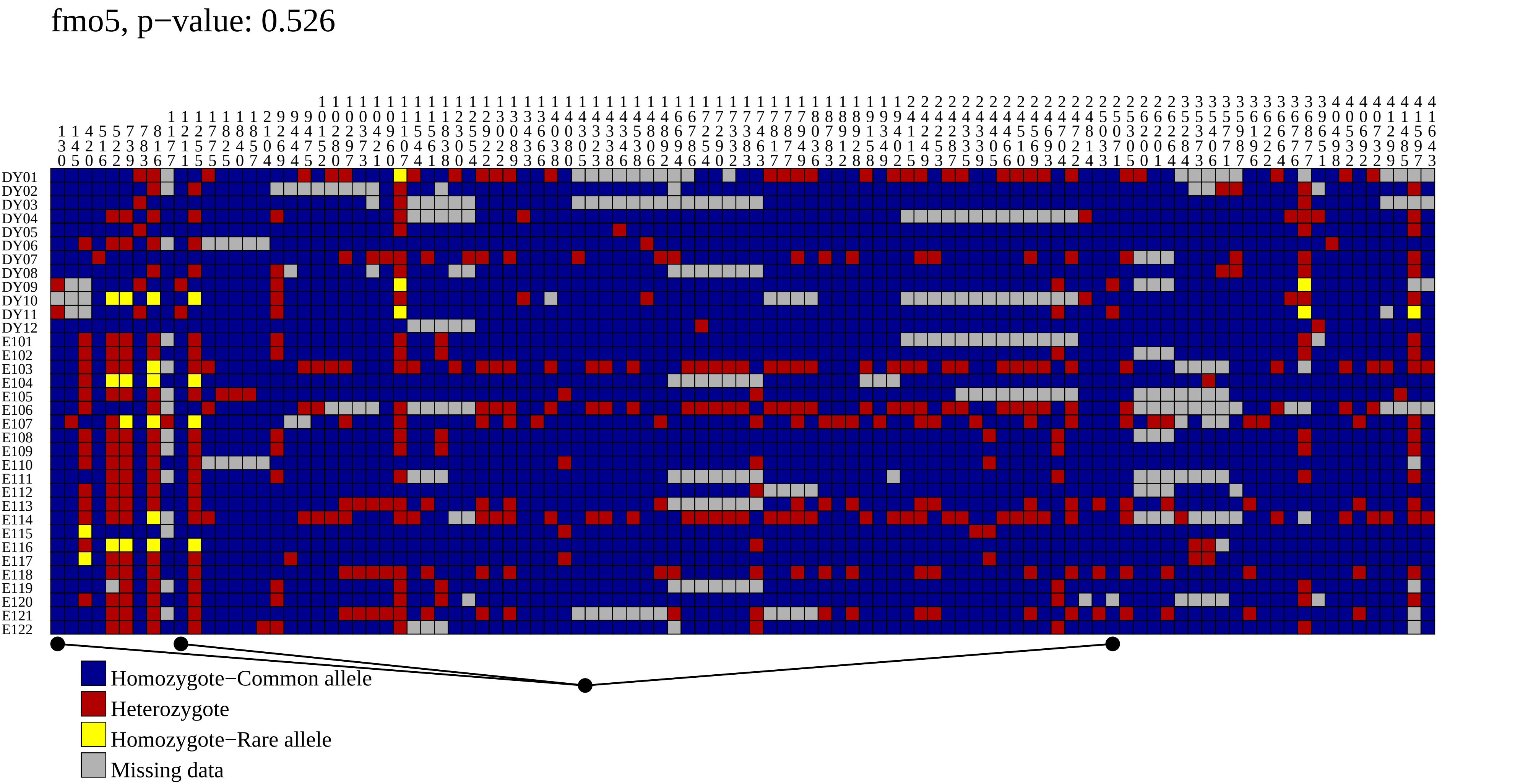
fmo5, p−value: 0.526
DY01
DY02
DY03
DY04
DY05
DY06
DY07
DY08
DY09
DY10
DY11
DY12
E101
E102
E103
E104
E105
E106
E107
E108
E109
E110
E111
E112
E113
E114
E115
E116
E117
E118
E119
E120
E121
E122
Homozygote−Common allele
Heterozygote
Homozygote−Rare allele
Missing data

fosb, p−value: 0.2166

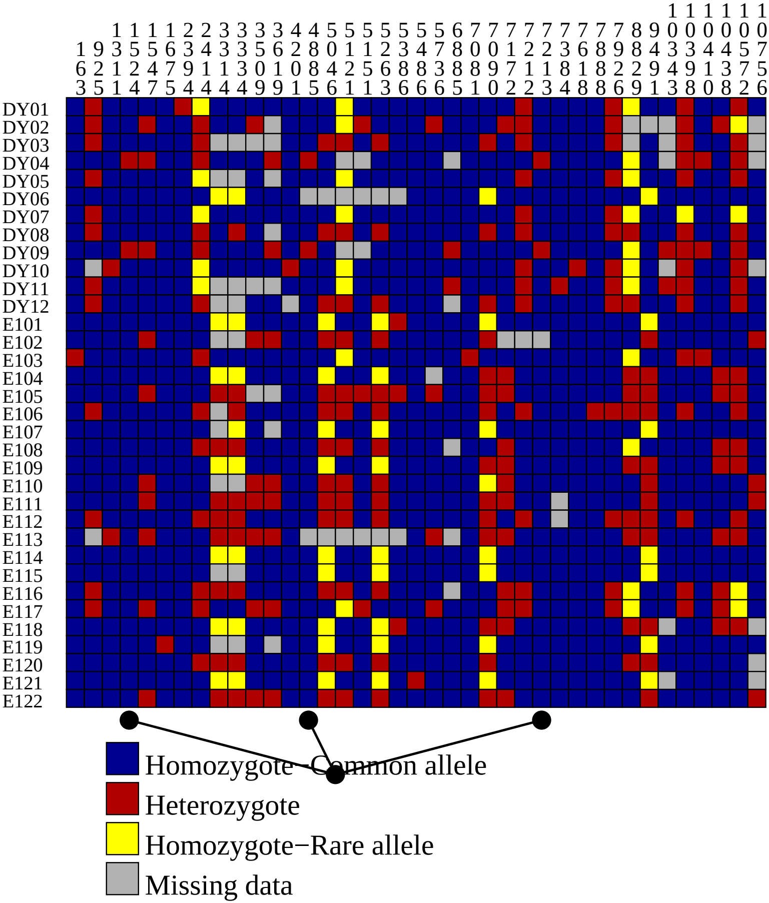

foxm1, p−value: 0.0746

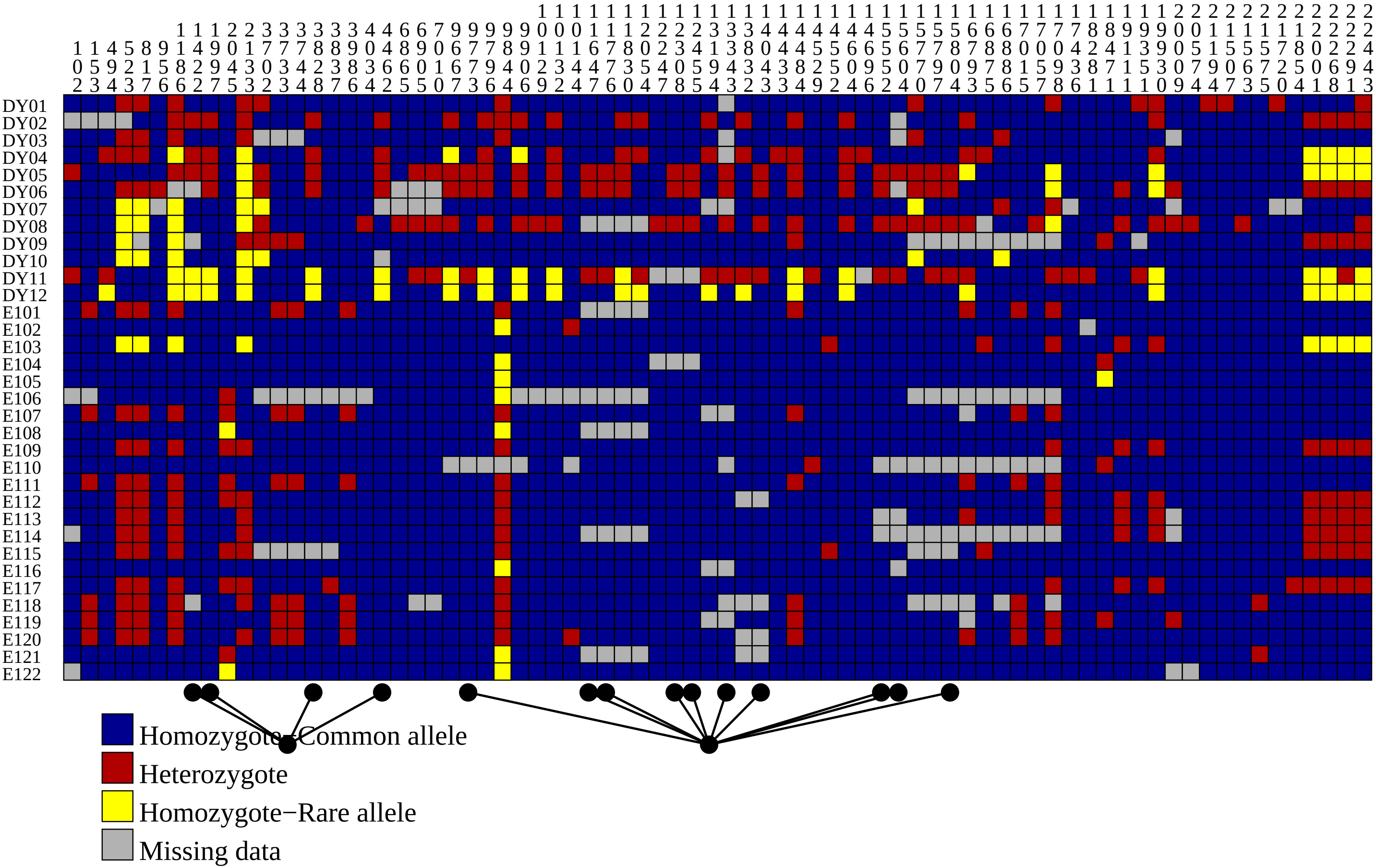

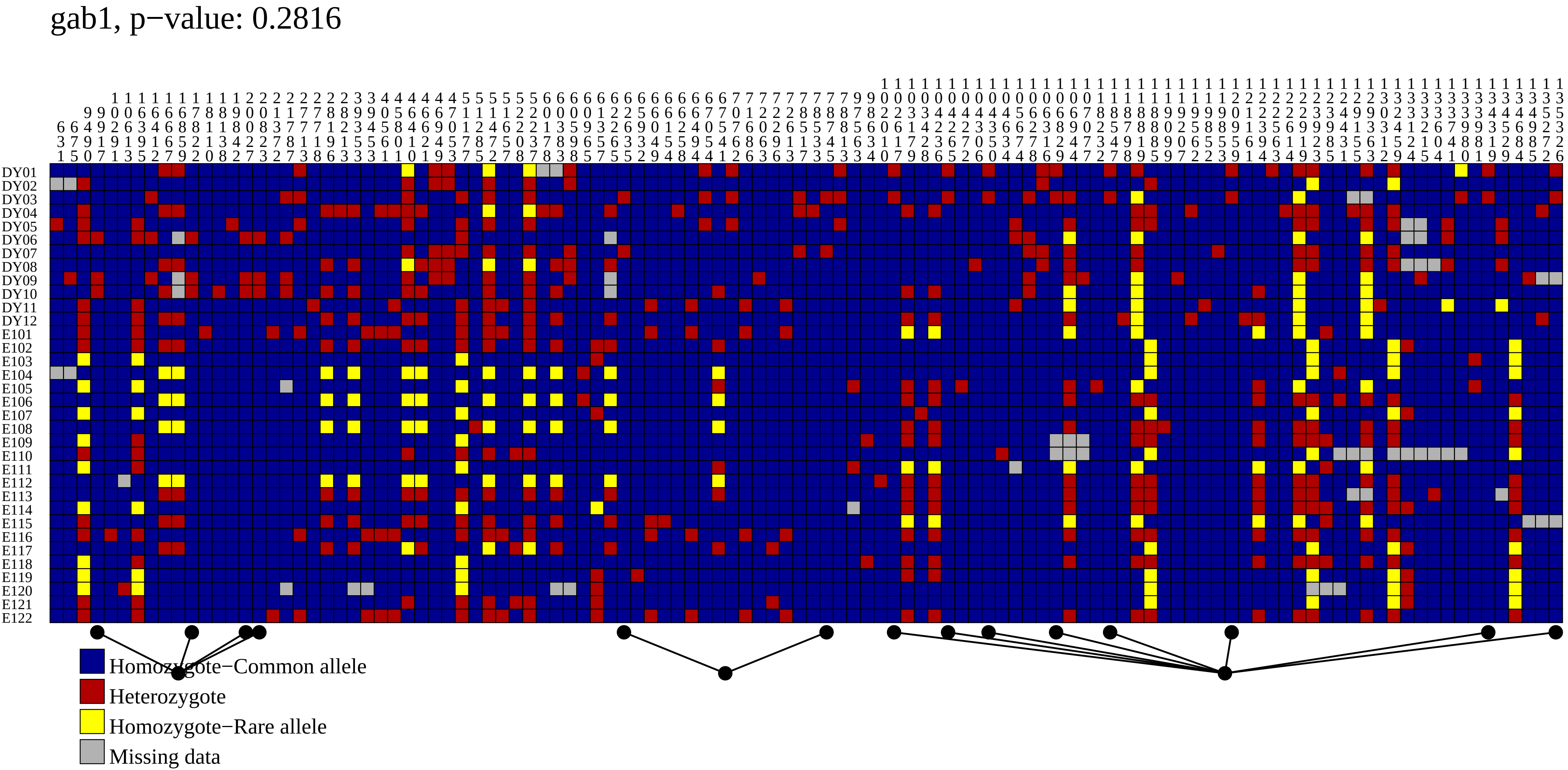

gab1, p−value: 0.2816
DY01
DY02
DY03
DY04
DY05
DY06
DY07
DY08
DY09
DY10
DY11
DY12
E101
E102
E103
E104
E105
E106
E107
E108
E109
E110
E111
E112
E113
E114
E115
E116
E117
E118
E119
E120
E121
E122
Homozygote−Common allele
Heterozygote
Homozygote−Rare allele
Missing data

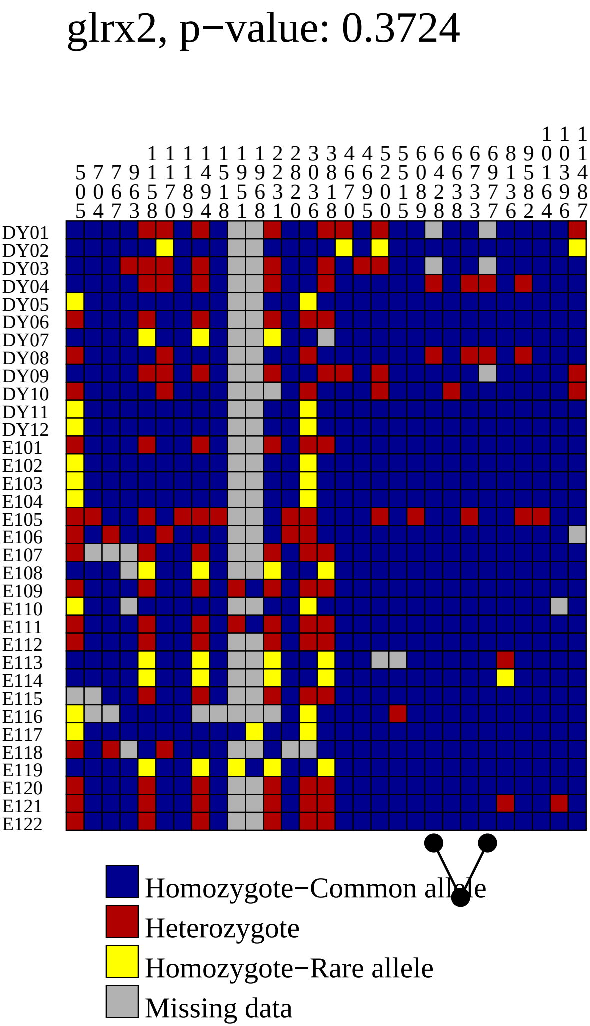

glrx2, p−value: 0.3724
505 704 767 963 1158 1170 1189 1494 1518 1951 1968 2231 2820 3036 3818 4670 4695 5200 5015 6089 6428 6638 6733 6977 8136 9582 10164 10396 11487
DY01 DY02 DY03 DY04 DY05 DY06 DY07 DY08 DY09 DY10 DY11 DY12
E101 E102 E103 E104 E105 E106 E107 E108 E109 E110 E111 E112 E113 E114 E115 E116 E117 E118 E119 E120 E121 E122
Homozygote−Common allele
Heterozygote
Homozygote−Rare allele
Missing data

glrx, p−value: 0.468

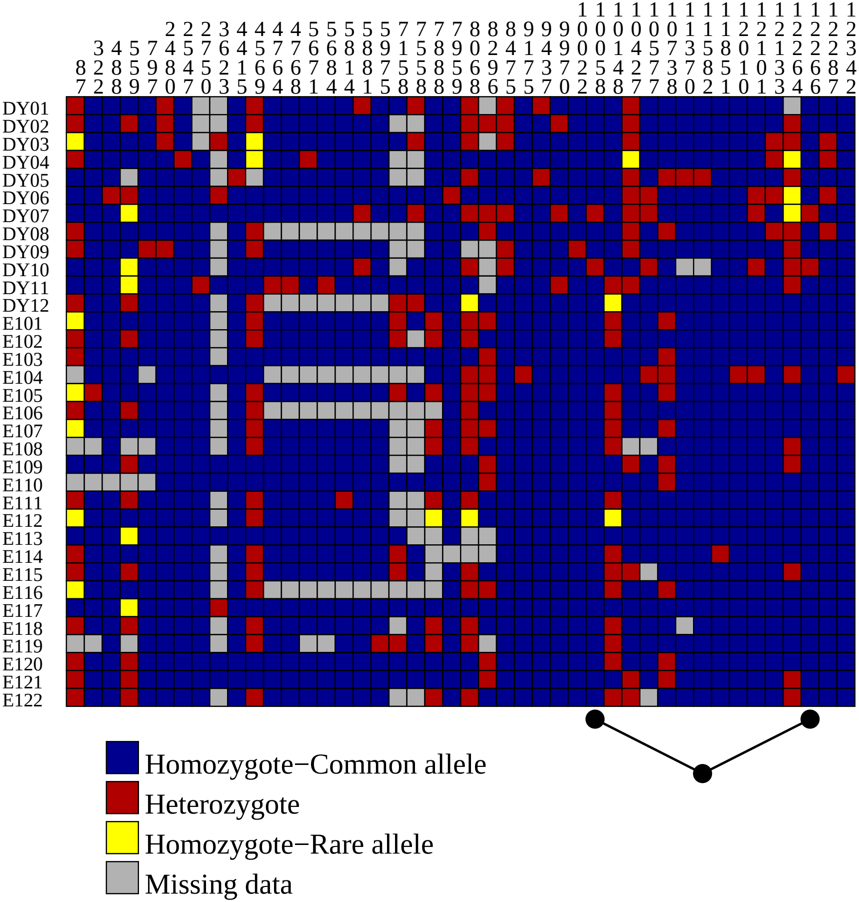

gpx5, p−value: 0.2684

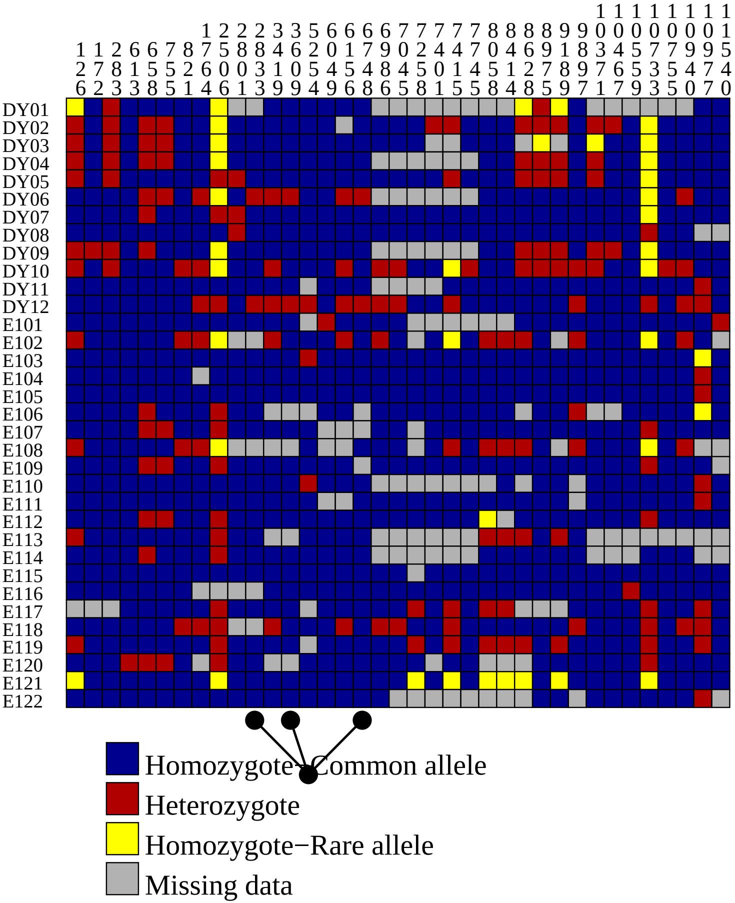

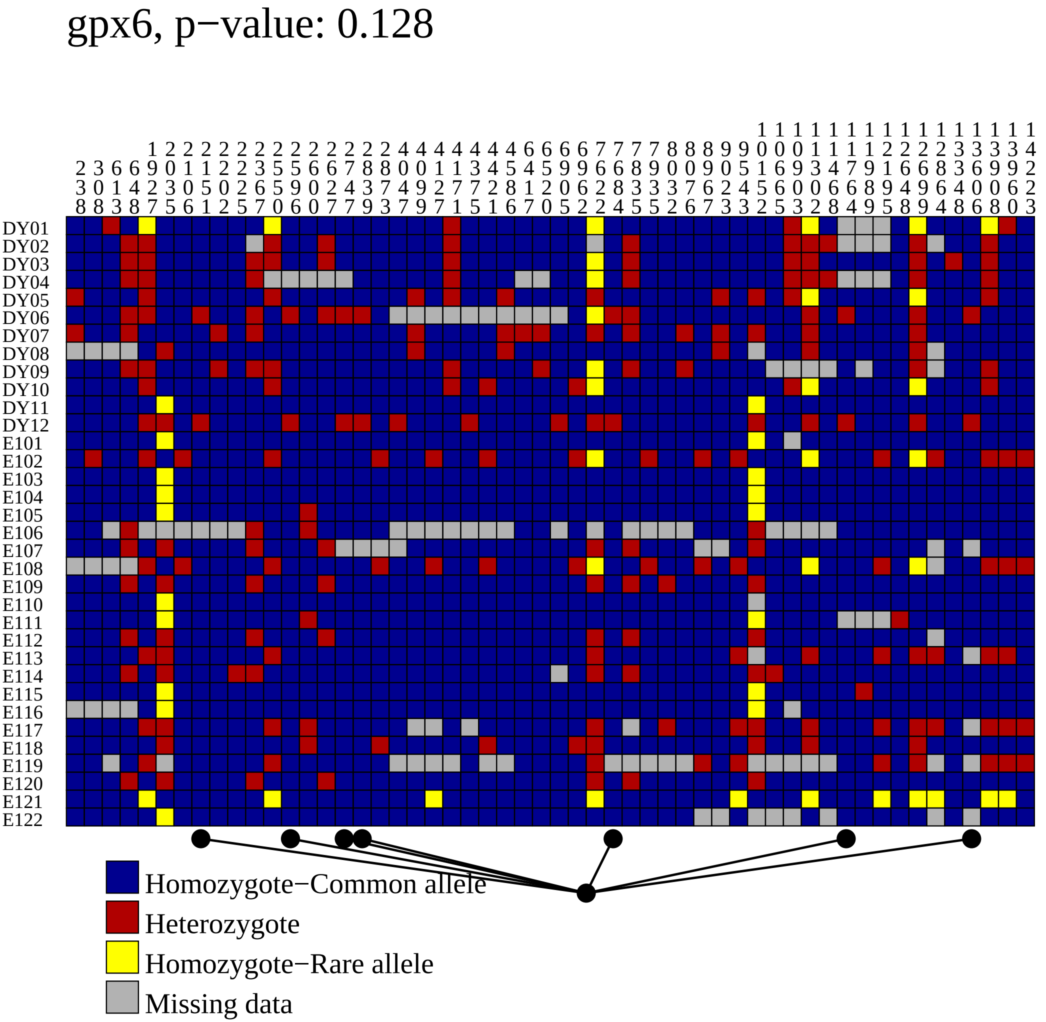
gpx6, p−value: 0.128
238 308 613 648 1927 2035 2106 2151 2202 2225 2367 2550 2596 2600 2627 2747 2839 2873 4047 4099 4127 4171 4375 4421 4586 6417 6520 6905 6962 7622 7684 7835 7935 8032 8076 8967 9023 9543 10152 10665 10903 11302 11468 11764 11989 12195 12648 12699 12864 13348 13606 13908 13960 14223
DY01
DY02
DY03
DY04
DY05
DY06
DY07
DY08
DY09
DY10
DY11
DY12
E101
E102
E103
E104
E105
E106
E107
E108
E109
E110
E111
E112
E113
E114
E115
E116
E117
E118
E119
E120
E121
E122
Homozygote−Common allele
Heterozygote
Homozygote−Rare allele
Missing data

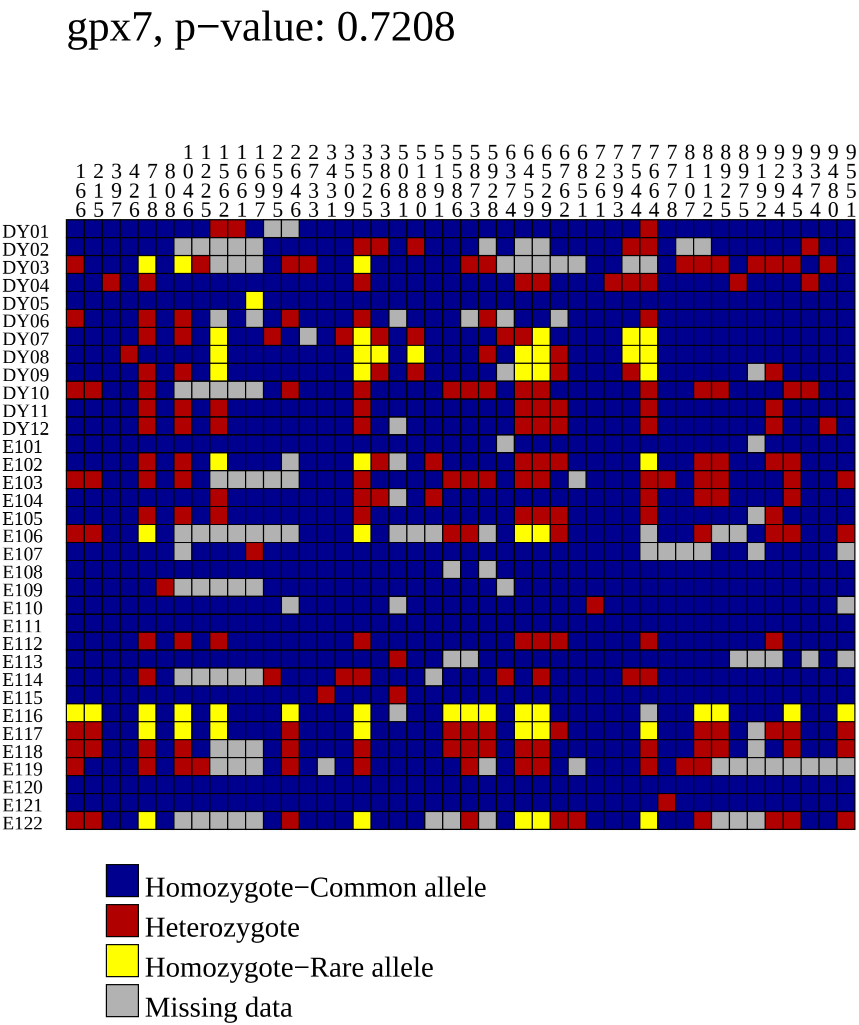
gpx7, p−value: 0.7208
166 215 397 426 718 808 1046 1225 1562 1661 1697 2595 2646 2733 3431 3509 3525 3863 5081 5180 5191 5586 5873 5928 6374 6459 6529 6762 6851 7261 7393 7544 7664 7778 8107 8112 8925 8975 9192 9294 9345 9374 9480 9551
DY01 DY02 DY03 DY04 DY05 DY06 DY07 DY08 DY09 DY10 DY11 DY12 E101 E102 E103 E104 E105 E106 E107 E108 E109 E110 E111 E112 E113 E114 E115 E116 E117 E118 E119 E120 E121 E122
Homozygote−Common allele
Heterozygote
Homozygote−Rare allele
Missing data

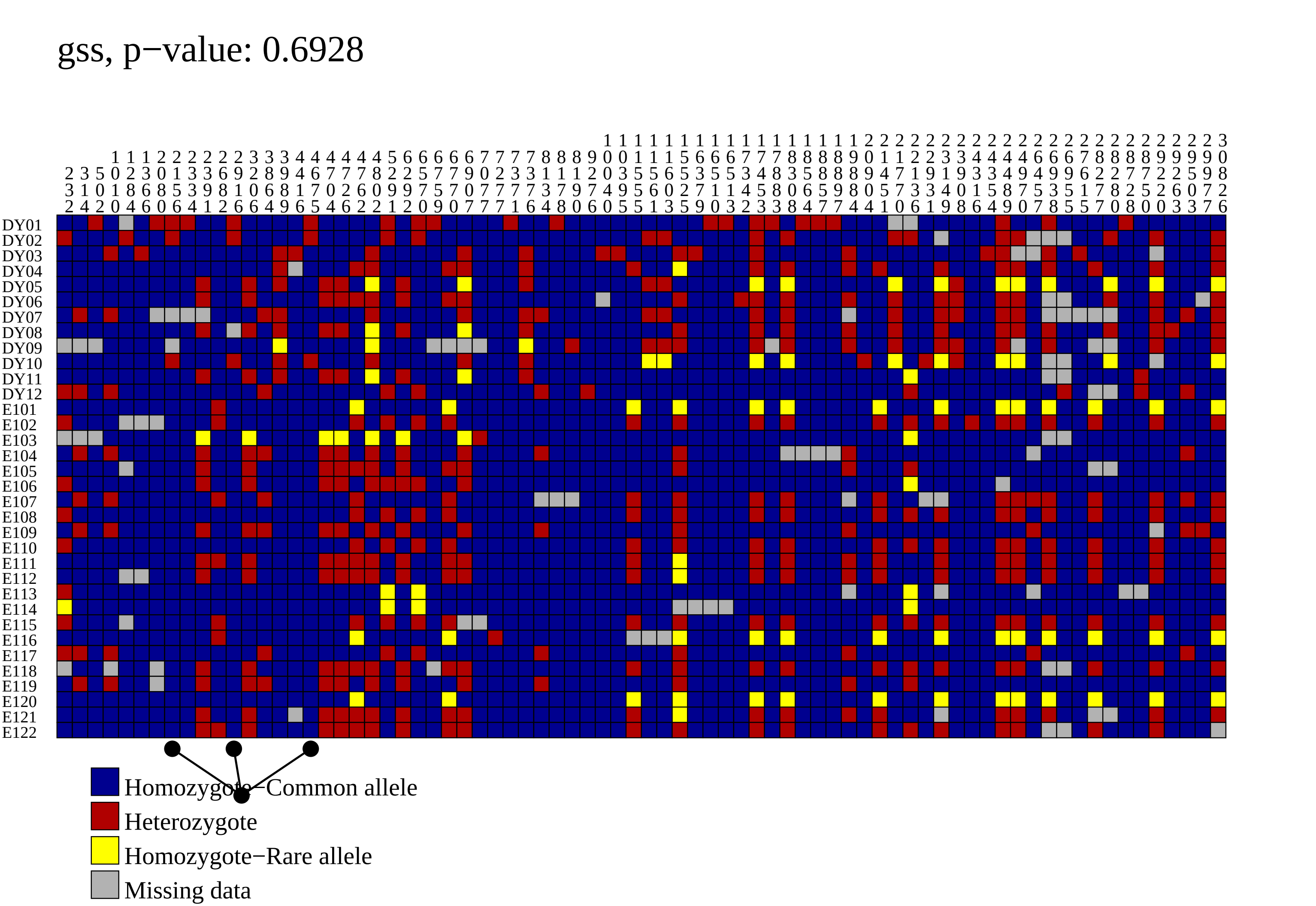
gss, p−value: 0.6928
232 314 502 1010 1284 1366 2080 2156 2334 2391 2682 2916 3206 3864 3989 4416 4675 4704 4726 4762 4802 5291 6292 6570 6759 6770 6907 7077 7277 7371 7376 8134 8178 8190 9276 10040 10395 11555 11561 11603 15525 16379 16510 16513 17342 17453 17883 18308 18564 18857 18897 19884 20904 21451 21770 22136 22931 23149 23908 24316 24354 24489 24970 26457 26938 26955 27615 28277 28280 28728 28750 29220 29263 29503 29977 30826
DY01
DY02
DY03
DY04
DY05
DY06
DY07
DY08
DY09
DY10
DY11
DY12
E101
E102
E103
E104
E105
E106
E107
E108
E109
E110
E111
E112
E113
E114
E115
E116
E117
E118
E119
E120
E121
E122
Homozygote−Common allele
Heterozygote
Homozygote−Rare allele
Missing data

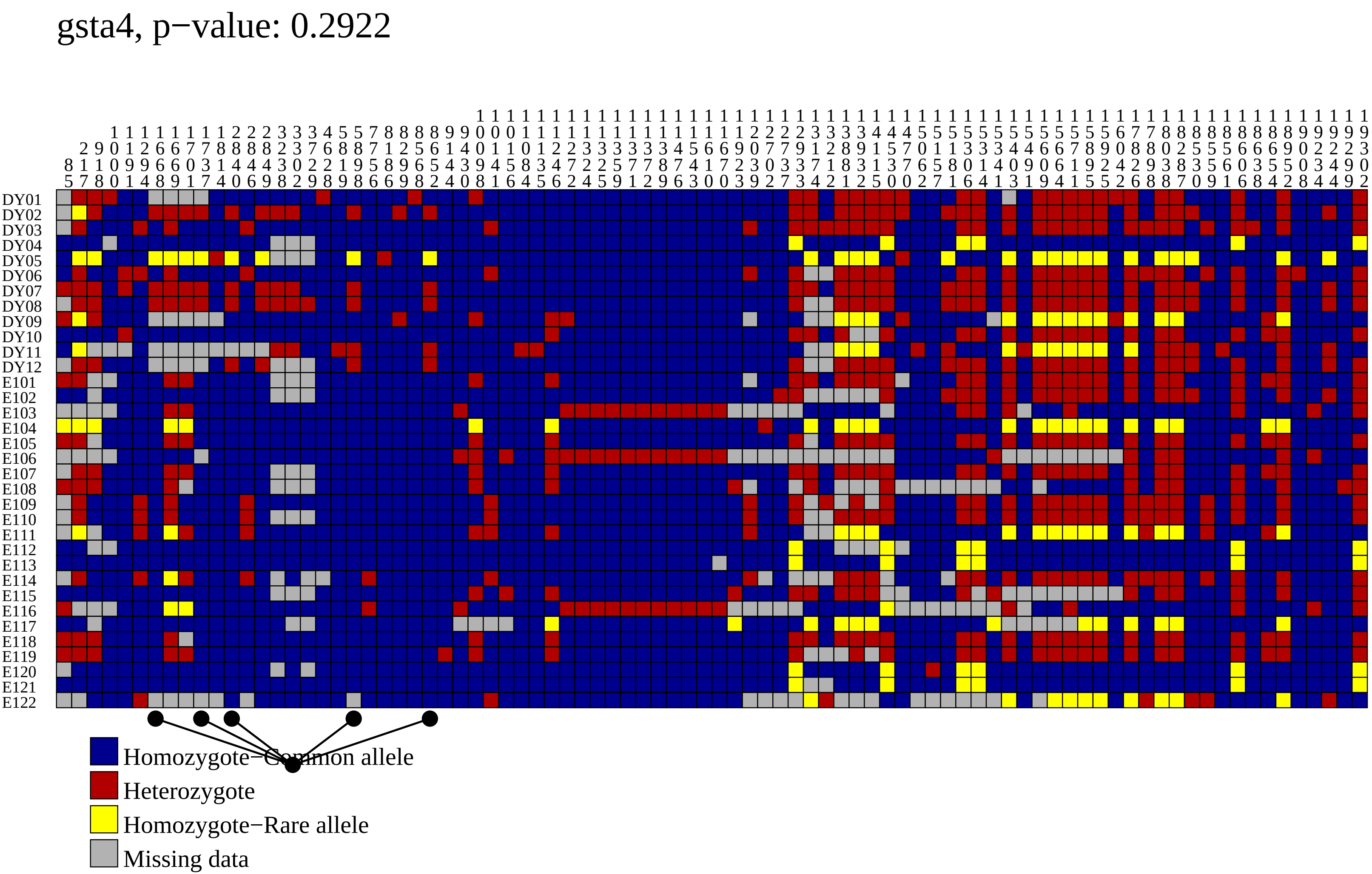
gsta4, p−value: 0.2922
Homozygote−Common allele
Heterozygote
Homozygote−Rare allele
Missing data

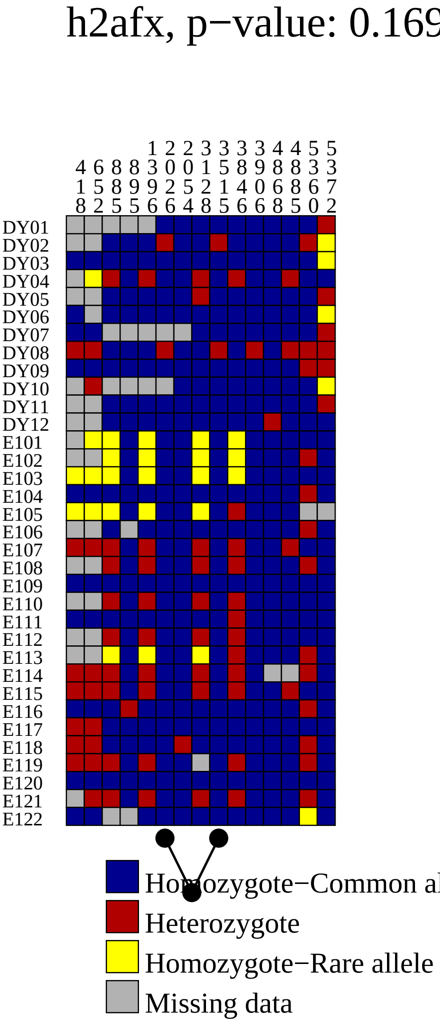

h2afx, p−value: 0.169
418
652
885
895
1396
2026
2054
3128
3515
3846
3906
4868
4885
5360
5372
DY01
DY02
DY03
DY04
DY05
DY06
DY07
DY08
DY09
DY10
DY11
DY12
E101
E102
E103
E104
E105
E106
E107
E108
E109
E110
E111
E112
E113
E114
E115
E116
E117
E118
E119
E120
E121
E122
Homozygote−Common al
Heterozygote
Homozygote−Rare allele
Missing data

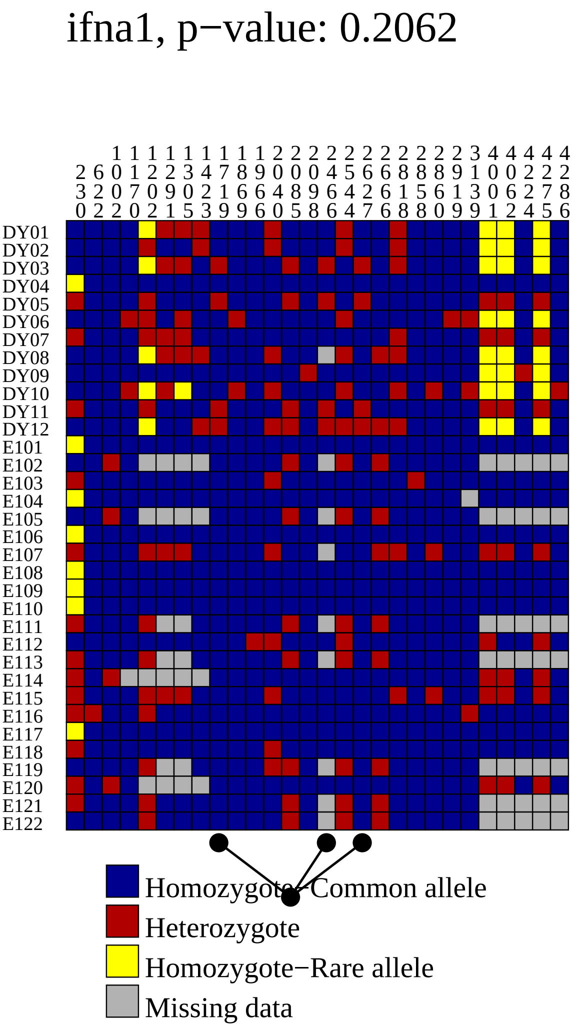
ifna1, p−value: 0.2062
230
602
1002
1170
1202
1291
1305
1423
1719
1869
1966
2040
2085
2098
2466
2544
2627
2666
2818
2858
2860
2919
3139
4001
4062
4224
4275
4286
DY01
DY02
DY03
DY04
DY05
DY06
DY07
DY08
DY09
DY10
DY11
DY12
E101
E102
E103
E104
E105
E106
E107
E108
E109
E110
E111
E112
E113
E114
E115
E116
E117
E118
E119
E120
E121
E122
Homozygote−Common allele
Heterozygote
Homozygote−Rare allele
Missing data

itgal, p−value: 0.437

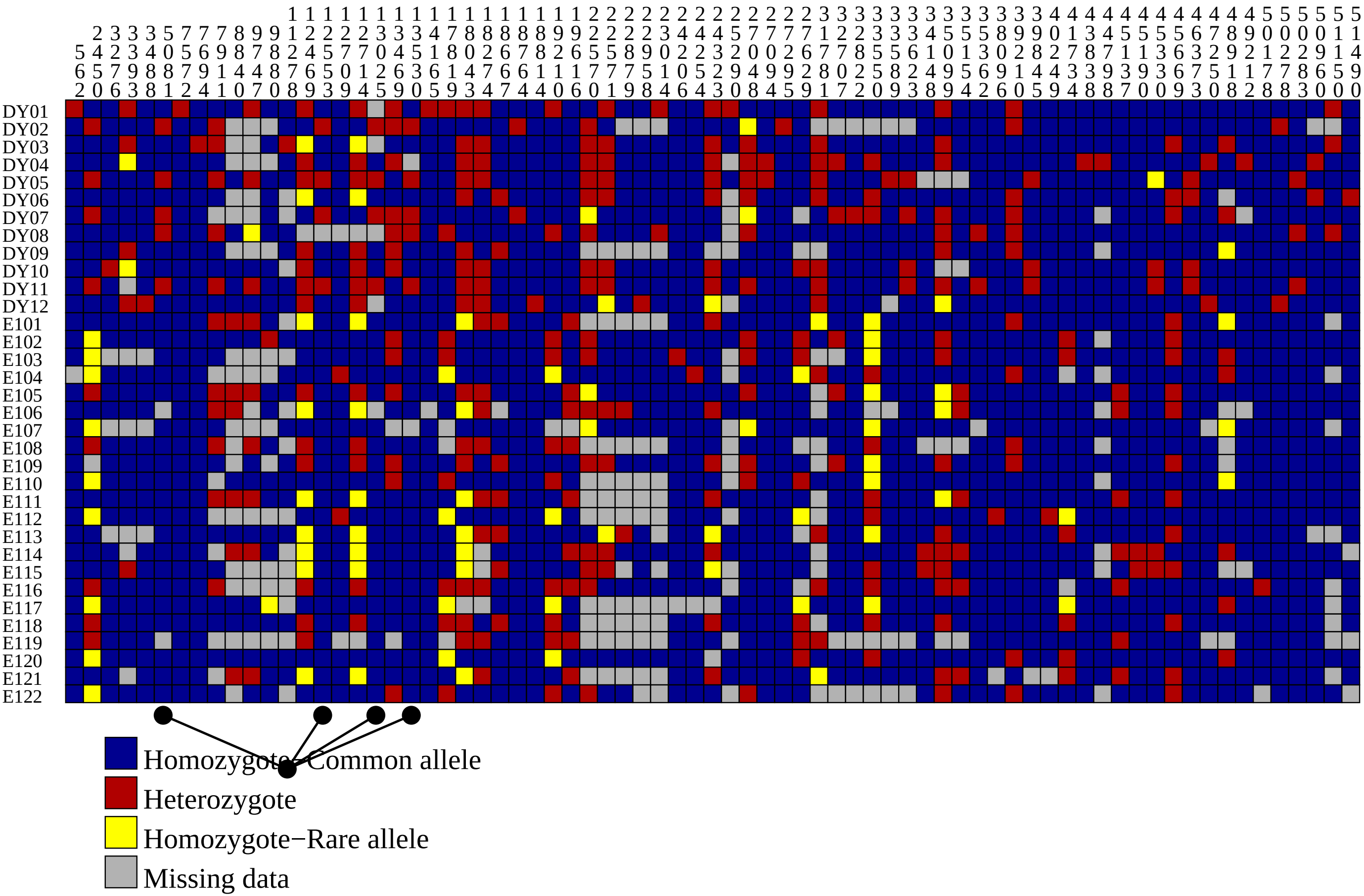

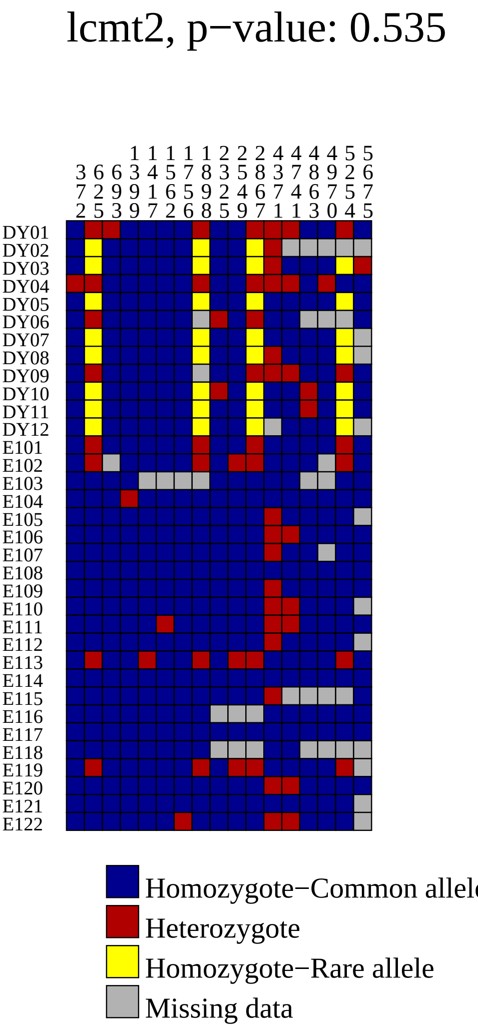
lcmt2, p−value: 0.535
372 625 693 1399 1417 1562 1756 1898 2325 2549 2867 4371 4741 4863 4970 5254 5675
DY01
DY02
DY03
DY04
DY05
DY06
DY07
DY08
DY09
DY10
DY11
DY12
E101
E102
E103
E104
E105
E106
E107
E108
E109
E110
E111
E112
E113
E114
E115
E116
E117
E118
E119
E120
E121
E122
Homozygote−Common allele
Heterozygote
Homozygote−Rare allele
Missing data

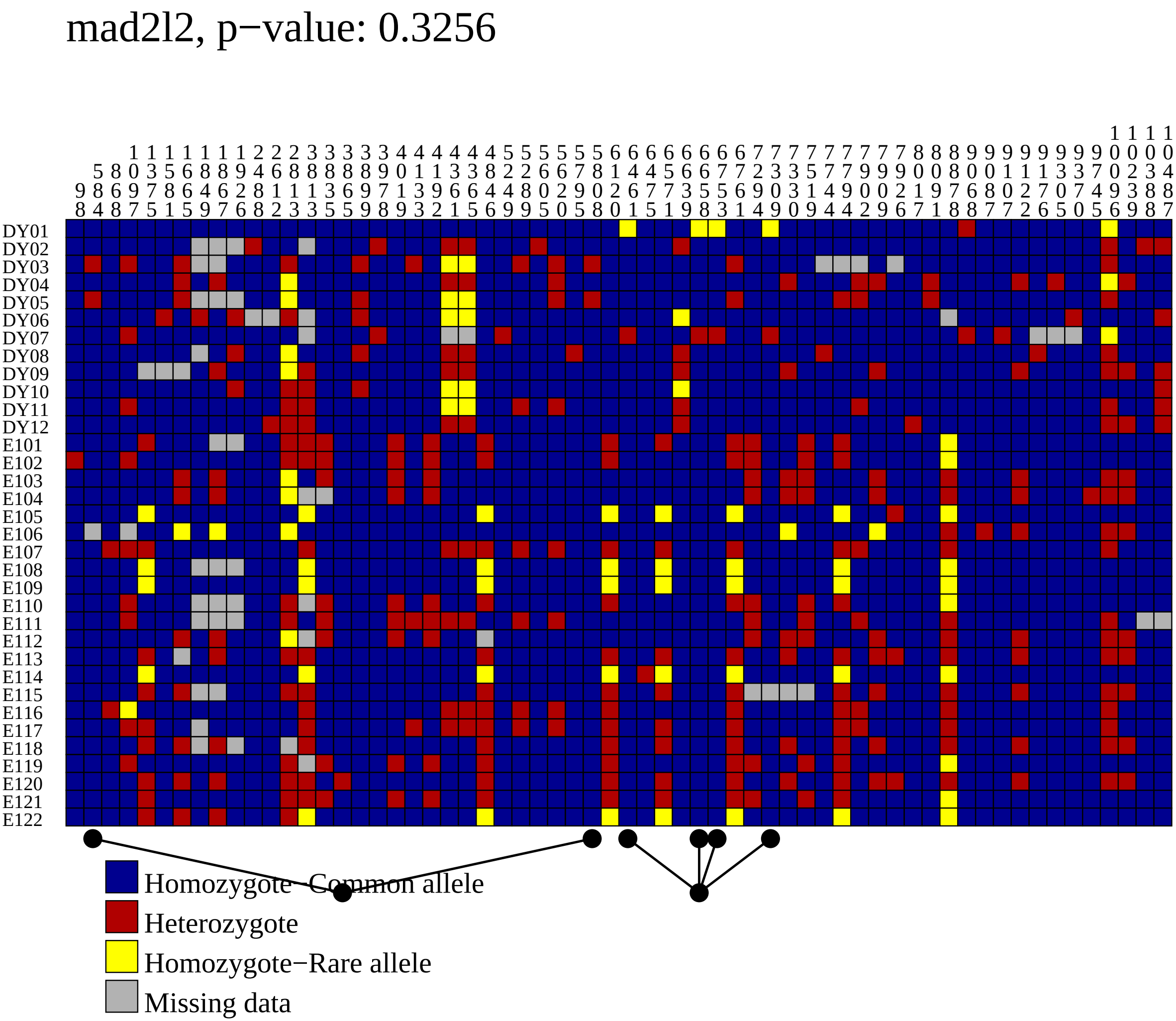

mad2l2, p−value: 0.3256
DY01
DY02
DY03
DY04
DY05
DY06
DY07
DY08
DY09
DY10
DY11
DY12
E101
E102
E103
E104
E105
E106
E107
E108
E109
E110
E111
E112
E113
E114
E115
E116
E117
E118
E119
E120
E121
E122
Homozygote−Common allele
Heterozygote
Homozygote−Rare allele
Missing data

map2k4, p−value: 0.0558

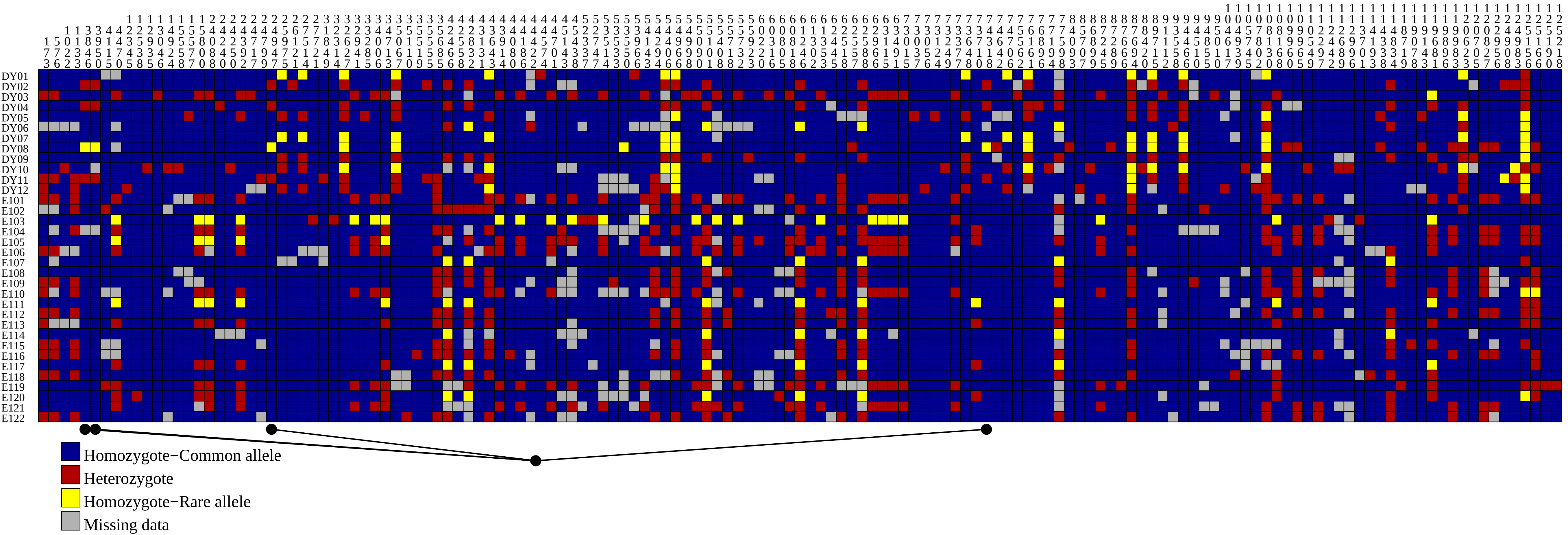

mapk9, p−value: 0.3838

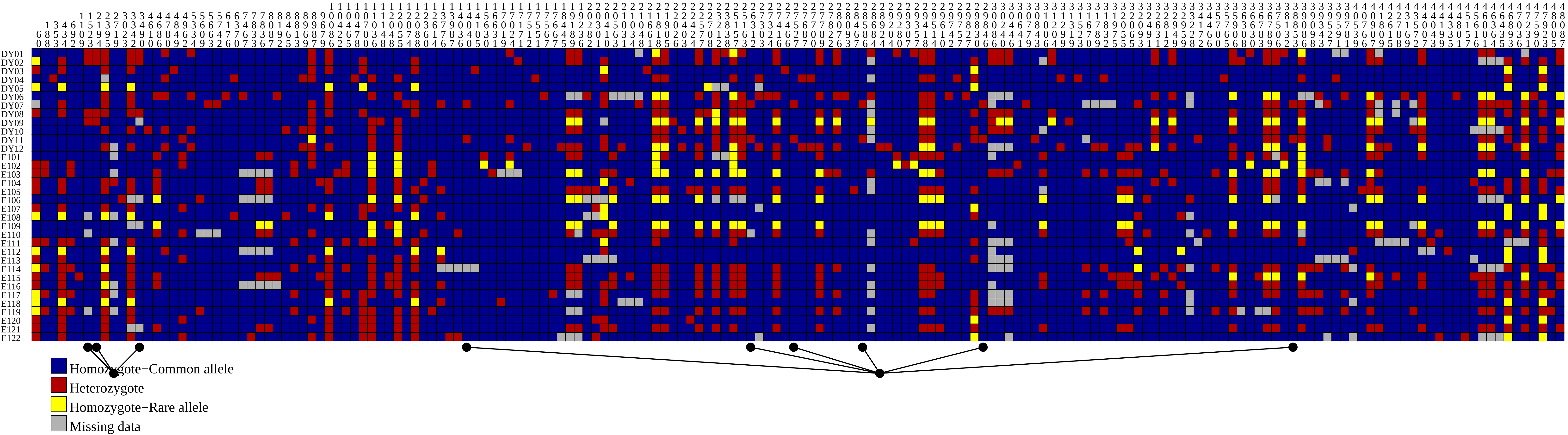

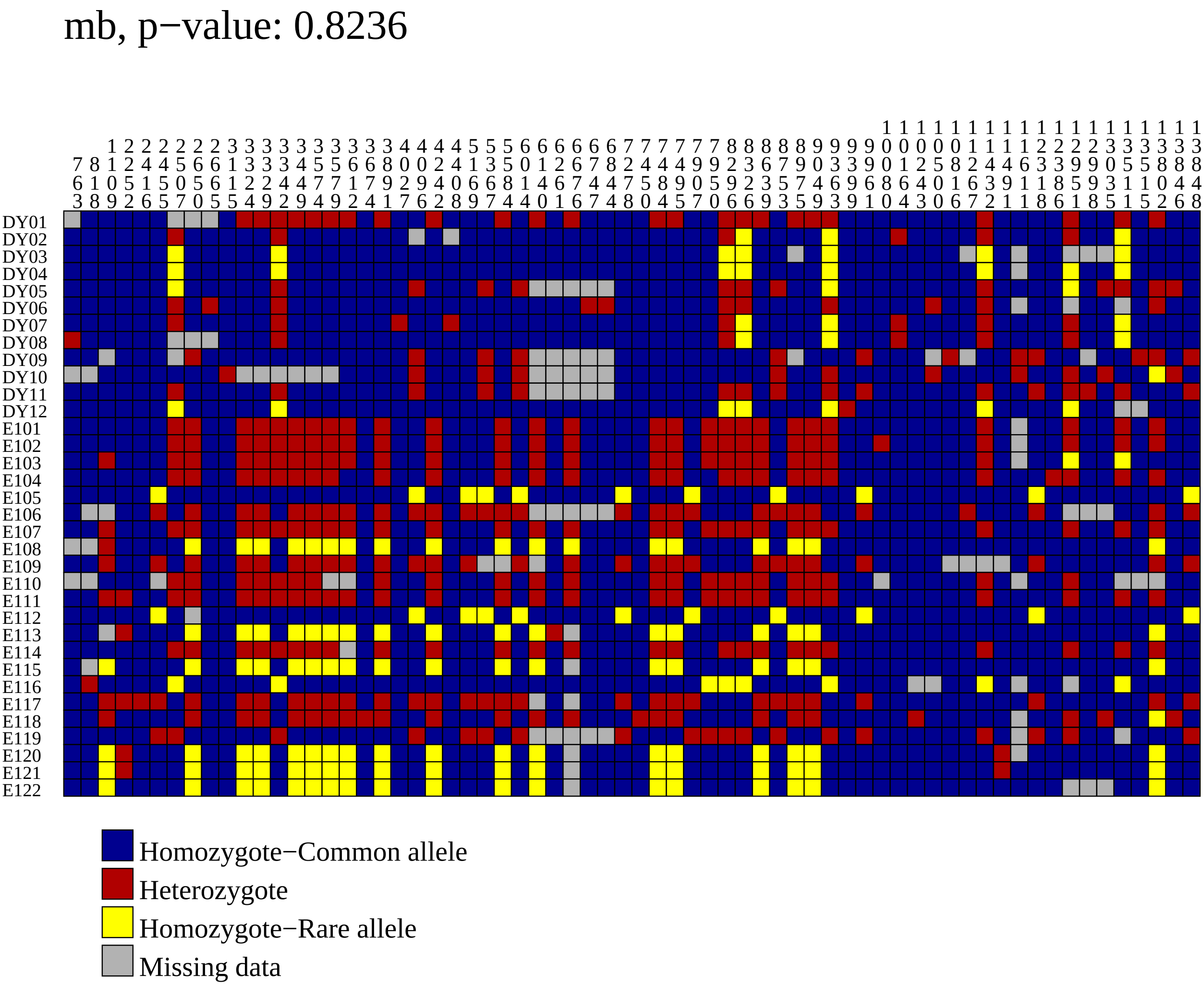
mb, p−value: 0.8236
DY01
DY02
DY03
DY04
DY05
DY06
DY07
DY08
DY09
DY10
DY11
DY12
E101
E102
E103
E104
E105
E106
E107
E108
E109
E110
E111
E112
E113
E114
E115
E116
E117
E118
E119
E120
E121
E122
Homozygote−Common allele
Heterozygote
Homozygote−Rare allele
Missing data

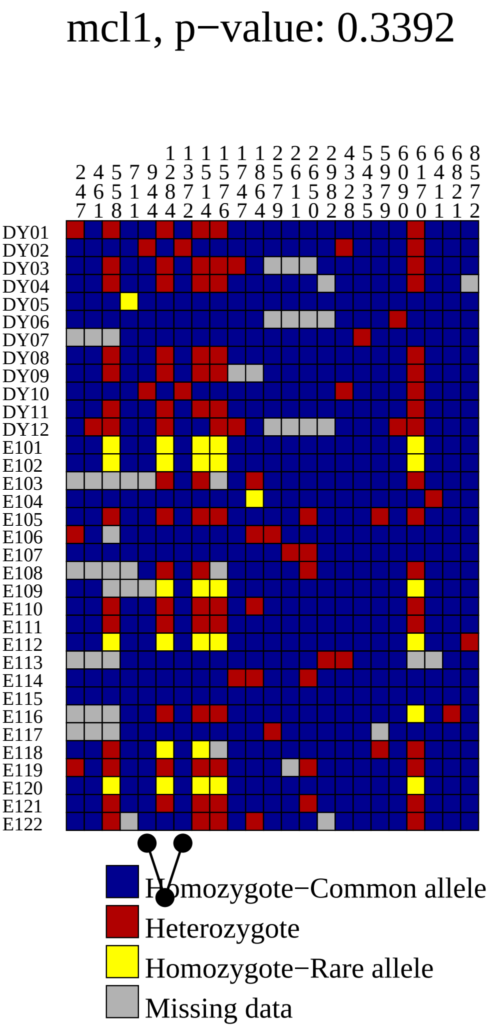
mcl1, p−value: 0.3392
247
461
558
711
944
1284
1372
1514
1576
1747
1864
2579
2611
2650
2982
4328
5435
5979
6090
6170
6411
6821
8572
DY01
DY02
DY03
DY04
DY05
DY06
DY07
DY08
DY09
DY10
DY11
DY12
E101
E102
E103
E104
E105
E106
E107
E108
E109
E110
E111
E112
E113
E114
E115
E116
E117
E118
E119
E120
E121
E122
Homozygote−Common allele
Heterozygote
Homozygote−Rare allele
Missing data

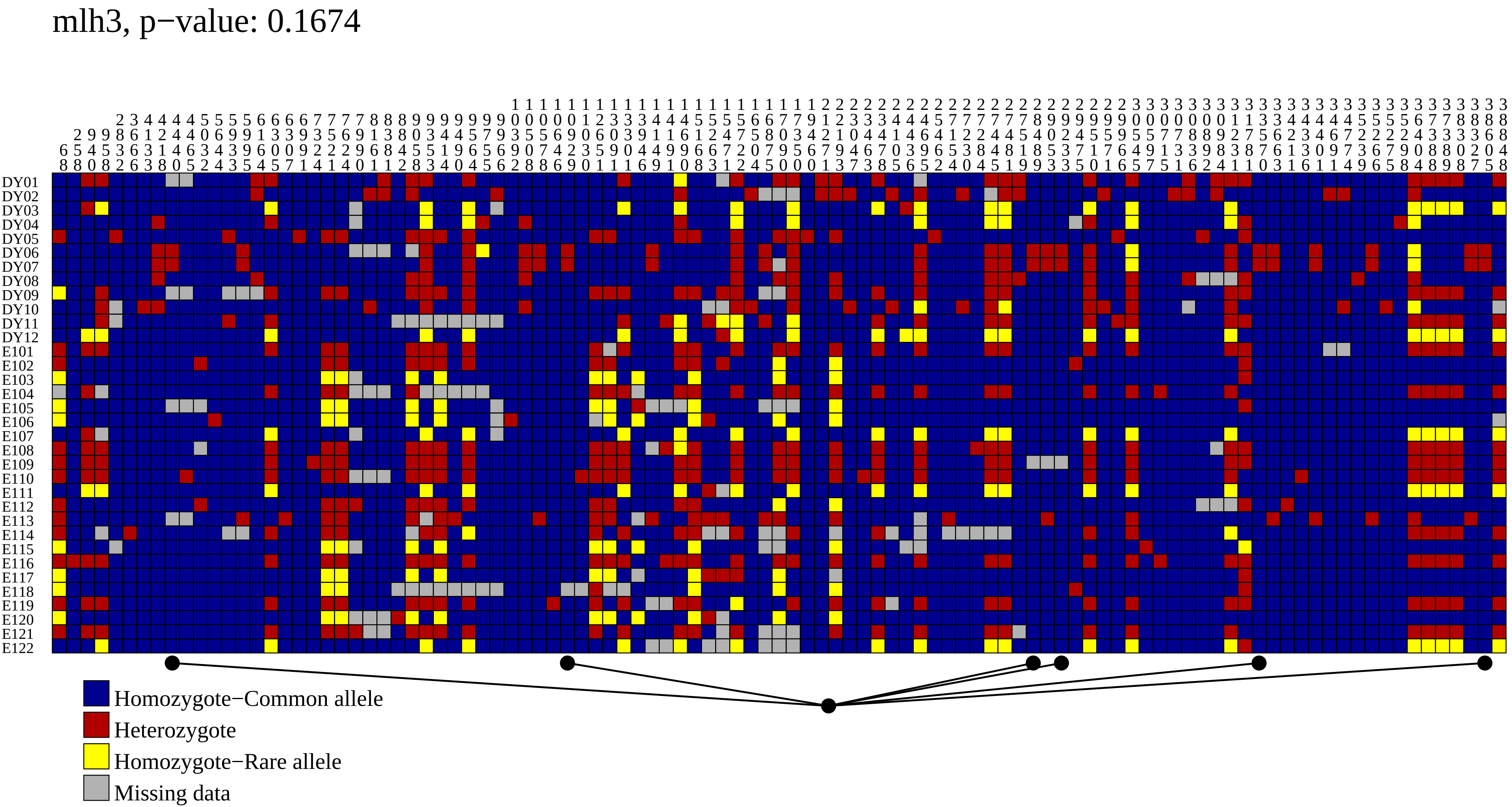
mlh3, p−value: 0.1674
Homozygote−Common allele
Heterozygote
Homozygote−Rare allele
Missing data

mmp11, p−value: 0.2266

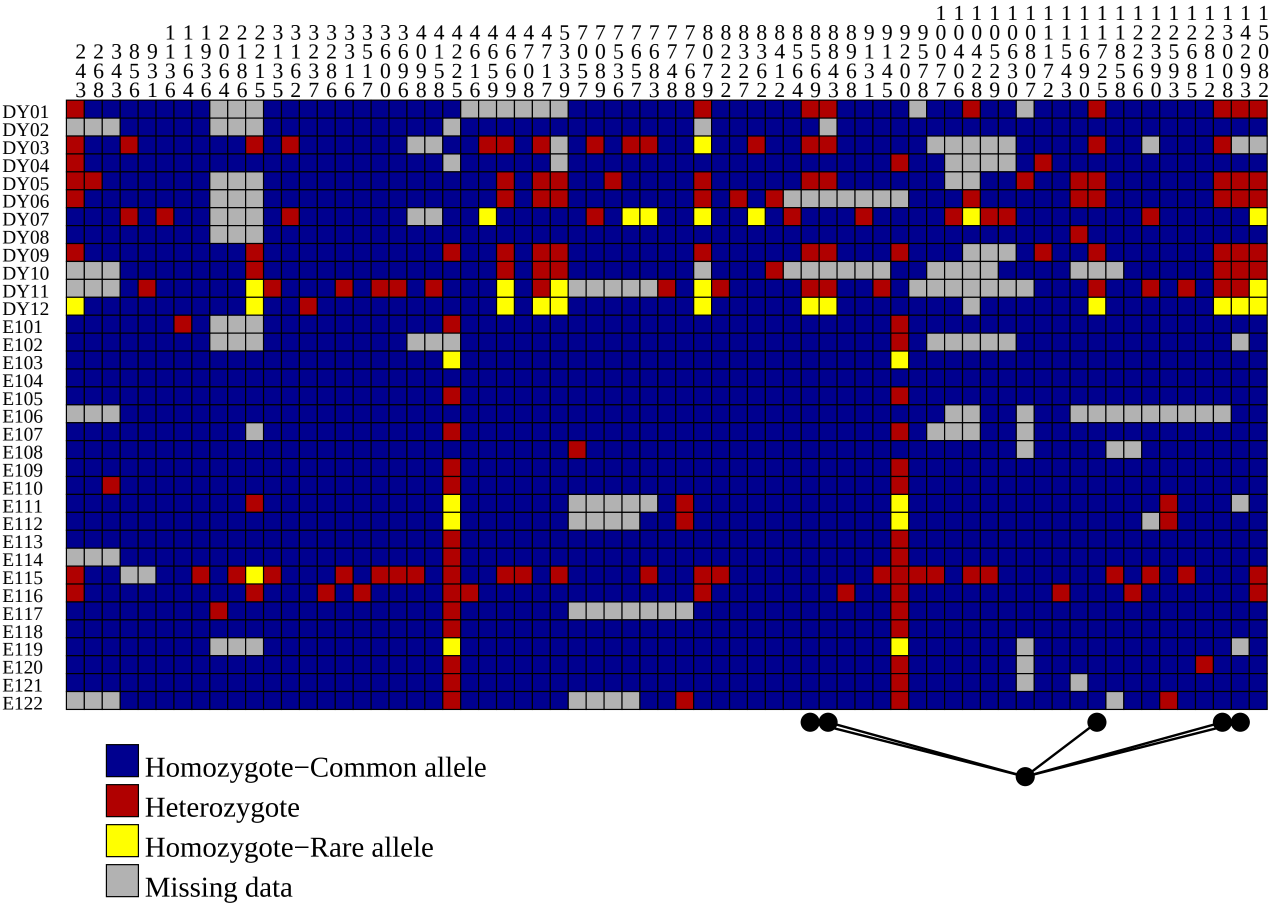

mmp12, p−value: 0.2398

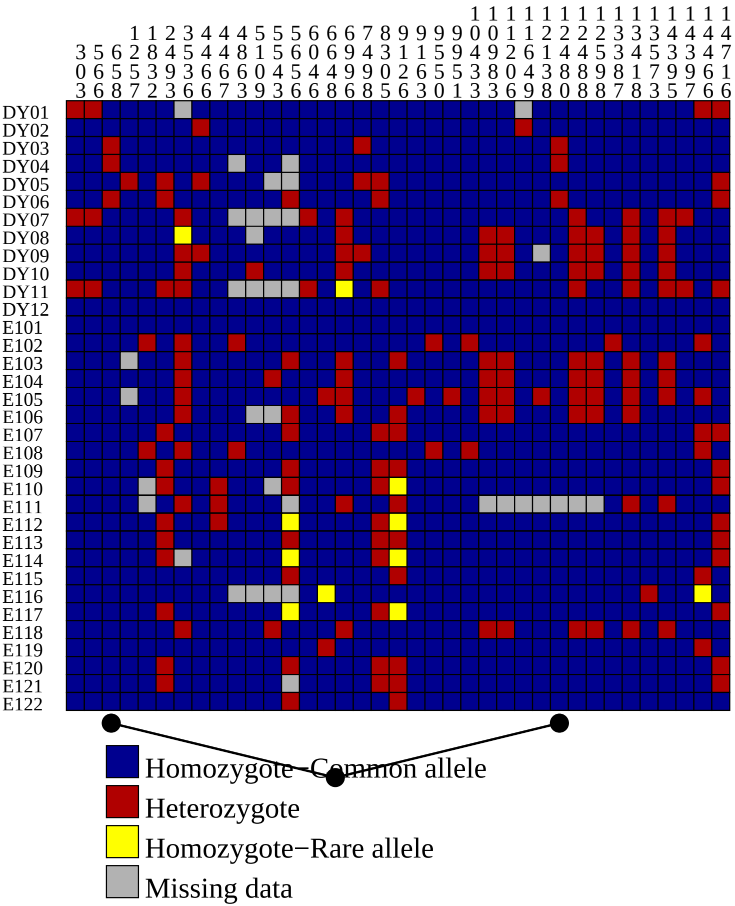

mmp16, p−value: 0.155

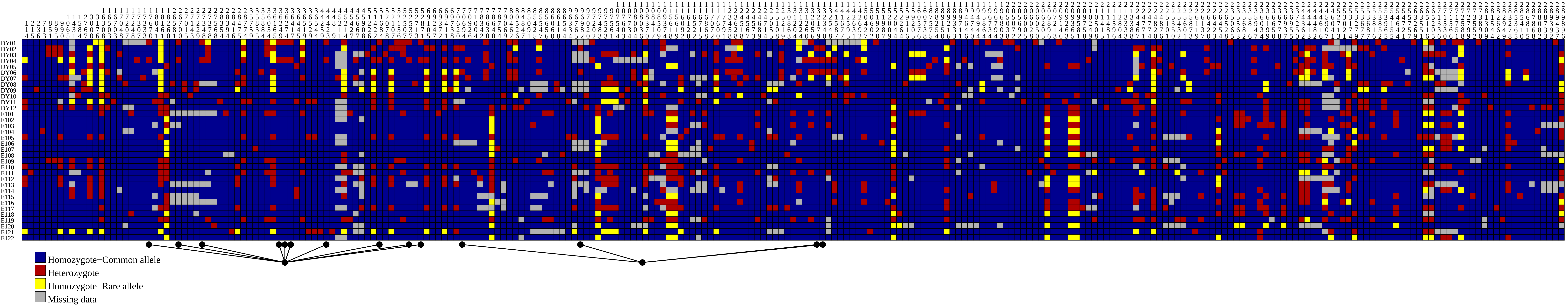

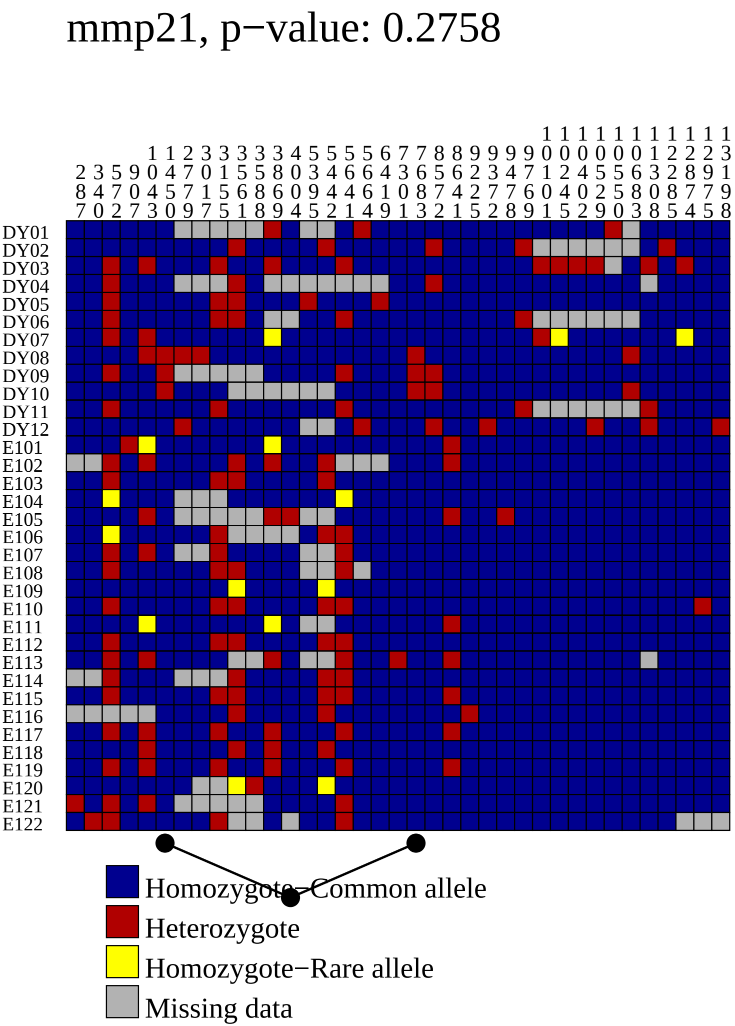

mmp21, p−value: 0.2758
287 340 572 907 1043 1450 2779 3017 3155 3561 3588 3869 4004 5395 5442 5641 5664 6419 7301 7683 8572 8641 9215 9372 9478 9769 10101 10245 10402 10529 10550 10683 11308 12285 12874 12975 13198
DY01
DY02
DY03
DY04
DY05
DY06
DY07
DY08
DY09
DY10
DY11
DY12
E101
E102
E103
E104
E105
E106
E107
E108
E109
E110
E111
E112
E113
E114
E115
E116
E117
E118
E119
E120
E121
E122
Homozygote−Common allele
Heterozygote
Homozygote−Rare allele
Missing data

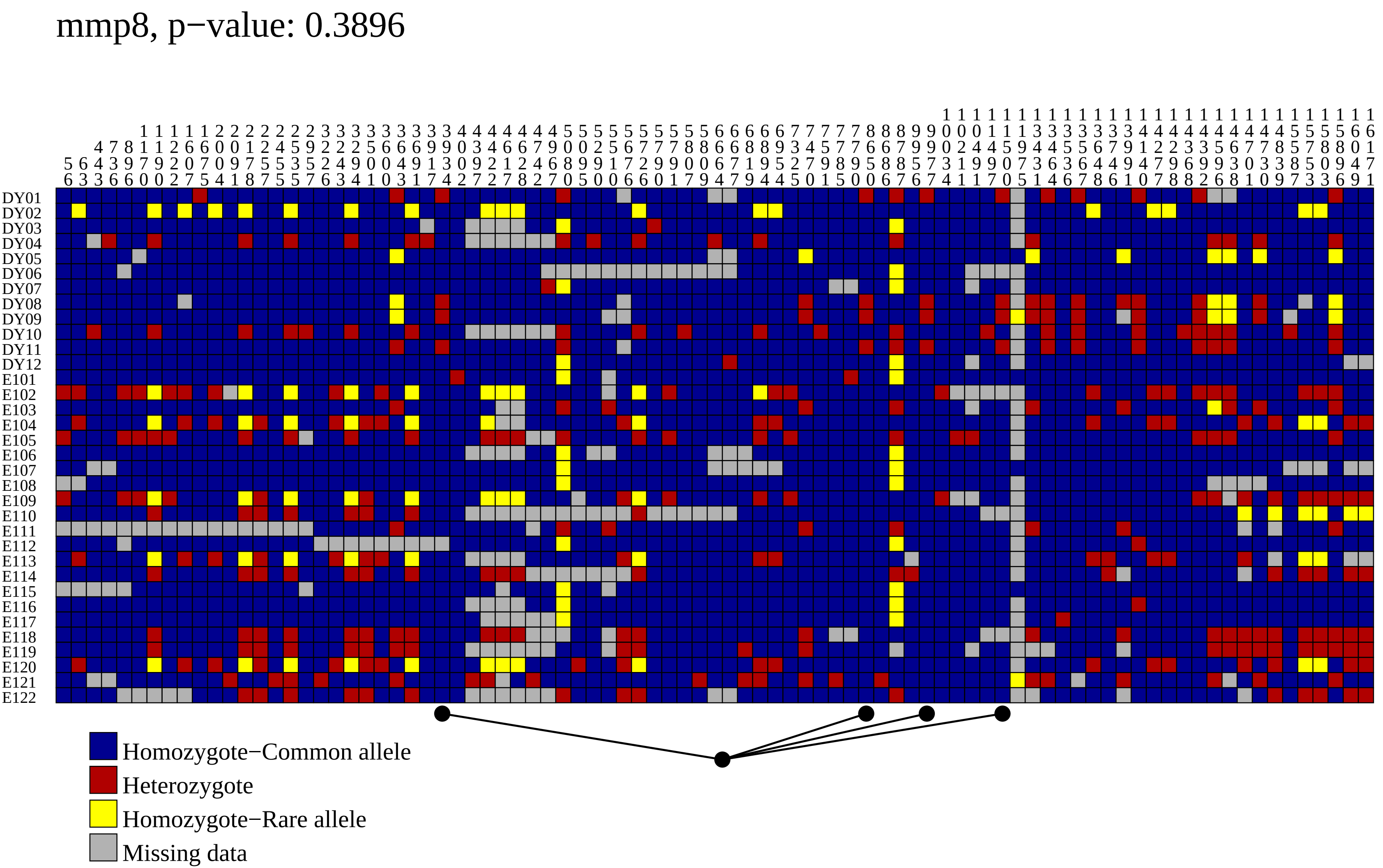

mmp8, p−value: 0.3896
DY01
DY02
DY03
DY04
DY05
DY06
DY07
DY08
DY09
DY10
DY11
DY12
E101
E102
E103
E104
E105
E106
E107
E108
E109
E110
E111
E112
E113
E114
E115
E116
E117
E118
E119
E120
E121
E122
Homozygote−Common allele
Heterozygote
Homozygote−Rare allele
Missing data

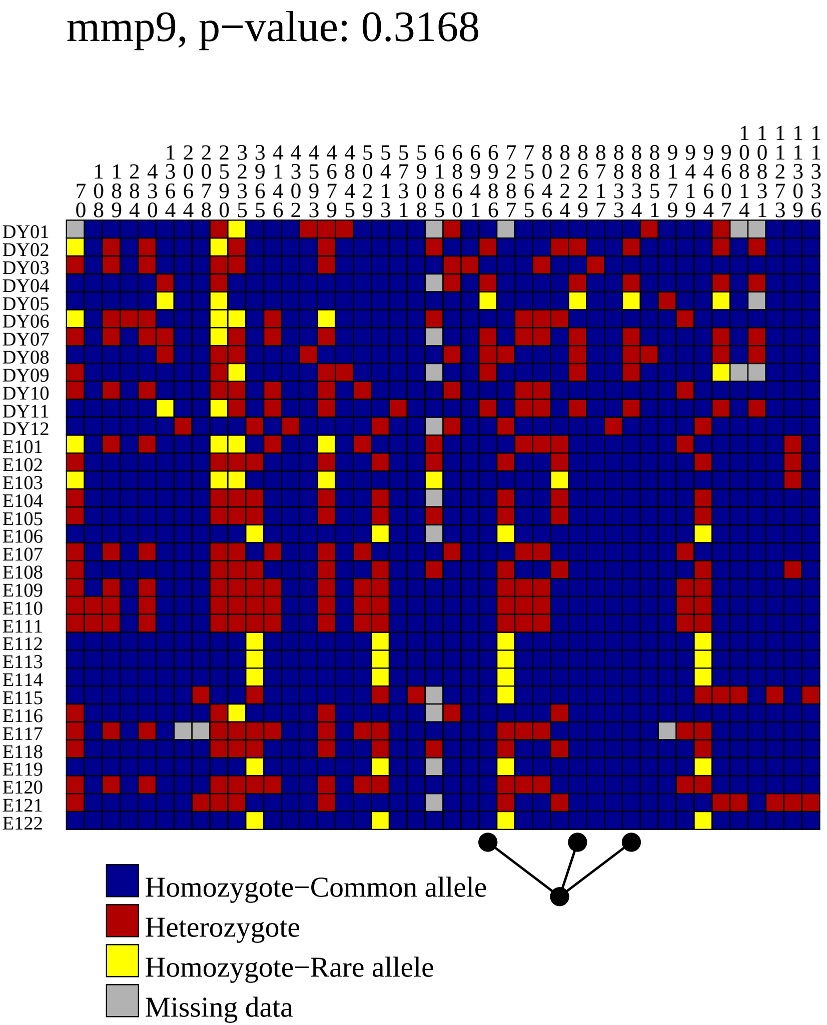
mmp9, p−value: 0.3168
70
108
189
284
430
1364
2064
2078
2590
3235
3965
4146
4302
4593
4679
4845
5029
5413
5731
5908
6185
6860
6941
6986
7287
7565
8046
8224
8629
8717
8833
8834
8851
9179
9419
9464
9607
10814
10831
11213
11309
11336
DY01
DY02
DY03
DY04
DY05
DY06
DY07
DY08
DY09
DY10
DY11
DY12
E101
E102
E103
E104
E105
E106
E107
E108
E109
E110
E111
E112
E113
E114
E115
E116
E117
E118
E119
E120
E121
E122
Homozygote−Common allele
Heterozygote
Homozygote−Rare allele
Missing data

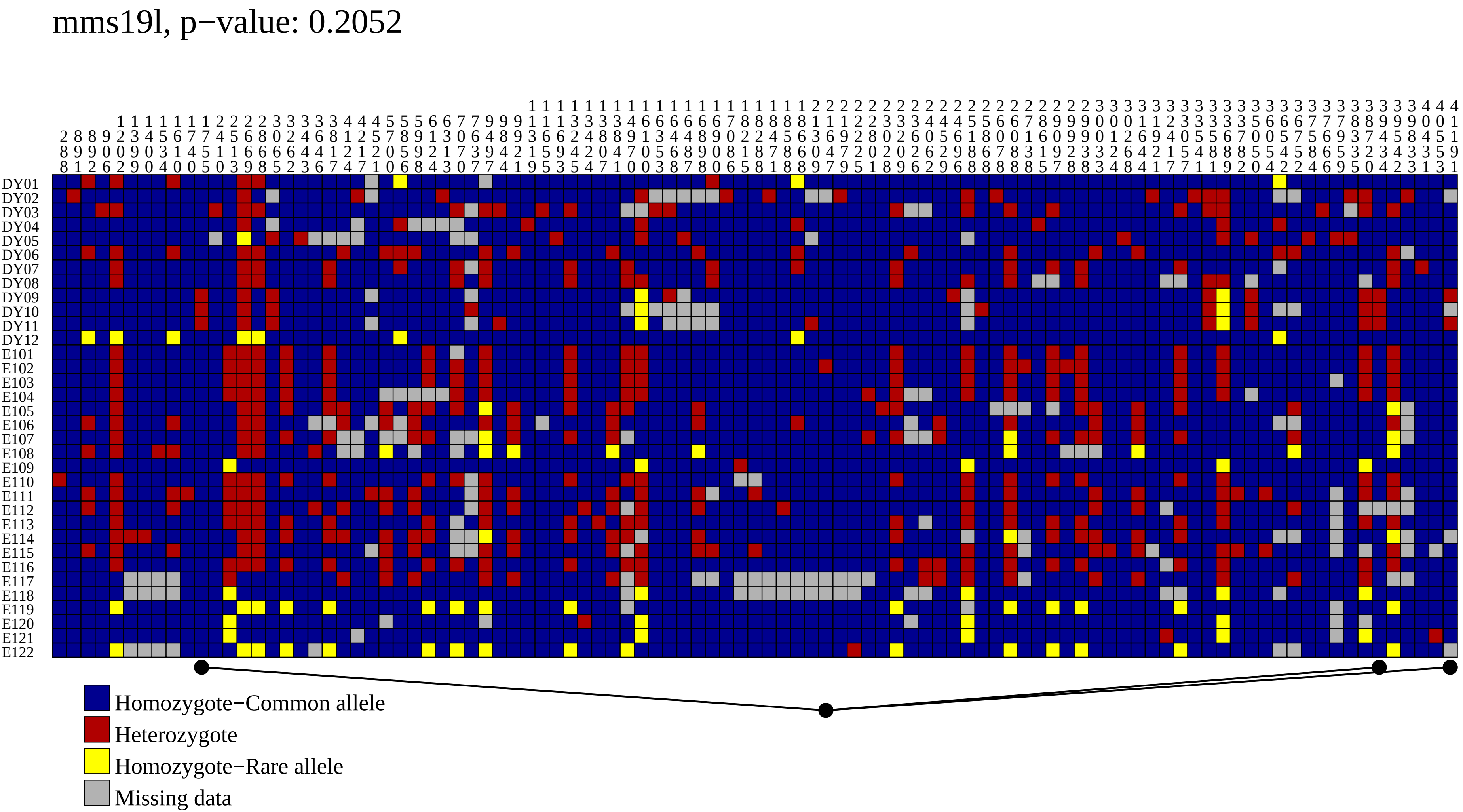
mms19l, p−value: 0.2052
DY01
DY02
DY03
DY04
DY05
DY06
DY07
DY08
DY09
DY10
DY11
DY12
E101
E102
E103
E104
E105
E106
E107
E108
E109
E110
E111
E112
E113
E114
E115
E116
E117
E118
E119
E120
E121
E122
Homozygote−Common allele
Heterozygote
Homozygote−Rare allele
Missing data

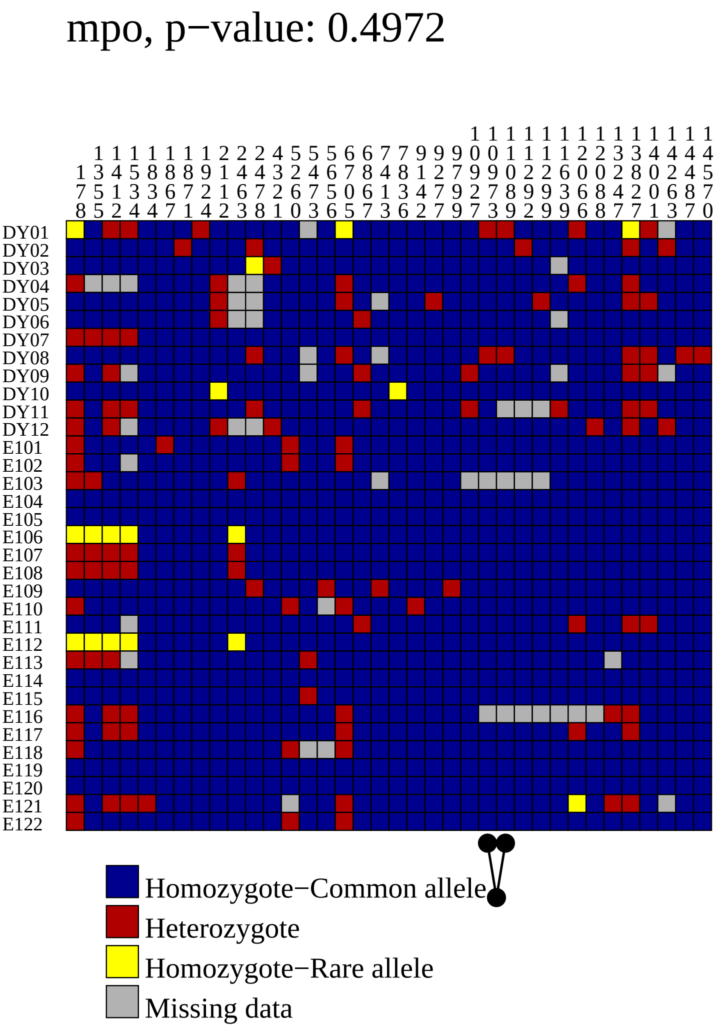
mpo, p−value: 0.4972
178 1355 1412 1534 1834 1867 1871 1914 2112 2463 2478 4321 5260 5473 5656 6705 6867 7413 7836 9142 9279 9799 10927 10973 11089 11292 11299 11639 12066 12088 13247 13827 14001 14263 14487 14570
DY01 DY02 DY03 DY04 DY05 DY06 DY07 DY08 DY09 DY10 DY11 DY12
E101 E102 E103 E104 E105 E106 E107 E108 E109 E110 E111 E112 E113 E114 E115 E116 E117 E118 E119 E120 E121 E122
Homozygote−Common allele
Heterozygote
Homozygote−Rare allele
Missing data

msh5, p−value: 0.2694

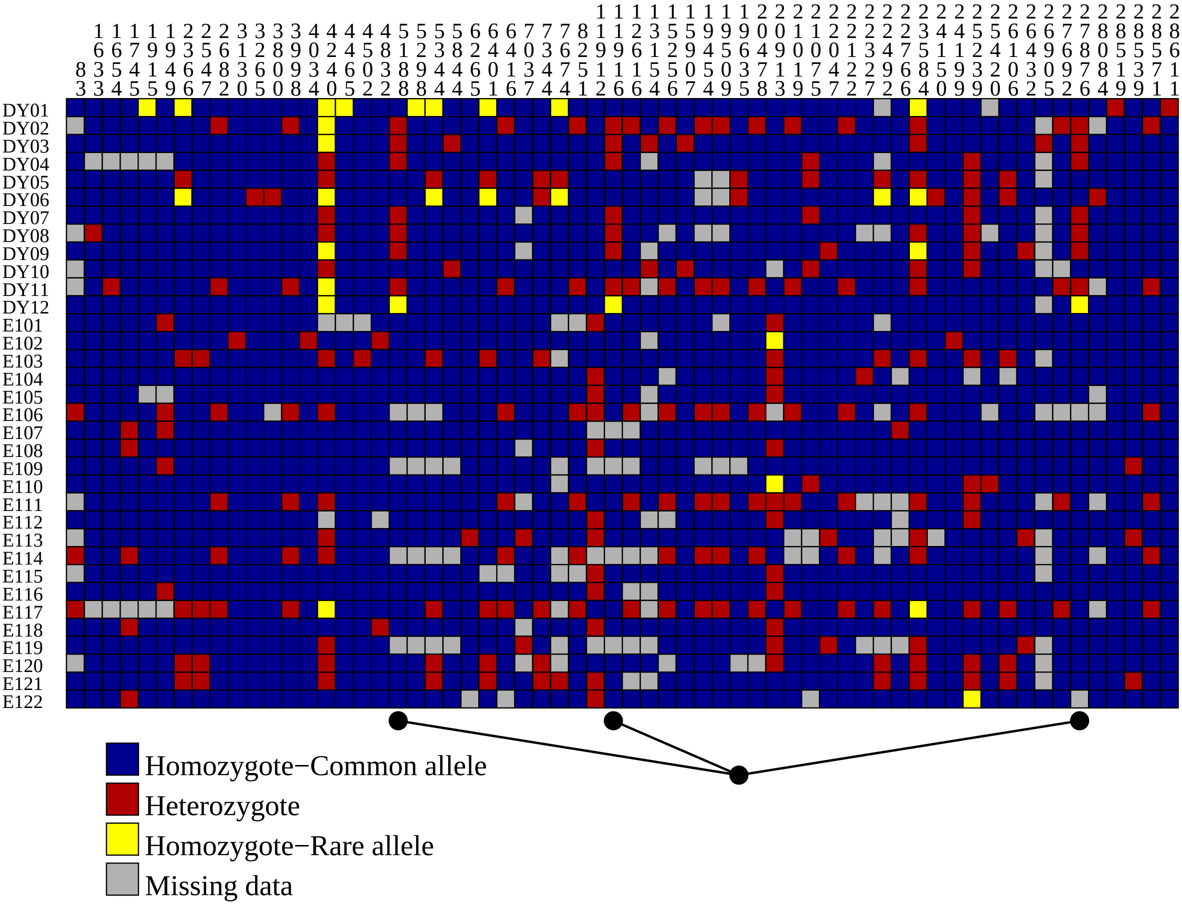

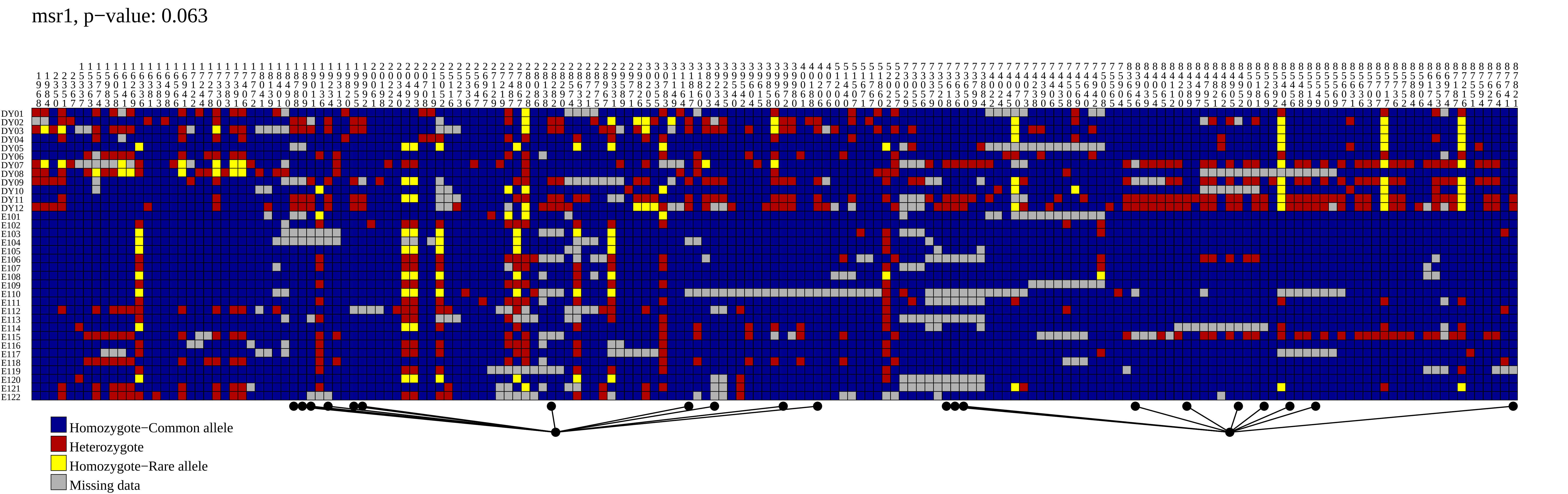
msr1, p−value: 0.063
Homozygote−Common allele
Heterozygote
Homozygote−Rare allele
Missing data

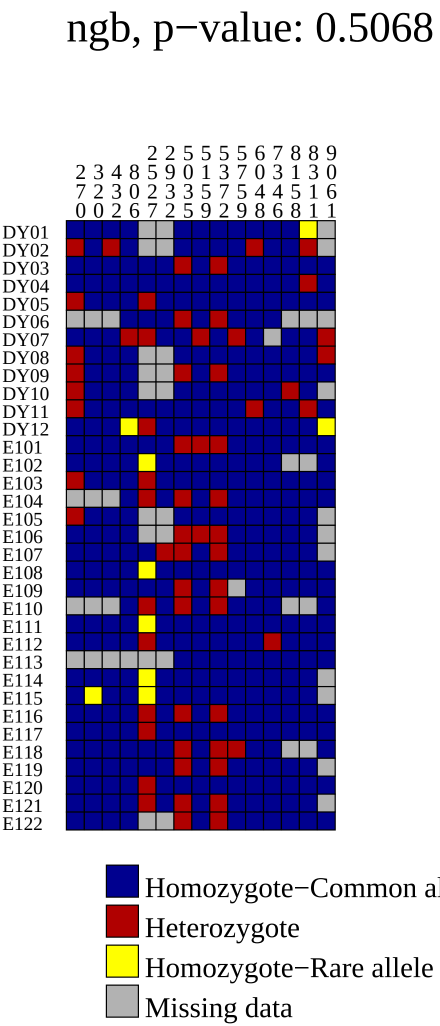

ngb, p−value: 0.5068
270
320
432
806
2527
2932
5035
5159
5372
5759
6048
7346
8158
8301
9061
DY01
DY02
DY03
DY04
DY05
DY06
DY07
DY08
DY09
DY10
DY11
DY12
E101
E102
E103
E104
E105
E106
E107
E108
E109
E110
E111
E112
E113
E114
E115
E116
E117
E118
E119
E120
E121
E122
Homozygote−Common
Heterozygote
Homozygote−Rare allele
Missing data

nos2a, p−value: 0.1934

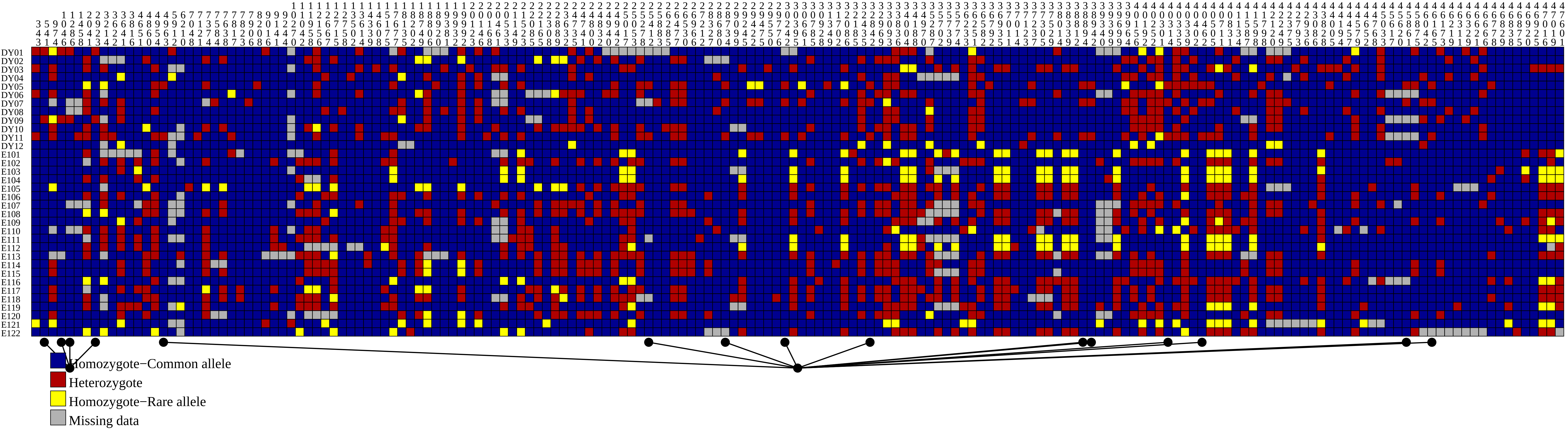

nudt1, p−value: 0.4298

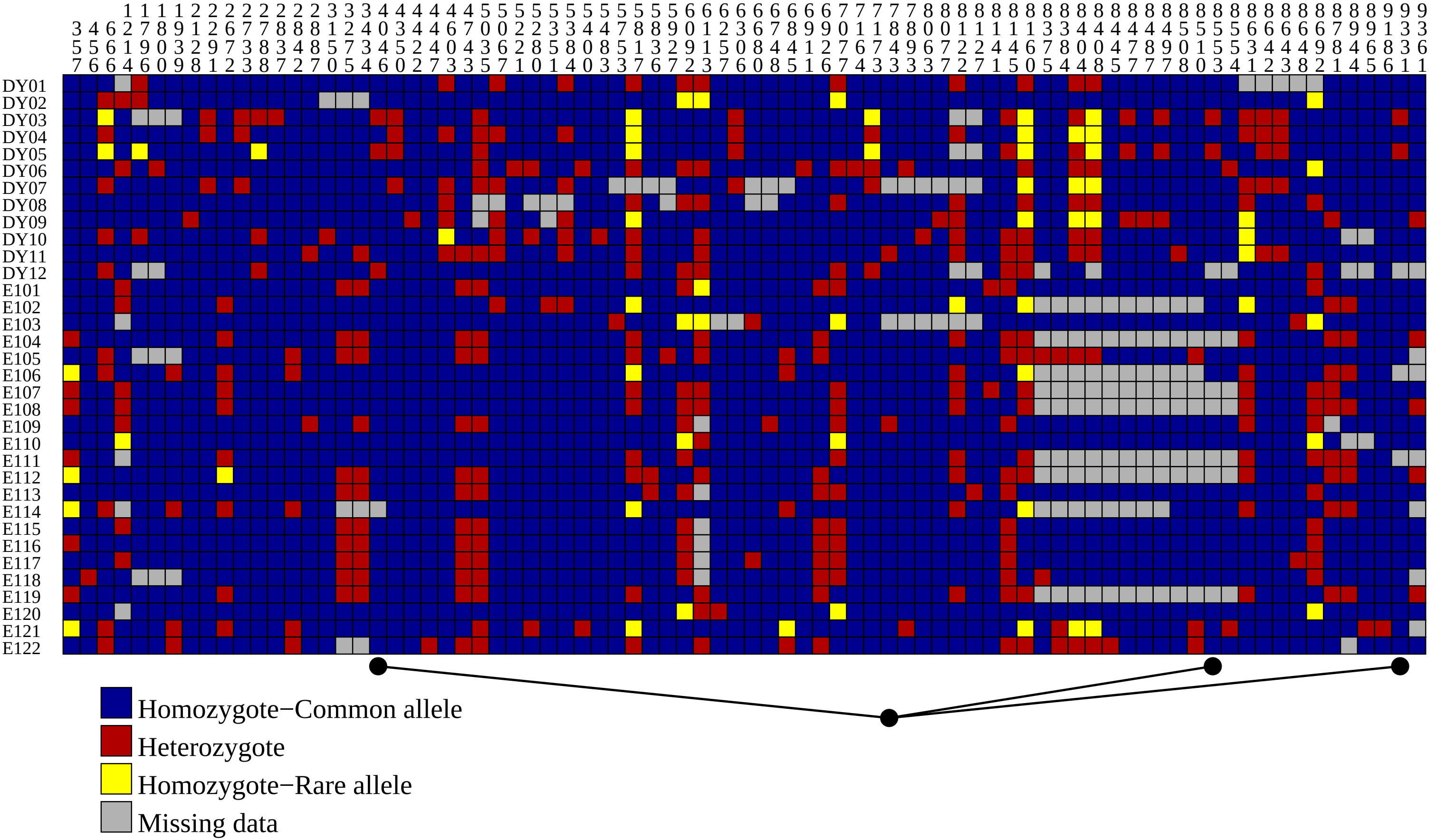

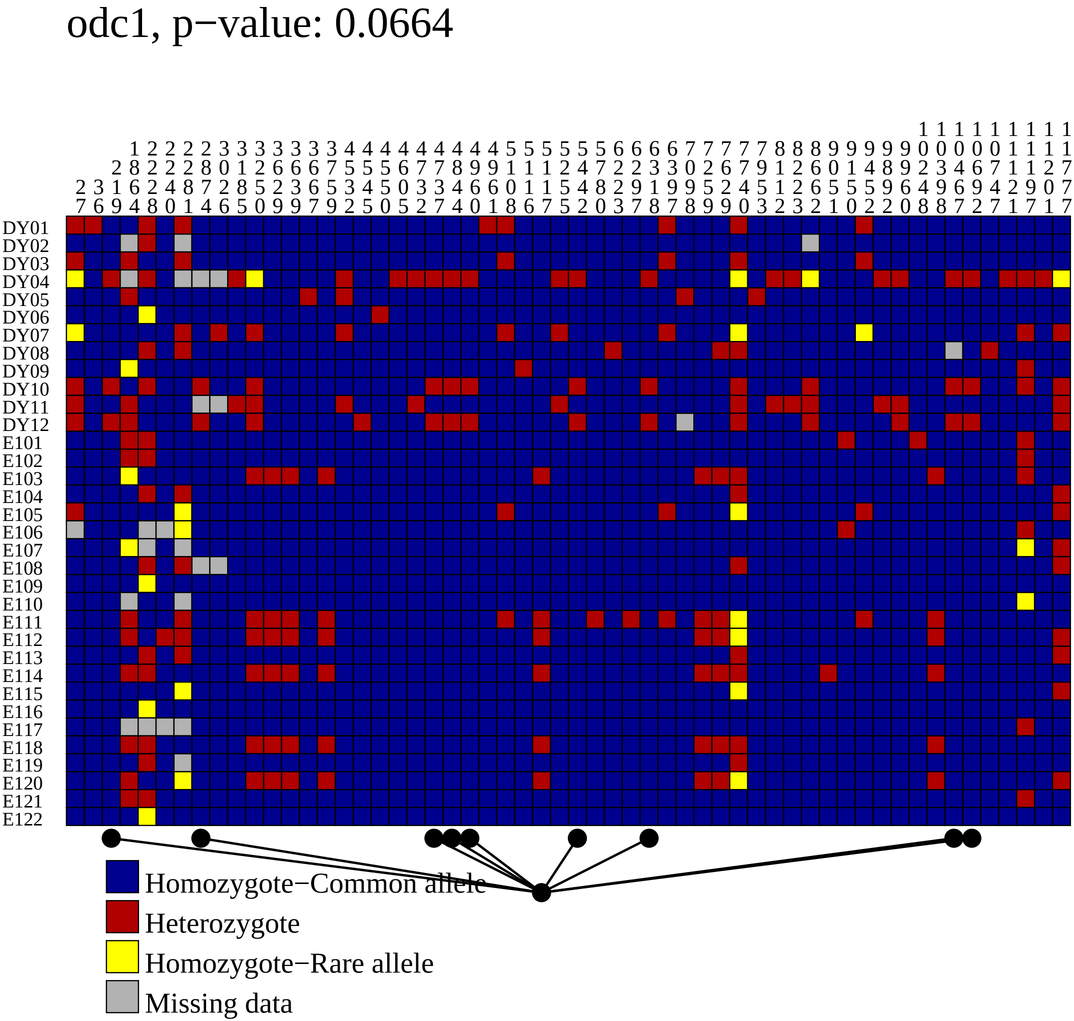

odc1, p−value: 0.0664
27
36
219
1864
2228
2240
2281
2874
3026
3185
3250
3629
3639
3667
3759
4532
4545
4550
4605
4732
4737
4844
4960
4961
5108
5116
5117
5255
5442
5780
6223
6297
6318
6397
7098
7259
7629
7740
7953
8112
8223
8662
9051
9150
9452
9892
9960
10248
10398
10467
10692
10747
11121
11197
11201
11717
DY01
DY02
DY03
DY04
DY05
DY06
DY07
DY08
DY09
DY10
DY11
DY12
E101
E102
E103
E104
E105
E106
E107
E108
E109
E110
E111
E112
E113
E114
E115
E116
E117
E118
E119
E120
E121
E122
Homozygote−Common allele
Heterozygote
Homozygote−Rare allele
Missing data

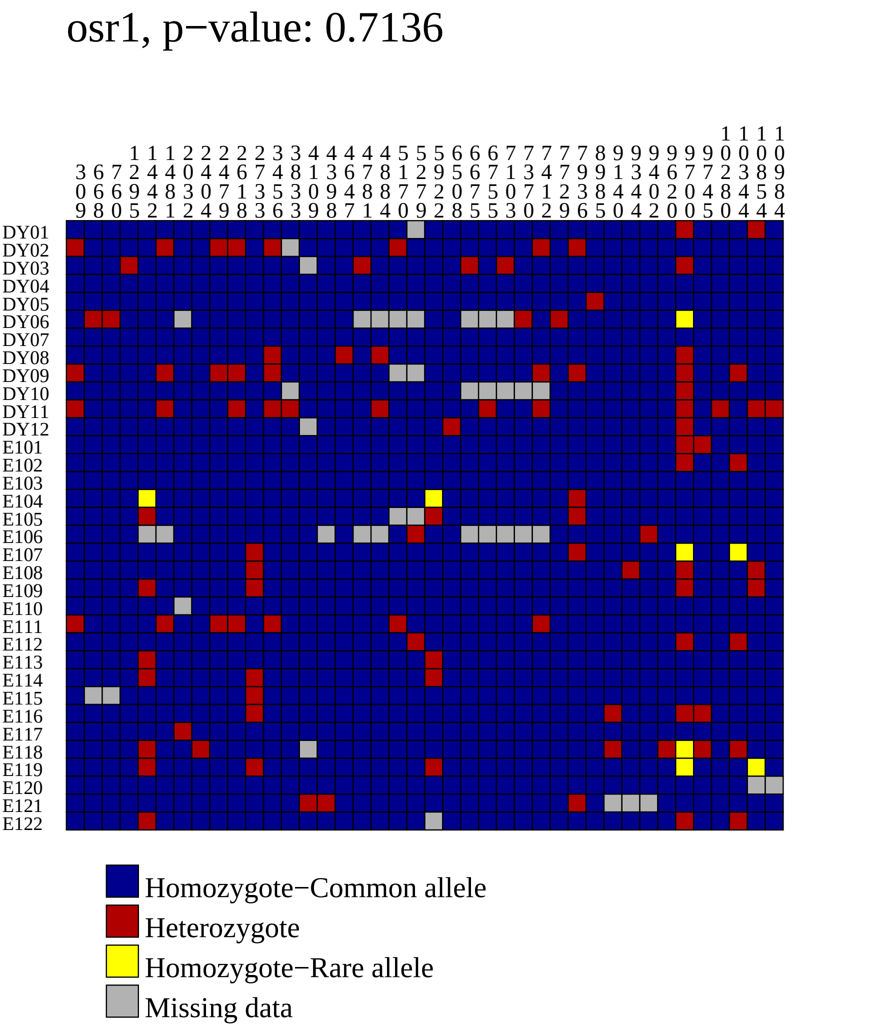
osr1, p−value: 0.7136
Homozygote−Common allele
Heterozygote
Homozygote−Rare allele
Missing data

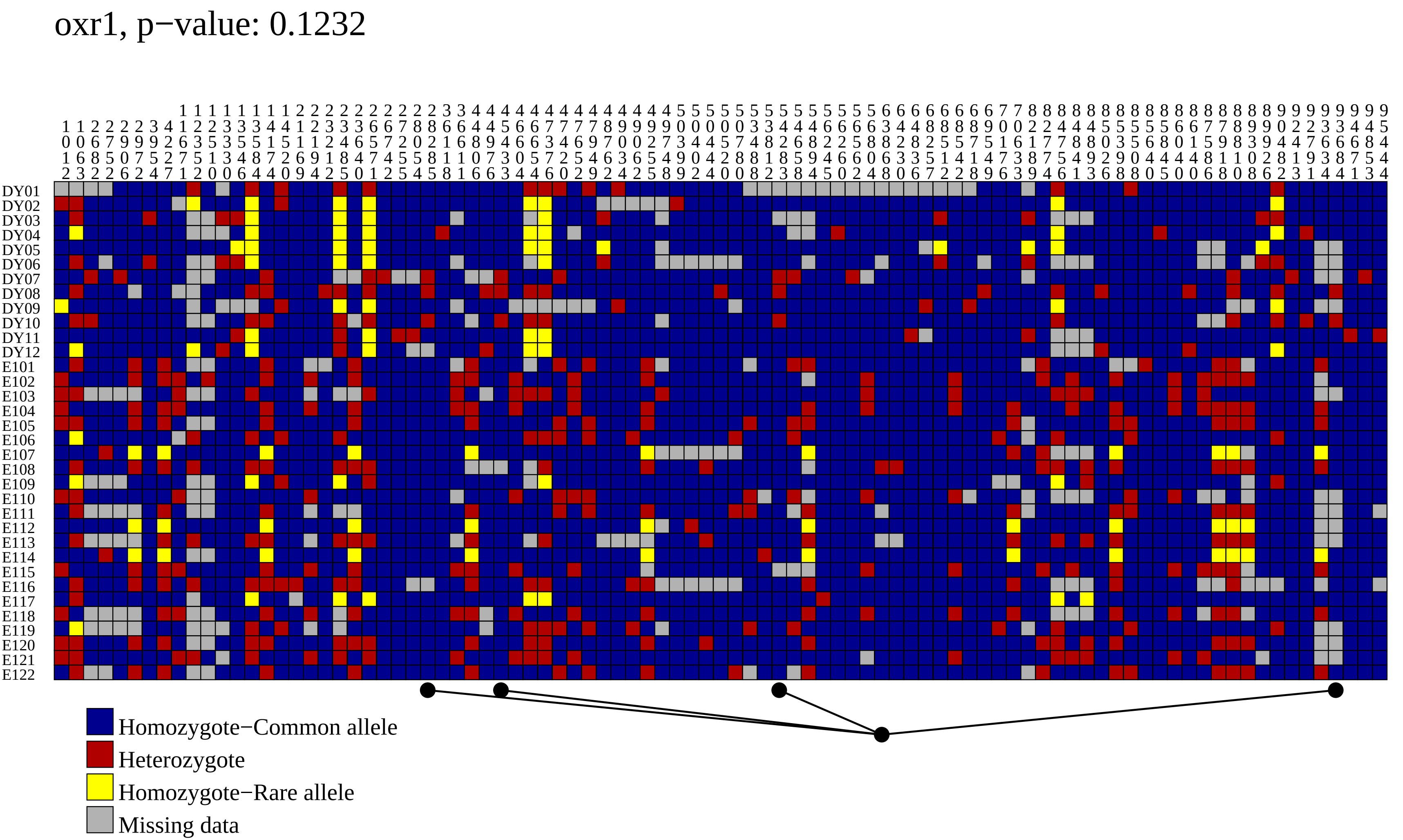
oxr1, p−value: 0.1232
DY01
DY02
DY03
DY04
DY05
DY06
DY07
DY08
DY09
DY10
DY11
DY12
E101
E102
E103
E104
E105
E106
E107
E108
E109
E110
E111
E112
E113
E114
E115
E116
E117
E118
E119
E120
E121
E122
Homozygote−Common allele
Heterozygote
Homozygote−Rare allele
Missing data

oxsr1, p−value: 0.0188

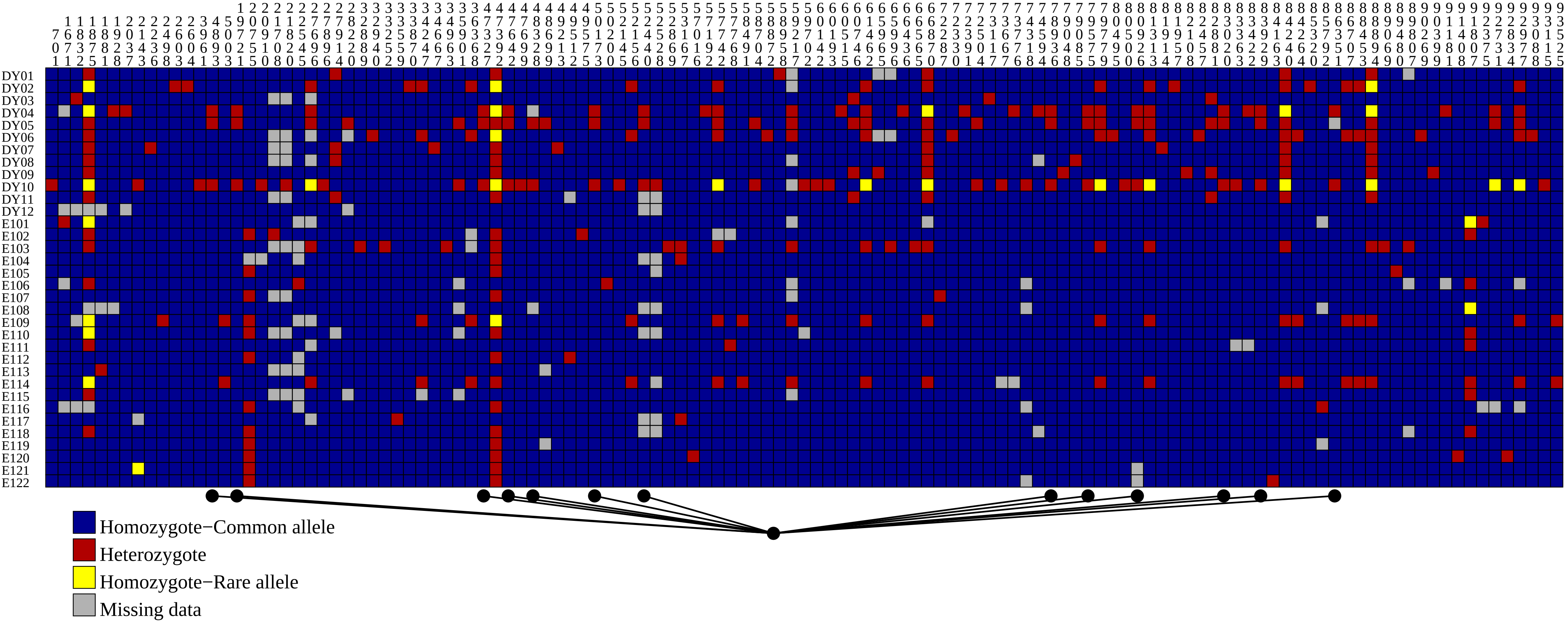

pax3, p−value: 0.8552

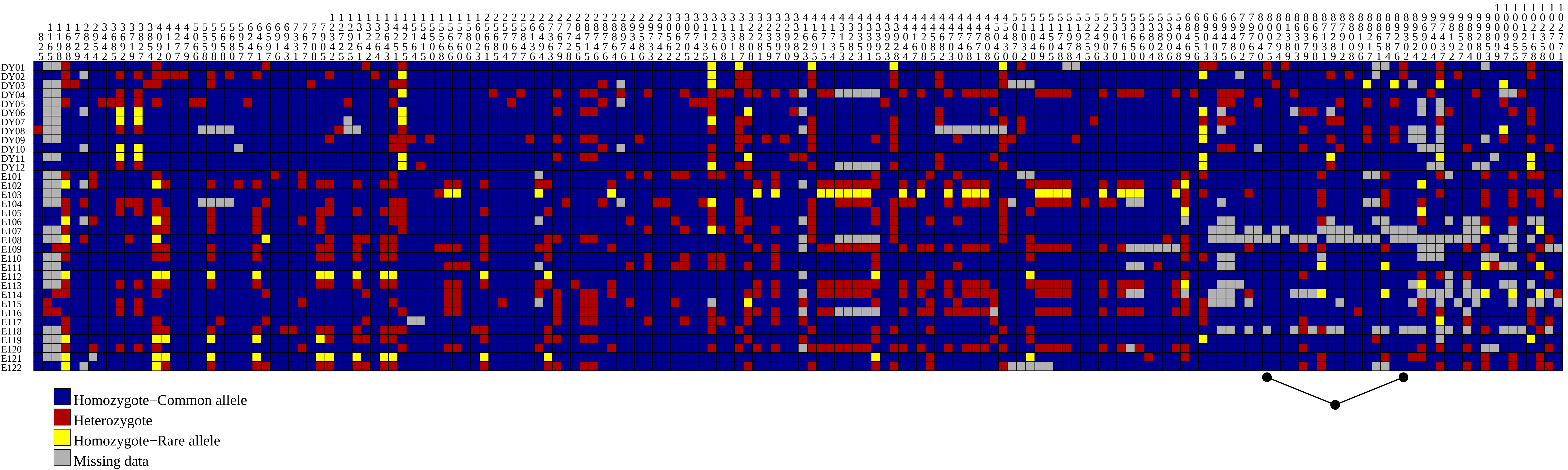

pdlim1, p−value: 0.3724

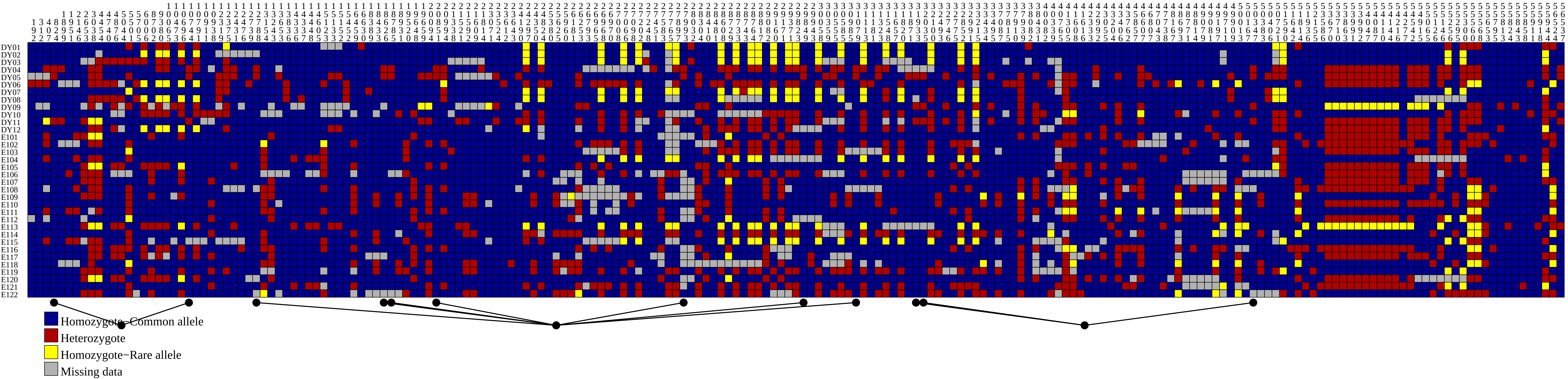

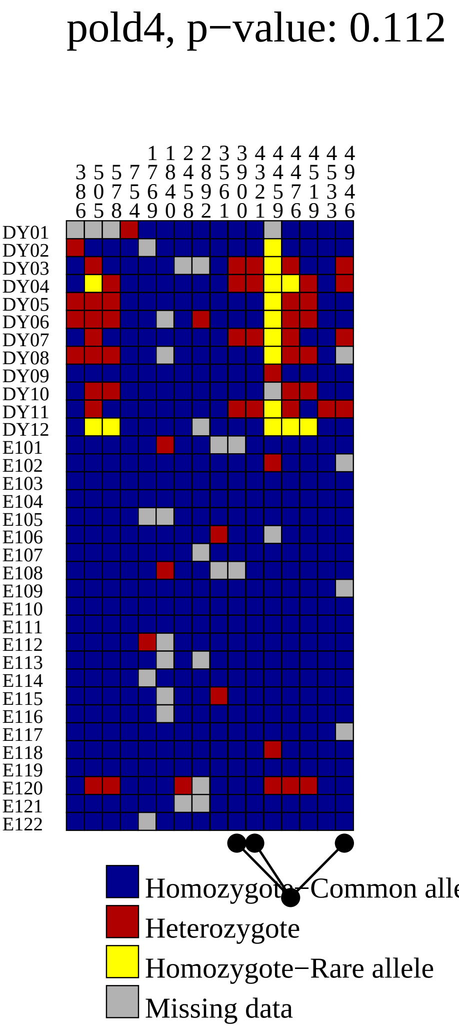
pold4, p−value: 0.112
386 505 578 754 1769 1840 2458 2892 3561 3900 4321 4459 4476 4519 4533 4946
DY01
DY02
DY03
DY04
DY05
DY06
DY07
DY08
DY09
DY10
DY11
DY12
E101
E102
E103
E104
E105
E106
E107
E108
E109
E110
E111
E112
E113
E114
E115
E116
E117
E118
E119
E120
E121
E122
Homozygote−Common alle
Heterozygote
Homozygote−Rare allele
Missing data

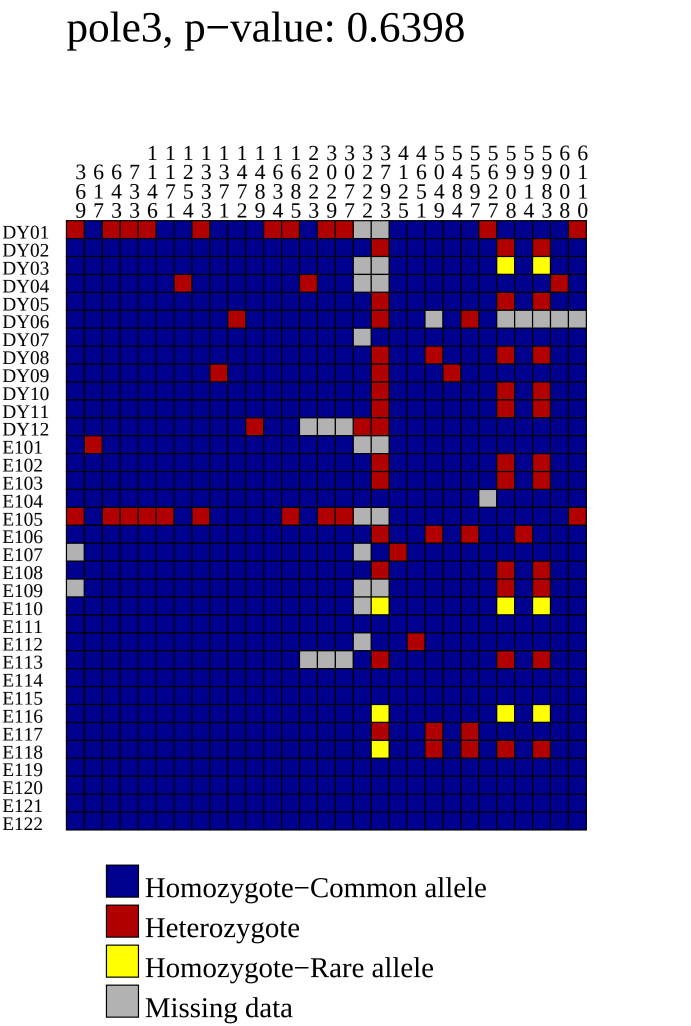

pole3, p−value: 0.6398
369
617
643
733
1146
1171
1254
1333
1371
1472
1489
1634
1685
2223
3029
3077
3222
3793
4125
4651
5049
5484
5597
5627
5908
5914
5983
6008
6010
DY01
DY02
DY03
DY04
DY05
DY06
DY07
DY08
DY09
DY10
DY11
DY12
E101
E102
E103
E104
E105
E106
E107
E108
E109
E110
E111
E112
E113
E114
E115
E116
E117
E118
E119
E120
E121
E122
Homozygote−Common allele
Heterozygote
Homozygote−Rare allele
Missing data

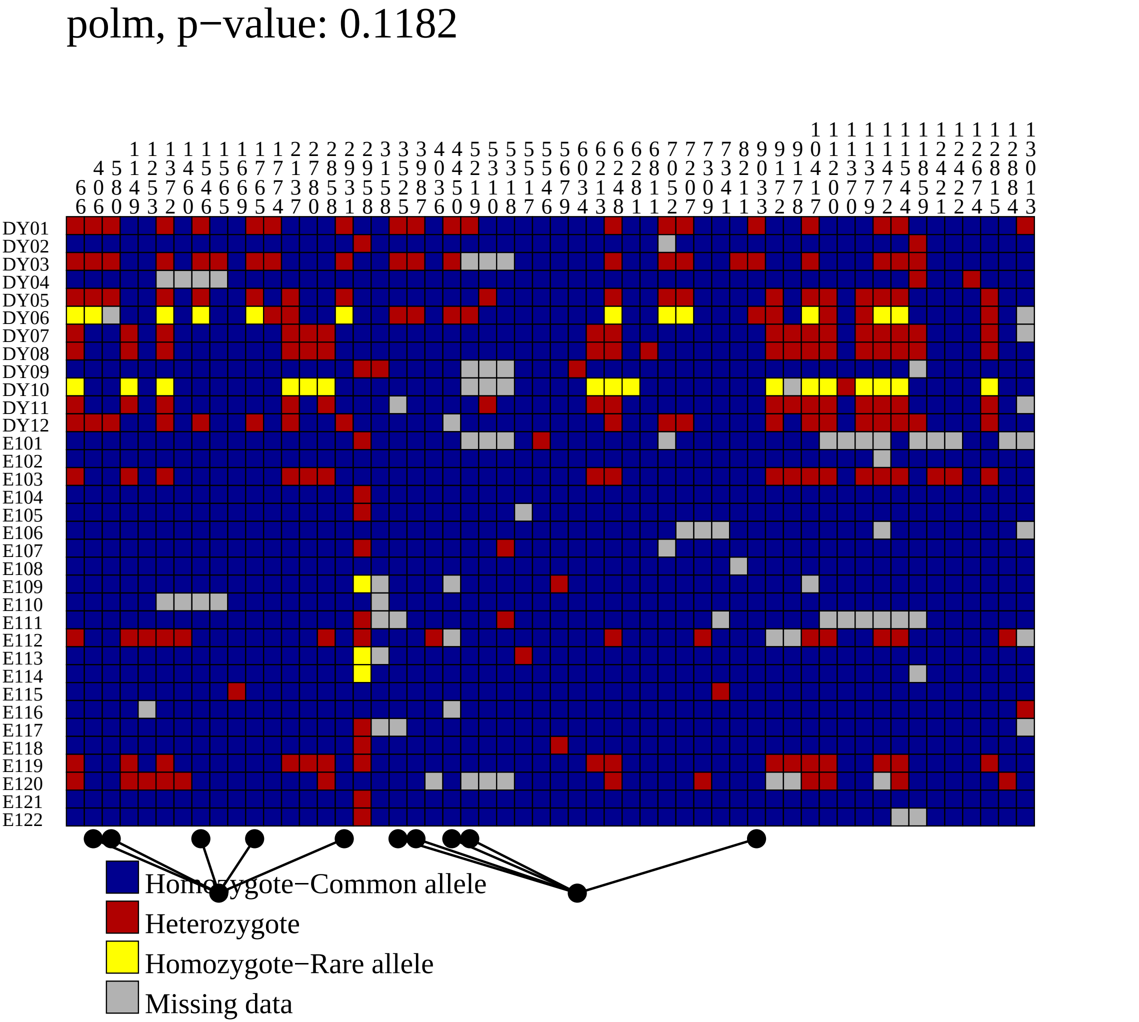
polm, p−value: 0.1182
Homozygote−Common allele
Heterozygote
Homozygote−Rare allele
Missing data

poln, p−value: 0.2084

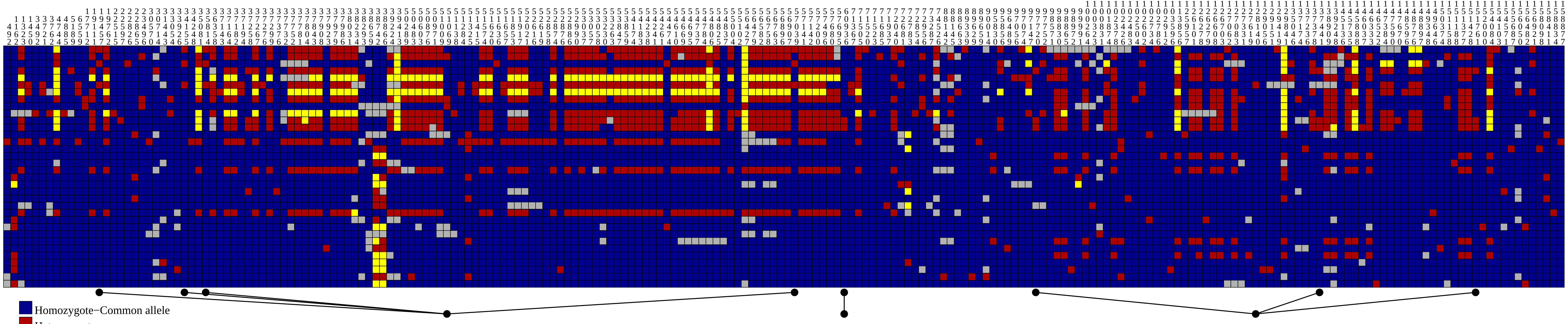

Homozygote−Common allele
Heterozygote
Homozygote−Rare allele
Missing data

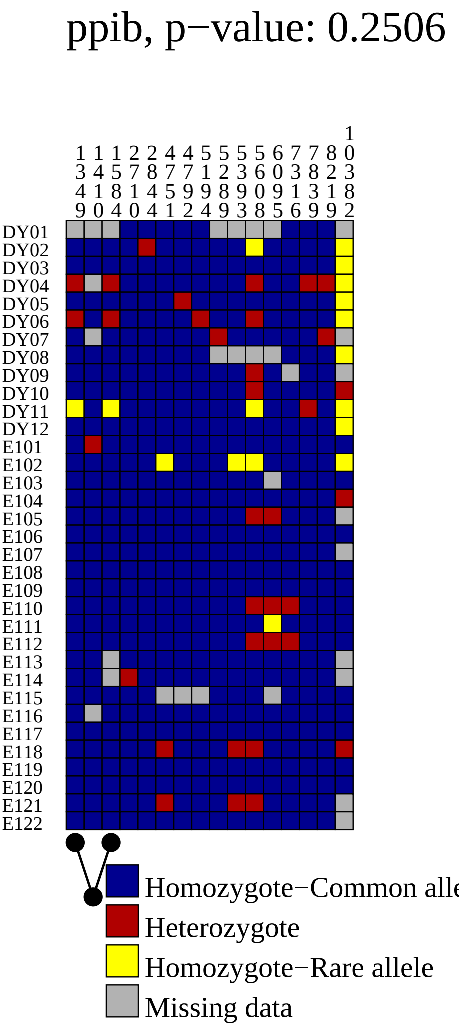
ppib, p−value: 0.2506
1349 1410 1584 2710 2844 4751 4792 5194 5289 5393 5608 6095 7316 7839 8219 10382
DY01 DY02 DY03 DY04 DY05 DY06 DY07 DY08 DY09 DY10 DY11 DY12
E101 E102 E103 E104 E105 E106 E107 E108 E109 E110 E111 E112 E113 E114 E115 E116 E117 E118 E119 E120 E121 E122
Homozygote−Common alle
Heterozygote
Homozygote−Rare allele
Missing data

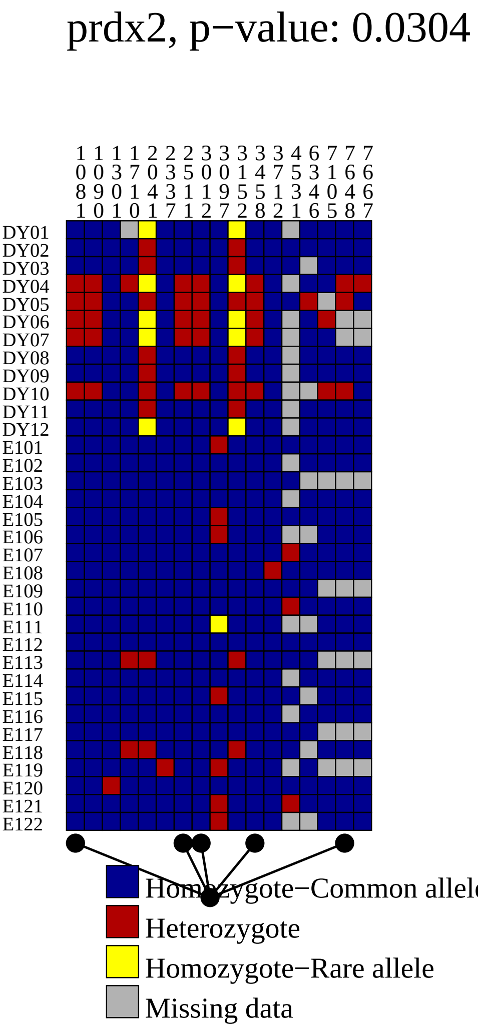
prdx2, p−value: 0.0304
1081
1090
1301
1710
2041
2337
2511
3012
3097
3152
3458
3712
4531
6346
7105
7648
7667
DY01
DY02
DY03
DY04
DY05
DY06
DY07
DY08
DY09
DY10
DY11
DY12
E101
E102
E103
E104
E105
E106
E107
E108
E109
E110
E111
E112
E113
E114
E115
E116
E117
E118
E119
E120
E121
E122
Homozygote−Common allele
Heterozygote
Homozygote−Rare allele
Missing data

prdx5, p−value: 0.0744

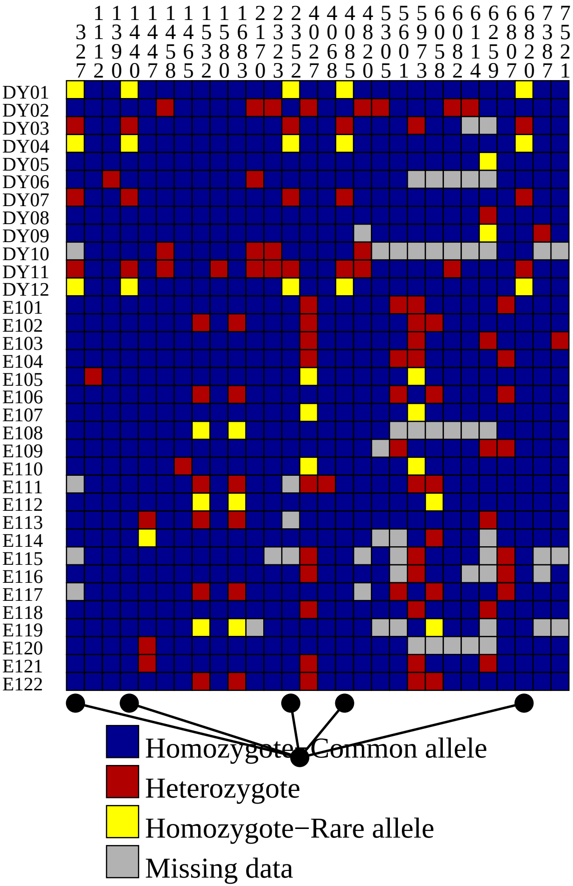

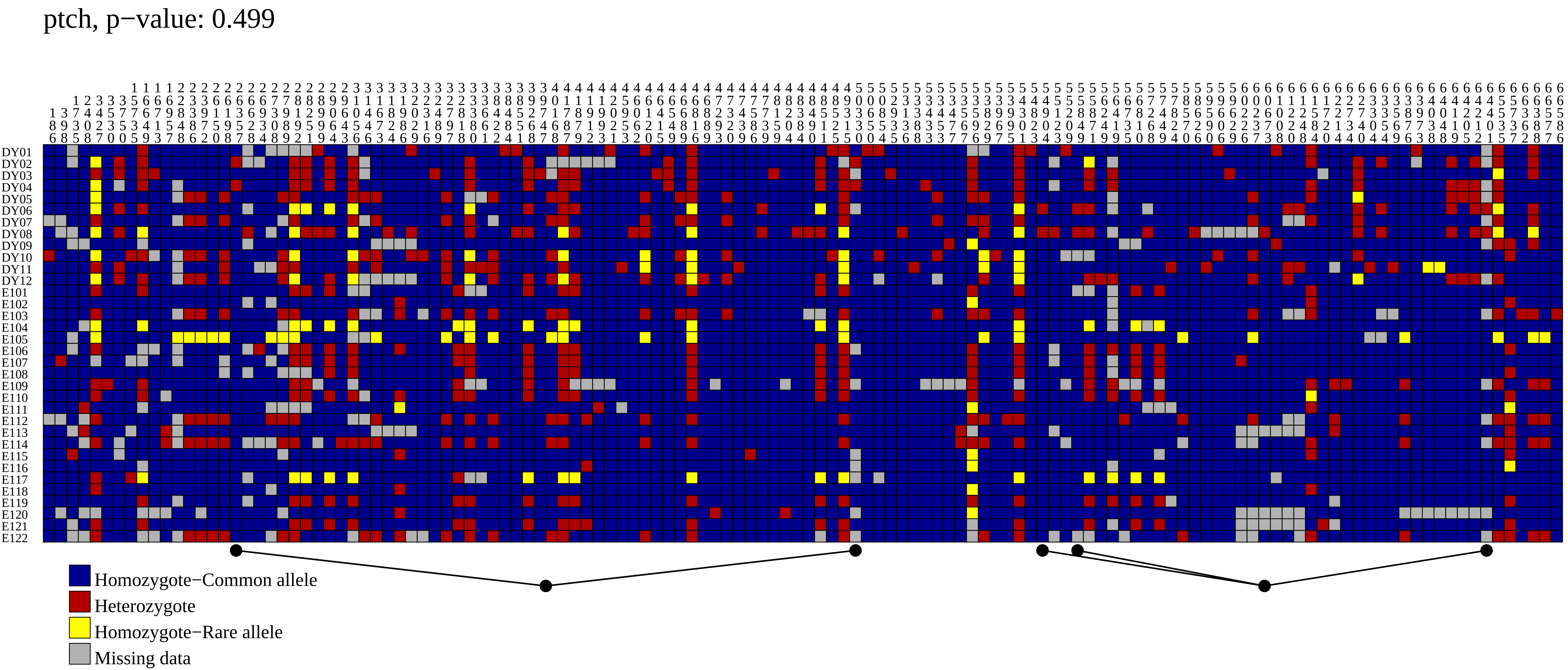
ptch, p−value: 0.499
Homozygote−Common allele
Heterozygote
Homozygote−Rare allele
Missing data

pten, p−value: 0.488

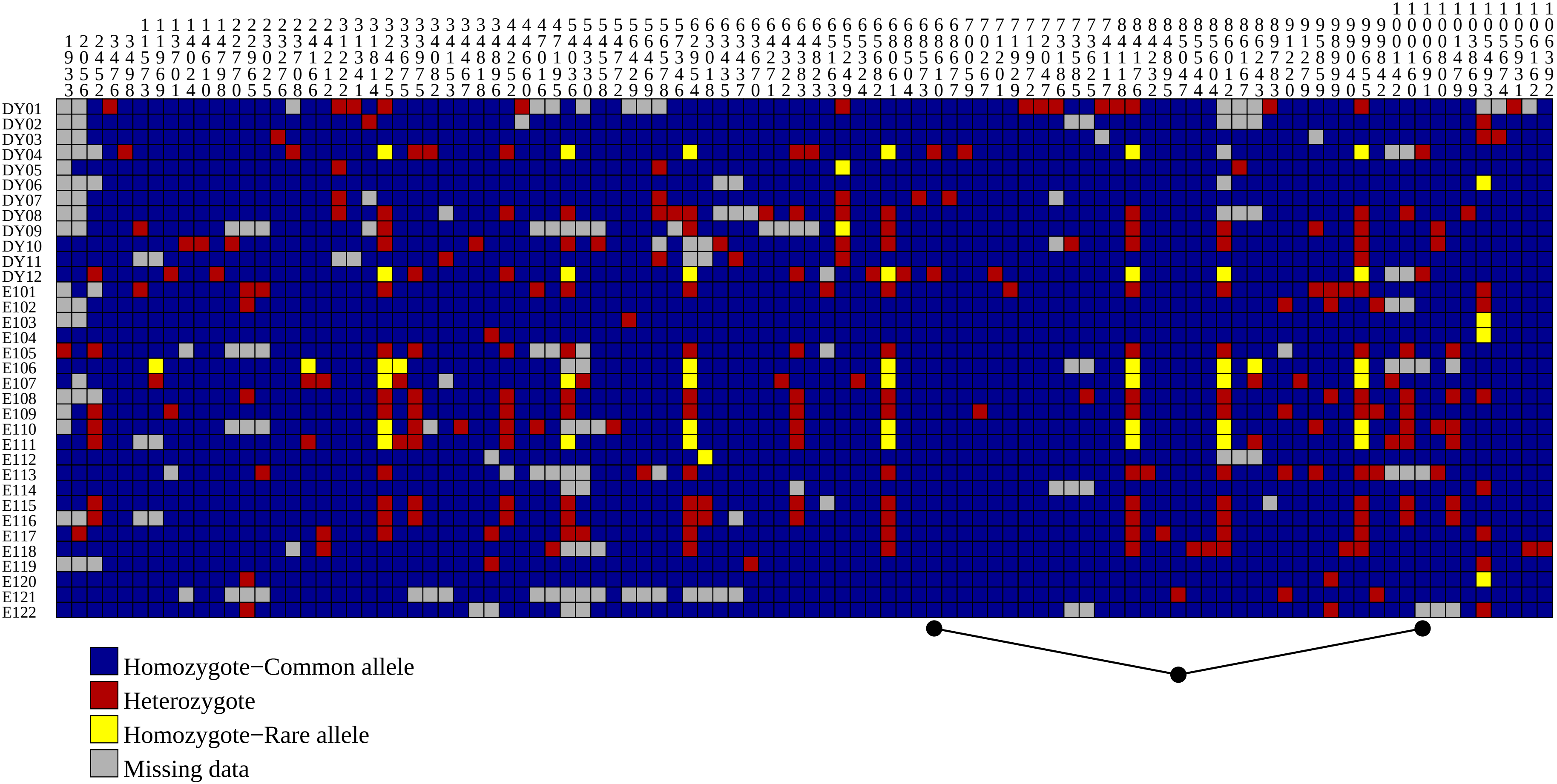

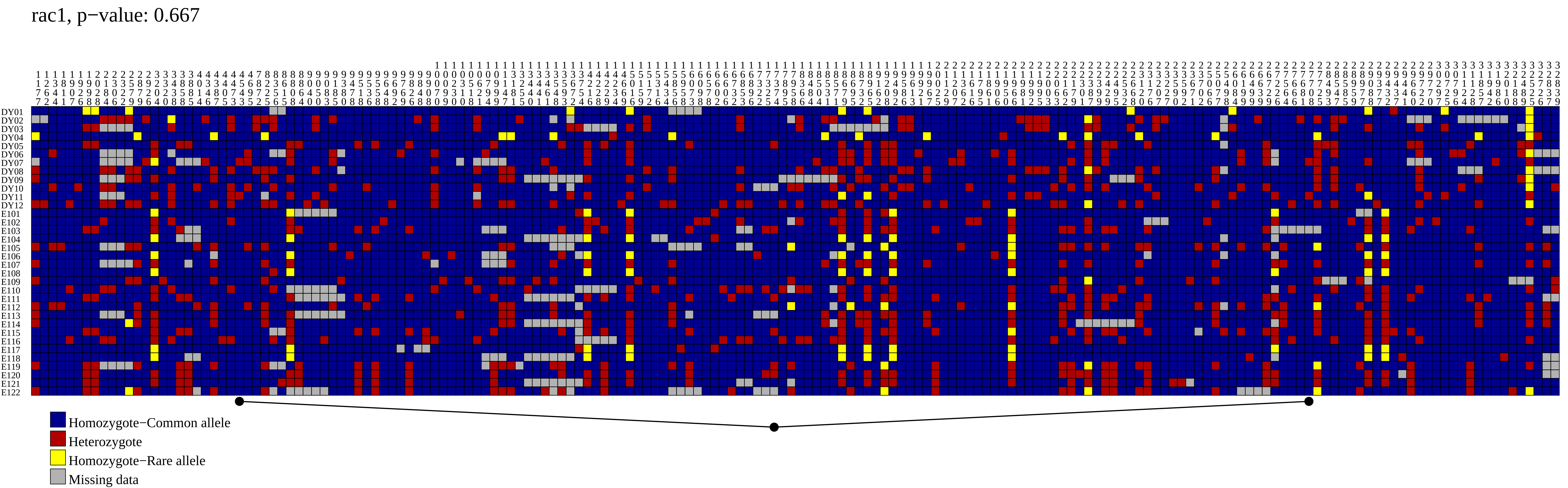
rac1, p−value: 0.667
DY01
DY02
DY03
DY04
DY05
DY06
DY07
DY08
DY09
DY10
DY11
DY12
E101
E102
E103
E104
E105
E106
E107
E108
E109
E110
E111
E112
E113
E114
E115
E116
E117
E118
E119
E120
E121
E122
Homozygote−Common allele
Heterozygote
Homozygote−Rare allele
Missing data

rad18, p−value: 0.06

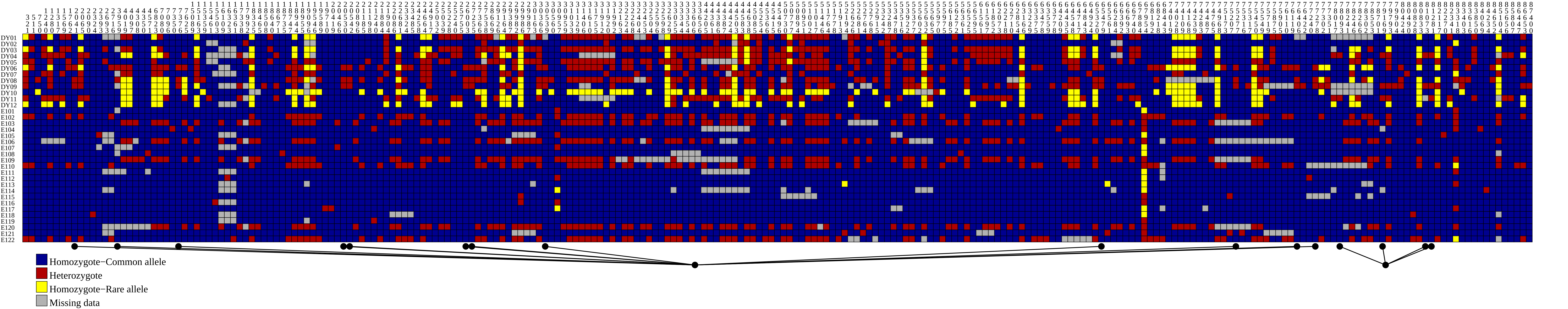

rad51l1, p−value: 0.4056

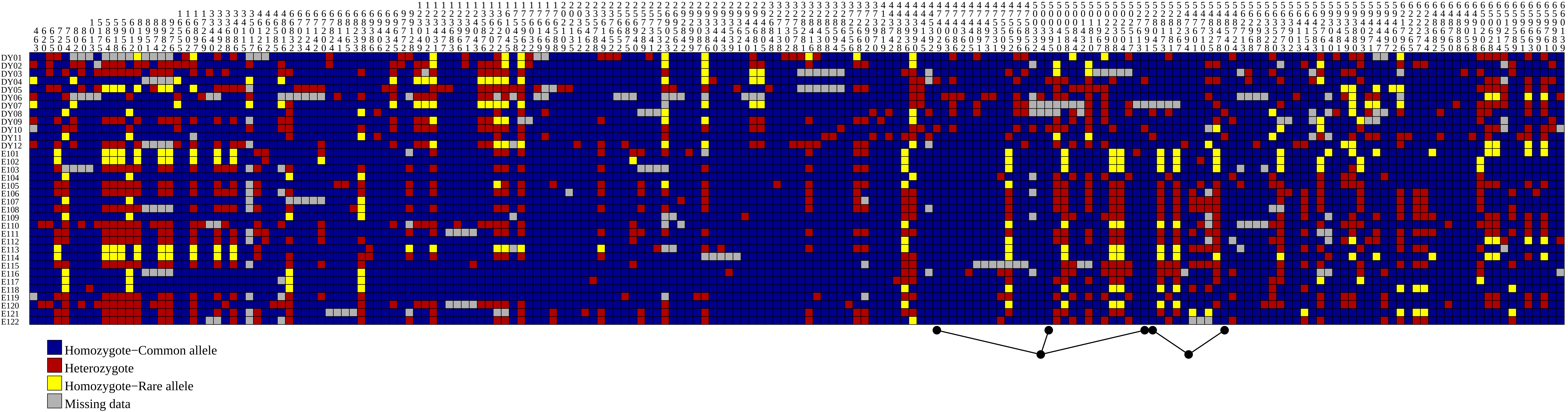

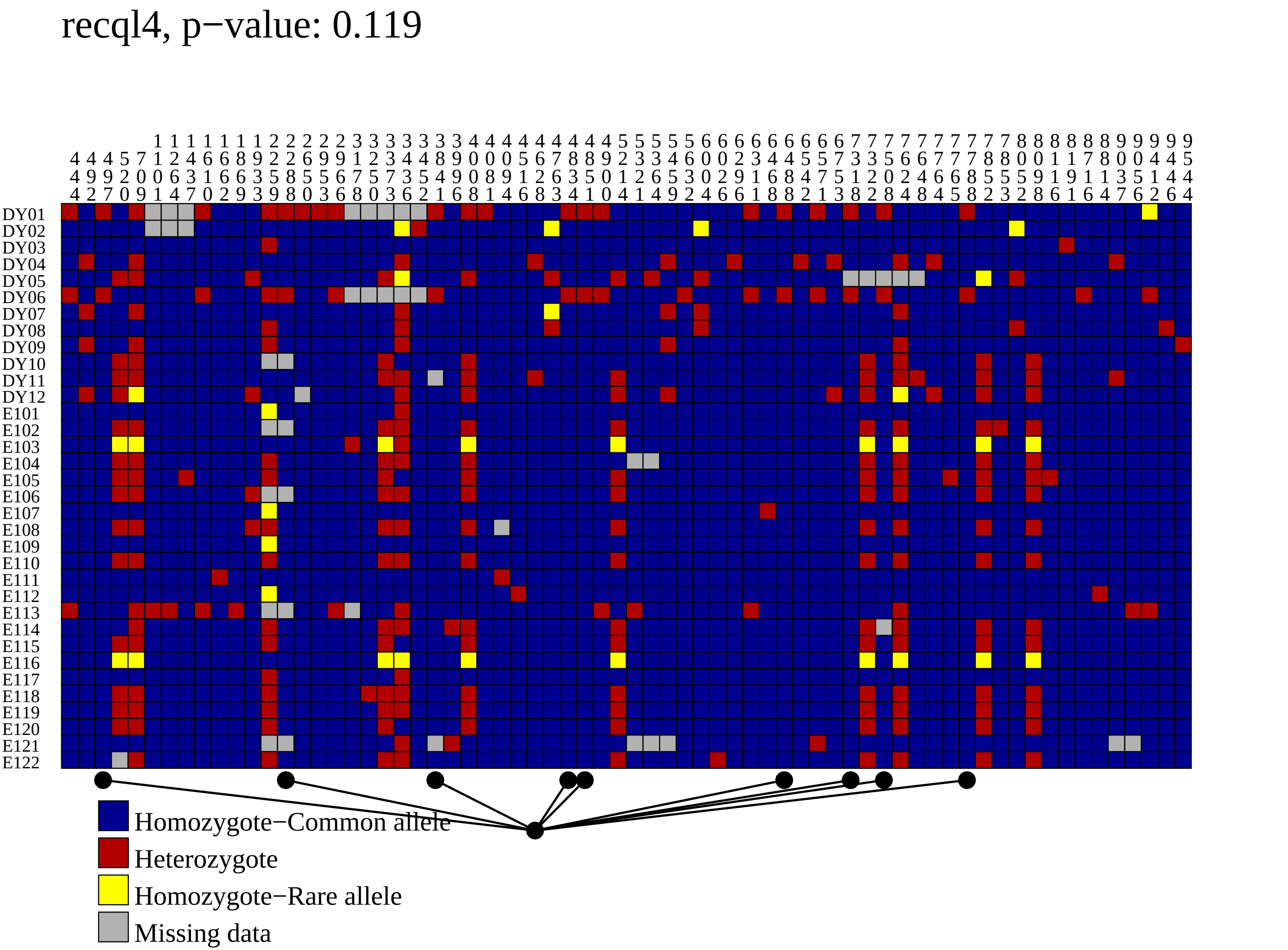
recql4, p−value: 0.119
444 492 497 520 709 1101 1164 1437 1610 1662 1869 1933 2259 2280 2650 2953 2966 3178 3250 3373 3436 3452 3841 3996 4008 4081 4094 4516 4628 4763 4834 4851 4900 5214 5321 5364 5459 5632 6004 6026 6296 6318 6468 6488 6542 6571 6753 7318 7322 7508 7624 7648 7764 7785 7788 7852 7853 8052 8098 8116 8191 8716 8814 9037 9056 9412 9446 9544
DY01
DY02
DY03
DY04
DY05
DY06
DY07
DY08
DY09
DY10
DY11
DY12
E101
E102
E103
E104
E105
E106
E107
E108
E109
E110
E111
E112
E113
E114
E115
E116
E117
E118
E119
E120
E121
E122
Homozygote−Common allele
Heterozygote
Homozygote−Rare allele
Missing data

rev1l, p−value: 0.6642

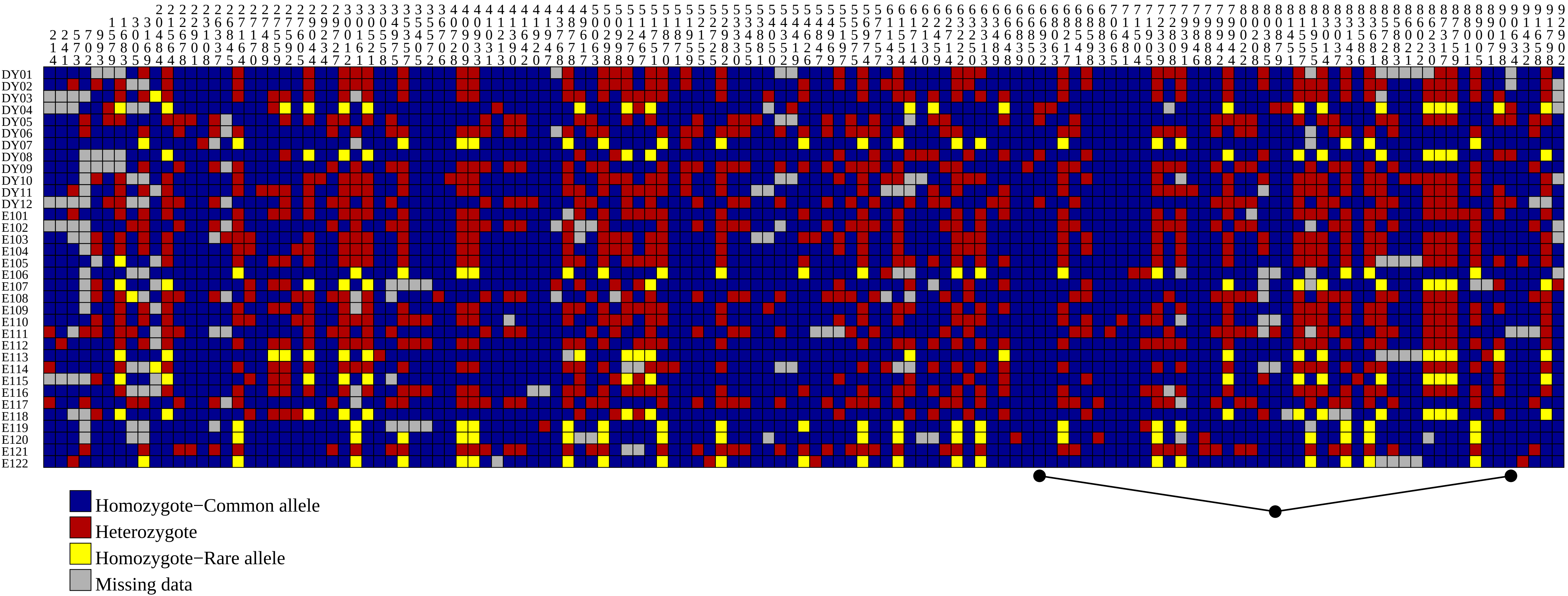

rpa2, p−value: 0.2302

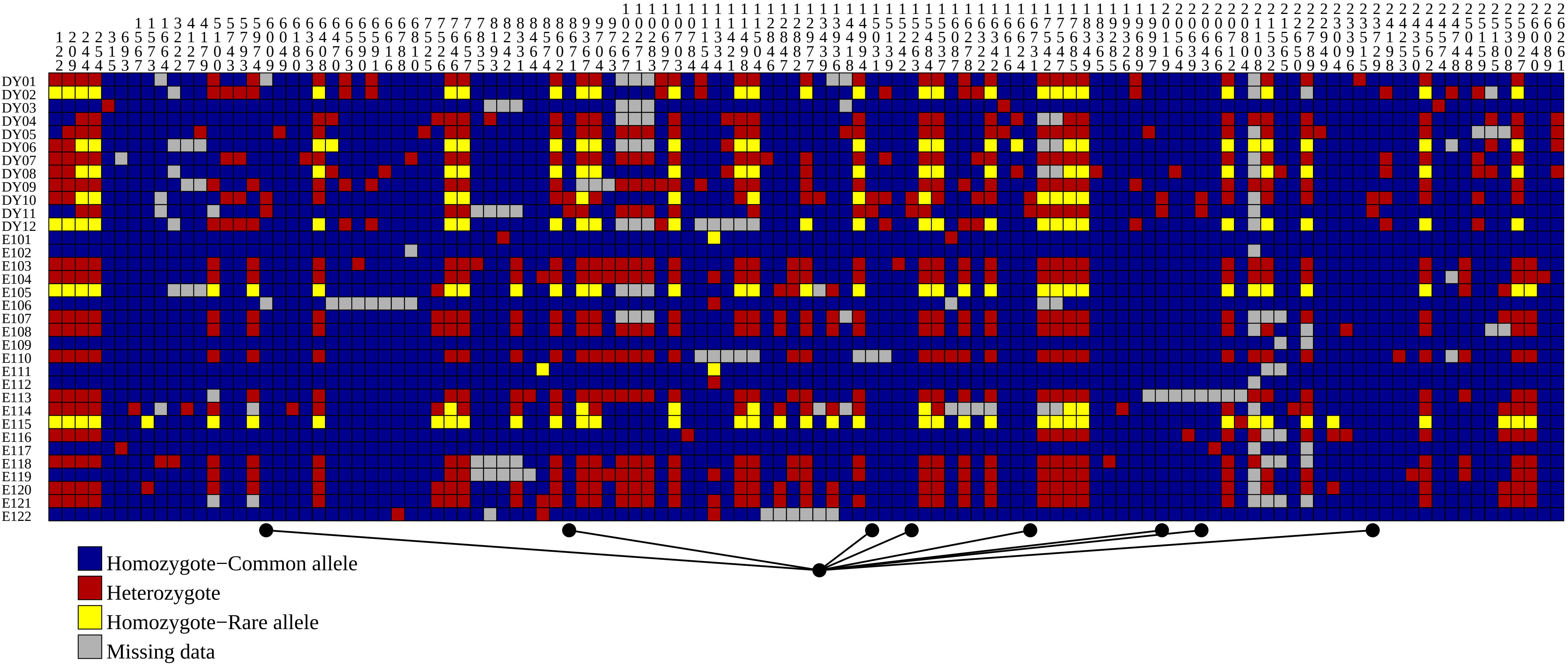

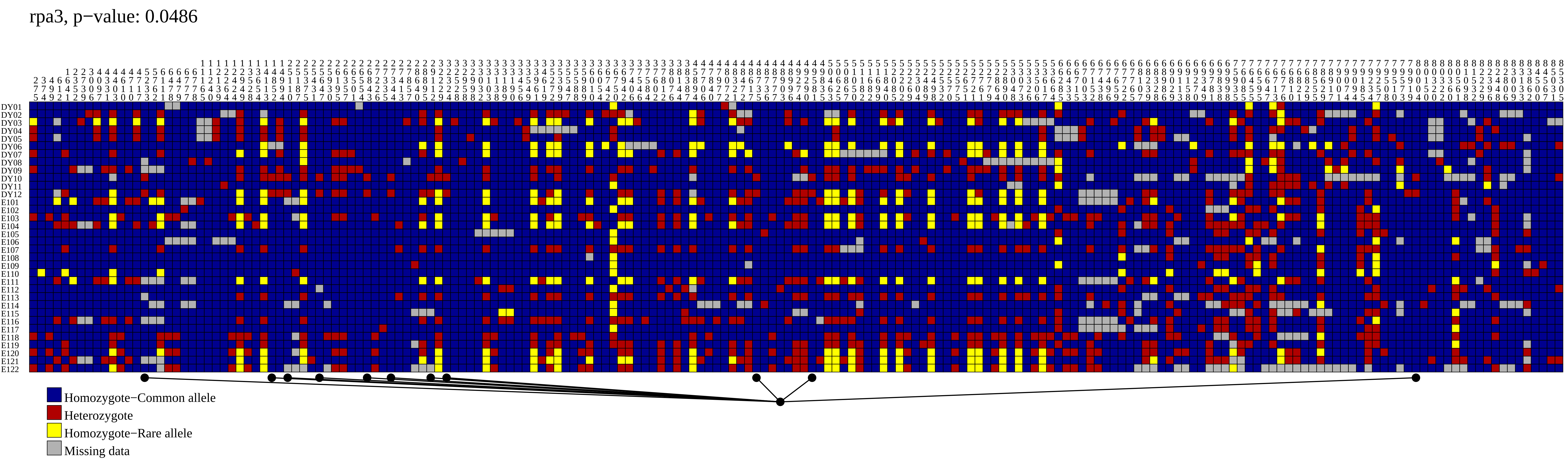
rpa3, p−value: 0.0486
DY01
DY02
DY03
DY04
DY05
DY06
DY07
DY08
DY09
DY10
DY11
DY12
E101
E102
E103
E104
E105
E106
E107
E108
E109
E110
E111
E112
E113
E114
E115
E116
E117
E118
E119
E120
E121
E122
Homozygote−Common allele
Heterozygote
Homozygote−Rare allele
Missing data

rrm2b, p−value: 0.7854

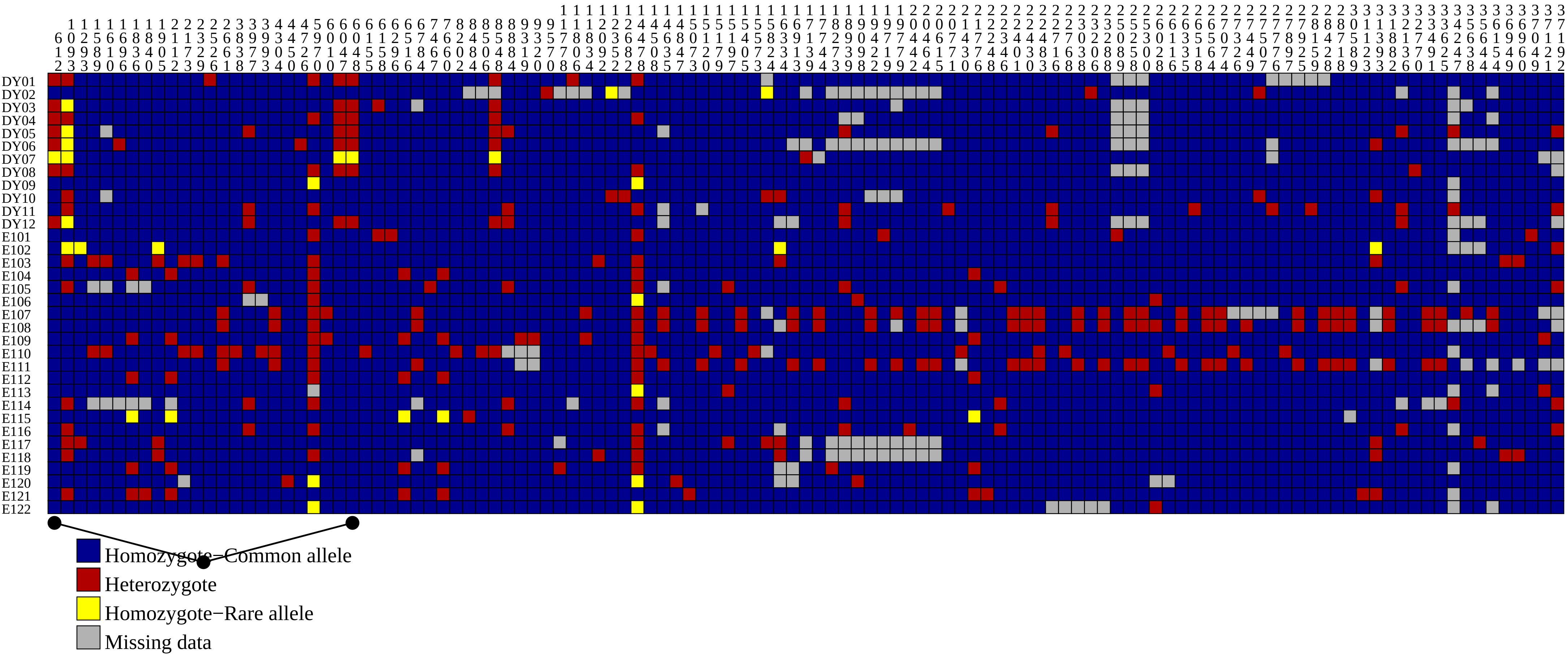

scara3, p−value: 0.2458

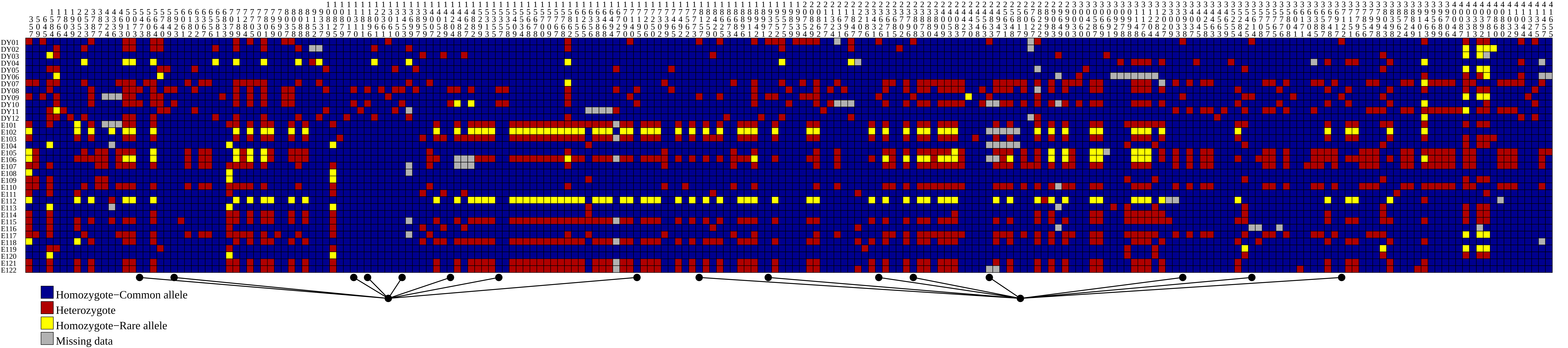

sepp1, p−value: 0.7278

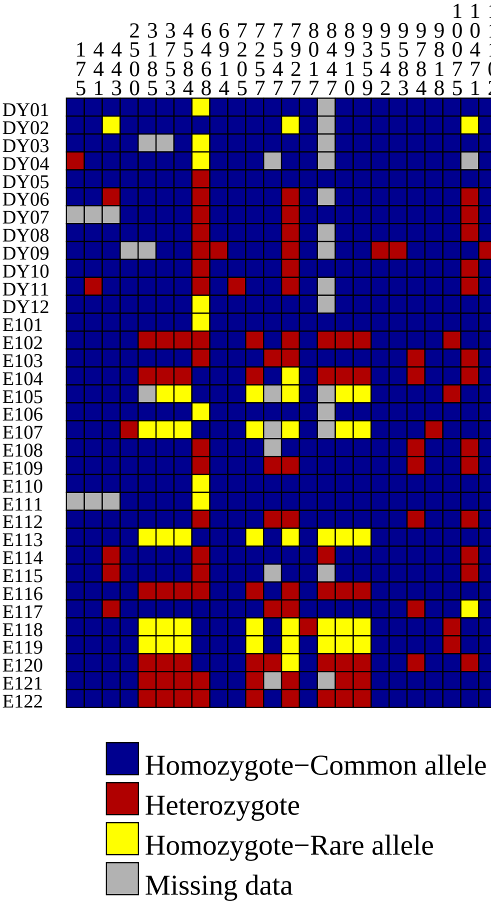

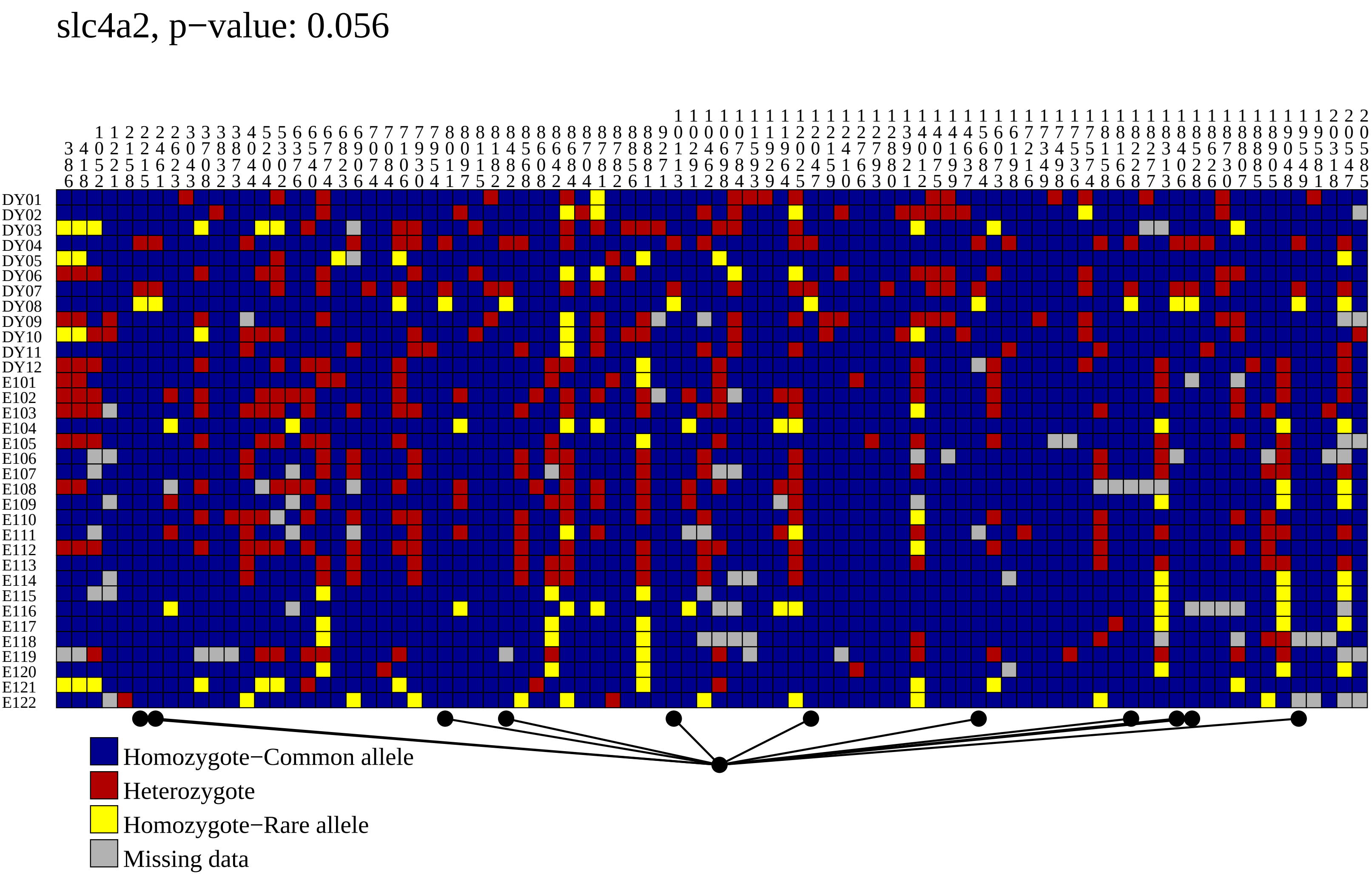

slc4a2, p−value: 0.056
Homozygote−Common allele
Heterozygote
Homozygote−Rare allele
Missing data

smarcb1, p−value: 0.2264

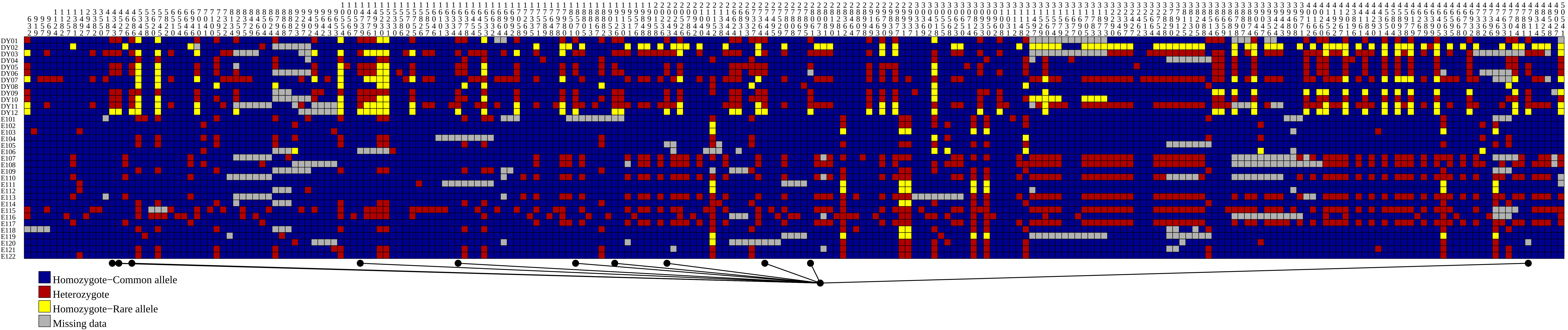

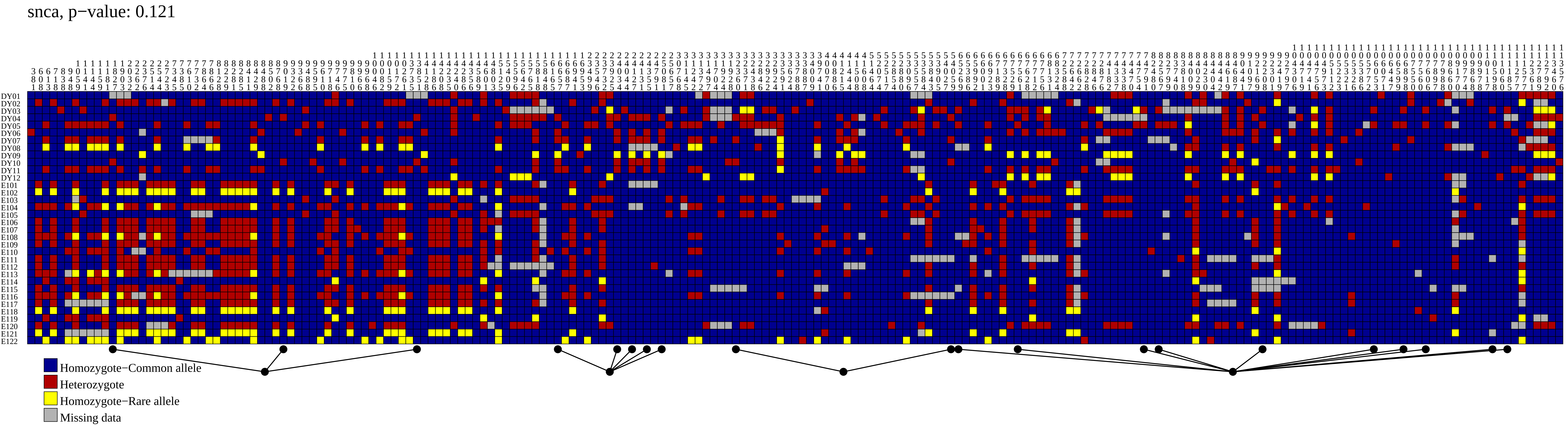
snca, p−value: 0.121
DY01
DY02
DY03
DY04
DY05
DY06
DY07
DY08
DY09
DY10
DY11
DY12
E101
E102
E103
E104
E105
E106
E107
E108
E109
E110
E111
E112
E113
E114
E115
E116
E117
E118
E119
E120
E121
E122
Homozygote−Common allele
Heterozygote
Homozygote−Rare allele
Missing data

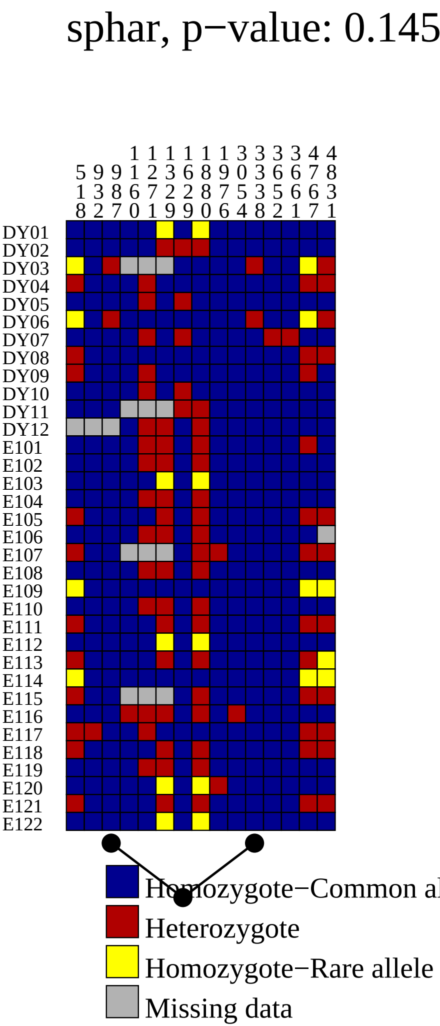

sphar, p−value: 0.145
518
932
987
1160
1271
1329
1629
1880
1976
3054
3338
3652
3661
4767
4831
DY01
DY02
DY03
DY04
DY05
DY06
DY07
DY08
DY09
DY10
DY11
DY12
E101
E102
E103
E104
E105
E106
E107
E108
E109
E110
E111
E112
E113
E114
E115
E116
E117
E118
E119
E120
E121
E122
Homozygote−Common al
Heterozygote
Homozygote−Rare allele
Missing data

spo11, p−value: 0.5298

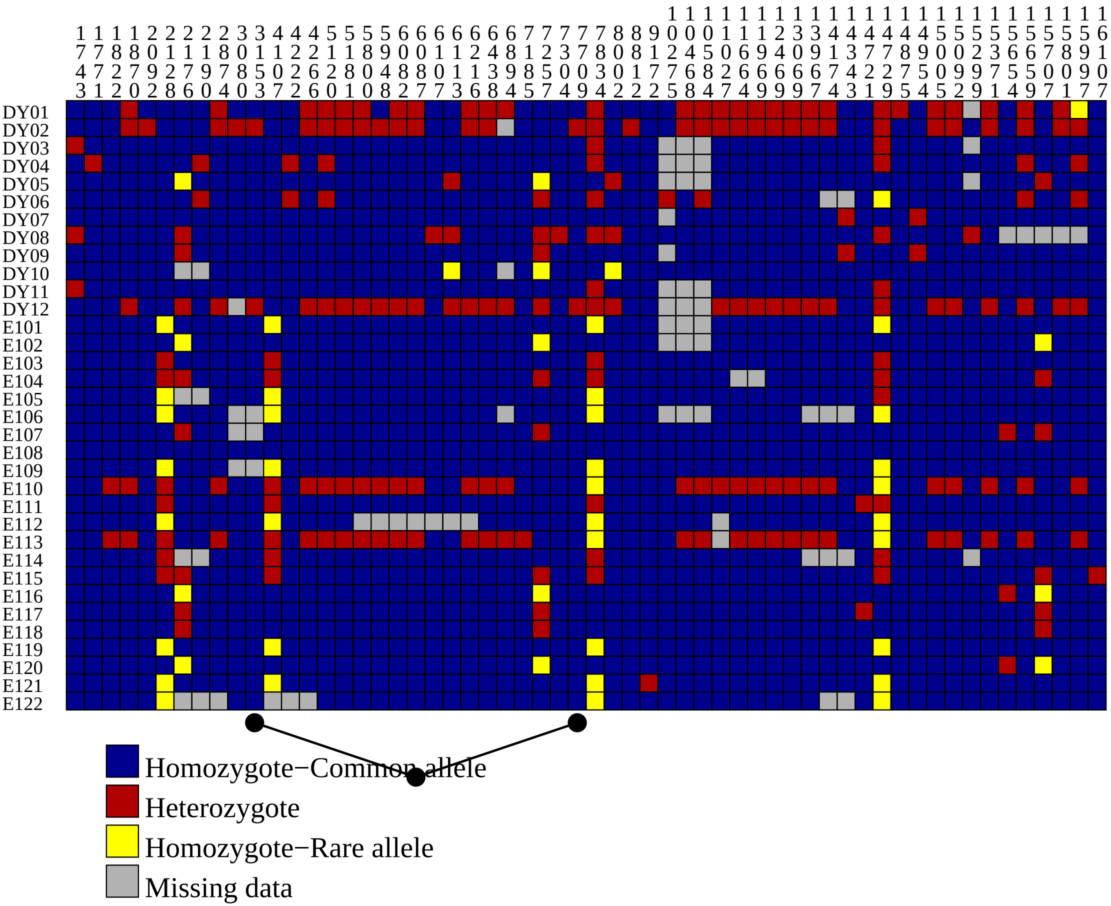

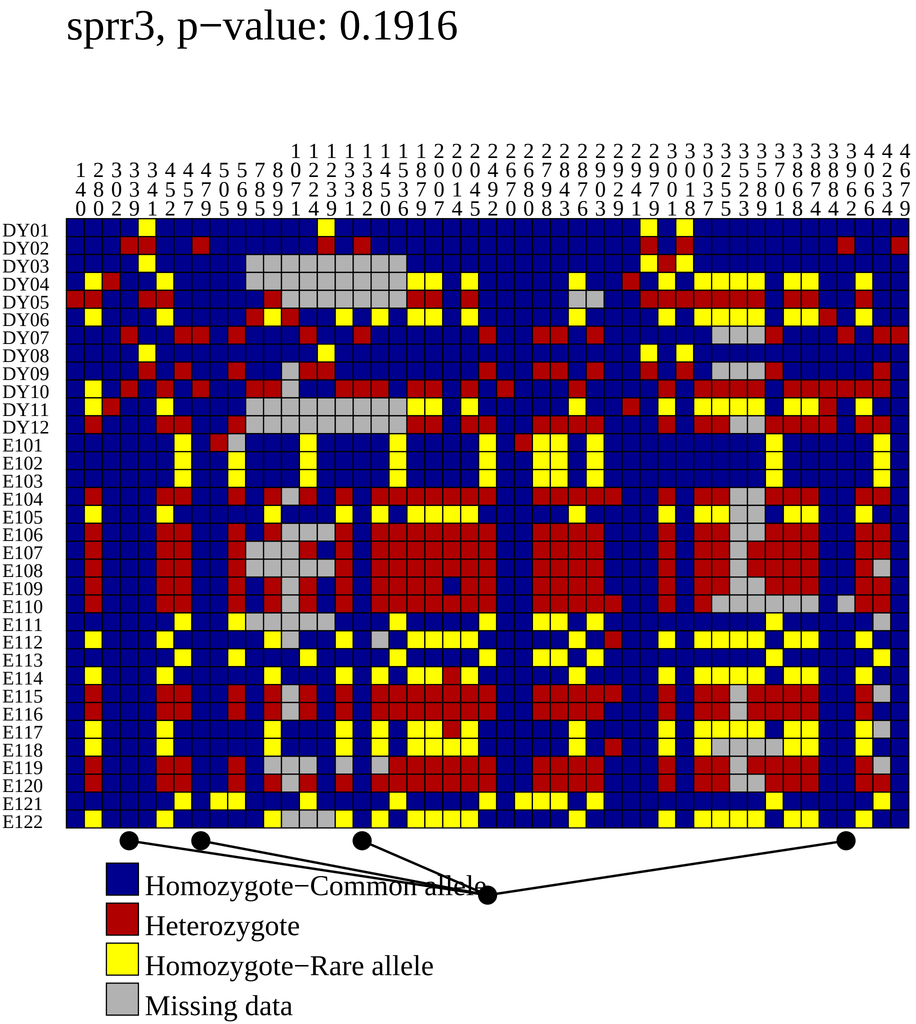

sprr3, p−value: 0.1916
140
280
302
339
341
452
457
479
505
569
785
899
1071
1224
1239
1331
1382
1450
1536
1879
2007
2014
2045
2492
2670
2680
2798
2843
2876
2903
2929
2941
2979
3001
3018
3037
3255
3523
3589
3701
3868
3874
3884
3962
4026
4234
4679
DY01
DY02
DY03
DY04
DY05
DY06
DY07
DY08
DY09
DY10
DY11
DY12
E101
E102
E103
E104
E105
E106
E107
E108
E109
E110
E111
E112
E113
E114
E115
E116
E117
E118
E119
E120
E121
E122
Homozygote−Common allele
Heterozygote
Homozygote−Rare allele
Missing data

srd5a2, p−value: 0.8714

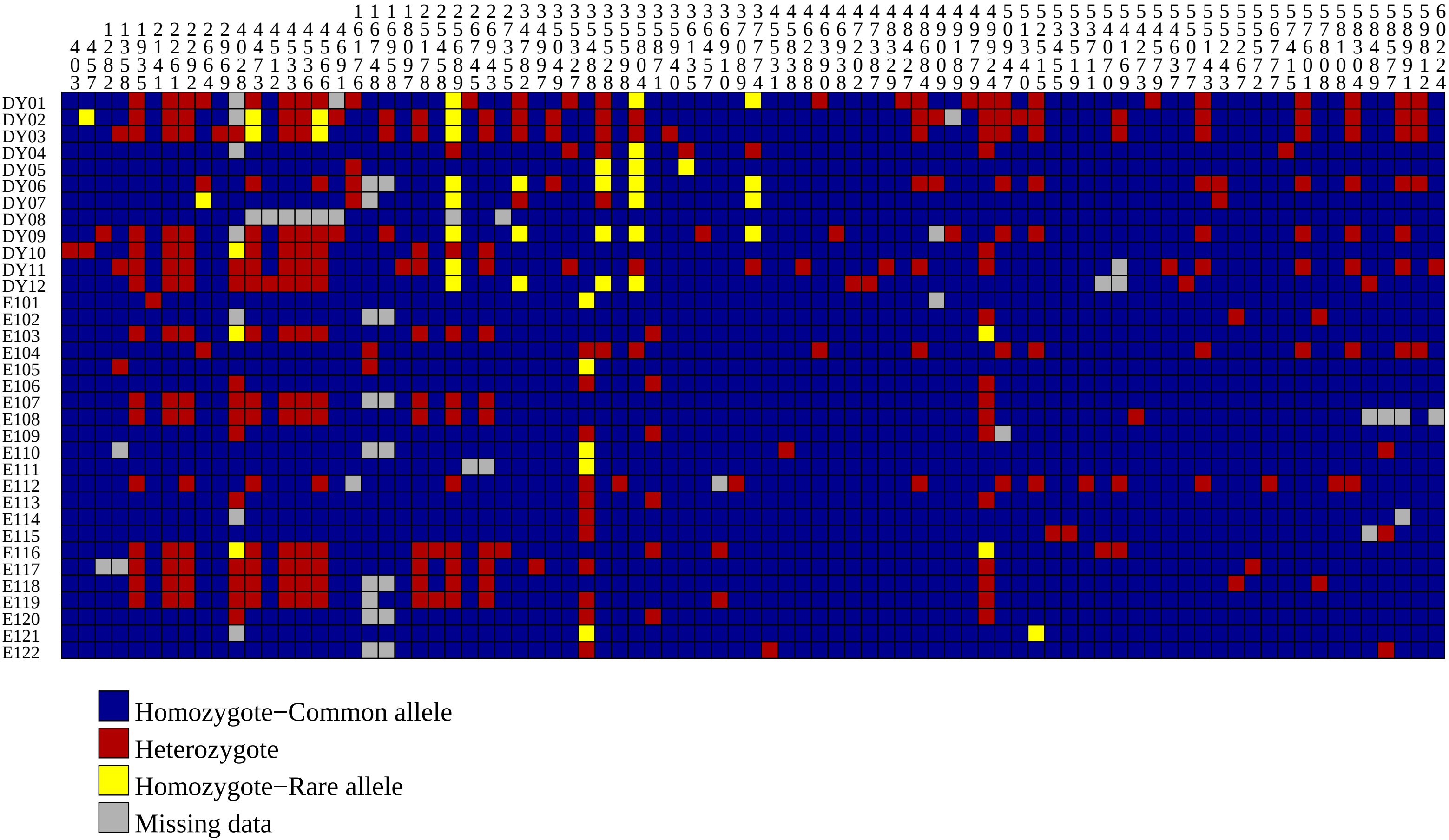

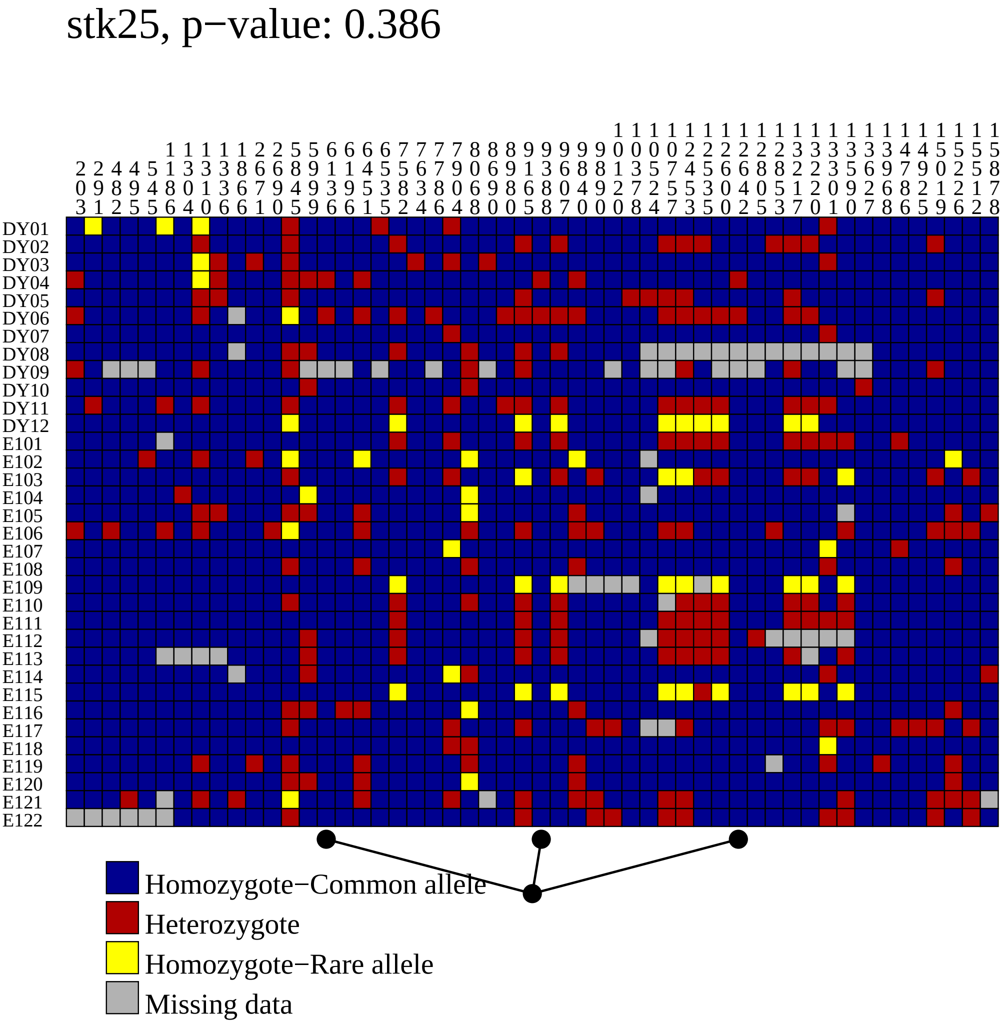
stk25, p−value: 0.386
203 291 482 495 545 1186 1304 1310 1336 1866 2671 2690 5845 5999 6136 6196 6451 6535 7582 7634 7786 7904 8068 8690 8980 9165 9388 9607 9840 9890 10120 10378 10524 10757 12453 12535 12600 12602 12805 12853 13217 13220 13301 13590 13627 13968 14786 14925 15019 15226 15512 15878
DY01
DY02
DY03
DY04
DY05
DY06
DY07
DY08
DY09
DY10
DY11
DY12
E101
E102
E103
E104
E105
E106
E107
E108
E109
E110
E111
E112
E113
E114
E115
E116
E117
E118
E119
E120
E121
E122
Homozygote−Common allele
Heterozygote
Homozygote−Rare allele
Missing data

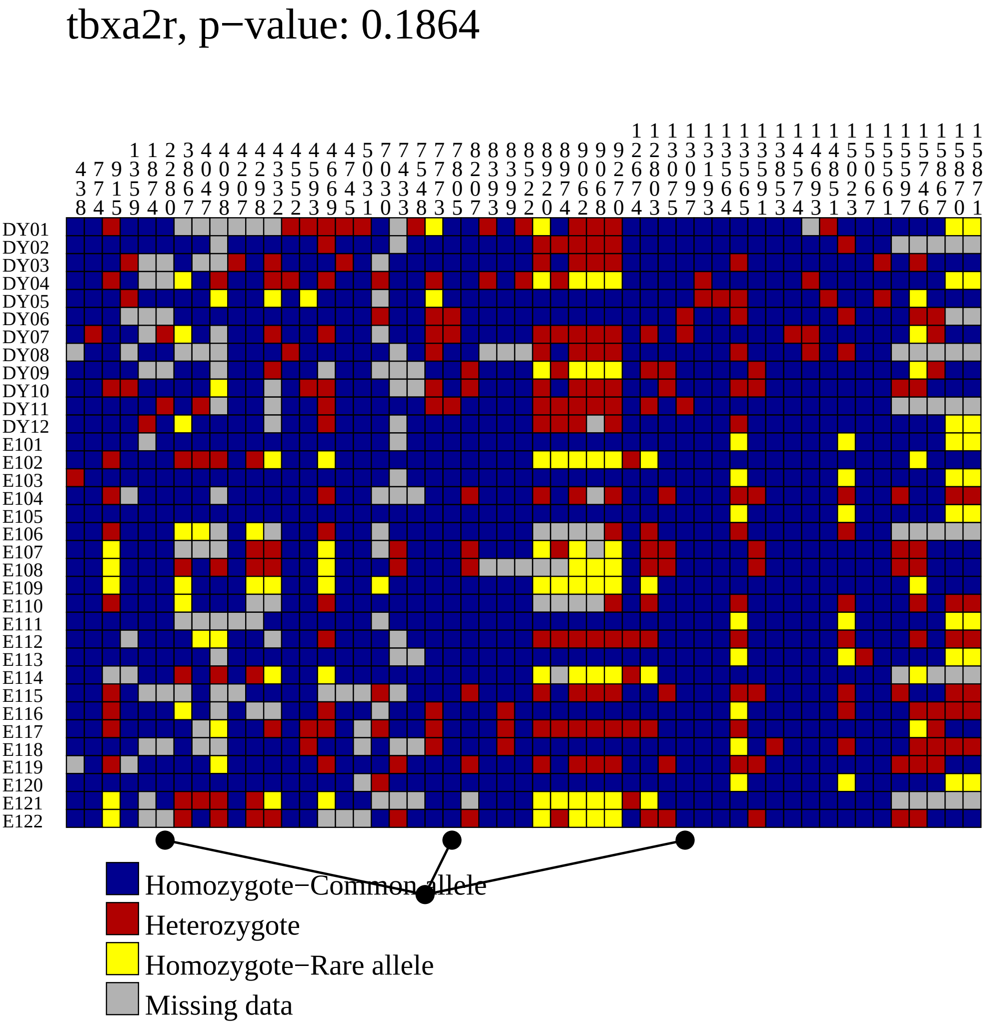

tbxa2r, p−value: 0.1864
Homozygote−Common allele
Heterozygote
Homozygote−Rare allele
Missing data

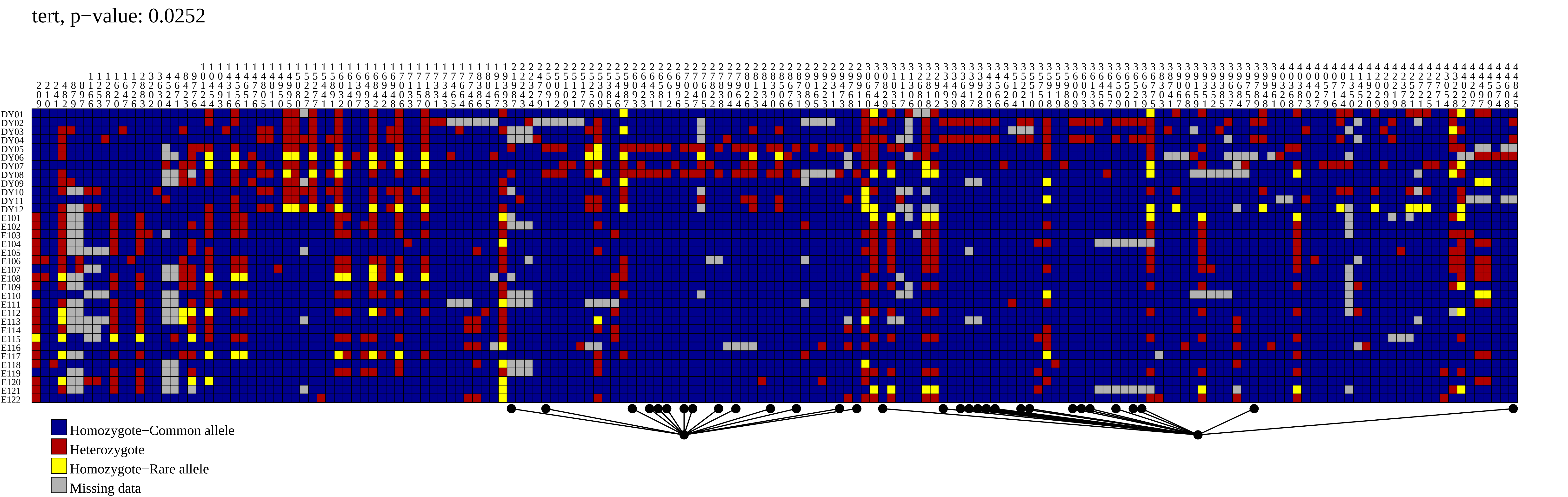
tert, p−value: 0.0252
DY01
DY02
DY03
DY04
DY05
DY06
DY07
DY08
DY09
DY10
DY11
DY12
E101
E102
E103
E104
E105
E106
E107
E108
E109
E110
E111
E112
E113
E114
E115
E116
E117
E118
E119
E120
E121
E122
Homozygote−Common allele
Heterozygote
Homozygote−Rare allele
Missing data

tjp1, p−value: 0.0052

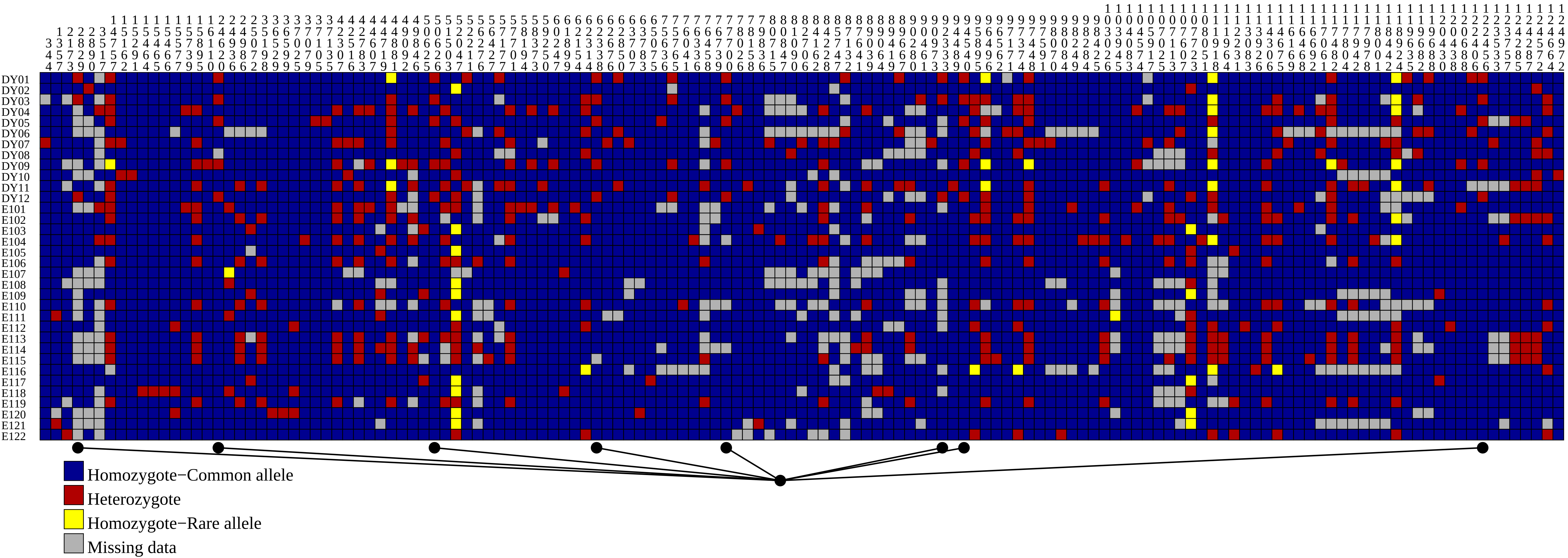

tnfrsf4, p−value: 0.4562

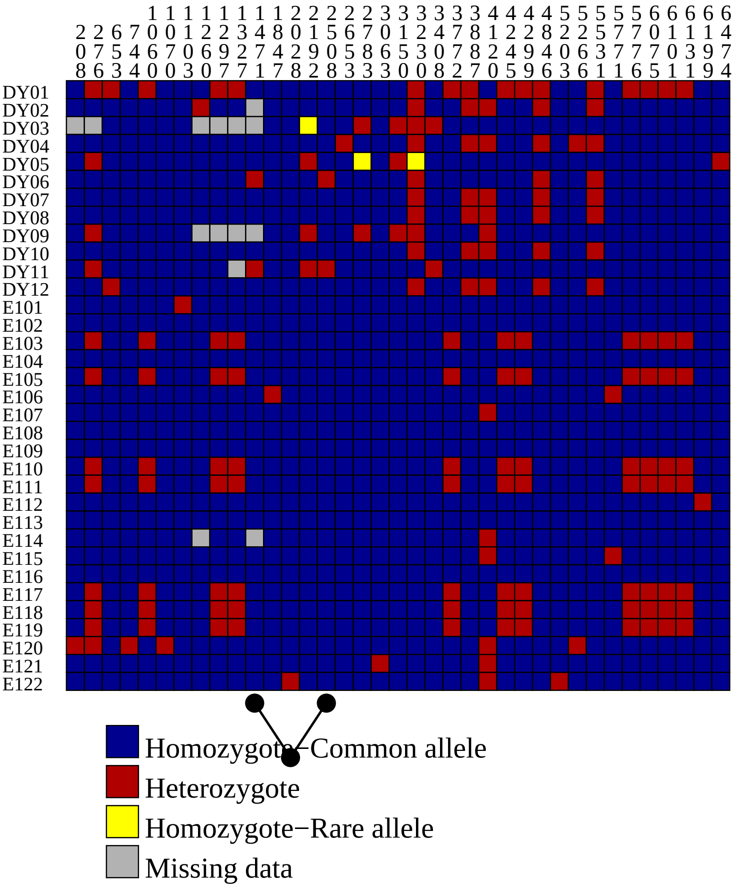

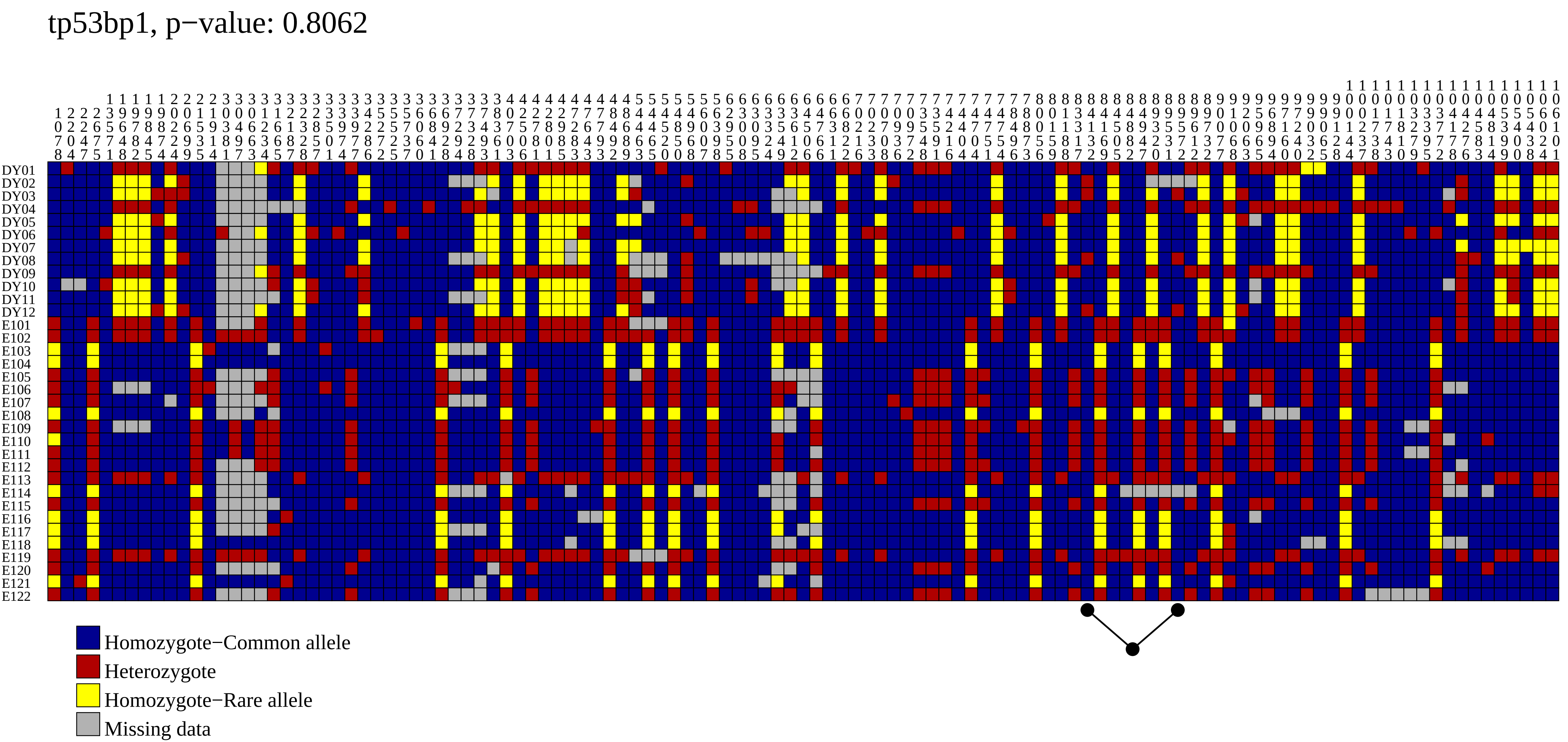
tp53bp1, p−value: 0.8062
DY01
DY02
DY03
DY04
DY05
DY06
DY07
DY08
DY09
DY10
DY11
DY12
E101
E102
E103
E104
E105
E106
E107
E108
E109
E110
E111
E112
E113
E114
E115
E116
E117
E118
E119
E120
E121
E122
Homozygote−Common allele
Heterozygote
Homozygote−Rare allele
Missing data

tp53i3, p−value: 0.6318

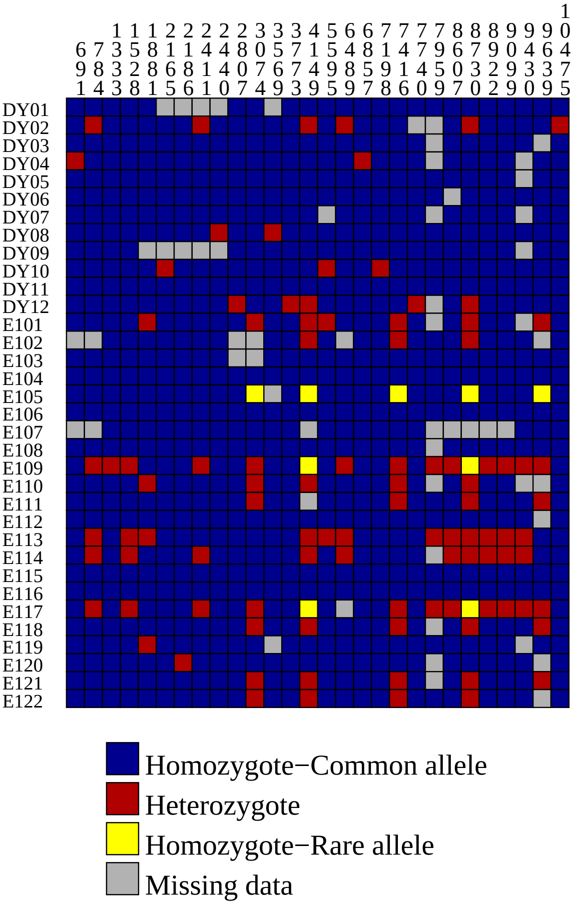

tpo, p−value: 0.0298

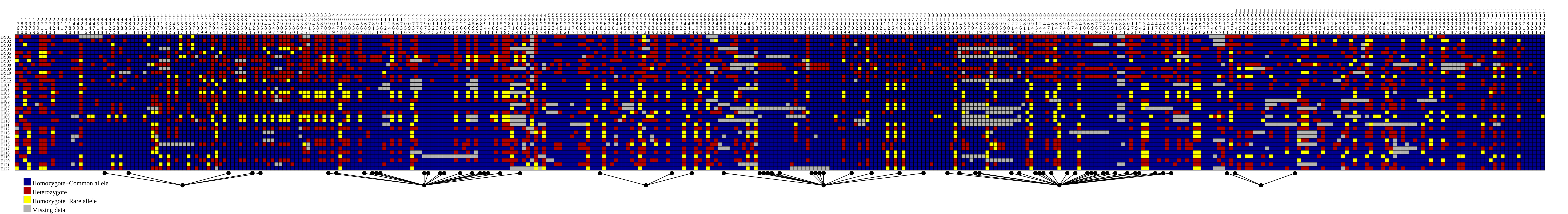

trpm2, p−value: 0.119

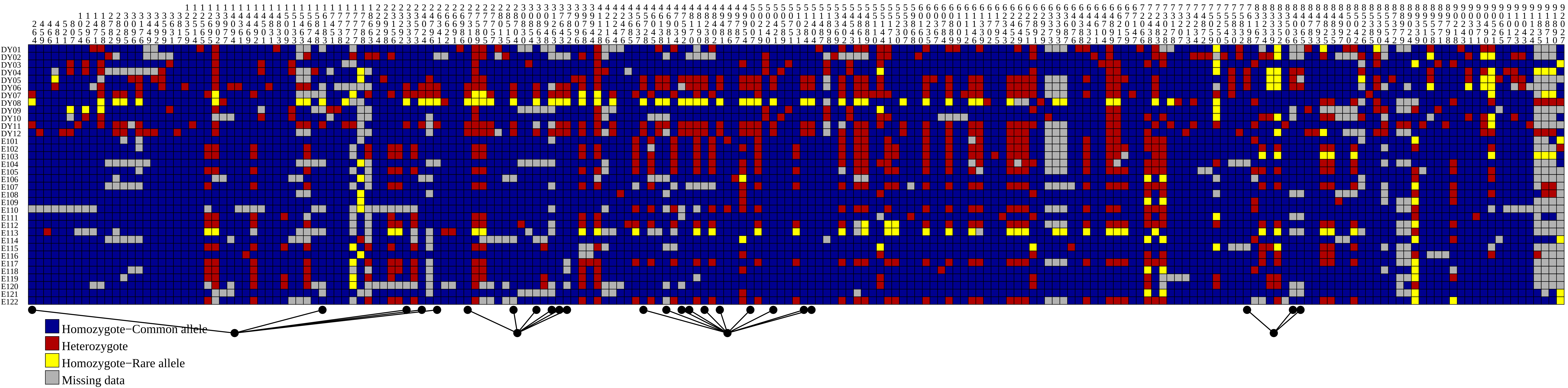

tuba1, p−value: 0.083

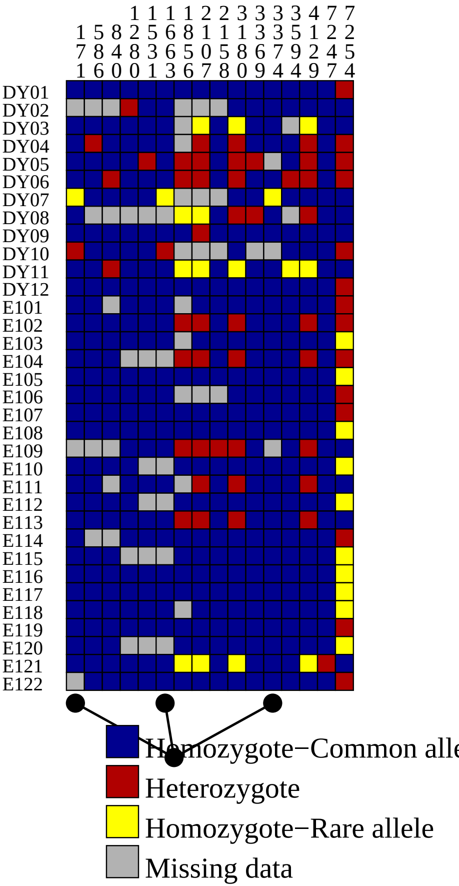

txnrd1, p−value: 0.1626

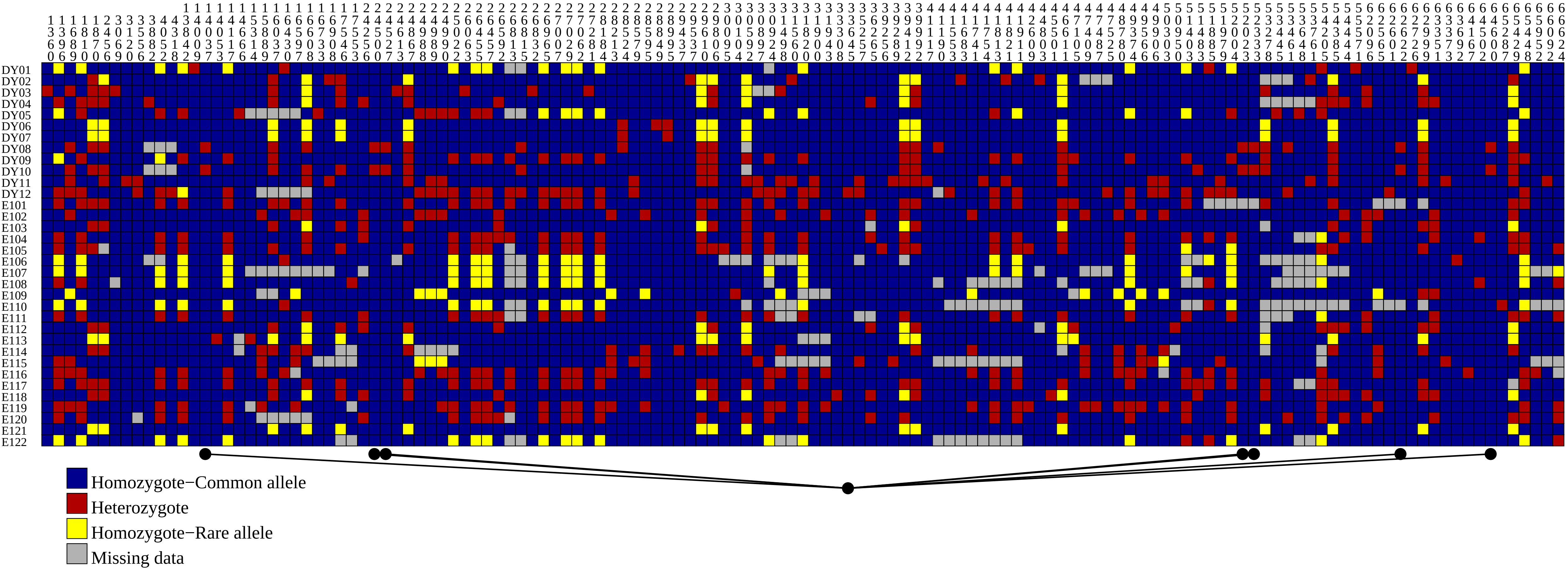

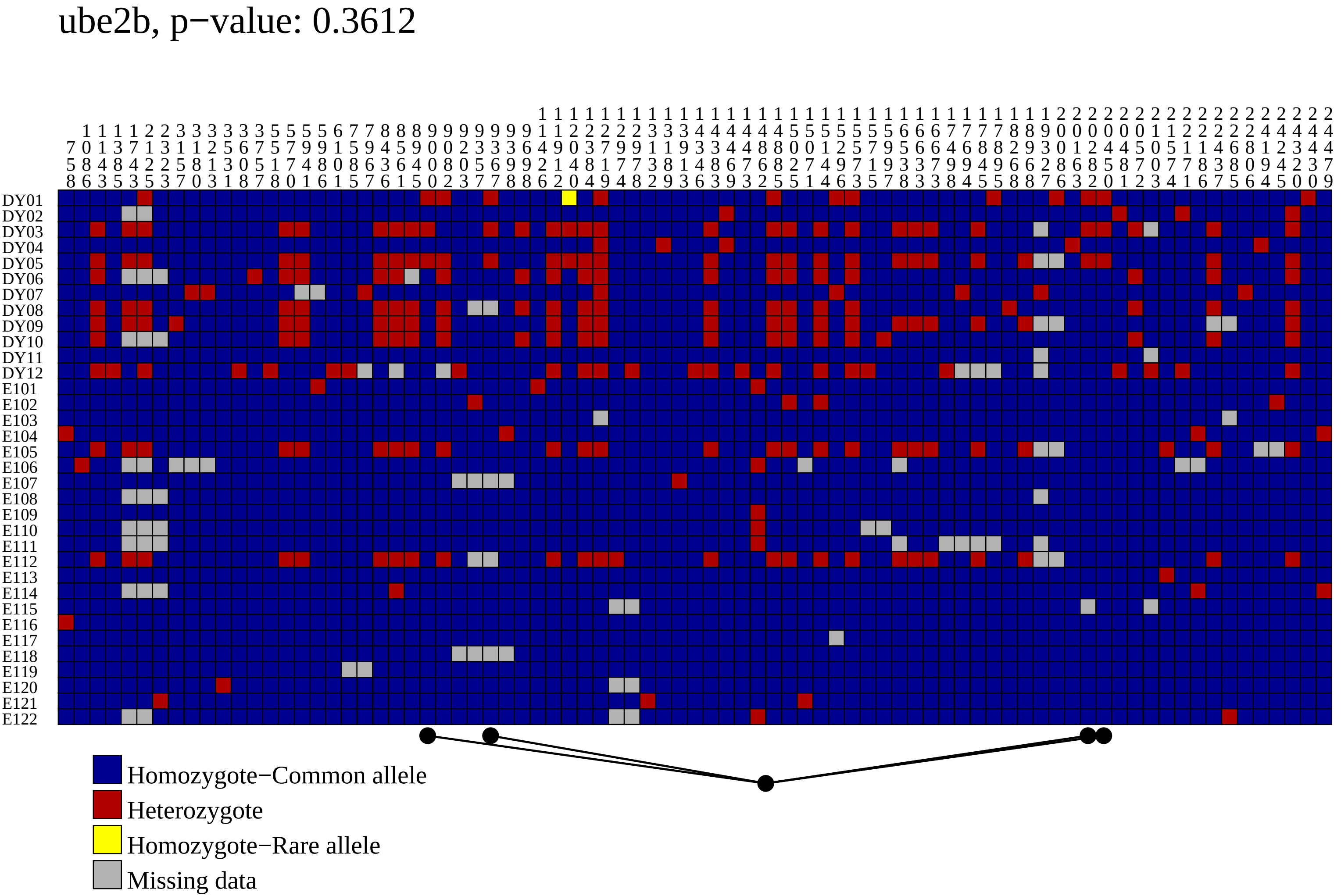

ube2b, p−value: 0.3612
Homozygote−Common allele
Heterozygote
Homozygote−Rare allele
Missing data

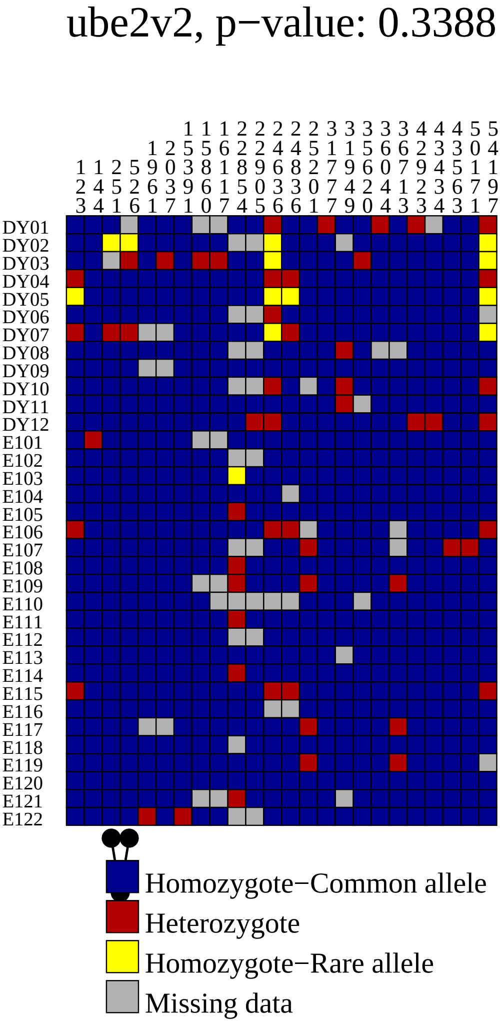
ube2v2, p−value: 0.3388
123
144
251
526
1961
2037
15391
15810
16117
22854
22905
24636
24836
25201
31777
31949
35620
36044
36713
42923
43434
43563
50171
54197
DY01
DY02
DY03
DY04
DY05
DY06
DY07
DY08
DY09
DY10
DY11
DY12
E101
E102
E103
E104
E105
E106
E107
E108
E109
E110
E111
E112
E113
E114
E115
E116
E117
E118
E119
E120
E121
E122
Homozygote−Common allele
Heterozygote
Homozygote−Rare allele
Missing data

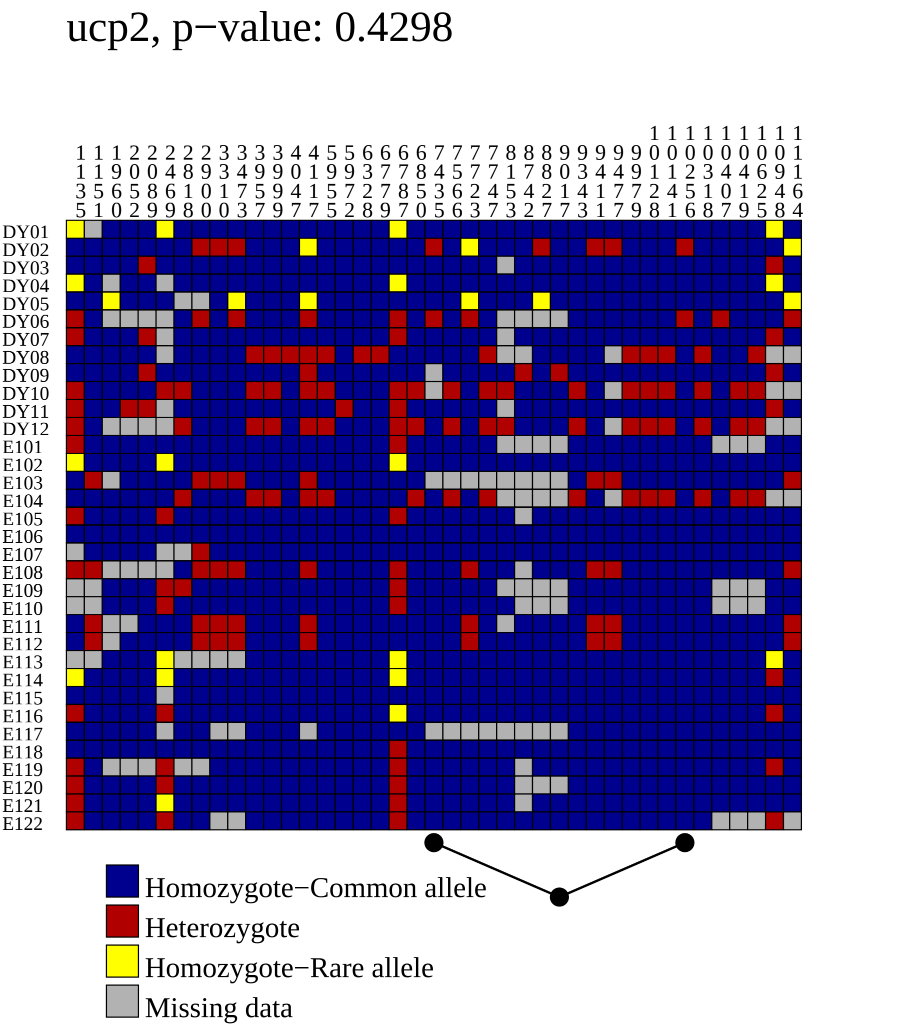
ucp2, p−value: 0.4298
1135 1151 1960 2052 2089 2469 2818 2900 3310 3473 3957 3999 4047 4117 5955 5972 6328 6779 6787 6850 7435 7566 7723 7747 8153 8742 8827 9017 9343 9411 9477 9979 10128 10141 10256 10318 10407 10419 10625 10948 11164
DY01
DY02
DY03
DY04
DY05
DY06
DY07
DY08
DY09
DY10
DY11
DY12
E101
E102
E103
E104
E105
E106
E107
E108
E109
E110
E111
E112
E113
E114
E115
E116
E117
E118
E119
E120
E121
E122
Homozygote−Common allele
Heterozygote
Homozygote−Rare allele
Missing data

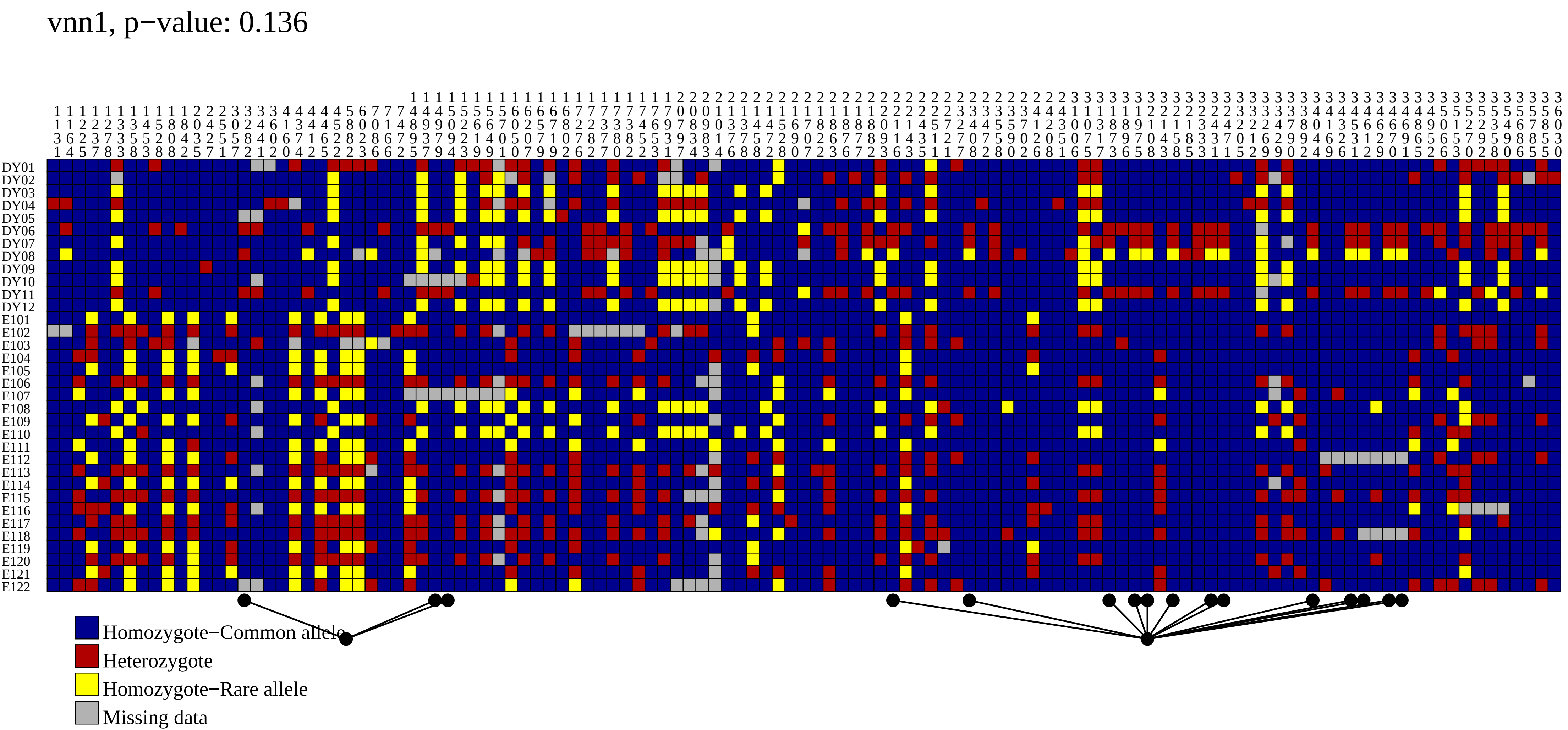
vnn1, p−value: 0.136
DY01
DY02
DY03
DY04
DY05
DY06
DY07
DY08
DY09
DY10
DY11
DY12
E101
E102
E103
E104
E105
E106
E107
E108
E109
E110
E111
E112
E113
E114
E115
E116
E117
E118
E119
E120
E121
E122
Homozygote−Common allele
Heterozygote
Homozygote−Rare allele
Missing data

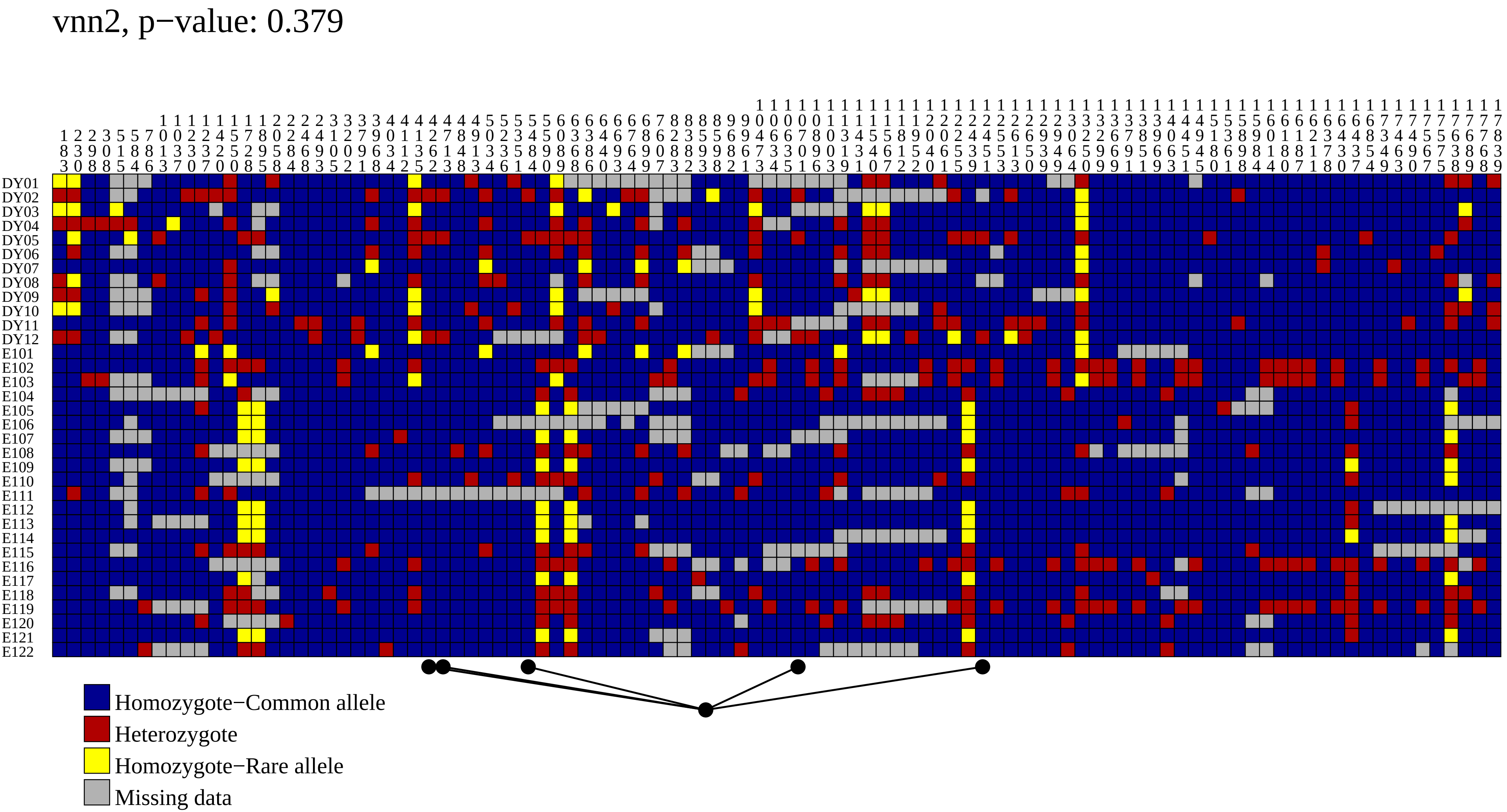

vnn2, p−value: 0.379
DY01
DY02
DY03
DY04
DY05
DY06
DY07
DY08
DY09
DY10
DY11
DY12
E101
E102
E103
E104
E105
E106
E107
E108
E109
E110
E111
E112
E113
E114
E115
E116
E117
E118
E119
E120
E121
E122
Homozygote−Common allele
Heterozygote
Homozygote−Rare allele
Missing data

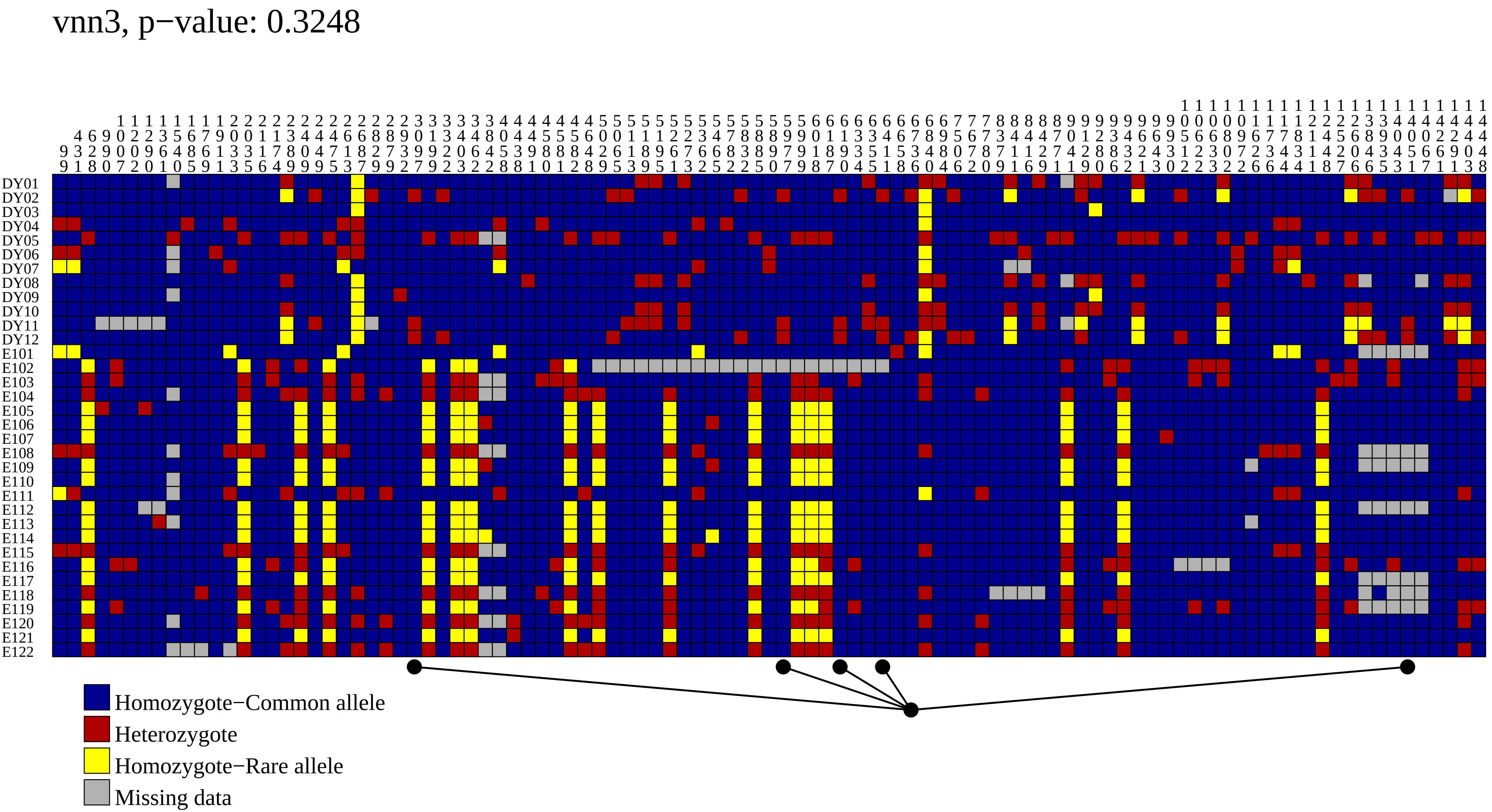
vnn3, p−value: 0.3248
Homozygote−Common allele
Heterozygote
Homozygote−Rare allele
Missing data

xdh, p−value: 0.1856

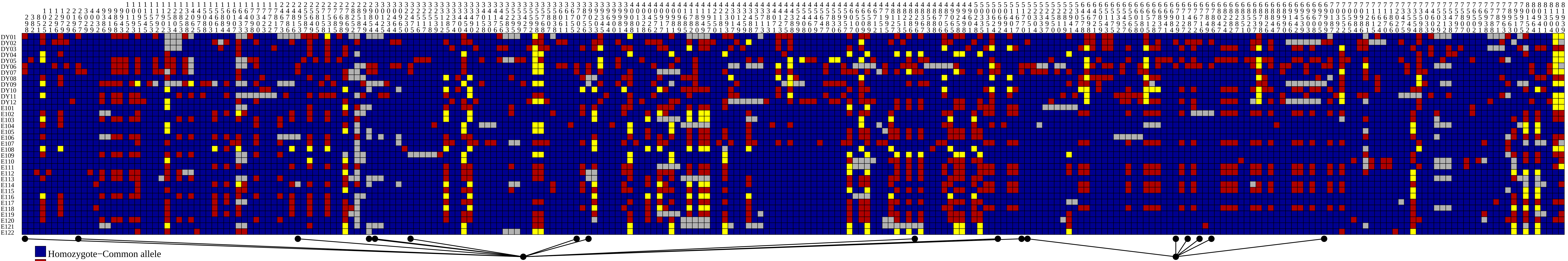

Homozygote−Common allele
Heterozygote
Homozygote−Rare allele
Missing data

xrcc4, p−value: 0.0012

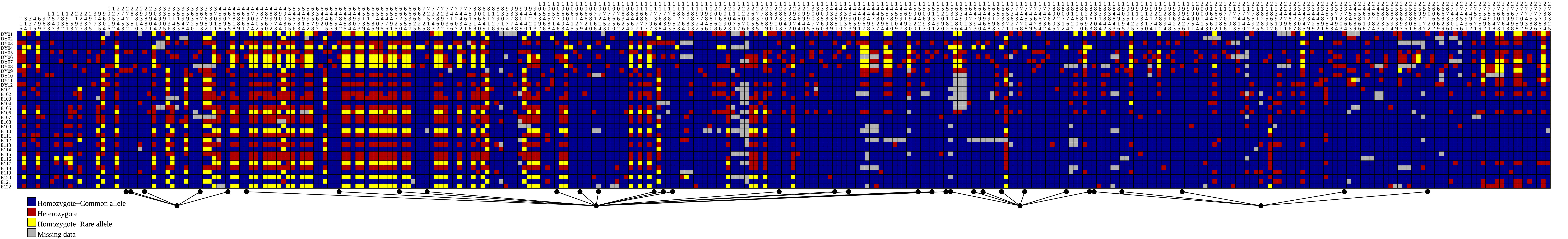

Supplement: Figure S6 — (2.8 MB PDF) [file pgen.0020105.sg006.pdf]
