# Supplementary material for: Possible Ancestral Structure in Human Populations
Source: PLoS Genet. 2006 Jul 28;2(7):e105. doi: 10.1371/journal.pgen.0020105 (PMC1523253; doi:10.1371/journal.pgen.0020105)
Supplement: Protocol S1 — (28 KB PDF) [file pgen.0020105.sd001.pdf]

Protocol S1: *ms* command lines

The *ms* command line that we use to generate a 50 kb sequence using our best fitting model is:

> ms 68 1 -t 1882. -r 3764 50000 -I 2 24 44 160 -g 1 4303.9 -g 2 41228 -eg 0.00107 1 0. -eg 0.0001117 2 0. -en 0.000775 2 9.1e-05 -en 0.000785 2 0.01 -ej 0.0016 2 1

With the scaling we use the current population size is 100 fold the ancestral population size so 0.001 unit of times corresponds to 0.1 normalized with the ancestral population size (ie $0.1 \times 4 \times 10,000$ generations $=$ $80,000$ yrs). Note that we assume that the recombination rate ρ is randomly distributed and therefore the sixth argument of *ms* is chosen at random (in this case $f = \rho/\theta = 2$).

With a rate of admixture set to 5% the new *ms* command line becomes:

> ms 68 1 -t 1792 -r 3584 50000 -I 2 24 44 160 -g 1 3704.88 -g 2 71464.5 -eg 0.001243 1 0. -eg 6.444e-05 2 0. -es 0.00059 2 0.95 -en 0.00059 3 0.01 -en 0.000775 2 7.14286e-05 -en 0.000785 2 0.01 -ej 0.0016 2 1 -ej 0.005 3 1

Note on the computation of F_{ST}

There are several definitions of F_{ST} in the literature. In this paper we follow (1). Precisely, for a pair of population (Yoruba and CEPH in this paper), we define:

$$F_{ST} = \frac{\Pi_{Between} - \Pi_{Within}}{\Pi_{Within}}$$

To compute Π_{Within} one typically uses all pairs of individuals within each populations. However, if the sample sizes n_1 and n_2 are not equal, this formula puts more weight on the population with the largest sample size. Consequently with this definition the value of Π_{Within} (and also F_{ST}) is affected by the difference between n_1 and n_2, even if both sample sizes are large. This is a problem if one wants to compare F_{ST} across situations where the sample sizes vary. To avoid that issue we define Π_{Within} in the following way:

$$\Pi_{Within} = \frac{\Pi_1 + \Pi_2}{2}$$

where Π_1 and Π_2 designate the average number of pairwise differences in each population. With this definition F_{ST} reaches a limiting value when the sample sizes n_1 and n_2 become large and it becomes possible to compare F_{ST} values even when sample sizes differ.

References

[1] Hudson RR, Slatkin M, Maddison WP (1992) Estimation of levels of gene flow from DNA sequence data. Genetics 132:583–589.
