# Supplementary material for: Possible Ancestral Structure in Human Populations
Source: PLoS Genet. 2006 Jul 28;2(7):e105. doi: 10.1371/journal.pgen.0020105 (PMC1523253; doi:10.1371/journal.pgen.0020105)
Supplement: Table S1 — (21 KB PDF) [file pgen.0020105.st001.pdf]

CEPH

Name	p-value	Function
rrm2b	0.0018	Ribonucleotide reductase M2 B (TP53 inducible)
tp53bp1	0.0026	Tumor protein p53 binding protein, 1
rpa3	0.0052	Replication protein A3, 14kDa
oxr1	0.0094	Oxidation resistance 1
tp53i3	0.0132	Tumor protein p53 inducible protein 3
sepp1	0.014	Selenoprotein P, plasma, 1
vnn2	0.0162	Vanin 2
birc2	0.0178	Baculoviral IAP repeat-containing 2
fdxr	0.0266	Ferredoxin reductase
gsta4	0.0294	Glutathione S-transferase A4
scara3	0.0322	Scavenger receptor class A, member 3
fgf5	0.0332	Fibroblast growth factor 5
adh1c	0.0346	Alcohol dehydrogenase 1C (class I), gamma polypeptide
rad18	0.0362	RAD18 homolog (S. cerevisiae)
chrna4	0.0388	Cholinergic receptor, nicotinic, alpha polypeptide 4
adh1b	0.04	Alcohol dehydrogenase IB (class I), beta polypeptide
fmo5	0.0404	Flavin containing monooxygenase 5
adh5	0.0404	Alcohol dehydrogenase 5 (class III), chi polypeptide
odc1	0.0416	Ornithine decarboxylase 1
adh6	0.042	Alcohol dehydrogenase 6 (class V)
mmp9	0.0422	Matrix metalloproteinase 9 (gelatinase B, 92kDa gelatinase, 92kDa type IV collagenase)
txnrd1	0.044	Thioredoxin reductase 1
abl2	0.045	V-abl Abelson murine leukemia viral oncogene homolog 2 (arg, Abelson-related gene)
cyp1a2	0.0486	Cytochrome P450, family 1, subfamily A, polypeptide 2
mmp12	0.0508	Matrix metalloproteinase 12 (macrophage elastase)
polm	0.055	Polymerase (DNA directed), mu
vnn3	0.06	Vanin 3
csk	0.0644	C-src tyrosine kinase
adh1a	0.0646	Alcohol dehydrogenase 1A (class I), alpha polypeptide
mad2l2	0.0736	MAD2 mitotic arrest deficient-like 2 (yeast)
mmp8	0.0828	Matrix metalloproteinase 8 (neutrophil collagenase)
bace1	0.0914	Beta-site APP-cleaving enzyme 1
fmo3	0.0946	Flavin containing monooxygenase 3
ube2v2	0.1038	Ubiquitin-conjugating enzyme E2 variant 2
ppib	0.1044	Peptidylprolyl isomerase B (cyclophilin B)
tpo	0.1076	Thyroid peroxidase
nudt1	0.11	Nudix (nucleoside diphosphate linked moiety X)-type motif 1
foxm1	0.1108	Forkhead box M1
gpx6	0.1118	Glutathione peroxidase 6 (olfactory)
mb	0.1122	Myoglobin
app	0.1276	Amyloid beta (A4) precursor protein (protease nexin-II, Alzheimer disease)
mmp16	0.144	Matrix metalloproteinase 16 (membrane-inserted)
dclre1b	0.1512	DNA cross-link repair 1B (PSO2 homolog, S. cerevisiae)
fbp1	0.1858	Fructose-1,6-bisphosphatase 1
pax3	0.1876	Paired box gene 3 (Waardenburg syndrome 1)
prdx5	0.1878	Peroxiredoxin 5
xrcc4	0.2016	X-ray repair complementing defective repair in Chinese hamster cells 4
slc4a2	0.221	Solute carrier family 4, anion exchanger, member 2 (erythrocyte membrane protein band 3-like 1)
ptch	0.2276	Patched homolog (Drosophila)
sprr3	0.2414	Small proline-rich protein 3

Table 1: List of most significant genes identified in the CEPH sample, ordered by p-values. 24 genes are significant at $p < 0.05$ (7 expected).
