# Supplementary material for: Possible Ancestral Structure in Human Populations
Source: PLoS Genet. 2006 Jul 28;2(7):e105. doi: 10.1371/journal.pgen.0020105 (PMC1523253; doi:10.1371/journal.pgen.0020105)
Supplement: Table S2 — (29 KB PDF) [file pgen.0020105.st002.pdf]

Yoruba

Name	p-value	Function
xrcc4	0.0012	X-ray repair complementing defective repair in Chinese hamster cells 4
tjp1	0.0052	Tight junction protein 1 (zona occludens 1)
dut	0.009	DUTP pyrophosphatase
oxsr1	0.0188	Oxidative-stress responsive 1
tert	0.0252	Telomerase reverse transcriptase
tpo	0.0298	Thyroid peroxidase
prdx2	0.0304	Peroxiredoxin 2
abcb1	0.04	ATP-binding cassette, sub-family B (MDR/TAP), member 1
angptl7	0.046	Angiopoietin-like 7
rpa3	0.0486	Replication protein A3, 14kDa
ctnna1	0.05	Catenin (cadherin-associated protein), alpha 1, 102kDa
fmo4	0.0532	Flavin containing monooxygenase 4
map2k4	0.0558	Mitogen-activated protein kinase kinase 4
slc4a2	0.056	Solute carrier family 4, anion exchanger, member 2 (erythrocyte membrane protein band 3-like 1)
rad18	0.06	RAD18 homolog (S. cerevisiae)
msr1	0.063	Macrophage scavenger receptor 1
csk	0.0634	C-src tyrosine kinase
odc1	0.0664	Ornithine decarboxylase 1
prdx5	0.0744	Peroxiredoxin 5
foxm1	0.0746	Forkhead box M1
fancf	0.0778	Fanconi anemia, complementation group F
app	0.0804	Amyloid beta (A4) precursor protein (protease nexin-II, Alzheimer disease)
tuba1	0.083	Tubulin, alpha 1 (testis specific)
abl1	0.0832	V-abl Abelson murine leukemia viral oncogene homolog 1
ddb1	0.086	Damage-specific DNA binding protein 1, 127kDa
eno1	0.0882	Enolase 1, (alpha)
calca	0.100	Calcitonin/calcitonin-related polypeptide, alpha
capn3	0.103	Calpain 3, (p94)
pold4	0.112	Polymerase (DNA-directed), delta 4
polm	0.1182	Polymerase (DNA directed), mu
recql4	0.119	RecQ protein-like 4
trpm2	0.119	Transient receptor potential cation channel, subfamily M, member 2
snca	0.121	Synuclein, alpha (non A4 component of amyloid precursor)
oxr1	0.1232	Oxidation resistance 1
cdc37	0.1266	CDC37 cell division cycle 37 homolog (S. cerevisiae)
gpx6	0.128	Glutathione peroxidase 6 (olfactory)
vnn1	0.136	Vanin 1
sphar	0.1458	S-phase response (cyclin-related)
blm	0.146	Bloom syndrome
aoc3	0.1488	Amine oxidase, copper containing 3 (vascular adhesion protein 1)
fdxr	0.1546	Ferredoxin reductase
mmp16	0.155	Matrix metalloproteinase 16 (membrane-inserted)
txnrd1	0.1626	Thioredoxin reductase 1
mlh3	0.1674	MutL homolog 3 (E. coli)
h2afx	0.1696	H2A histone family, member X
xdh	0.1856	Xanthine dehydrogenase
tbxa2r	0.1864	Thromboxane A2 receptor
abl2	0.1898	V-abl Abelson murine leukemia viral oncogene homolog 2 (arg, Abelson-related gene)
sprr3	0.1916	Small proline-rich protein 3
nos2a	0.1934	Nitric oxide synthase 2A (inducible, hepatocytes)

Table 1: List of most significant genes identified in the Yoruba sample, ordered by p-values. We find a significant deviation from the expected distribution of p-values (27 genes at $p < 0.1$ for 13 expected).
